# Development of Zeise’s Salt Derivatives Bearing Substituted Acetylsalicylic Acid Substructures as Cytotoxic COX Inhibitors

**DOI:** 10.3390/pharmaceutics15061573

**Published:** 2023-05-23

**Authors:** Alexander Weninger, Jessica Sagasser, Victoria Obermoser, Josef Egger, Susanna Wisboeck, Qianqian Qiu, Miriam Ladstaetter, Andrea Cucchiaro, Klaus Wurst, Daniel Baecker, Ronald Gust

**Affiliations:** 1 Department of Pharmaceutical Chemistry, Institute of Pharmacy, CMBI—Center for Molecular Biosciences Innsbruck, CCB—Center for Chemistry and Biomedicine, University of Innsbruck, Innrain 80-82, 6020 Innsbruck, Austria; 2 Department of General, Inorganic and Theoretical Chemistry, University of Innsbruck, Innrain 80-82, 6020 Innsbruck, Austria; 3 Department of Pharmaceutical and Medicinal Chemistry, Institute of Pharmacy, University of Greifswald, Friedrich-Ludwig-Jahn-Straße 17, 17489 Greifswald, Germany

**Keywords:** anticancer, antiproliferative activity, capillary electrophoresis, chlorination, COX-inhibition, cyclooxygenase enzyme, fluorination, methylation, platinum, Zeise’s salt

## Abstract

Zeise’s salt derivatives of the potassium trichlorido[η^2^-((prop-2-en/but-3-en)-1-yl)-2-acetoxybenzoate]platinate(II) type (ASA-Prop-PtCl_3_/ASA-But-PtCl_3_ derivatives) were synthesized and characterized regarding their structure, stability, and biological activity. It is proposed that the leads ASA-Prop-PtCl_3_ and ASA-But-PtCl_3_ interfere with the arachidonic acid cascade as part of their mode of action to reduce the growth of COX-1/2-expressing tumor cells. With the aim to increase the antiproliferative activity by strengthening the inhibitory potency against COX-2, F, Cl, or CH_3_ substituents were introduced into the acetylsalicylic acid (ASA) moiety. Each structural modification improved COX-2 inhibition. Especially compounds with F substituents at ASA-But-PtCl_3_ reached the maximum achievable inhibition of about 70% already at 1 µM. The PGE_2_ formation in COX-1/2-positive HT-29 cells was suppressed by all F/Cl/CH_3_ derivatives, indicating COX inhibitory potency in cellular systems. The CH_3_-bearing complexes showed the highest cytotoxicity in COX-1/2-positive HT-29 cells with IC_50_ values of 16–27 µM. In COX-negative MCF-7 cells, they were 2–3-fold less active. These data clearly demonstrate that it is possible to increase the cytotoxicity of ASA-Prop-PtCl_3_ and ASA-But-PtCl_3_ derivatives by enhancing COX-2 inhibition.

## 1. Introduction

The enzymes cyclooxygenase-1 and -2 (COX-1/2) catalyze the synthesis of prostaglandins (PGs) via the conversion of arachidonic acid [1,2]. The COX-1 isoenzyme is constitutively expressed in nearly all tissues and is responsible for basal PG synthesis, which is required for platelet activity, regulation of peripheral vascular resistance, reproduction, or the production of the protective gastric mucosa [3].

COX-2 is the inducible isoform, and cytokines, growth factors, mitogens, endotoxins, or tumor promotors stimulate its expression [1,2]. Various cancer types overexpress COX-2, which raises the content of PGs in tumor tissue. Especially PGE_2_ induces proliferation, inhibits apoptosis, initiates angiogenesis, suppresses the immune response, or forces tumor aggressiveness and fast tumor growth [4]. Reduction of the PGE_2_ level through COX-2 inhibition is therefore discussed as a possible mode of action for the design of new antitumor drugs.

Interestingly, there is evidence that COX-1 plays a pivotal role in some tumors, and COX-1 and COX-2 operate in a coordinative manner [5]. The ability of COX-1 inhibitors to arrest cell growth and cause poptosis has already been demonstrated [6]. Furthermore, simultaneous drug-induced reduction of the COX-1 and COX-2 activity has superior effects on the growth of tumor cells in vitro [7] and in vivo [8]. Therefore, the development of metal complexes targeting both isoenzymes presents an appropriate drug design.

**Figure 1:**
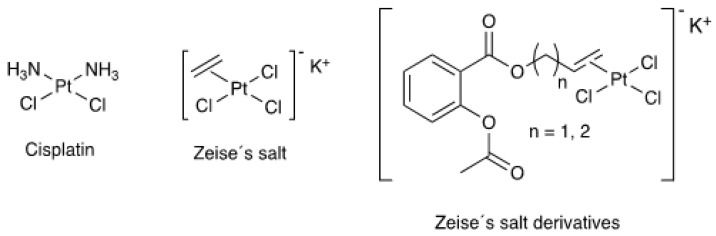
Chemical formulae of Cisplatin, Zeise’s salt, and Zeise’s salt derivatives ASA-Prop-PtCl_3_ (n = 1) and ASA-But-PtCl_3_ (n = 2).

The most common metal complex for cancer therapy is Cisplatin (Figure 1). As part of its mode of action, the formation of intrastrand crosslinks at the DNA is well accepted [9,10]. Unfortunately, selectivity for tumor cells is not given, leading to severe side effects, which are often the limiting factors in cancer therapy with Cisplatin [9]. In addition, resistance frequently occurs during therapy [11]. For these reasons, the development of new platinum-based drugs with a distinct mode of action is desirable.

We demonstrated in a previous study that it is possible to optimize Zeise’s salt (Figure 1), which is not therapeutically used as an antitumor agent [12]. The ethylene of Zeise’s salt represents a non-leaving group that can be easily linked to an active drug to obtain a multitargeting compound.

Acetylsalicylic acid (ASA), a well-known non-steroidal anti-inflammatory drug, demonstrated chemo-preventative and adjuvant chemotherapeutic properties [13,14] and was already derived successfully as a ligand for the development of cytotoxic metal-organic compounds [12,15,16,17,18,19].

Esterification of ASA with prop-2-en-1-ol or but-3-en-1-ol allows the coordination to platinum(II) via the alkene comparable to Zeise’s salt. The products ASA-Prop-PtCl_3_ and ASA-But-PtCl_3_ (Figure 1) showed low growth inhibitory potential in COX-1/2-positive HT-29 colon carcinoma cells and were nearly inactive in COX-negative MCF-7 breast cancer cells. Interference with the arachidonic acid cascade was confirmed, but with preferential binding to COX-1 [12].

Continuing this study, we investigated the possibility of enhancing the cytotoxic effects in HT-29 cells by blocking COX-2 to a greater extent. Inhibition of COX-2-mediated PGE_2_ synthesis should decrease the intracellular level and significantly reduce the growth-promoting effects.

We used a drug design that exploits the larger binding site in COX-2 [20,21], which was previously followed in our approach to optimize [(prop-2-ynyl)-2-acetoxybenzoate]dicobalthexacarbonyl as a COX-binding cytostatic metal-organic compound [16,22,23,24].

**Scheme 1:**
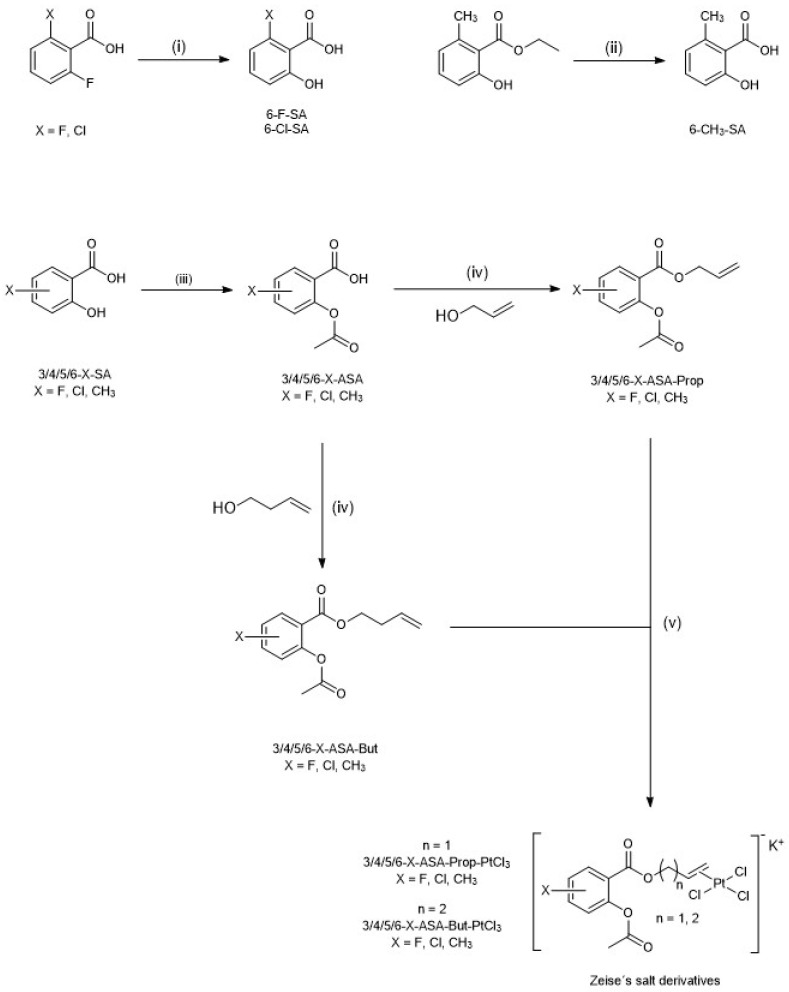
Synthesis of the ligands and the corresponding Zeise’s salt derivatives. Reagents and conditions: (**i**) NaOH, DMSO, 130 °C, 12 h; (**ii**) NaOH, THF:MeOH:H_2_O (3:1:1), 70 °C, 6 h; (**iii**) Ac_2_O, TEA, dry THF, 0 °C-room temperature, 12 h; (**iv**) DCC, DMAP, dry CH_2_Cl_2_, 0 °C-room temperature, 3.5 h; (**v**) Zeise’s salt, dry EtOH, 48 °C, 3.5 h.

For this purpose, the ASA moiety of ASA-Prop-PtCl_3_ as well as of ASA-But-PtCl_3_ was equipped with a F, Cl, or CH_3_ substituent at the position 3, 4, 5, or 6 of the aromatic ring (Scheme 1). The newly synthesized platinum complexes were tested for stability, inhibitory effects on the isolated COX isoenzymes, and cytotoxicity in COX-1/2-positive HT-29 and COX-1/2-negative MCF-7 cells. Furthermore, COX inhibition was determined in HT-29 cells to achieve a possible correlation with the potency to inhibit cell growth.

## 2. Materials and Methods

### 2.1. General Material and Methods

All solvents and chemicals were commercially obtained from Alfa Aesar (Haverhill, MA, USA), Euriso-Top (Saarbrücken, Germany), Fluka (Buchs, Switzerland), Sigma-Aldrich (Waltham, MA, USA), and TCI Chemicals (Tokyo, Japan). They were used as delivered without purification. Solvents were gained in suitable purity or underwent distillation prior to use. Milli-Q water was retrieved from a Milli-Q Gradient A10 water purification system (Merck Millipore, Billerica, MA, USA). The reactions were monitored by thin layer chromatography (TLC) on silica gel plates 60 F_254_ (Merck, Darmstadt, Germany) and visualized by observation under UV light at 254 and/or 365 nm. Silica gel 60 (particle size: 40–63 µm) was employed for the conduction of column chromatography.

Capillary electrophoresis (CE) experiments to determine the purity of the platinum complexes were performed on a 3D-CE system from Agilent (Santa Clara, CA, USA) equipped with a temperature-controlled autosampler, a diode array detector, and a column compartment. Fused-silica capillaries (75 µm inner diameter; 56 cm effective length, 64.5 cm total length) from Agilent were obtained from VWR (Vienna, Austria). All final products had a purity higher than 95%, as assessed by CE (Figures S1–S24).

^1^H and ^13^C{^1^H}, [^1^H,^1^H]-COSY, [^1^H,^13^C]-HMBC, and [^1^H,^13^C]-HSQC nuclear magnetic resonance (NMR) spectra were recorded on a 200 MHz Gemini (now Agilent) or a Bruker Avance 4 Neo spectrometer, operating at 400 MHz (^1^H) or 101 MHz (^13^C{^1^H}), respectively. Chemical shifts (given in parts per million, ppm) were referenced using the center of the internal residual peak of the solvent (acetone-*d_6_*) multiplet. The latter was referred to tetramethylsilane (TMS) as δ = 2.05 (^1^H) and δ = 29.84 (^13^C{^1^H}). The assignment of the chemical shifts to the respective H and C atoms was performed according to the labeling as shown in Chart 1. Coupling constants are given in Hertz (Hz). The downfield shifted proton (subscript α) or the upfield shifted proton (subscript β) of a diastereotopically split methylene moiety are indicated by the subscripts α and β (Figures S25–S72). An Orbitrap Elite mass spectrometer (Thermo Fisher Scientific, Waltham, MA, USA) was used to conduct high-resolution electrospray-ionization mass spectrometry (HR-ESI-MS) analyses. The peaks of greatest intensity out of the isotopic pattern are presented (Figures S73–S95). The absorbance measurements within the biological assays were performed with an Enspire multimodal plate reader (Perkin Elmer Life Sciences, Waltham, MA, USA).

### 2.2. General Procedure for the Synthesis of Zeise’s Salt Derivatives

Zeise’s salt monohydrate (0.20 mmol) dissolved in 8 mL of dry ethanol (EtOH), which was degassed (three cycles of freeze-pump-thaw), and stirred at room temperature under protection from light and under an argon atmosphere. Then, 0.24 mmol of the respective ligand (1.2 eq.), solved in about 2 mL of dry and degassed EtOH, was added drop by drop with a syringe. Next, the mixture underwent stirring at 48 °C for 3.5 h, followed by cooling to room temperature. The solution was filtered and evaporated to dryness. The recrystallization of the crude product from EtOH/diethyl ether or EtOH/diisopropyl ether afforded pure solid products.

Potassium trichlorido[η^2^-(prop-2-en-1-yl)-2-acetoxy-3-fluorobenzoate]platinate(II) (3-F-ASA-Prop-PtCl_3_). Yellow powder; yield: 71%. ^1^H NMR (400 MHz, acetone-d_6_): δ = 7.91 (ddd, ^3^J = 7.9 Hz, ^4^J = 1.5 Hz, ^5^J_H-F_ = 1.5 Hz, 1H, Ar-H6), 7.54 (ddd, ^3^J_H-F_ = 9.9 Hz, ^3^J = 8.3 Hz, ^4^J = 1.6 Hz, 1H, Ar-H4), 7.43 (ddd, ^3^J = 8.1 Hz, ^3^J = 8.1 Hz, ^4^J_H-F_ = 5.1 Hz, 1H, Ar-H5), 4.99 (dddd, ^2^J_H-Pt_ = 59 Hz, ^3^J = 12.6 Hz, ^3^J = 9.0 Hz, ^3^J = 6.5 Hz, ^3^J = 6.5 Hz, 1H, -CH=), 4.91 (dd, ^3^J_H-Pt_ = 35 Hz, ^2^J = 11.9 Hz, ^3^J = 5.8 Hz, 1H, -OC**H**_α_H_β_-), 4.50 (dd, ^3^J_H-Pt_ = 38 Hz, ^2^J = 11.9 Hz, ^3^J = 7.3 Hz, 1H, -OCH_α_**H**_β_-), 4.34 (dd, ^2^J_H-Pt_ = 74 Hz, ^3^J = 12.4 Hz, ^2^J = 1.2 Hz, 1H, =CH_2, trans_), 4.30 (d, ^2^J_H-Pt_ = 59 Hz, ^3^J = 7.9 Hz, ^2^J = 1.0 Hz, 1H, =CH_2,cis_), 2.40 (s, 3H, -OAc). ^13^C NMR (101 MHz, acetone-d_6_): δ = 168.04 (-(**C**=O)-CH_3_), 163.31 (d, ^4^J_C-F_ = 3.1 Hz, Ar-(**C**=O)), 154.94 (d, ^1^J_C-F_ = 247.1 Hz, C3), 138.55 (d, ^2^J_C-F_ = 14.8 Hz, C2), 126.95 (d, ^4^J_C-F_ = 3.6 Hz, C6), 126.79 (d, ^3^J_C-F_ = 7.7 Hz, C5), 126.00 (C1), 120.48 (d, ^2^J_C-F_ = 19.1 Hz, C4), 76.43 (C2′), 64.85 (C1′), 64.12 (C3′), 19.85 (-OAc). HR-ESI-MS (*m*/*z*): 539.9333 ([M-K]^−^, calcd for C_12_H_11_Cl_3_FO_4_Pt: 539.9320). CE-purity: 95.2%.

Potassium trichlorido[η^2^-(prop-2-en-1-yl)-2-acetoxy-4-fluorobenzoate]platinate(II) (4-F-ASA-Prop-PtCl_3_). Yellow powder; yield: 70%. ^1^H NMR (400 MHz, acetone-*d_6_*): δ = 8.19 (dd, ^3^J = 8.8 Hz, ^4^J_H-F_ = 6.5 Hz, 1H, Ar-H6), 7.21 (ddd, ^3^J = 8.8 Hz, ^3^J_H-F_ = 8.1 Hz, ^4^J = 2.6 Hz, 1H, Ar-H5), 7.07 (dd, ^3^J_H-F_ = 9.3 Hz, ^4^J = 2.6 Hz, 1H, Ar-H3), 4.99 (dddd, ^2^J_H-Pt_ = 61 Hz, ^3^J = 12.9 Hz, ^3^J = 7.6 Hz, ^3^J = 7.6 Hz, ^3^J = 5.7 Hz, 1H, -CH = ), 4.88 (dd, ^3^J_H-Pt_ = 30 Hz, ^2^J = 11.9 Hz, ^3^J = 5.7 Hz, 1H, -OC**H**_α_H_β_-), 4.48 (dd, ^2^J = 12.0 Hz, ^3^J = 7.5 Hz, 1H, -OCH_α_**H**_β_-), 4.33 (dd, ^2^J_H-Pt_ = 72 Hz, ^3^J = 12.7 Hz, ^2^J = 1.3 Hz, 1H, =CH_2,_ *_trans_*), 4.30 (ddd, ^2^J_H-Pt_ = 60 Hz, ^3^J = 7.9 Hz, ^2^J = 1.3 Hz, ^4^J = 0.5 Hz, 1H, =CH_2,_ *_cis_*), 2.34 (s, 3H, -OAc). ^13^C NMR (101 MHz, acetone-*d_6_*): δ = 169.54 (-(**C**=O)-CH_3_), 166.11 (d, ^1^J_C-F_ = 253.4 Hz, C4), 164.12 (Ar-(**C**=O)), 153.42 (d, ^3^J_C-F_ = 11.7 Hz, C2), 134.81 (d, ^3^J_C-F_ = 10.3 Hz, C6), 121.31 (d, ^4^J_C-F_ = 3.7 Hz, C1), 113.96 (d, ^2^J_C-F_ = 21.7 Hz, C5), 112.50 (d, ^2^J_C-F_ = 24.6 Hz, C3), 77.44 (C3′), 65.48 (C1′), 65.02 (C2′), 21.18 (-OAc). HR-ESI-MS (*m*/*z*): 537.9390 ([M-K]^−^, calcd for C_12_H_11_Cl_3_FO_4_Pt: 537.9349). CE-purity: 97.1%.

Potassium trichlorido[η^2^-(prop-2-en-1-yl)-2-acetoxy-5-fluorobenzoate]platinate(II) (5-F-ASA-Prop-PtCl_3_). Yellow powder; yield: 75%. ^1^H NMR (400 MHz, acetone-*d_6_*): δ = 7.81 (dd, ^3^J_H-F_ = 9.0 Hz, ^4^J = 3.2 Hz, 1H, Ar-H6), 7.44 (ddd, ^3^J = 8.9 Hz, ^3^J_H-F_ = 7.7 Hz, ^4^J = 3.2 Hz, 1H, Ar-H4), 7.25 (dd, ^3^J = 8.9 Hz, ^4^J_H-F_ = 4.7 Hz, 1H, Ar-H3), 4.99 (dddd, ^2^J_H-Pt_ = 68 Hz, ^3^J = 12.7 Hz, ^3^J = 7.6 Hz, ^3^J = 7.6 Hz, ^3^J = 5.6 Hz, 1H, -CH=), 4.90 (dd, ^3^J_H-Pt_ = 34 Hz, ^2^J = 11.9 Hz, ^3^J = 5.7 Hz, 1H, -OC**H**_α_H_β_-), 4.50 (dd, ^3^J_H-Pt_ = 40 Hz, ^2^J = 11.9 Hz, ^3^J = 7.5 Hz, 1H, -OCH_α_**H**_β_-), 4.34 (dd, ^2^J_H-Pt_ = 74 Hz, ^3^J = 12.6 Hz, ^2^J = 1.2 Hz, 1H, =CH_2,_ *_trans_*), 4.31 (dd, ^2^J_H-Pt_ = 60 Hz, ^3^J = 7.9 Hz, ^2^J = 0.9 Hz, 1H, =CH_2, *cis*_), 2.33 (s, 3H, -OAc). ^13^C NMR (101 MHz, acetone-*d_6_*): δ = 169.92 (-(**C**=O)-CH_3_), 163.97 (d, ^4^J_C-F_ = 2.3 Hz, Ar-(**C**=O)), 160.50 (d, ^1^J_C-F_ = 244.1 Hz, C5), 147.69 (d, ^4^J_C-F_ = 2.9 Hz, C2), 126.80 (d, ^3^J_C-F_ = 8.5 Hz, C3), 126.17 (d, ^3^J_C-F_ = 7.4 Hz, C1), 121.37 (d, ^2^J_C-F_ = 23.3 Hz, C4), 118.67 (d, ^2^J_C-F_ = 25.4 Hz, C6), 77.17 (C3′), 65.76 (C1′), 65.13 (C2′), 21.15 (-OAc). HR-ESI-MS (*m*/*z*): 538.9330 ([M-K]^−^, calcd for C_12_H_11_Cl_3_FO_4_Pt: 538.9299). CE-purity: 95.8%.

Potassium trichlorido[η^2^-(prop-2-en-1-yl)-2-acetoxy-6-fluorobenzoate]platinate(II) (6-F-ASA-Prop-PtCl_3_). Light yellow crystals; yield: 79%. ^1^H NMR (400 MHz, acetone-*d_6_*): δ = 7.60 (ddd, ^3^J = 8.4 Hz, ^3^J = 8.4 Hz, ^4^J_H-F_ = 6.2 Hz, 1H, Ar-H4), 7.18 (ddd, ^3^J_H-F_ = 9.6 Hz, ^3^J = 8.5 Hz, ^4^J = 1.0 Hz, 1H, Ar-H5), 7.09 (ddd, ^3^J = 8.2 Hz, ^4^J = 1.0 Hz, ^5^J_H-F_ = 1.0 Hz, 1H, Ar-H3), 5.11-4.80 (m, 2H, -CH= and -OC**H**_α_H_β_-), 4.65-4.53 (m, ^3^J_H-Pt_ = 41 Hz, 1H, -OCH_α_**H**_β_-), 4.46-4.21 (m, ^2^J_H-Pt_ = 70 Hz, 2H, =CH_2_), 2.31 (s, 3H, -CH_3_). ^13^C NMR (101 MHz, acetone-*d_6_*): δ = 169.28 (-(**C**=O)-CH_3_), 162.66 (Ar-(**C**=O)), 161.21 (d, ^1^J_C-F_ = 254.1 Hz, C6), 150.67 (d, ^3^J_C-F_ = 5.2 Hz, C2), 133.37 (d, ^3^J_C-F_ = 10.3 Hz, C4), 120.36 (d, ^4^J_C-F_ = 3.6 Hz, C3), 116.64 (d, ^2^J_C-F_ = 17.6 Hz, C1), 114.38 (d, ^2^J_C-F_ = 21.9 Hz, C5), 76.51 (C2′), 65.79 (C1′), 65.27 (C3′), 21.03 (-OAc). HR-ESI-MS (*m*/*z*): 538.9330 ([M-K]^−^, calcd for C_12_H_11_Cl_3_FO_4_Pt: 538.9299). CE-purity: 95.5%.

Potassium trichlorido[η^2^-(but-3-en-1-yl)-2-acetoxy-3-fluorobenzoate]platinate(II) (3-F-ASA-But-PtCl_3_). Yellow powder; yield: 29%. ^1^H NMR (400 MHz, acetone-*d_6_*): δ = 7.90 (ddd, ^3^J = 7.9 Hz, ^4^J = 1.5 Hz, ^5^J_H-F_ = 1.5 Hz, 1H, Ar-H6), 7.53 (ddd, ^3^J_H-F_ = 9.9 Hz, ^3^J = 8.3 Hz, ^4^J = 1.6 Hz, 1H, Ar-H4), 7.43 (ddd, ^3^J = 8.1 Hz, ^3^J = 8.1 Hz, ^4^J_H-F_ = 5.1 Hz, 1H, Ar-H5), 5.00 (dddd, ^2^J_H-Pt_ = 66 Hz, ^3^J = 13.7 Hz, ^3^J = 7.9 Hz, ^3^J = 6.2 Hz, ^3^J = 6.2 Hz, 1H, -CH=), 4.74-4.61 (m, 2H, -OCH_2_-), 4.36-4.11 (m, ^2^J_H-Pt_ = 60 Hz, 2H, =CH_2_), 2.58 (ddt, ^2^J = 14.2 Hz, ^3^J = 8.1 Hz, ^3^J = 6.0 Hz, 1H, -C**H**_α_H_β_-), 2.09-2.00 (m, 1H, -CH_α_**H**_β_-), 2.37 (s, 3H, -OAc). ^13^C NMR (101 MHz, acetone-*d_6_*): δ = 168.83 (-(**C**=O)-CH_3_), 164.32 (d, ^4^J_C-F_ = 3.6 Hz, Ar-(**C**=O)), 155.84 (d, ^1^J_C-F_ = 247.1 Hz, C3), 139.45 (d, ^2^J_C-F_ = 14.5 Hz, C2), 127.73 (d, ^4^J_C-F_ = 3.8 Hz, C6), 127.71 (d, ^3^J_C-F_ = 7.5 Hz, C5), 126.96 (C1), 121.31 (d, ^2^J_C-F_ = 19.6 Hz, C4), 84.00 (C3′), 65.96 (C4′), 65.27 (C1′), 33.25 (C2′), 20.61 (-OAc). HR-ESI-MS (*m*/*z*): 534.9825 ([M-K-Cl + OH]^−^, calcd for C_13_H_14_Cl_2_FO_5_Pt: 534.9846), 552.9475 ([M-K]^−^, calcd for C_13_H_13_Cl_3_FO_4_Pt: 552.9455). CE-purity: 97.8%.

Potassium trichlorido[η^2^-(but-3-en-1-yl)-2-acetoxy-4-fluorobenzoate]platinate(II) (4-F-ASA-But-PtCl_3_). Yellow powder; yield: 85%. ^1^H NMR (400 MHz, acetone-*d_6_*): δ = 8.20 (dd, ^3^J = 8.9 Hz, ^4^J_H-F_ = 6.5 Hz, 1H, Ar-H6), 7.19 (ddd, ^3^J = 8.9 Hz, ^3^J_H-F_ = 8.1 Hz, ^4^J = 2.6 Hz, 1H, Ar-H5), 7.05 (dd, ^3^J_H-F_ = 9.3 Hz, ^4^J = 2.6 Hz, 1H, Ar-H3), 5.00 (dddd, ^2^J_H-Pt_ = 66 Hz, ^3^J = 12.0 Hz, ^3^J = 9.1 Hz, ^3^J = 8.0 Hz, ^3^J = 6.1 Hz, 1H, -CH=), 4.71-4.58 (m, 2H, -OCH_2_-), 4.37-4.11 (m, ^2^J_H-Pt_ = 60 Hz, 2H, =CH_2_), 2.57 (ddt, ^2^J = 14.2 Hz, ^3^J = 8.1 Hz, ^3^J = 6.1 Hz, 1H, -C**H**_α_H_β_-), 2.09-1.98 (m, 1H, -CH_α_**H**_β_-), 2.31 (s, 3H, -OAc). ^13^C NMR (101 MHz, acetone-*d_6_*): δ = 169.46 (-(**C**=O)-CH_3_), 166.06 (d, ^1^J_C-F_ = 252.9 Hz, C4), 164.24 (Ar-(**C**=O)), 153.47 (d, ^3^J_C-F_ = 12.3 Hz, C2), 134.76 (d, ^3^J_C-F_ = 10.6 Hz, C6), 121.35 (d, ^4^J_C-F_ = 3.7 Hz, C1), 113.96 (d, ^2^J_C-F_ = 21.3 Hz, C5), 112.48 (d, ^2^J_C-F_ = 24.1 Hz, C3), 84.26 (C3′), 65.96 (C4′), 64.97 (C1′), 33.25 (C2′), 21.07 (-OAc). HR-ESI-MS (*m*/*z*): 552.9525 ([M-K]^−^, calcd for C_13_H_13_Cl_3_FO_4_Pt: 552.9505). CE-purity: 96.9%.

Potassium trichlorido[η^2^-(but-3-en-1-yl)-2-acetoxy-5-fluorobenzoate]platinate(II) (5-F-ASA-But-PtCl_3_). Yellow powder; yield: 51%. ^1^H NMR (400 MHz, acetone-*d_6_*): δ = 7.78 (dd, ^3^J_H-F_ = 8.9 Hz, ^4^J = 3.2 Hz, 1H, Ar-H6), 7.43 (ddd, ^3^J = 9.0 Hz, ^3^J_H-F_ = 7.8 Hz, ^4^J = 3.2 Hz, 1H, Ar-H4), 7.25 (dd, ^3^J = 8.9 Hz, ^4^J_H-F_ = 4.7 Hz, 1H, Ar-H3), 5.00 (dddd, ^2^J_H-Pt_ = 64 Hz, ^3^J = 13.7 Hz, ^3^J = 8.0 Hz, ^3^J = 8.0 Hz, ^3^J = 6.1 Hz, 1H, -CH=), 4.73-4.62 (m, 2H, -OCH_2_-), 4.35-4.12 (m, ^2^J_H-Pt_ = 63 Hz, 2H, =CH_2_), 2.56 (ddt, ^2^J = 14.3 Hz, ^3^J = 8.1 Hz, ^3^J = 6.1 Hz, 1H, -C**H**_α_H_β_-), 2.09-1.99 (m, 1H, -CH_α_**H**_β_-), 2.30 (s, 3H, -OAc). ^13^C NMR (101 MHz, acetone-*d_6_*): δ = 169.86 (-(**C**=O)-CH_3_), 164.10 (d, ^4^J_C-F_ = 2.2 Hz, Ar-(**C**=O)), 160.50 (d, ^1^J_C-F_ = 245.0 Hz, C5), 147.72 (d, ^4^J_C-F_ = 2.9 Hz, C2), 126.81 (d, ^3^J_C-F_ = 8.5 Hz, C3), 126.23 (d, ^3^J_C-F_ = 7.7 Hz, C1), 121.32 (d, ^2^J_C-F_ = 23.5 Hz, C4), 118.53 (d, ^2^J_C-F_ = 25.5 Hz, C6), 83.90 (C3′), 65.91 (C4′), 65.33 (C1′), 33.21 (C2′), 21.04 (-OAc). HR-ESI-MS (*m*/*z*): 552.9474 ([M-K]^−^, calcd for C_13_H_13_Cl_3_FO_4_Pt: 552.9455). CE-purity: 98.0%.

Potassium trichlorido[η^2^-(but-3-en-1-yl)-2-acetoxy-6-fluorobenzoate]platinate(II) (6-F-ASA-But-PtCl_3_). Yellow powder; yield: 84%. ^1^H NMR (400 MHz, acetone-*d_6_*): δ = 7.60 (ddd, ^3^J = 8.4 Hz, ^3^J = 8.4 Hz, ^4^J_H-F_ = 6.2 Hz, 1H, Ar-H4), 7.18 (ddd, ^3^J_H-F_ = 9.5 Hz, ^3^J = 8.5 Hz, ^4^J = 1.0 Hz, 1H, Ar-H5), 7.09 (ddd, ^3^J = 8.3 Hz, ^4^J = 1.0 Hz, ^5^J_H-F_ = 1.0 Hz, 1H, Ar-H3), 4.98 (dddd, ^2^J_H-Pt_ = 64 Hz, ^3^J = 12.0 Hz, ^3^J = 8.9 Hz, ^3^J = 7.7 Hz, ^3^J = 6.6 Hz, 1H, -CH=), 4.68 (dd, ^3^J = 7.3 Hz, ^3^J = 6.2 Hz, 2H, -OCH_2_-), 4.35-4.13 (m, ^2^J_H-Pt_ = 62 Hz, 2H, =CH_2_), 2.57 (ddt, ^2^J = 14.0 Hz, ^3^J = 7.8 Hz, ^3^J = 6.2 Hz, 1H, -C**H**_α_H_β_-), 2.09-2.00 (m, 1H, -CH_α_**H**_β_-), 2.29 (s, 3H, -OAc). ^13^C NMR (101 MHz, acetone-*d_6_*): δ = 169.24 (-(**C**=O)-CH_3_), 162.90 (Ar-(**C**=O)), 161.21 (d, ^1^J_C-F_ = 254.0 Hz, C6), 150.74 (d, ^3^J_C-F_ = 5.2 Hz, C2), 133.34 (d, ^3^J_C-F_ = 10.2 Hz, C4), 120.38 (d, ^4^J_C-F_ = 3.5 Hz, C3), 116.72 (d, ^2^J_C-F_ = 17.6 Hz, C1), 114.39 (d, ^2^J_C-F_ = 22.3 Hz, C5), 83.81 (C3′), 66.10 (C4′), 65.54 (C1′), 33.41 (C2′), 20.96 (-OAc). HR-ESI-MS (*m*/*z*): 552.9489 ([M-K]^−^, calcd for C_13_H_13_Cl_3_FO_4_Pt: 552.9455). CE-purity: 96.7%.

Potassium trichlorido[η^2^-(prop-2-en-1-yl)-2-acetoxy-3-chlorobenzoate]platinate(II) (3-Cl-ASA-Prop-PtCl_3_). Yellow powder; yield: 76%. ^1^H NMR (400 MHz, acetone-*d_6_*): δ = 8.07 (dd, ^3^J = 7.9 Hz, ^4^J = 1.6 Hz, 1H, Ar-H6), 7.78 (dd, ^3^J = 8.0 Hz, ^4^J = 1.6 Hz, 1H, Ar-H4), 7.44 (dd, ^3^J = 8.0 Hz, ^3^J = 8.0 Hz, 1H, Ar-H5), 4.99 (dddd, ^2^J_H-Pt_ = 61 Hz, ^3^J = 12.8 Hz, ^3^J = 7.5 Hz, ^3^J = 5.8 Hz, ^3^J = 5.8 Hz, 1H, -CH=), 4.90 (dd, ^3^J_H-Pt_ = 26 Hz, ^2^J = 11.9 Hz, ^3^J = 5.8 Hz, 1H, -OC**H**_α_H_β_-), 4.49 (dd, ^3^J_H-Pt_ = 32 Hz, ^2^J = 11.9 Hz, ^3^J = 7.3 Hz, 1H, -OCH_α_**H**_β_-), 4.34 (dd, ^2^J_H-Pt_ = 59 Hz, ^3^J = 12.6 Hz, ^2^J = 1.3 Hz, 1H, =CH_2, *trans*_), 4.30 (d, ^2^J_H-Pt_ = 59 Hz, ^3^J = 7.9 Hz, ^2^J = 1.1 Hz, 1H, =CH_2, *cis*_), 2.40 (s, 3H, -OAc). ^13^C NMR (101 MHz, acetone-*d_6_*): δ = 168.70 (-(**C**=O)-CH_3_), 164.19 (Ar-(**C**=O)), 147.85 (C2), 134.96 (C4), 131.35 (C6), 129.41 (C3), 127.80 (C5), 126.76 (C1), 77.34 (C2′), 66.09 (C3′), 65.79 (C1′), 20.84 (-OAc). HR-ESI-MS (*m/z*): 555.9045 ([M-K]^−^, calcd for C_12_H_11_Cl_4_O_4_Pt: 555.9024. CE-purity: 95.2%.

Potassium trichlorido[η^2^-(prop-2-en-1-yl)-2-acetoxy-4-chlorobenzoate]platinate(II) (4-Cl-ASA-Prop-PtCl_3_). Yellow powder; yield: 88%. ^1^H NMR (200 MHz, acetone-*d_6_*): 8.12 (d, ^3^J = 8.8 Hz, 1H, Ar-H6), 7.46 (dd, ^3^J = 8.4 Hz, ^4^J = 2.2 Hz, 1H, Ar-H5), 7.32 (d, ^4^J = 2.2 Hz, 1H, Ar-H3), 5.05–4.93 (m, 1H, -CH=CH_2_), 4.93–4.84 (m, 1H, CH_2_-CH=), 4.53–4.44 (m, 1H, CH_2_-CH=), 4.36–4.27 (m, 2H, CH=CH_2_), 2.34 (s, 3H, -CH_3_). ^13^C NMR (101 MHz, acetone-*d_6_*): δ = 169.60 (-(**C**=O)-CH_3_), 164.20 (Ar-(**C**=O)), 152.33 (C2), 139.39 (C4), 133.95 (C6), 127.10 (C5), 125.19 (C3), 123.58 (C1), 77.31 (C2′), 66.10 (C1′), 65.04 (C3′), 21.07 (-OAc). HR-ESI-MS (*m*/*z*): 555.9036 ([M-K]^−^, calcd for C_12_H_11_Cl_4_O_4_Pt: 555.9024). CE-purity: 95.6%.

Potassium trichlorido[η^2^-(prop-2-en-1-yl)-2-acetoxy-5-chlorobenzoate]platinate(II) (5-Cl-ASA-Prop-PtCl_3_). Yellow powder; yield: 53%. ^1^H NMR (400 MHz, acetone-*d_6_*): 8.13 (d, ^3^J = 8.4 Hz, 1H, Ar-H3), 7.47 (dd, ^3^J = 8.4 Hz, 4J = 2.2 Hz, 1H, Ar-H4), 7.32 (d, ^3^J = 2.2 Hz, 1H, Ar-H6), 5.06–4.93 (m, 1H, -CH=CH_2_), 4.93–4.80 (m, 1H, -CH_2_-CH), 4.56–4.44 (m, 1H, -CH_2_-CH), 4.36–4.28 (m, 2H, -CH=CH_2_), 2.34 (s, 3H, -CH_3_) δ = 8.06 (d, ^4^J = 2.7 Hz, 1H, Ar-H6), 7.68 (dd, ^3^J = 8.7 Hz, ^4^J = 2.7 Hz, 1H, Ar-H4), 7.25 (d, ^3^J = 8.6 Hz, 1H, Ar-H3), 4.99 (dddd, ^2^J_H-Pt_ = 64 Hz, ^3^J = 12.8 Hz, ^3^J = 7.8 Hz, ^3^J = 7.5 Hz, ^3^J = 5.5 Hz, 1H, -CH=CH_2_), 4.91 (dd, ^2^J = 11.8 Hz, ^3^J = 5.6 Hz, 1H, -OC**H**_α_H_β-_), 4.50 (dd, ^2^J = 11.9 Hz, ^3^J = 7.5 Hz, 1H, -OCH_α_**H**_β_-), 4.35 (dd, ^3^J = 12.5 Hz, ^2^J = 1.3 Hz, 1H, =CH_2, *trans*_), 4.31 (dd, ^3^J = 7.8 Hz, ^2^J = 1.3 Hz, 1H, =CH_2, *cis*_), 2.34 (s, 3H, -CH_3_). ^13^C NMR (101 MHz, acetone-*d_6_*): δ = 169.74 (-(**C**=O)-CH_3_), 163.89 (Ar-(**C**=O)), 150.23 (C2), 134.45 (C4), 131.96 (C6), 131.62 (C5), 126.77 (C3), 126.25 (C1), 77.15 (C2′), 65.78 (C3′), 65.19 (C1′), 21.17 (-OAc). HR-ESI-MS (*m*/*z*): 555.8581 ([M-K]^−^, calcd for C_12_H_11_Cl_4_O_4_Pt: 555.9024). CE-purity: 96.1%.

Potassium trichlorido[η^2^-(prop-2-en-1-yl)-2-acetoxy-6-chlorobenzoate]platinate(II) (6-Cl-ASA-Prop-PtCl_3_). Yellow powder; yield: 66%. ^1^H NMR (400 MHz, acetone-*d_6_*): δ = 7.53 (dd, ^3^J = 8.2 Hz, ^3^J = 8.2 Hz, 1H, Ar-H4), 7.42 (dd, ^3^J = 8.1 Hz, ^4^J = 1.0 Hz, 1H, Ar-H5), 7.26 (dd, ^3^J = 8.2 Hz, ^4^J = 1.1 Hz, 1H, Ar-H3), 5.09–4.88 (m, 2H, -CH= and -OC**H**_α_H_β_-), 4.71–4.48 (m, 1H, -OCH_α_**H**_β_-), 4.45–4.22 (m, 2H, =CH_2_), 2.30 (s, 3H, -OAc). ^13^C NMR (101 MHz, acetone-*d_6_*): δ = 169.06 (-(**C**=O)-CH_3_), 164.35 (Ar-(**C**=O)), 149.72 (C2), 132.16 (C4), 132.05 (C6), 128.34 (C1), 127.76 (C5), 123.10 (C3), 76.52 (C2′), 65.93 and 65.29 (C1′and C3′), 20.97 (-OAc). HR-ESI-MS (*m/z*): 555.9046 ([M-K]^−^, calcd for C_12_H_11_Cl_4_O_4_Pt: 555.9024). CE-purity: 95.1%.

Potassium trichlorido[η^2^-(but-3-en-1-yl)-2-acetoxy-3-chlorobenzoate]platinate(II) (3-Cl-ASA-But-PtCl_3_). Yellow powder; yield: 40%. ^1^H NMR (400 MHz, acetone-*d_6_*): δ = 8.06 (dd, ^3^J = 7.9 Hz, ^4^J = 1.6 Hz, 1H, Ar-H6), 7.77 (dd, ^3^J = 8.1 Hz, ^4^J = 1.6 Hz, 1H, Ar-H4), 7.43 (dd, ^3^J = 8.0 Hz, ^3^J = 8.0 Hz, 1H, Ar-H5), 5.14–4.88 (m, ^2^J_H-Pt_ = 67 Hz, 1H, -CH=), 4.74–4.60 (m, 2H, -OCH_2_-), 4.37–4.11 (m, ^2^J_H-Pt_ = 71 Hz, 2H, =CH_2_), 2.57 (ddt, ^2^J = 14.3 Hz, ^3^J = 8.1 Hz, ^3^J = 6.0 Hz, 1H, -C**H**_α_H_β_-), 2.37 (s, 3H, -OAc), 2.08–1.99 (m, 1H, -CH_α_**H**_β_-). ^13^C NMR (101 MHz, acetone-*d_6_*): δ = 168.59 (-(**C**=O)-CH_3_), 164.31 (Ar-(**C**=O)), 147.85 (C2), 134.89 (C4), 131.23 (C6), 129.39 (C3), 127.83 (C5), 126.78 (C1), 84.18 (C3′), 66.02 (C4′), 65.29 (C1′), 33.20 (C2′), 20.70 (-OAc). HR-ESI-MS (*m*/*z*): 569.9204 ([M-K]^−^, calcd for C_13_H_13_Cl_4_O_4_Pt: 569.9181). CE-purity: 100.0%.

Potassium trichlorido[η^2^-(but-3-en-1-yl)-2-acetoxy-4-chlorobenzoate]platinate(II) (4-Cl-ASA-But-PtCl_3_). Yellow powder; yield: 92%. ^1^H NMR (200 MHz, acetone-*d_6_*): 8.14 (d, ^3^J = 8.4 Hz, 1H, Ar-H6), 7.45 (dd, ^3^J = 8.4 Hz, ^4^J = 1.8 Hz, 1H, Ar-H5), 7.31 (d, ^4^J = 2.2 Hz, 1H, Ar-H3), 5.07–4.90 (m, 1H, -CH=CH_2_), 4.65 (t, ^3^J = 6.8 Hz, 2H, O-CH_2_-CH_2_), 4.26–4.20 (m, 2H, -CH=CH_2_), 2.65–2.48 (m, 1H, -CH_2_-CH_2_-CH), 2.32 (s, 3H, -CH_3_), 2.09–1.97 (m, 1H, -CH_2_-CH_2_-CH). ^13^C NMR (101 MHz, acetone-*d_6_*): δ = 169.52 (-(**C**=O)-CH_3_), 164.31 (Ar-(**C**=O)), 152.37 (C2), 139.31 (C4), 133.91 (C6), 127.12 (C5), 125.15 (C3), 123.59 (C1), 84.22 (C2′), 65.96 (C1′), 65.10 (C4′), 33.19 (C3′), 21.05 (-OAc). HR-ESI-MS (*m*/*z*): 569.9204 ([M-K]^−^, calcd for C_13_H_13_Cl_4_O_4_Pt: 569.9181). CE-purity: 97.8%.

Potassium trichlorido[η^2^-(but-3-en-1-yl)-2-acetoxy-5-chlorobenzoate]platinate(II) (5-Cl-ASA-But-PtCl_3_).

Yellow powder; yield: 58%. ^1^H NMR (200 MHz, acetone-*d_6_*): 8.03 (d, ^4^J = 2.6 Hz, 1H, Ar-H6), 7.67 (dd, ^3^J = 8 Hz, ^4^J = 2.2 Hz, 1H, Ar-H4), 7.25 (d, ^3^J = 8.6 Hz, 1H, Ar-H3), 5.08–4.90 (m, 1H, -CH = CH_2_), 4.68 (t, ^3^J = 6.2 Hz, 2H, O-CH_2_-CH_2_), 4.26–4.21 (m, 2H, -CH=CH_2_), 2.65–2.48 (m, 1H, -CH_2_-CH_2_-CH), 2.31 (s, 3H, -CH_3_), 2.07–1.95 (m, 1 H, -CH_2_-CH_2_-CH). ^13^C NMR (101 MHz, acetone-d_6_): δ = 169.66 (-(**C**=O)-CH_3_), 164.00 (Ar-(**C**=O)), 150.23 (C2), 134.41 (C4), 131.77 (C6), 131.61 (C5), 126.77 (C3), 126.28 (C1), 84.15 (C2′), 66.05 (C1′), 65.30 (C4′), 33.20 (C3′), 21.04 (-OAc). HR-ESI-MS (*m*/*z*): 569.9193 ([M-K]^−^, calcd for C_13_H_13_Cl_4_O_4_Pt: 569.9181). CE-purity: 97.5%.

Potassium trichlorido[η^2^-(but-3-en-1-yl)-2-acetoxy-6-chlorobenzoate]platinate(II) (6-Cl-ASA-But-PtCl_3_).

Yellow powder; yield: 68%. ^1^H NMR (200 MHz, aceton-*d_6_*): 7.57–7.39 (m, 2H, Ar-H), 7.25 (dd, ^3^J = 7.6 Hz, ^4^J = 1.6 Hz, 1H, Ar-H), 5.08–4.91 (m, 1H, -CH=CH_2_), 4.71 (t, ^3^J = 6.2 Hz, 2H, O-CH_2_-CH_2_), 4.26–4.19 (m, 2H, -CH=CH_2_), 2.66–2.49 (m, 1H, -CH_2_-CH_2_-CH), 2.29 (s, 3H, -CH_3_), 2.09–1.95 (m, 1H, -CH_2_-CH_2_-CH). ^13^C NMR (101 MHz, acetone-*d_6_*): δ = 169.01 (-(**C**=O)-CH_3_), 164.56 (Ar-(**C**=O)), 149.71 (C2), 132.10 (C4), 131.99 (C6), 128.50 (C1), 127.74 (C5), 123.09 (C3), 83.85 (C2′), 66.12 (C1′), 65.76 (C4′), 33.33 (C3′), 20.93 (-OAc). HR-ESI-MS (*m/z*): 569.9202 ([M-K]^−^, calcd for C_13_H_13_Cl_4_O_4_Pt: 569.9181). CE-purity: 95.6%.

Potassium trichlorido[η^2^-(prop-2-en-1-yl)-2-acetoxy-3-methylbenzoate]platinate(II) (3-CH_3_-ASA-Prop-PtCl_3_). Yellow powder; yield: 83%. ^1^H NMR (400 MHz, acetone-*d_6_*): δ = 7.92 (dd, ^3^J = 7.9 Hz, ^4^J = 1.6 Hz, 1H, Ar-H6), 7.53 (d, ^3^J = 7.3 Hz, 1H, Ar-H4), 7.53 (dd, ^3^J = 7.7 Hz, ^3^J = 7.7 Hz, 1H, Ar-H5), 5.00 (dddd, ^2^J_H-Pt_ = 63 Hz, ^3^J = 13.2 Hz, ^3^J = 7.7 Hz, ^3^J = 5.6 Hz, ^3^J = 5.6 Hz, 1H, -CH=), 4.89 (dd, ^3^J_H-Pt_ = 34 Hz, ^2^J = 11.9 Hz, ^3^J = 5.5 Hz, 1H, -OC**H**_α_H_β_-), 4.48 (dd, ^3^J_H-Pt_ = 33 Hz, ^2^J = 12.0 Hz, ^3^J = 7.6 Hz, 1H, -OCH_α_**H**_β_-), 4.35 (d, ^2^J_H-Pt_ = 72 Hz, ^3^J = 12.8 Hz, 1H, =CH_2, *trans*_), 4.31 (d, ^2^J_H-Pt_ = 58 Hz, ^3^J = 7.9 Hz, 1H, =CH_2, *cis*_), 2.36 (s, 3H, -OAc), 2.21 (s, 3H, -CH_3_). ^13^C NMR (101 MHz, acetone-*d_6_*): δ = 169.40 (-(**C**=O)-CH_3_), 165.17 (Ar-(**C**=O)), 150.13 (C2), 136.02 (C4), 133.07 (C3), 130.24 (C6), 126.42 (C5), 124.56 (C1), 77.77 (C2′), 65.30 (C3′), 65.15 (C1′), 21.04 (-OAc), 16.14 (-CH_3_). HR-ESI-MS (*m/z*): 534.9588 ([M-K]^−^, calcd for C_13_H_14_Cl_3_O_4_Pt: 534.9575). CE-purity: 96.7%.

Potassium trichlorido[η^2^-(prop-2-en-1-yl)-2-acetoxy-4-methylbenzoate]platinate(II) (4-CH_3_-ASA-Prop-PtCl_3_). Yellow powder; yield: 75%. ^1^H NMR (400 MHz, acetone-*d_6_*): δ = 7.98 (d, ^3^J = 8.0 Hz, 1H, Ar-H6), 7.20 (dq, ^3^J = 8.2 Hz, ^4^J = 0.9 Hz, 1H, Ar-H5), 7.00 (d, ^4^J = 0.4 Hz, 1H, Ar-H3), 5.00 (dddd, ^2^J_H-Pt_ = 62 Hz, ^3^J = 13.0 Hz, ^3^J = 7.8 Hz, ^3^J = 5.4 Hz, ^3^J = 5.4 Hz, 1H, -CH=), 4.88 (dd, ^3^J_H-Pt_ = 34 Hz, ^2^J = 12.0 Hz, ^3^J = 5.5 Hz, 1H, -OC**H**_α_H_β_-), 4.47 (dd, ^3^J_H-Pt_ = 31 Hz, ^2^J = 12.0 Hz, ^3^J = 7.7 Hz, 1H, -OCH_α_**H**_β_-), 4.34 (dd, ^2^J_H-Pt_ = 75 Hz, ^3^J = 12.8 Hz, ^2^J = 1.2 Hz, 1H, =CH_2, *trans*_), 4.30 (dd, ^2^J_H-Pt_ = 59 Hz, ^3^J = 7.9 Hz, ^2^J = 0.8 Hz, 1H, =CH_2, *cis*_), 2.40 (s_br_, 3H, -CH_3_), 2.31 (s, 3H, -OAc). ^13^C NMR (101 MHz, acetone-*d_6_*): δ = 169.86 (-(**C**=O)-CH_3_), 164.89 (Ar-(**C**=O)), 151.71 (C2), 145.96 (C4), 132.54 (C6), 127.47 (C5), 125.20 (C3), 121.63 (C1), 77.81 (C2′), 65.16 (C1′), 65.14 (C3′), 21.28 (-CH_3_), 21.25 (-OAc). HR-ESI-MS (*m*/*z*): 534.9586 ([M-K]^−^, calcd for C_13_H_14_Cl_3_O_4_Pt: 534.9575). CE-purity: 97.1%.

Potassium trichlorido[η^2^-(prop-2-en-1-yl)-2-acetoxy-5-methylbenzoate]platinate(II) (5-CH_3_-ASA-Prop-PtCl_3_). Bright yellow needles; yield: 87%. ^1^H NMR (400 MHz, acetone-*d_6_*): δ = 7.90 (d, ^4^J = 2.3 Hz, 1H, Ar-H6), 7.44 (dd, ^3^J = 8.3 Hz, ^4^J = 2.3 Hz, 1H, Ar-H4), 7.05 (d, ^3^J = 8.2 Hz, 1H, Ar-H3), 5.00 (dddd, ^2^J_H-Pt_ = 59 Hz, ^3^J = 13.0 Hz, ^3^J = 7.8 Hz, ^3^J = 5.4 Hz, ^3^J = 5.4 Hz, 1H, -CH=), 4.89 (dd, ^3^J_H-Pt_ = 35 Hz, ^2^J = 12.0 Hz, ^3^J = 5.4 Hz, 1H, -OC**H**_α_H_β_-), 4.49 (dd, ^3^J_H-Pt_ = 44 Hz, ^2^J = 12.0 Hz, ^3^J = 7.8 Hz, 1H, -OCH_α_**H**_β_-), 4.34 (dd, ^2^J_H-Pt_ = 73 Hz, ^3^J = 12.7 Hz, ^2^J = 1.3 Hz, 1H, =CH_2, *trans*_), 4.30 (dd, ^2^J_H-Pt_ = 59 Hz, ^3^J = 7.8 Hz, ^2^J = 1.3 Hz, 1H, =CH_2, *cis*_), 2.39 (s_br_, 3H, -CH_3_), 2.30 (s, 3H, -OAc). ^13^C NMR (101 MHz, acetone-*d_6_*): δ = 169.95 (-(**C**=O)-CH_3_), 165.11 (Ar-(**C**=O)), 149.41 (C2), 136.57 (C5), 135.11 (C4), 132.77 (C6), 124.50 (C3), 124.23 (C1), 77.64 (C2′), 65.30 (C1′), 65.17 (C3′), 21.24 (-OAc), 20.64 (-CH_3_). HR-ESI-MS (*m*/*z*): 534.9587 ([M-K]^−^, calcd for C_13_H_14_Cl_3_O_4_Pt: 534.9578). CE-purity: 97.1%.

Potassium trichlorido[η^2^-(prop-2-en-1-yl)-2-acetoxy-6-methylbenzoate]platinate(II) (6-CH_3_-ASA-Prop-PtCl_3_). Bright yellow needles; yield: 73%. ^1^H NMR (400 MHz, acetone-*d_6_*): δ = 7.38 (dd, ^3^J = 7.9 Hz, ^3^J = 7.9 Hz, 1H, Ar-H4), 7.23-7.17 (m, 1H, Ar-H5), 7.03 (d, ^3^J = 8.1 Hz, 1H, Ar-H3), 5.12-4.85 (m, ^2^J_H-Pt_ = 62 Hz, ^3^J_H-Pt_ = 34 Hz, 2H, -CH= and -OC**H**_α_H_β_-), 4.51 (dd, ^3^J_H-Pt_ = 45 Hz, ^2^J = 11.5 Hz, ^3^J = 7.2 Hz, 1H, -OCH_α_**H**_β_-), 4.34 (dd, ^2^J_H-Pt_ = 74 Hz, ^3^J = 11.8 Hz, ^2^J = 1.4 Hz, 1H, =CH_2, *trans*_), 4.30 (dd, ^2^J_H-Pt_ = 62 Hz, ^3^J = 7.6 Hz, ^2^J = 1.6 Hz, 1H, =CH_2, *cis*_), 2.44 (s_br_, 3H, -CH_3_), 2.26 (s, 3H, -OAc). ^13^C NMR (101 MHz, acetone-*d_6_*): δ = 169.37 (-(**C**=O)-CH_3_), 166.72 (Ar-(**C**=O)), 149.35 (C2), 138.65 (C6), 131.25 (C4), 128.56 (C5), 127.83 (C1), 121.45 (C3), 77.43 (C2′), 65.45 (C1′), 65.03 (C3′), 21.06 (-OAc), 20.24 (-CH_3_). HR-ESI-MS (*m/z*): 534.9587 ([M-K]^−^, calcd for C_13_H_14_Cl_3_O_4_Pt: 534.9578). CE-purity: 98.2%.

Potassium trichlorido[η^2^-(but-3-en-1-yl)-2-acetoxy-3-methylbenzoate]platinate(II) (3-CH_3_-ASA-But-PtCl_3_). Yellow powder; yield: 82%. ^1^H NMR (400 MHz, acetone-*d_6_*): δ = 7.98–7.80 (m, 1H, Ar-H6), 7.51 (dq, ^3^J = 7.6 Hz, ^4^J = 0.8 Hz, 1H, Ar-H4), 7.27 (dd, ^3^J = 7.7 Hz, ^3^J = 7.7 Hz, 1H, Ar-H5), 5.15–4.89 (m, ^2^J_H-Pt_ = 62 Hz, 1H, -CH=), 4.71–4.56 (m, 2H, -OCH_2_-), 4.35–4.12 (m, ^2^J_H-Pt_ = 62 Hz, 2H, =CH_2_), 2.57 (ddt, ^2^J = 14.1 Hz, ^3^J = 8.0 Hz, ^3^J = 6.0 Hz, 1H, -C**H**_α_H_β_-), 2.33 (s, 3H, -OAc), 2.21 (s, 3H, -CH_3_), 2.10–2.00 (m, 1H, -CH_α_**H**_β_-). ^13^C NMR (101 MHz, acetone-*d_6_*): δ = 169.32 (-(**C**=O)-CH_3_), 165.31 (Ar-(**C**=O)), 150.20 (C2), 135.94 (C4), 133.08 (C3), 130.10 (C6), 126.44 (C5), 124.67 (C1), 84.17 (C3′), 65.94 (C4′), 64.80 (C1′), 33.38 (C2′), 20.93 (-OAc), 16.13 (-CH_3_). HR-ESI-MS (*m*/*z*): 547.9763 ([M-K]^−^, calcd for C_14_H_16_Cl_3_O_4_Pt: 547.9756). CE-purity: 99.3%.

Potassium trichlorido[η^2^-(but-3-en-1-yl)-2-acetoxy-4-methylbenzoate]platinate(II) (4-CH_3_-ASA-But-PtCl_3_). Yellow powder; yield: 28%. ^1^H NMR (400 MHz, acetone-*d_6_*): δ = 7.97 (d, ^3^J = 8.0 Hz, 1H, Ar-H6), 7.19 (dq, ^3^J = 8.0 Hz, ^4^J = 0.8 Hz, 1H, Ar-H5), 6.99 (d, ^4^J = 0.8 Hz, 1H, Ar-H3), 5.01 (dddd, ^2^J_H-Pt_ = 64 Hz, ^3^J = 13.7 Hz, ^3^J = 7.7 Hz, ^3^J = 6.3 Hz, ^3^J = 6.3 Hz, 1H, -CH=), 4.68–4.55 (m, 2H, -OCH_2_-), 4.35–4.11 (m, ^2^J_H-Pt_ = 62 Hz, 2H, =CH_2_), 2.56 (ddt, ^2^J = 12.2 Hz, ^3^J = 8.0 Hz, ^3^J = 6.1 Hz, 1H, -C**H**_α_H_β_-), 2.39 (s, 3H, -CH_3_), 2.29 (s, 3H, -OAc), 2.08–1.99 (m, 1H, -CH_α_**H**_β_-). ^13^C NMR (101 MHz, acetone-*d_6_*): δ = 169.77 (-(**C**=O)-CH_3_), 165.02 (Ar-(**C**=O)), 151.77 (C2), 145.86 (C4), 132.41 (C6), 127.49 (C5), 125.21 (C3), 121.73 (C1), 84.26 (C3′), 65.94 (C4′), 64.63 (C1′), 33.41 (C2′), 22.24 (-CH_3_), 21.16 (-OAc). HR-ESI-MS (*m*/*z*): 547.9778 ([M-K]^−^, calcd for C_14_H_16_Cl_3_O_4_Pt: 547.9756). CE-purity: 98.5%.

Potassium trichlorido[η^2^-(but-3-en-1-yl)-2-acetoxy-5-methylbenzoate]platinate(II) (5-CH_3_-ASA-But-PtCl_3_). Yellow powder; yield: 97%. ^1^H NMR (400 MHz, acetone-*d_6_*): δ = 7.88 (d, ^4^J = 2.3 Hz, 1H, Ar-H6), 7.43 (dd, ^3^J = 8.1 Hz, ^4^J = 2.2 Hz, 1H, Ar-H4), 7.05 (d, ^3^J = 8.2 Hz, 1H, Ar-H3), 5.17–4.86 (m, ^2^J_H-Pt_ = 61 Hz, 1H, -CH=), 4.72–4.53 (m, 2H, -OCH_2_-), 4.40–4.10 (m, ^2^J_H-Pt_ = 60 Hz, 2H, =CH_2_), 2.56 (ddt, ^2^J = 14.2 Hz, ^3^J = 8.0 Hz, ^3^J = 6.0 Hz, 1H, -C**H**_α_H_β_-), 2.38 (s, 3H, -CH_3_), 2.28 (s, 3H, -OAc), 2.09–1.98 (m, 1H, -CH_α_**H**_β_-). ^13^C NMR (101 MHz, acetone-d_6_): δ = 169.87 (-(**C**=O)-CH_3_), 165.22 (Ar-(**C**=O)), 149.46 (C2), 136.59 (C5), 135.05 (C4), 132.63 (C6), 124.51 (C3), 124.31 (C1), 84.20 (C3′), 65.94 (C4′), 64.76 (C1′), 33.34 (C2′), 21.13 (-OAc), 20.66 (-CH_3_). HR-ESI-MS (*m*/*z*): 547.9766 ([M-K]^−^, calcd for C_14_H_16_Cl_3_O_4_Pt: 547.9756). CE-purity: 99.2%.

Potassium trichlorido[η^2^-(but-3-en-1-yl)-2-acetoxy-6-methylbenzoate]platinate(II) (6-CH_3_-ASA-But-PtCl_3_). Yellow powder; yield: 82%. ^1^H NMR (400 MHz, acetone-*d_6_*): δ = 7.37 (dd, ^3^J = 7.9 Hz, ^3^J = 7.9 Hz, 1H, Ar-H4), 7.17 (d, ^3^J = 7.6 Hz, 1H, Ar-H5), 7.02 (d, ^3^J = 8.1 Hz, 1H, Ar-H3), 4.98 (dddd, ^2^J_H-Pt_ = 68 Hz, ^3^J = 12.2 Hz, ^3^J = 8.1 Hz, ^3^J = 6.3 Hz, ^3^J = 6.3 Hz, 1H, -CH=), 4.68 (dd, ^3^J = 7.4 Hz, ^3^J = 6.2 Hz, 2H, -OCH_2_-), 4.38–4.08 (m, ^2^J_H-Pt_ = 60 Hz, 2H, =CH_2_), 2.55 (ddt, ^2^J = 14.0 Hz, ^3^J = 8.0 Hz, ^3^J = 6.0 Hz, 1H, -C**H**_α_H_β_-), 2.39 (s, 3H, -CH_3_), 2.26 (s, 3H, -OAc), 2.07–1.97 (m, 1H, -CH_α_**H**_β_-). ^13^C NMR (101 MHz, acetone-d_6_): δ = 169.34 (-(**C**=O)-CH_3_), 166.99 (Ar-(**C**=O)), 149.34 (C2), 138.42 (C6), 131.16 (C4), 128.52 (C5), 128.08 (C1), 121.49 (C3), 83.94 (C3′), 65.98 (C4′), 65.11 (C1′), 33.41 (C2′), 21.05 (-OAc), 20.03 (-CH_3_). HR-ESI-MS (*m*/*z*): 547.9766 ([M-K]^−^, calcd for C_14_H_16_Cl_3_O_4_Pt: 547.9756). CE-purity: 100.0%.

### 2.3. Capillary Electrophoresis

The analysis was performed as previously described [12]. A 50 mM sodium tetraborate solution (pH = 9.3, adjusted with 1 M NaOH), served as background electrolyte (BGE). Washing of novel capillaries consecutively with 1 M NaOH (45 min), water (45 min), and BGE (45 min) was performed. The hydrodynamic injection mode was utilized to introduce the samples into the capillary (50 mbar for 2 s). The capillary was kept at 25 °C, and the autosampler was thermostatted at 37 °C with an external water bath. For separation, the voltage was set to 20 kV, and the detection was carried out at the wavelength of 230 nm. Before each measurement, flushing of the capillary with 0.1 M NaOH (3 min), water (3 min), and BGE (5 min) took place. The samples were dissolved in methanol (MeOH) and further diluted in equal parts by volume with water to give a final concentration of 1 mM. Triple determinations were performed, and the half-lives (τ_½_) are presented as mean ± SD of ≥3 independent experiments. Samples, washing solutions, and buffers were filtered through a membrane with 0.22 µm porosity and degassed by ultrasonication before use. To simplify the reaction dynamics from a possible second-order to pseudo-first-order, one of two reactants must be present in great excess (>10 to 25-fold). If this condition is fulfilled, logarithmic plotting of the gradual decrease in the other reactant’s concentration (or increase in the concentration of a product, respectively) against the time yields a straight line, and half-lives can be calculated.

### 2.4. X-ray Crystallography

The X-ray analyses were conducted as run for similar platinum complexes before [12]. For single crystal structure analysis, an appropriate crystal has been produced under a polarization microscope and directly placed into a stream of cold N_2_ (173 K) inside a Bruker D8 Quest diffractometer (Photon 100). The instrument was supplied with an Incoatec Microfocus source generator (multi-layered optics monochromatized Mo-K_α_ radiation, λ = 71.073 pm). The program SADABS-2014/5 was adduced for multi-scan absorption corrections. After structure solution and parameter refinement with anisotropic displacement parameters for all atoms using the SHELXS/L13 software suite [25,26], the space group P2_1_/c was considered to be correct in both cases. Hydrogen atoms at C1 and C2 were identified and refined with isotropic displacement parameters without any restraints. Further details of determining the crystal structure can be acquired from the Cambridge Crystallographic Data Centre (CCDC). The supplementary crystallographic data of 4-F-ASA-Prop-PtCl_3_ and 5-CH_3_-ASA-Prop-PtCl_3_ were deposed as CCDC numbers 2112877 and 2112876, respectively, and are available for free.

### 2.5. General Cell Culture Methods

The hormone-sensitive breast cancer cell line MCF-7 and the colon cancer cell line HT-29 were purchased from the cell line service (CLS, Eppelheim, Germany). Both cell lines were grown as monolayer in Dulbecco’s Modified Eagle Medium (DMEM), without phenol red, with glucose (4.5 g L^−1^) (GE-Healthcare, Solingen, Germany), supplemented with fetal calf serum (10%; Biochrom, Berlin, Germany), and sodium pyruvate (1%, GE-Healthcare, Solingen, Germany) in a humidified atmosphere (5% CO_2_/95% air) at 37 °C. The cells were passaged twice weekly, routinely checked for mycoplasma infection, and were authenticated by typing short tandem repeats. Stock solutions (200 mM) of the compounds were prepared by dissolving in DMF and further diluted with medium to achieve the respective final concentrations.

### 2.6. Inhibition of COX-1/2 Isoenzyme

The extent of the inhibition of the isolated ovine/human recombinant COX-1/2 isoenzymes by the complexes (1 µM and 10 µM, prepared from a methanolic stock solution (10 mM)) was determined by an enzyme immunoassay (EIA) (COX Inhibitor Screening Assay, Cayman Chemicals, Ann Arbor, MI, USA) following the manufacturer’s protocol. The incubation of the respective complexes with the isoenzymes was exactly 10 min. The values of COX inhibition were calculated as the mean of duplicates and shown as the mean ± SD of ≥2 independent experiments. The solvent-treated control was set to 0% inhibition of the isoenzymes.

### 2.7. Inhibition of the PGE_2_ Synthesis in HT-29 Cells

Exponentially growing HT-29 cells were seeded into 24-well plates at a concentration of 5 × 10^4^ cells in 500 µL of cell culture medium per well. After incubation for 24 h at 37 °C, the platinum complexes were added to the cells at a concentration of 10 µM, and it was incubated for further 24 h. In the next step, the substrate arachidonic acid (Sigma Aldrich, St. Louis, MO, USA) at a concentration of 50 µM was added. After 1 h, the COX-catalyzed PGE_2_ production was stopped by removing the supernatant from the cells, which was analyzed by EIA (Prostaglandin E_2_ Kit—Monoclonal, Cayman Chemicals, Ann Arbor, MI, USA) according to the manufacturer’s protocol. The solvent-treated control was set to 0% inhibition of PGE_2_ induction. The inhibition of PGE_2_ synthesis was calculated as the mean of duplicates and shown as the mean ± SD of ≥ 2 independent experiments.

### 2.8. Antiproliferative Effects

In order to investigate the cytotoxic effects in MCF-7 and HT-29 cells, an already published crystal violet assay was used [27]. The cells (HT-29: 3 × 10^3^ cells per well in 100 µL of medium; MCF-7: 2 × 10^3^ cells per well in 100 µL of medium) were seeded in their exponential growth phase in completed DMEM into 96-well microtiter plates in quadruples. After keeping the cells for 24 h at 37 °C, completed medium containing the vehicle DMF or the newly synthesized complexes, respectively, was added. After incubation for further 72 h, the medium was removed, and cells were washed with phosphate-buffered saline (PBS). Cells were then fixed with a solution of 1% (*v*/*v*) glutaric dialdehyde in PBS. In order to determine the cell biomass, the chromatin of the attached cells was stained with crystal violet, and the bound dye was extracted with EtOH (70%). The absorbance was measured at 590 nm. Mean values ± SD of ≥2 independent experiments were calculated, and the activity of the complexes was expressed as the cell mass of the vehicle-treated control, which was set to 100%. To calculate the IC_50_ values of the complexes, Prism 7.0 (Graph Pad, San Diego, CA, USA) was applied, using non-linear regression and the decadal logarithm of the inhibitor versus variable slope response equation, whereas the top constraint was set to 100%.

## 3. Results and Discussion

### 3.1. Synthesis and Structural Characterization

The synthetic procedures for obtaining the target compounds are summarized in Scheme 1.

Substituted salicylic acids (SAs) were used as educts. Nucleophilic aromatic substitution at the 2,6-difluorobenzoic acid or 2-chloro-6-fluorobenzoic acid with sodium hydroxide in dimethyl sulfoxide (DMSO) yielded 6-F-SA and 6-Cl-SA, respectively. Saponification of ethyl 2-hydroxy-6-methylbenzoate gave 6-CH_3_-SA. All other SA derivatives were commercially available.

The phenolic group of the substituted SAs was then acetylated with acetic anhydride (Ac_2_O) and triethyl amine (TEA) as an auxiliary base in dry tetrahydrofuran (THF), followed by Steglich esterification in dry dichloromethane (CH_2_Cl_2_) with prop-2-en-1-ol or but-3-en-1-ol utilizing *N,N’*-dicyclohexylcarbodiimide (DCC) and 4-dimethylaminopyridine (DMAP) for activation.

The resulting olefinic ligands were reacted with a slight excess of Zeise’s salt in dry EtOH for 3.5 h under protection from light to cause the exchange of the alkene ligands.

It is worth mentioning that the final metal-organic compounds are hygroscopic and photosensitive. They are easily soluble in polar protic solvents but insoluble in CH_2_Cl_2_, diethyl ether, and hydrocarbons. A rapid decomposition to the Pt-DMSO adduct occurs in DMSO, so this solvent must be excluded for the preparation of solutions for NMR studies or stock solutions for in vitro studies.

All the newly produced compounds were characterized by ^1^H and ^13^C{1H} NMR spectroscopy. To correctly assign signals, 2D NMR experiments ([^1^H,^1^H]-COSY, [^1^H,^13^C]-HMBC, and [^1^H,^13^C]-HSQC) were carried out.

Coordination to platinum(II) shifts the vinyl protons of the ligand in the ^1^H NMR spectra significantly to a higher field. Furthermore, the asymmetric character of the ethene moiety results in three =C-H resonances (e.g., 4-F-ASA-Prop-PtCl_3_: δ = 4.99 (-CH=) and 4.33, 4.30 (=CH_2_); Zeise’s salt: δ = 4.20) with geminal and vicinal couplings as well as Pt-H satellites (J_H-Pt_, see experimental part). The methylene protons in ASA-Prop-PtCl_3_ derivatives are diastereotopically split (e.g., 4-F-ASA-Prop-PtCl_3_: δ = 4.88 (dd, ^2^J = 11.9 Hz, ^3^J = 5.7 Hz, 1H) and 4.48 (dd, ^2^J = 12.0 Hz, ^3^J = 7.5 Hz, 1H)).

**Figure 2:**
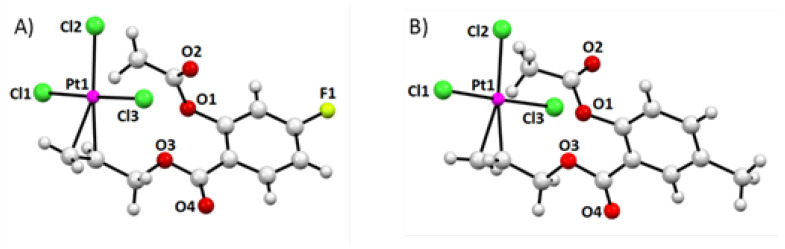
Oak Ridge Thermal-Ellipsoid Plot (ORTEP) of (**A**) 4-F-ASA-Prop-PtCl_3_. Selected bond lengths [Å] and angles [°] are as follows: Pt(1)-Cl(3) 2.2891(18), Pt(1)-Cl(2) 2.3029(19), Pt(1)-Cl(1) 2.3209(17), (C(1) = C(2))-Pt(1) 2.022, C(1)-C(2) 1.388(19), (C1)-C(2)-C(3) 121.7(12). (**B**) 5-CH_3_-ASA-Prop-PtCl_3_. Selected bond lengths [Å] and angles [°] are as follows: Pt(1)-Cl(3) 2.3056(12), Pt(1)-Cl(2) 2.3112(16), Pt(1)-Cl(1) 2.320(3), (C(1) = C(2))-Pt(1) 2.019, C(1)-C(2) 1.420(7), (C1)-C(2)-C(3) 119.7(5). The numbers in the round brackets indicate the “error of the last digit”.

The X-ray crystallography of 4-F-ASA-Prop-PtCl_3_ and 5-CH_3_-ASA-Prop-PtCl_3_ (Figure 2 and Table 1) provides a more detailed insight into the three-dimensional structure.

The crystals of 4-F-ASA-Prop-PtCl_3_ were grown via the vapor diffusion technique from concentrated EtOH solution by the addition of diisopropyl ether. 5-CH_3_-ASA-Prop-PtCl_3_ crystallized from EtOH and diethyl ether. Both complexes possess a comparable structure (Figure 2).

The compounds were additionally characterized by high resolution electrospray ionization mass spectrometry. The free ligands were analyzed in the positive mode via aggregation with ubiquitous sodium and potassium ions. In contrast, the platinum compounds are negatively charged species with characteristic signals in the negative voltage mode (e.g., 4-F-ASA-Prop-PtCl_3_: *m*/*z* 537.9390 [M-K]^−^, calcd for C_12_H_11_Cl_3_FO_4_Pt: 537.9349).

The olefinic ligands are π-bound to the PtCl_3_ moiety in a perpendicular fashion, similar to Zeise’s salt and our lead compound ASA-Prop-PtCl_3_, whose crystal structure was previously published [12].

The ligands at the platinum(II) center adapt a distorted trigonal bispyramidal coordination sphere (Figure 2A,B), with a distance of 2.022 Å and 2.019 Å from the center of the C=C bond to the metal center, similar to that of Zeise’s salt (2.022 Å) [28]. The increased C=C bond length of 4-F-ASA-Prop-PtCl_3_ and 5-CH_3_-ASA-Prop-PtCl_3_ (1.388 and 1.420 Å, respectively; Zeise’s salt: 1.375 Å) further documents the π-bonding to platinum(II). Interestingly, the trans effect of the olefin is not pronounced. The length of the Pt-Cl bond trans to the olefin (4-F-ASA-Prop-PtCl_3_: 2.3029 Å; 5-CH_3_-ASA-Prop-PtCl_3_: 2.3112 Å) differs only slightly from the others (4-F-ASA-Prop-PtCl_3_: 2.2891 and 2.3209 Å; 5-CH_3_-ASA-Prop-PtCl_3_: 2.320 and 2.3056 Å) and is consistent with that of the lead ASA-Prop-PtCl_3_ (2.3244 Å). These data document that the substituents at the ASA core do not influence the binding behavior of the ligands at the platinum(II).

### 3.2. Evaluation of the Complex Stability

An already-established method was used to study the stability of the X-ASA-Prop-PtCl_3_ and X-ASA-But-PtCl_3_ complexes [12]. The degradation products were separated by capillary electrophoresis using a fused-silica capillary (64.5 × 75 µm, effective length: 56 cm, capillary temperature: 25 °C) and a 50 mM tetraborate buffer (pH = 9.3) as BGE. For separation, the voltage was set to 20 kV and the compounds were detected at 230 nm. Benzoic acid was used as an internal standard. Exemplary electropherograms of each complex can be found in the Supplementary Material (Figures S1–S24).

The complexes (1 mM) were dissolved in MeOH or MeOH/water (50/50, *v*/*v*) and stored at 37 °C. The decrease in the initial amount of complex was monitored time-dependently by CE, and the half-life (τ_½_) was calculated (Table 2).

All complexes were stable in pure MeOH for at least 48 h. The addition of water forced the formation of breakdown products.

**Scheme 2:**
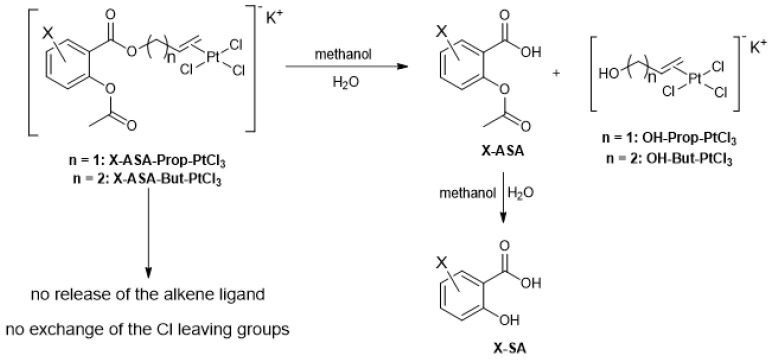
Degradation profile of X-ASA-Prop/But-PtCl_3_ in MeOH/water (50/50, *v*/*v*) solution.

The platinum(II) moiety remained unchanged, but ester cleavage at the X-ASA took place. The degradation profile in MeOH/water (50/50, *v*/*v*) is depicted in Scheme 2.

All of the Zeise’s salt derivatives did not degrade through the release of the alkene from the platinum(II) or the exchange of the chlorido leaving groups. HO-Prop-PtCl_3_ or HO-But-PtCl_3_, as well as X-ASA and X-SA, were detected as degradation products.

The substitution effect was very low in the X-ASA-Prop-PtCl_3_ series with the marginal trend F (τ_½_ = 27.6–32.1 min) < Cl (τ_½_ = 31.4–39.5 min) < CH_3_ (τ_½_ = 35.1–44.8 min). Prop-PtCl_3_ showed a τ_½_ = 35.7 min.

In contrast, X-ASA-But-PtCl_3_ derivatives decomposed with τ_½_ in the range of 35–66 h. The substituents in the ASA group reduced the stability. Only the 5-Cl (τ_½_ = 62.4 h) and the 4-CH_3_ derivatives (τ_½_ = 65.7 h) were as stable as ASA-But-PtCl_3_ (τ_½_ = 69.6 h). The compounds 6-F-ASA-But-PtCl_3_ (τ_½_ = 34.6 h), 4-Cl-ASA-But-PtCl_3_ (τ_½_ = 39.5 h), and 6-CH_3_-ASA-But-PtCl_3_ (τ_½_ = 35.0 h) showed the lowest τ_½_ values. A clear trend is not discernible.

Generally, the degradation of the X-ASA-Prop-PtCl_3_ and X-ASA-But-PtCl_3_ complexes differs from that of Zeise’s salt, which is not stable in an aqueous environment and forms elemental platinum in an internal redox reaction [29].

### 3.3. COX-1/2 Isoenzyme Inhibition

**Figure 3:**
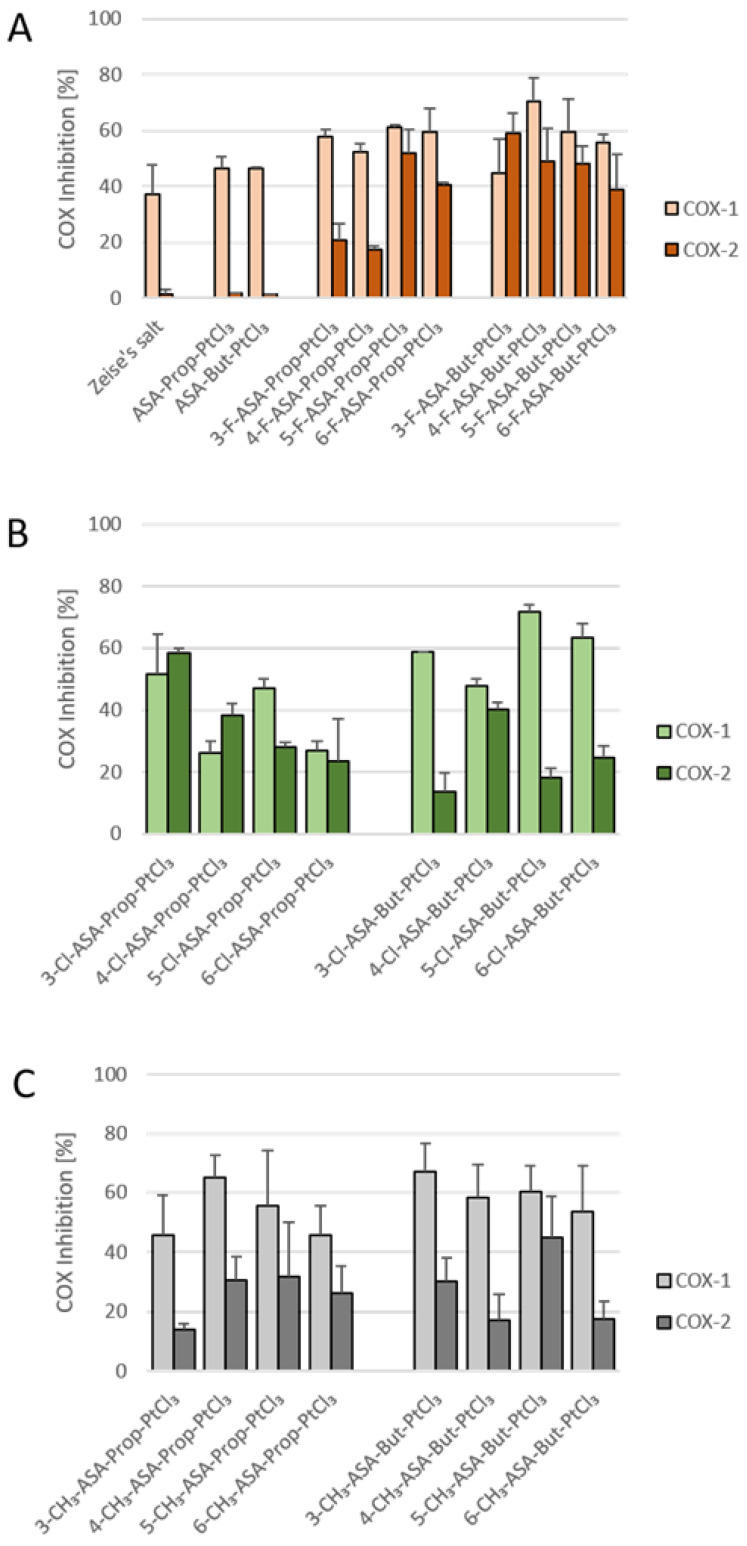
Inhibition of the isolated ovine/human recombinant COX-1 and COX-2 isoenzymes by (**A**) ASA, Zeise’s salt, and the fluorinated derivatives, (**B**) the chlorinated derivatives, and (**C**) the methylated derivatives. Concentration: 1 µM; solvent: MeOH; incubation time: 10 min. The data represent the mean + SD of ≥2 independent experiments.

The potency of the X-ASA-Prop-PtCl_3_ and X-ASA-But-PtCl_3_ complexes to inhibit COX1–1/2 was studied on the isolated isoenzymes at concentrations of 10 µM and 1 µM. The incubation time of 10 min guaranteed that the intact (not decomposed) Zeise’s salt derivatives caused the interaction with the enzyme. The results at 1 µM are depicted in Figure 3, and those at 10 µM in Figure S96 (Supplementary Material).

Zeise’s salt and ASA were used as references (Figure 3 and Figure S96). ASA caused significant effects only at concentrations higher than 100 µM [22,23]. Zeise’s salt represents a selective COX-1 blocker (inhibition at 10 µM: 94.4%; at 1 µM: 37.3%), with no activity at COX-2 (inhibition at 10 µM: 8.3%; at 1 µM: 1.5%). A comparable profile was observed for ASA-Prop-PtCl_3_ and ASA-But-PtCl_3_.

Substituents at the ASA moiety of ASA-Prop-PtCl_3_ and ASA-But-PtCl_3_ enhanced the effects on both isoenzymes. All compounds completely terminated the activity of COX-1 at 10 µM (Figure S96) and reached a maximum inhibition of 70% at COX-2. Escalation to concentrations higher than 10 µM did not further increase the effects on COX-2.

The influence of the substitution pattern is discussed on the results obtained at a concentration of 1 µM (Figure 3).

A fluorine substituent at ASA-Prop-PtCl_3_ and ASA-But-PtCl_3_ only slightly enhanced the effects against COX-1 to 50–60%, independent of its position at the aromatic ring. The influence was more pronounced against COX-2. With the exception of 3-F-ASA-Prop-PtCl_3_ (inhibition: 20.7%) and 4-F-ASA-Prop-PtCl_3_ (inhibition: 17.5%), all fluorinated compounds caused a reduction of enzyme activity between 40% and 60% (Figure 3A), which was very similar to the effects on COX-1.

3-Cl-ASA-Prop-PtCl_3_ effectively inhibited both isoenzymes (inhibition: 51.7% and 58.8%), while the 4-, 5-, and 6-Cl isomers were less effective (Figure 3B). 4-Cl-ASA-Prop-PtCl_3_ had 1.5-fold COX-2 selectivity, whereas 5-Cl-ASA-Prop-PtCl_3_ was 1.7-fold more selective for COX-1. Chlorine substituents at ASA-But-PtCl_3_ increased the inhibition of COX-1 to 50–70%. At COX-2, 4-Cl-ASA-But-PtCl_3_ caused nearly the same effects (inhibition: 40.0%) as for COX-1 (inhibition: 47.9%). The other complexes were also active against COX-2 but with a 2.4- to 4-fold COX-1 selectivity.

A methyl group enhanced the effects of ASA-Prop-PtCl_3_ and ASA-But-PtCl_3_ on COX-1 to 45–65% (Figure 3C). The activity on COX-2 was much less pronounced, so the CH_3_-ASA-Prop-PtCl_3_ complexes exhibited 1.5- to 3-fold COX-1 selectivity.

The highest effect in the CH_3_-ASA-But-PtCl_3_ series was achieved with substitution at position 5. The resulting complex 5-CH_3_-ASA-But-PtCl_3_ decreased the activity of COX-1 by 60.4% and COX-2 by 45.0%. The other complexes of the CH_3_-ASA-But series were about 3-fold more active against COX-1.

### 3.4. Inhibition of the PGE_2_ Synthesis in HT-29 Cells

**Figure 4:**
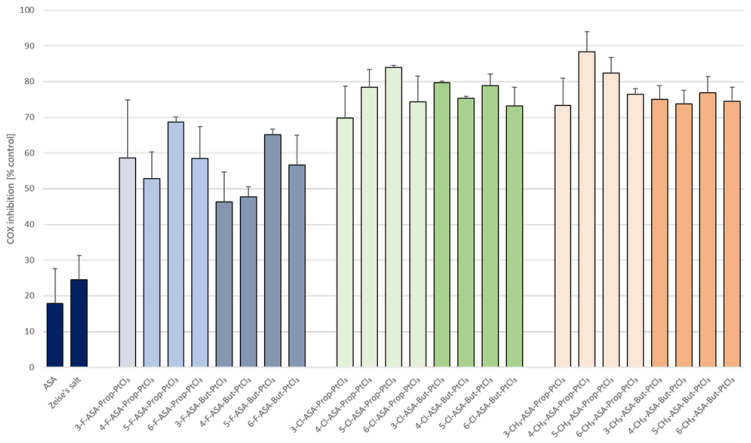
Inhibition of the intracellular PGE_2_ synthesis in the HT-29 cell line by ASA, Zeise’s salt, and its derivatives at 10 µM after incubating for 24 h at 37 °C. The data represent the mean + SD of ≥2 independent experiments.

The stability of Zeise’s salt derivatives permitted the determination of biological effects in cellular systems. HT-29 cells express both isoenzymes at high levels [22], allowing COX-1/2 interaction to be studied using an antibody-based PGE_2_ assay, which quantifies the PGE_2_ released by the cells into the cell culture medium. All Zeise’s salt derivatives were tested at a concentration of 10 µM (Figure 4).

The references ASA and Zeise’s salt reduced the cellular PGE_2_ synthesis only to 17.9% and 24.5%, respectively. While it is well known that cellular response to ASA requires distinctly higher concentrations, it is very likely that Zeise’s salt degrades prior to cellular uptake.

In contrast, Zeise’s salt derivatives are highly active. The F-substituted complexes were less active (inhibition: 46–69%) than their Cl- and CH_3_-bearing congeners (inhibition: 70–84% and 73–88%, respectively). The effects were nearly independent of the position of the substituent and the length of the linker connecting the ASA and Zeise’s salt moiety. Inhibition of the PGE_2_ synthesis higher than 80% was observed for 5-Cl-ASA-Prop-PtCl_3_ (84.0%), 4-CH_3_-ASA-Prop-PtCl_3_ (88.4%), and 5-CH_3_-ASA-Prop-PtCl_3_ (82.4%).

### 3.5. Antiproliferative Effects

The compounds were investigated regarding their cytotoxicity (in vitro) employing an already published crystal violet assay [27]. It is based on the quantification of the cell biomass of living cells by staining the chromatin. In order to evaluate the possible influence of COX inhibition on cell growth, Zeise’s salt derivatives were investigated comparatively in HT-29 and MCF-7 cell lines [22]. The IC_50_ values calculated after an incubation period of 72 h are listed in Table 3. Cisplatin, which was applied as a positive control, caused similar cytotoxicity in both cell lines (HT-29: IC_50_ = 2.6 ± 0.1 µM; MCF-7: IC_50_ = 3.7 ± 0.3 µM).

All fluorinated derivatives showed extremely low cytotoxicity, as evidenced by IC_50_ values of 70–110 µM at the HT-29 and 85–150 µM at the MCF-7 cell line.

More diverged effects resulted from the chlorination of the ASA moiety. With the exception of 3-Cl-ASA-Prop-PtCl_3_ (IC_50_ = 89.7 µM) and 4-Cl-ASA-Prop-PtCl_3_ (IC_50_ = 89.8 µM) all complexes showed cytotoxicity in HT-29 cells with IC_50_ = 35−58 µM.

Against MCF-7 cells, the Cl-ASA-But-PtCl_3_ derivatives (IC_50_ = 28−42 µM) were more active than their Cl-ASA-Prop-PtCl_3_ congeners (IC_50_ = 53−86 µM).

The CH_3_-substituted complexes showed a very interesting activity profile. They reduced the growth of COX-positive HT-29 cells to the same extent (IC_50_ = 16−28 µM), independent of the CH_3_ position at the ASA moiety and the spacer length. They were distinctly less active (IC_50_ = 33−76 µM) in COX-negative MCF-7 cells.

In addition, the complexes were also tested against healthy SV80 cells. All compounds were revealed to be inactive with IC_50_ > 100 µM.

## 4. Conclusions

ASA-Prop-PtCl_3_ and ASA-But-PtCl_3_ were already discovered as suitable lead structures to introduce Zeise’s salt derivatives into medicinal chemistry research [12]. The compounds showed cytotoxicity and potent properties to inhibit COX-1. Because the COX-2 isoenzyme is a more relevant target for decreasing the growth of COX-dependent tumor cells, the aim of this study was to enhance the inhibitory effect on COX-2 by introducing a fluorine, chlorine, or methyl substituent in positions 3, 4, 5, or 6 of the ASA-moiety.

The influence of the substituents on the stability of the complexes is low. Of greater importance is the spacer length between ASA and the platinum-bound alkene. The X-ASA-But-PtCl_3_ derivatives (τ_½_ = 35−66 h) showed higher stability than their X-ASA-Prop-PtCl_3_ analogs (τ_½_ = 28−45 min).

The effects on the isolated COX-1 isoenzyme were slightly higher than that of the unsubstituted leads at a test concentration of 1 µM, while the COX-2 inhibition distinctly raised in all cases. In particular, the F-substituted compounds reached the maximum COX-2 inhibition possible for Zeise’s salt derivatives (60–70%).

COX inhibition was also confirmed in a cellular PGE_2_ assay. All complexes strongly reduced the COX-mediated PGE_2_ formation.

The antiproliferative activity in COX-positive HT-29 and COX-negative MCF-7 cell lines depended on the substituents introduced. Especially the methylated complexes showed the desired cytotoxicity profile.

The complexes had increased cytotoxic potency in COX-positive HT-29 cells (IC_50_ = 16–28 µM) and were less active in COX-negative MCF-7 cells (IC_50_ = 63–76 µM for ASA-Prop-PtCl_3_ derivatives and IC_50_ = 32–46 µM for ASA-But-PtCl_3_ derivatives).

## Figures and Tables

**Chart 1:**
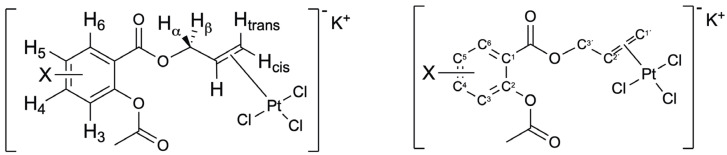
Labeling of selected H and C atoms in ASA-Prop-PtCl_3_ derivatives.

**Table 1:** Selected X-ray data of 4-F-ASA-Prop-PtCl_3_ and 5-CH_3_-ASA-Prop-PtCl_3_.

	4-F-ASA-Prop-PtCl_3_		5-CH_3_-ASA-Prop-PtCl_3_	
Empirical formula	C_12_H_11_Cl_3_FKO_4_Pt		C_13_H_14_Cl_3_KO_4_Pt	
Formula weight	578.75		574.80	
Temperature	173(2) K		173(2) K	
Wavelength	0.71073 Å		0.71073 Å	
Crystal system	Monoclinic		Monoclinic	
Space group	P2_1/c_ (no. 14)		P2_1/c_ (no. 14)	
Unit cell dimensions	a = 15.4348(9) Å	α = 90°	a = 11.1542(5) Å	α = 90°
	b = 9.1776(5) Å	β = 98.677(2)°	b = 12.9486(4) Å	β = 106.491(1)°
	c = 11.7122(6) Å	γ = 90°	c = 16.2368(7) Å	γ = 90°
Volume	4		4	
Z	2.344 mg/m³		1.917 mg/m³	
Density (calculated)	9.319 mm^−1^		6.805 mm^−1^	
Absorption coefficient	1088		1256	
F(000)	0.060 × 0.060 × 0.010 mm³		0.160 × 0.050 × 0.030 mm³	
Crystal size	2.590 to 23.999°		2.046 to 25.250°	
Theta range for data collection	−17 ≤ h ≤ 17, −10 ≤ k ≤ 10, −13 ≤ l ≤ 13		−13 ≤ h ≤ 13, −15 ≤ k ≤ 15, −19 ≤ l ≤ 19	
Index ranges	23154		36453	
Reflections collected	2580 [R(int) = 0.0784]		4075 [R(int) = 0.0354]	
Independent reflections	100.0%		99.7%	
Completeness to theta = 25.242°	Semi-empirical from equivalents		Semi-empirical from equivalents	
Absorption correction	0.888 and 0.747		0.745 and 0.598	
Max. and min. transmission	Full-matrix least-squares on F²		Full-matrix least-squares on F²	
Refinement method	2580/0/194		4075/3/287	
Data/restraints/parameters	1.040		1.292	
Goodness-of-fit on F²	R_1_ = 0.0289, wR_2_ = 0.0527		R_1_ = 0.0244, wR_2_ = 0.0505	
Final R indices [I > 2 sigma(I)]	R_1_ = 0.0479, wR_2_ = 0.0568		R_1_ = 0.0284, wR_2_ = 0.0513	
R indices (all data)	n/a		n/a	
Extinction coefficient	0.706 and −0.758 eÅ^−3^		0.613 and −0.909 eÅ^−3^	
Largest diff. peak and hole	4		4	

**Table 2:** Stability of X-ASA-Prop-PtCl_3_ and X-ASA-But-PtCl_3_ in MeOH/water (50/50, *v*/*v*). Half-lives (τ_½_) were calculated from the decrease in the initial complex determined time-dependently by CE. The data represent the mean ± standard deviation (SD) of ≥3 independent experiments.

Compound	X = F	X = Cl	X = CH_3_
	[τ½/min]	[τ½/min]	[τ½/min]
3-X-ASA-Prop-PtCl_3_	30.5 ± 1.4	31.4 ± 1.7	35.1 ± 2.3
4-X-ASA-Prop-PtCl_3_	32.1 ± 1.1	32.7 ± 1.8	40.4 ± 1.1
5-X-ASA-Prop-PtCl_3_	27.6 ± 1.2	35.2 ± 0.9	36.7 ± 1.1
6-X-ASA-Prop-PtCl_3_	29.1 ± 0.3	39.5 ± 1.9	44.8 ± 1.1
**Compound**	**X = F**	**X = Cl**	**X = CH_3_**
	**[τ½/h]**	**[τ½/h]**	**[τ½/h]**
3-X-ASA-But-PtCl_3_	51.9 ± 2.5	47.0 ± 14.0	45.9 ± 6.0
4-X-ASA-But-PtCl_3_	49.9 ± 4.5	39.5 ± 2.4	65.7 ± 7.2
5-X-ASA-But-PtCl_3_	48.1 ± 4.3	62.4 ± 1.6	52.4 ± 4.3
6-X-ASA-But-PtCl_3_	34.6 ± 1.5	51.8 ± 7.7	35.0 ± 3.7

**Table 3:** Inhibition of the tumor cell growth (HT-29 and MCF-7 cells) was investigated based on the cell mass in a crystal violet assay. The data represent the mean ± SD of ≥ 2 independent experiments.

Compound	X = F		X = Cl		X = CH_3_	
	IC_50_ [µM]		IC_50_ [µM]		IC_50_ [µM]	
	HT-29	MCF-7	HT-29	MCF-7	HT-29	MCF-7
3-X-ASA-Prop-PtCl_3_	86.9 ± 3.9	85.2 ± 21.8	89.7 ± 25.7	56.3 ± 5.8	27.8 ± 3.4	71.4 ± 3.1
4-X-ASA-Prop-PtCl_3_	106.6 ± 3.1	150.1 ± 33.6	89.8 ± 23.9	70.7 ± 3.6	24.2 ± 2.4	62.9 ± 1.6
5-X-ASA-Prop-PtCl_3_	109.7 ± 21.7	106.4 ± 4.1	53.4 ± 0.7	52.8 ± 9.2	21.9 ± 4.6	70.2 ± 2.8
6-X-ASA-Prop-PtCl_3_	113.7 ± 7.1	≥200	38.4 ± 3.4	85.9 ± 9.2	27.4 ± 1.4	76.2 ± 1.4
3-X-ASA-But-PtCl_3_	69.4 ± 1.5	97.4 ± 17.6	43.0 ± 10.9	33.5 ± 1.9	18.0 ± 2.4	45.6 ± 7.2
4-X-ASA-But-PtCl_3_	89.2 ± 3.5	105.9 ± 0.4	35.2 ± 9.3	31.7 ± 8.7	27.1 ± 1.5	38.8 ± 6.5
5-X-ASA-But-PtCl_3_	75.8 ± 13.2	93.8 ± 10.8	49.1 ± 6.2	28.2 ± 4.5	16.2 ± 2.5	32.5 ± 4.4
6-X-ASA-But-PtCl_3_	99.7 ± 0.4	92.6 ± 23.8	57.5 ± 19.2	42.3 ± 8.3	20.2 ± 0.2	35.3 ± 0.9

## Data Availability

The data presented in this study are available in the manuscript and the corresponding Supplementary Materials.

## Supplementary Materials

The following supporting information can be downloaded at: https://www.mdpi.com/article/10.3390/pharmaceutics15061573/s1, Figure S1: Electropherogram of 3-F-Prop-PtCl_3_; Figure S2: Electropherogram of 4-F-Prop-PtCl_3_; Figure S3: Electropherogram of 5-F-Prop-PtCl_3_; Figure S4: Electropherogram of 6-F-Prop-PtCl_3_; Figure S5: Electropherogram of 3-F-But-PtCl_3_; Figure S6: Electropherogram of 4-F-But-PtCl_3_; Figure S7: Electropherogram of 5-F-But-PtCl_3_; Figure S8: Electropherogram of 6-F-But-PtCl_3_; Figure S9: Electropherogram of 3-Cl-Prop-PtCl_3_; Figure S10: Electropherogram of 4-Cl-Prop-PtCl_3_; Figure S11: Electropherogram of 5-Cl-Prop-PtCl_3_; Figure S12: Electropherogram of 6-Cl-Prop-PtCl_3_; Figure S13: Electropherogram of 3-Cl-But-PtCl_3_; Figure S14: Electropherogram of 4-Cl-But-PtCl_3_; Figure S15: Electropherogram of 5-Cl-But-PtCl_3_; Figure S16: Electropherogram of 6-Cl-But-PtCl_3_; Figure S17: Electropherogram of 3-CH_3_-Prop-PtCl_3_; Figure S18: Electropherogram of 4-CH_3_-Prop-PtCl_3_; Figure S19: Electropherogram of 5-CH_3_-Prop-PtCl_3_; Figure S20: Electropherogram of 6-CH_3_-Prop-PtCl_3_; Figure S21: Electropherogram of 3-CH_3_-But-PtCl_3_; Figure S22: Electropherogram of 4-CH_3_-But-PtCl_3_; Figure S23: Electropherogram of 5-CH_3_-But-PtCl_3_; Figure S24: Electropherogram of 6-CH_3_-But-PtCl_3_; Figure S25: ^1^H and ^13^C NMR spectra of 3-F-ASA-Prop; Figure S26: ^1^H and ^13^C NMR spectra of 3-F-ASA-But; Figure S27: ^1^H and ^13^C NMR spectra of 4-F-ASA-Prop; Figure S28: ^1^H and ^13^C NMR spectra of 4-F-ASA-But; Figure S29: ^1^H and ^13^C NMR spectra of 5-F-ASA-Prop; Figure S30: ^1^H and ^13^C NMR spectra of 5-F-ASA-But; Figure S31: ^1^H and ^13^C NMR spectra of 6-F-ASA-Prop; Figure S32: ^1^H and ^13^C NMR spectra of 6-F-ASA-But; Figure S33: ^1^H and ^13^C NMR spectra of 3-Cl-ASA-Prop; Figure S34: ^1^H and ^13^C NMR spectra of 3-Cl-ASA-But; Figure S35: ^1^H and ^13^C NMR spectra of 4-Cl-ASA-Prop; Figure S36: ^1^H and ^13^C NMR spectra of 4-Cl-ASA-But; Figure S37: ^1^H and ^13^C NMR spectra of 5-Cl-ASA-Prop; Figure S38: ^1^H and ^13^C NMR spectra of 5-Cl-ASA-But; Figure S39: ^1^H and ^13^C NMR spectra of 6-Cl-ASA-Prop; Figure S40: ^1^H and ^13^C NMR spectra of 6-Cl-ASA-But; Figure S41: ^1^H and ^13^C NMR spectra of 3-CH_3_-ASA-Prop; Figure S42: ^1^H and ^13^C NMR spectra of 3-CH_3_-ASA-But; Figure S43: ^1^H and ^13^C NMR spectra of 4-CH_3_-ASA-Prop; Figure S44: ^1^H and ^13^C NMR spectra of 4-CH_3_-ASA-But; Figure S45: ^1^H and ^13^C NMR spectra of 5-CH_3_-ASA-Prop; Figure S46: ^1^H and ^13^C NMR spectra of 5-CH_3_-ASA-But; Figure S47: ^1^H and ^13^C NMR spectra of 6-CH_3_-ASA-Prop; Figure S48: ^1^H and ^13^C NMR spectra of 6-CH_3_-ASA-But; Figure S49: ^1^H and ^13^C NMR spectra of 3-F-ASA-Prop-PtCl_3_; Figure S50: ^1^H and ^13^C NMR spectra of 3-F-ASA-But-PtCl_3_; Figure S51: ^1^H and ^13^C NMR spectra of 4-F-ASA-Prop-PtCl_3_; Figure S52: ^1^H and ^13^C NMR spectra of 4-F-ASA-But-PtCl_3_; Figure S53: ^1^H and ^13^C NMR spectra of 5-F-ASA-Prop-PtCl_3_; Figure S54: ^1^H and ^13^C NMR spectra of 5-F-ASA-But-PtCl_3_; Figure S55: ^1^H and ^13^C NMR spectra of 6-F-ASA-Prop-PtCl_3_; Figure S56: ^1^H and ^13^C NMR spectra of 6-F-ASA-But-PtCl_3_; Figure S57: ^1^H and ^13^C NMR spectra of 3-Cl-ASA-Prop-PtCl_3_; Figure S58: ^1^H and ^13^C NMR spectra of 3-Cl-ASA-But-PtCl_3_; Figure S59: ^1^H and ^13^C NMR spectra of 4-Cl-ASA-Prop-PtCl_3_; Figure S60: ^1^H and ^13^C NMR spectra of 4-Cl-ASA-But-PtCl_3_; Figure S61: ^1^H and ^13^C NMR spectra of 5-Cl-ASA-Prop-PtCl_3_; Figure S62: ^1^H and ^13^C NMR spectra of 5-Cl-ASA-But-PtCl_3_; Figure S63: ^1^H and ^13^C NMR spectra of 6-Cl-ASA-Prop-PtCl_3_; Figure S64: ^1^H and ^13^C NMR spectra of 6-Cl-ASA-But-PtCl_3_; Figure S65: ^1^H and ^13^C NMR spectra of 3-CH_3_-ASA-Prop-PtCl_3_; Figure S66: ^1^H and ^13^C NMR spectra of 3-CH_3_-ASA-But-PtCl_3_; Figure S67: ^1^H and ^13^C NMR spectra of 4-CH_3_-ASA-Prop-PtCl_3_; Figure S68: ^1^H and ^13^C NMR spectra of 4-CH_3_-ASA-But-PtCl_3_; Figure S69: ^1^H and ^13^C NMR spectra of 5-CH_3_-ASA-Prop-PtCl_3_; Figure S70: ^1^H and ^13^C NMR spectra of 5-CH_3_-ASA-But-PtCl_3_; Figure S71: ^1^H and ^13^C NMR spectra of 6-CH_3_-ASA-Prop-PtCl_3_; Figure S72: ^1^H and ^13^C NMR spectra of 6-CH_3_-ASA-But-PtCl_3_; Figure S73: HR-ESI-MS spectrum of 3-F-ASA-Prop-PtCl_3_; Figure S74: HR-ESI-MS spectrum of 4-F-ASA-Prop-PtCl_3_; Figure S75: HR-ESI-MS spectrum of 5-F-ASA-Prop-PtCl_3_; Figure S76: HR-ESI-MS spectrum of 6-F-ASA-Prop-PtCl_3_; Figure S77: HR-ESI-MS spectrum of 3-F-ASA-But-PtCl_3_; Figure S78: HR-ESI-MS spectrum of 4-F-ASA-But-PtCl_3_; Figure S79: HR-ESI-MS spectrum of 5-F-ASA-But-PtCl_3_; Figure S80: HR-ESI-MS spectrum of 6-F-ASA-But-PtCl_3_; Figure S81: HR-ESI-MS spectrum of 3-Cl-ASA-Prop-PtCl_3_; Figure S82: HR-ESI-MS spectrum of 4-Cl-ASA-Prop-PtCl_3_; Figure S83: HR-ESI-MS spectrum of 5-Cl-ASA-Prop-PtCl_3_; Figure S84: HR-ESI-MS spectrum of 6-Cl-ASA-Prop-PtCl_3_; Figure S84: HR-ESI-MS spectrum of 3-Cl-ASA-But-PtCl_3_; Figure S85: HR-ESI-MS spectrum of 4-Cl-ASA-But-PtCl_3_; Figure S86: HR-ESI-MS spectrum of 5-Cl-ASA-But-PtCl_3_; Figure S87: HR-ESI-MS spectrum of 6-Cl-ASA-But-PtCl_3_; Figure S88: HR-ESI-MS spectrum of 3-CH_3_-ASA-Prop-PtCl_3_; Figure S89 HR-ESI-MS spectrum of 4-CH_3_-ASA-Prop-PtCl_3_; Figure S90: HR-ESI-MS spectrum of 5-CH_3_-ASA-Prop-PtCl_3_; Figure S91: HR-ESI-MS spectrum of 6-CH_3_-ASA-Prop-PtCl_3_; Figure S92: HR-ESI-MS spectrum of 3-CH_3_-ASA-But-PtCl_3_; Figure S93: HR-ESI-MS spectrum of 4-CH_3_-ASA-But-PtCl_3_; Figure S94: HR-ESI-MS spectrum of 5-CH_3_-ASA-But-PtCl_3_; Figure S95: HR-ESI-MS spectrum of 6-CH_3_-ASA-But-PtCl_3_; Figure S96: Inhibition of COX-1/2.

## References

[1] Smith W.L., DeWitt D.L., Garavito R.M. (2000). Cyclooxygenases: Structural, cellular, and molecular biology. Annu. Rev. Biochem..

[2] Phillis J.W., Horrocks L.A., Farooqui A.A. (2006). Cyclooxygenases, lipoxygenases, and epoxygenases in CNS: Their role and involvement in neurological disorders. Brain Res. Rev..

[3] Simmons D.L., Botting R.M., Hla T. (2004). Cyclooxygenase isozymes: The biology of prostaglandin synthesis and inhibition. Pharmacol. Rev..

[4] Regulski M., Regulska K., Prukala W., Piotowska H., Stanisz B., Murias M. (2016). COX-2 inhibitors: A novel strategy in the management of breast cancer. Drug Discov. Today.

[5] Pannunzio A., Coluccia M. (2018). Cyclooxygenase-1 (COX-1) and COX-1 Inhibitors in cancer: A review of oncology and medicinal chemistry literature. Pharmaceuticals.

[6] Jeong H.-S., Kim J.-H., Choi H.Y., Lee E.-R., Cho S.-G. (2010). Induction of cell growth arrest and apoptotic cell death in human breast cancer MCF-7 cells by the COX-1 inhibitor FR122047. Oncol. Rep..

[7] McFadden D.W., Riggs D.R., Jackson B.J., Cunningham C. (2006). Additive effects of COX-1 and COX-2 inhibition on breast cancer in vitro. Int. J. Oncol..

[8] Li W., Wang J., Jiang H.-R., Xu X.-L., Zhang J., Liu M.-L., Zhai L.-Y. (2011). Combined effects of cyclooxygenase-1 and cyclooxygenase-2 selective inhibitors on ovarian carcinoma in vivo. Int. J. Mol. Sci..

[9] Ghosh S. (2019). Cisplatin: The first metal based anticancer drug. Bioorg. Chem..

[10] Dasari S., Tchounwou P.B. (2014). Cisplatin in cancer therapy: Molecular mechanisms of action. Eur. J. Pharmacol..

[11] Fuertes M.A., Alonso C., Pérez J.M. (2003). Biochemical modulation of cisplatin mechanisms of action: Enhancement of antitumor activity and circumvention of drug resistance. Chem. Rev..

[12] Weninger A., Baecker D., Obermoser V., Egger D., Wurst K., Gust R. (2018). Synthesis and biological evaluation of Zeise’s salt derivatives with acetylsalicylic acid substructure. Int. J. Mol. Sci..

[13] Thun M.J., Jacobs E.J., Patrono C. (2012). The role of aspirin in cancer prevention. Nat. Rev. Clin. Oncol..

[14] Nakanishi M., Rosenberg D.W. (2013). Multifaceted roles of PGE2 in inflammation and cancer. Semin. Immunopathol..

[15] Meieranz S., Stefanopoulou M., Rubner G., Bensdorf K., Kubutat D., Sheldrick W.S., Gust R. (2015). The biological activity of Zeise’s salt and its derivatives. Angew. Chem. Int. Ed. Engl..

[16] Ott I., Kircher B., Bagowski C.P., Vlecken D.H.W., Ott E.B., Will J., Bensdorf K., Sheldrick W.S., Gust R. (2009). Modulation of the biological properties of aspirin by formation of a bioorganometallic derivative. Angew. Chem. Int. Ed. Engl..

[17] Rubner G., Bensdorf K., Wellner A., Bergemann S., Gust R. (2011). Synthesis, characterisation and biological evaluation of copper and silver complexes based on acetylsalicylic acid. Arch. Pharm..

[18] Rubner G., Bensdorf K., Wellner A., Kircher B., Bergemann S., Ott I., Gust R. (2010). Synthesis and biological activities of transition metal complexes based on acetylsalicylic acid as neo-anticancer agents. J. Med. Chem..

[19] Ott I., Schmidt K., Kircher B., Schumacher P., Wiglenda T., Gust R. (2005). Antitumor-active cobalt-alkyne complexes derived from acetylsalicylic acid:  Studies on the mode of drug action. J. Med. Chem..

[20] Hinz B., Brune K. (2002). Cyclooxygenase-2—10 years later. J. Pharmacol. Exp. Ther..

[21] Rouzer C.A., Marnett L.J. (2009). Cyclooxygenases: Structural and functional insights. J. Lipid Res..

[22] Obermoser V., Baecker D., Schuster C., Braun V., Kircher B., Gust R. (2018). Chlorinated cobalt alkyne complexes derived from acetylsalicylic acid as new specific antitumor agents. Dalton Trans..

[23] Baecker D., Obermoser V., Kirchner E.A., Hupfauf A., Kircher B., Gust R. (2019). Fluorination as tool to improve bioanalytical sensitivity and COX-2-selective antitumor activity of cobalt alkyne complexes. Dalton Trans..

[24] Baecker D., Sagasser J., Karaman S., Hörmann A.A., Gust R. (2022). Development of methylated cobalt-alkyne complexes with selective cytotoxicity against COX-positive cancer cell lines. Arch. Pharm..

[25] Sheldrick G.M. (2015). Crystal structure refinement with SHELXL. Acta Crystallogr. C Struct. Chem..

[26] Sheldrick G.M. (2013). SHELXL-2013/1, Program Suit for the Solution and Refinement of Crystal Structures.

[27] Obermoser V., Urban M.E., Murgueitio M.S., Wolber G., Kintscher U., Gust R. (2016). New telmisartan-derived PPARγ agonists: Impact of the 3D-binding mode on the pharmacological profile. Eur. J. Med. Chem..

[28] Love R.A., Koetzle T.F., Williams G.J.B., Andrews L.C., Bau R. (1975). Neutron diffraction study of the structure of Zeise’s salt, KPtCl_3_(C_2_H_4_).H_2_O. Inorg. Chem..

[29] Joy J.R., Orchin M. (1960). Hydrolyse des Zeise-Salzes. Z. Anorg. Allg. Chem..

