# Supplementary material for: Development of Zeise’s Salt Derivatives Bearing Substituted Acetylsalicylic Acid Substructures as Cytotoxic COX Inhibitors
Source: Pharmaceutics. 2023 May 23;15(6):1573. doi: 10.3390/pharmaceutics15061573 (PMC10304174; doi:10.3390/pharmaceutics15061573)
Supplement: Supplementary file 1 [file pharmaceutics-15-01573-s001.zip › pharmaceutics-2264722-supplementary.pdf]

Supplementary Materials

Table of contents

Synthesis of intermediates and ligands……………………………………………...… S2
Electropherograms of the complexes……………………………………….……….. S9
NMR spectra of the ligands and complexes…………………………………...….. S21
HR-ESI-MS spectrua of the complexes………………………………………… …. S69
IR data of selected ligands and complexes…………………………………………. S81
Inhibition of COX-1/2………………………………………………………...……….. S82

Synthesis and characterization of intermediates and ligands

General procedure for the synthesis of 2-fluoro- and 2-chloro-6-hydroxybenzoic acid

2,6-Difluorobenzoic acid or 2-chloro-6-fluorobenzoic acid were respectively dissolved in 26 eq. of DMSO. After addition of 4 eq. of finely powdered NaOH, the reaction mixture was vigorously stirred at 130 °C for 12 h. Then, the solvent was removed in vacuo. The crude product was dissolved in 50 mL of 2 M HCl and extracted three times with 20 mL of CH_2Cl_2. The organic layers were combined and dried over anhydrous Na_2SO_4. The solvent was then evaporated to afford the pure crystalline respective substituted salicylic acids.

2-Fluoro-6-hydroxybenzoic acid (6-F-SA)

Colorless crystals; yield: 41%. ^{1}H NMR (400 MHz, acetone-d_6): δ = 7.51 (ddd, ^{3}J = 8.4 Hz, ^{3}J =8.4 Hz, $^4J_{H-F}$ = 6.1 Hz, 1H, Ar-H4), 6.79 (ddd, ^{3}J = 8.5 Hz, ^{4}J = 1.1 Hz, $^5J_{H-F}$ = 1.1 Hz, 1H, Ar-H3), 6.70 (ddd, $^3J_{H-F}$ = 11.0 Hz, ^{3}J = 8.3 Hz, ^{4}J = 1.1 Hz, 1H, Ar-H5). ^{13}C NMR (101 MHz, acetone-d_6): δ = 171.47 (d, $^3J_{C-F}$ = 3.1 Hz, -CO_2H), 164.46 (d, $^3J_{C-F}$ = 3.6 Hz, C2), 163.46 (d, $^1J_{C-F}$ = 258.7 Hz, C6), 136.54 (d, $^3J_{C-F}$ = 12.3 Hz, C4), 113.99 (d, $^4J_{C-F}$ = 3.4 Hz, C3), 107.47 (d, $^2J_{C-F}$ = 23.2 Hz, C5), 103.56 (d, $^2J_{C-F}$ = 13.5 Hz, C1).

2-Chloro-6-hydroxybenzoic acid (6-Cl-SA)

Colorless crystals; yield: Colorless crystals; yield: 61%. ^{1}H NMR (200 MHz, $CDCl_3$): δ = 11.8 (s, 1H, OH), 7.37 (dd, ^{3}J = 8.4 Hz, ^{3}J = 8.4 Hz, 1H, Ar-H4), 7.02 (dd, ^{3}J = 7.8 Hz, ^{4}J = 1.4 Hz, 1H, Ar-H5), 6.97 (d, J^3 = 8.4 Hz, 1H, Ar-H3).

General procedure for the saponification of ethyl 2-hydroxy-6-methylbenzoate

An amount of 1.0 eq. of ethyl 2-hydroxy-6-methylbenzoate was dissolved in 10 mL per eq. of a mixture of THF:MeOH:H_2O = 3:1:1 (*v*/*v*/*v*) upon which 5.0 eq. of finely powdered NaOH were added. The mixture was stirred at 70 °C for 6 h. After completion of the reaction, 60 mL of 1 *N* HCl was added (pH = 2) and a colorless solid precipitated that was separated, washed with three portions of H_2O and then dried in vacuo.

2-Hydroxy-6-methylbenzoic acid (6-M-SA)

Colorless powder; yield: 91%. ^{1}H NMR (400 MHz, acetone-d_6): δ = 11.84 (s_{br}, 2H, -CO_2H and -OH), 7.33 (dd, 1H, Ar-H4), 6.85-6.71 (m, 2H, Ar-H5 and Ar-H3), 2.58 (s, 3H, -CH_3). ^{13}C NMR (101 MHz, acetone-d_6): δ = 174.04 (Ar-(**C**=O)), 164.18 (C2), 142.55 (C6), 135.11 (C4), 123.45 and 116.09 (C3/C5), 113.18 (C1), 23.88 (-CH_3).

General procedure for the acetylation of salicylic acids

A total of 1.0 eq. of the 2-hydroxybenzoic acid derivative in 30 mL (per 20 mmol acid) anhydrous tetrahydrofuran was added dropwise to an ice-cold mixture of 10 eq. of acetic anhydride and 2.0 eq. of triethylamine (2.0 eq.) in 30 mL anhydrous tetrahydrofuran over a period of 30 min. After addition, the ice-bath was removed, and the mixture was stirred for 24 h at room temperature. Then, the mixture was diluted with 50 mL water and was acidified with 1 M HCl. The reaction mixture was extracted with 3x 100 mL diethyl ether. The organic layers were combined and washed once with brine after which the organic phase was dried over anhydrous Na_2SO_4. After evaporation of the solvent, pure solid 2-acetoxybenzoic acid derivatives were obtained by recrystallization from toluene.

2-Acetoxy-3-fluorobenzoic acid (3-F-ASA)

Colorless powder; yield: 71%. ^{1}H NMR (400 MHz, acetone-d_6): δ = 7.85 (ddd, ^{3}J = 7.9 Hz, ^{4}J = 1.5 Hz, $^5J_{H-F}$ = 1.5 Hz, 1H, Ar-H6), 7.54 (ddd, $^3J_{H-F}$ = 9.9 Hz, ^{3}J = 8.3 Hz, ^{4}J = 1.6 Hz, 1H, Ar-H4), 7.42 (ddd, ^{3}J = 8.1 Hz, ^{3}J = 8.1 Hz, $^4J_{H-F}$ = 5.1 Hz, 1H, Ar-H5), 2.31 (s, 3H, -OAc). ^{13}C NMR (101 MHz, acetone-d_6): δ = 168.85 (-(**C**=O)-CH_3), 165.06 (d, $^4J_{C-F}$ = 3.1 Hz, Ar-(**C**=O)), 155.95 (d, $^1J_{C-F}$ = 247.0 Hz, C3), 139.75 (d, $^2J_{C-F}$ = 14.8 Hz, C2), 127.79 (d, $^4J_{C-F}$ = 3.5 Hz, C6), 127.57 (d, $^3J_{C-F}$ = 7.9 Hz, C5), 127.04 (C1), 121.35 (d, $^2J_{C-F}$ = 19.4 Hz, C4), 20.51 (-OAc).

2-Acetoxy-4-fluorobenzoic acid (4-F-ASA)

Colorless needles; yield: 52%. ^{1}H NMR (400 MHz, acetone-d_6): δ = 11.45 (s_{br}, 1H, -CO_2H), 8.13 (dd, ^{3}J = 8.8 Hz, $^4J_{H-F}$ = 6.5 Hz, 1H, Ar-H6), 7.20 (ddd, ^{3}J = 8.7 Hz, $^3J_{H-F}$ = 8.1 Hz, ^{4}J = 2.6 Hz, 1H, Ar-H5), 7.06 (dd,

$^3J_{H\text{-}F}$ = 9.3 Hz, ^4J = 2.6 Hz, 1H, Ar-H3), 2.26 (s, 3H, -OAc). ^{13}C NMR (101 MHz, acetone-d_6): δ = 169.44 (-(**C**=O)-CH_3), 166.16 (d, $^1J_{C\text{-}F}$ = 253.8 Hz, C4), 164.95 (Ar-(**C**=O)), 153.84 (d, $^3J_{C\text{-}F}$ = 12.3 Hz, C2), 134.80 (d, $^3J_{C\text{-}F}$ = 10.3 Hz, C6), 121.28 (d, $^4J_{C\text{-}F}$ = 3.6 Hz, C1), 113.86 (d, $^2J_{C\text{-}F}$ = 21.3 Hz, C5), 112.51 (d, $^2J_{C\text{-}F}$ = 24.1 Hz, C3), 20.95 (-OAc).

2-Acetoxy-5-fluorobenzoic acid (5-F-ASA)

Transparent needles; yield: 88%. ^{1}H NMR (400 MHz, acetone-d_6): δ = 7.73 (dd, $^3J_{H\text{-}F}$ = 9.0 Hz, ^4J = 3.2 Hz, 1H, Ar-H6), 7.44 (ddd, $^3J_{H\text{-}F}$ = 8.9 Hz, ^3J = 7.8 Hz, ^4J = 3.2 Hz, 1H, Ar-H4), 7.25 (dd, ^3J = 8.9 Hz, $^4J_{H\text{-}F}$ = 4.7 Hz, 1H, Ar-H3), 2.22 (s, 3H, -OAc). ^{13}C NMR (101 MHz, acetone-d_6): δ = 169.82 (-(**C**=O)-CH_3), 164.76 (d, $^4J_{C\text{-}F}$ = 1.4 Hz, Ar-(**C**=O)), 160.48 (d, $^1J_{C\text{-}F}$ = 243.4 Hz, C5), 148.09 (d, $^4J_{C\text{-}F}$ = 2.6 Hz, C2), 126.79 (d, $^3J_{C\text{-}F}$ = 8.5 Hz, C3), 126.18 (d, $^3J_{C\text{-}F}$ = 7.4 Hz, C1), 121.38 (d, $^2J_{C\text{-}F}$ = 23.1 Hz, C4), 118.64 (d, $^2J_{C\text{-}F}$ = 25.3 Hz, C6), 20.91 (-OAc).

2-Acetoxy-6-fluorobenzoic acid (6-F-ASA)

Colorless crystals; yield: 65%. ^{1}H NMR (400 MHz, acetone-d_6): δ = 11.25 (s_{br}, 1H, -CO_2H), 7.58 (ddd, ^3J = 8.4 Hz, ^3J = 8.4 Hz, $^4J_{H\text{-}F}$ = 6.2 Hz, 1H, Ar-H4), 7.18 (ddd, $^3J_{H\text{-}F}$ = 9.6 Hz, ^3J = 8.5 Hz, ^4J = 1.0 Hz, 1H, Ar-H5), 7.07 (ddd, ^3J = 8.2 Hz, ^4J = 1.0 Hz, $^5J_{H\text{-}F}$ = 1.0 Hz, 1H, Ar-H3), 2.24 (s, 3H, -OAc). ^{13}C NMR (101 MHz, acetone-d_6): δ = 169.22 (-(**C**=O)-CH_3), 163.54 (Ar-(**C**=O)), 161.36 (d, $^1J_{C\text{-}F}$ = 252.9 Hz, C6), 150.92 (d, $^3J_{C\text{-}F}$ = 5.7 Hz, C2), 133.23 (d, $^3J_{C\text{-}F}$ = 10.2 Hz, C4), 120.36 (d, $^4J_{C\text{-}F}$ = 3.6 Hz, C3), 116.89 (d, $^2J_{C\text{-}F}$ = 17.4 Hz, C1), 114.42 (d, $^2J_{C\text{-}F}$ = 22.4 Hz, C5), 20.73 (-OAc).

2-Acetoxy-3-chlorobenzoic acid (3-Cl-ASA)

Colorless powder; yield: 69%. ^{1}H NMR (200 MHz, $CDCl_3$): 8.02 (dd, ^3J = 8 Hz, ^4J = 1.2 Hz, 1H, Ar-H6), 7.7 (dd, ^3J = 8.2 Hz, 4J = 1.2 Hz, 1H, Ar-H4), 7.33 (d, ^3J = 8 Hz, 1H, Ar-H5), 2.4 (s, 3H, CH_3).

2-Acetoxy-4-chlorobenzoic acid (4-Cl-ASA)

Colorless powder; yield: 78%.^{1}HNMR (200 MHz, $CDCl_3$): 8.06 (d, ^3J = 8.6 Hz, 1H, Ar-H6), 7.34 (dd, ^3J = 8.6 Hz, ^4J = 1.6 Hz, 1H, Ar-H5), 7.17 (d, ^4J = 2 Hz, 1H, Ar-H3), 2.34 (s, 3H, CH_3).

2-Acetoxy-5-chlorobenzoic acid (5-Cl-ASA)

Colorless powder; yield: 62%. ^{1}H NMR (200 MHz, $CDCl_3$): 8.09 (d, ^4J = 2.4 Hz, 1H, Ar-H6), 7.58 (dd, ^3J = 8.4 Hz, ^4J = 2.6 Hz, 1H, Ar-H4), 7.09 (d, ^3J = 8.8 Hz, 1H, Ar-H3), 2.34 (s, 3 H, CH_3).

2-Acetoxy-6-chlorobenzoic acid (6-Cl-ASA)

Colorless powder; yield: 77%. ^{1}H NMR (200 MHz, $CDCl_3$): 7.47–7.34 (m, 2H, Ar-H), 7.12 (dd, ^3J = 7.6 Hz, ^4J = 1.8 Hz, 1H, Ar-H), 2.31 (s, 3 H, CH_3).

2-Acetoxy-3-methylbenzoic acid (3-M-ASA)

Colorless powder; yield: 74%. ^{1}H NMR (400 MHz, acetone-d_6): δ = 11.29 (s_{br}, 1H, -CO_2H), 7.92-7.81 (m, 1H, Ar-H6), 7.57-7.47 (m, 1H, Ar-H4), 7.27 (dd, ^3J = 7.7 Hz, ^3J = 7.7 Hz, 1H, Ar-H5), 2.28 (s, 3H, -OAc), 2.21 (s, 3H, -CH_3). ^{13}C NMR (101 MHz, acetone-d_6): δ = 169.30 (-(**C**=O)-CH_3), 166.04 (Ar-(**C**=O)), 150.54 (C2), 136.02 (C4), 133.11 (C3), 130.25 (C6), 126.32 (C5), 124.47 (C1), 20.84 (-OAc), 16.11 (-CH_3).

2-Acetoxy-4-methylbenzoic acid (4-M-ASA)

Colorless powder; yield: 79%. ^{1}H NMR (400 MHz, acetone-d_6): δ = 11.20 (s_{br}, 1H, -CO_2H), 7.93 (d, ^3J = 8.0 Hz, 1H, Ar-H6), 7.23-7.15 (m, 1H, Ar-H5), 7.04-6,94 (m, 1H, Ar-H3), 2.40 (s, 3H, -CH_3), 2.23 (s, 3H, -OAc). ^{13}C NMR (101 MHz, acetone-d_6): δ = 169.73 (-(**C**=O)-CH_3), 165.73 (Ar-(**C**=O)), 152.13 (C2), 145.97 (C4), 132.58 (C6), 127.39 (C5), 125.21 (C3), 121.57 (C1), 21.24 (-CH_3), 21.04 (-OAc).

2-Acetoxy-5-methylbenzoic acid (5-M-ASA)

Colorless powder; yield: 68%. ^{1}H NMR (400 MHz, acetone-d_6): δ = 11.27 (s_{br}, 1H, -CO_2H), 7.88-7.78 (m, 1H, Ar-H6), 7.44 (ddq, ^3J = 8.2 Hz, ^4J = 2.2 Hz, ^4J = 0.7 Hz, 1H, Ar-H4), 7.05 (d, ^3J = 8.2 Hz, 1H, Ar-H3), 2.38 (s, 3H, -CH_3), 2.23 (s, 3H, -OAc). ^{13}C NMR (101 MHz, acetone-d_6): δ = 169.85 (-(**C**=O)-CH_3), 165.90 (Ar-(**C**=O)), 149.84 (C2), 136.47 (C5), 135.15 (C4), 132.82 (C6), 124.53 (C3), 124.14 (C1), 21.02 (-OAc), 20.64 (-CH_3).

2-Acetoxy-6-methylbenzoic acid (6-M-ASA)

Colorless powder; yield: 77%. ^{1}H NMR (400 MHz, acetone-d_6): δ = 11.54 (s_{br}, 1H, -CO_2H), 7.37 (dd, ^3J = 7.9 Hz, ^3J = 7.9 Hz, 1H, Ar-H4), 7.22-7.13 (m, 1H, Ar-H5), 7.07-6.96 (m, 1H, Ar-H3), 2.42 (s, 3H, -CH_3),

2.21 (s, 3H, -OAc). ^{13}C NMR (101 MHz, acetone-d_6): δ = 169.29 (-(**C**=O)-CH_3), 167.59 (Ar-(**C**=O)), 149.43 (C2), 138.41 (C6), 131.11 (C4), 128.63 (C3), 128.14 (C1), 121.56 (C5), 20.81 (-OAc), 20.03 (-CH_3).

General procedure for the synthesis of the acetylsalicylic acid esters

Under a protective atmosphere of argon, 1.0 eq. of ASA (5.00 mmol), 1.1 eq. of the respective alcohol (5.50 mmol) and 10.0 mol-% DMAP were dissolved in 30 mL of anhydrous dichloromethane and cooled with an ice-bath. Then, an ice-cold solution of 1.05 eq. of DCC (5.25 mmol) in 10 mL of anhydrous dichloromethane were added over a period of 5 min *via* syringe. The reaction mixture was stirred for another 10 min at 0 °C, then for 3.5 h at room temperature. After completion of the reaction (TLC monitoring), the mixture was washed each three times with an aqueous 1 M HCl solution and saturated aqueous $NaHCO_3$ solution and once with brine. The organic phase was dried over anhydrous Na_2SO_4 and evaporated. Pure products were obtained after column chromatography (fluorine-substituted derivatives: petroleum ether/ethyl acetate = 7:1; chlorine-substituted derivatives: petroleum ether/ethyl acetate = 9:1; methyl-substituted derivatives: petroleum ether/ethyl acetate = 8:1).

Prop-2-en-1-yl 2-acetoxy-3-fluorobenzoate (3-F-ASA-Propene)

Pale yellow oil; yield: 60%. ^{1}H NMR (400 MHz, acetone-d_6): δ = 7.83 (ddd, ^3J = 7.9 Hz, ^4J = 1.5 Hz, $^5J_{H\text{-}F}$ = 1.5 Hz, 1H, Ar-H6), 7.56 (ddd, $^3J_{H\text{-}F}$ = 9.8 Hz, ^3J = 8.3 Hz, ^4J = 1.6 Hz, 1H, Ar-H4), 7.44 (ddd, ^3J = 8.1 Hz, ^3J = 8.1 Hz, $^4J_{H\text{-}F}$ = 5.1 Hz, 1H, Ar-H5), 6.06 (ddt, ^3J = 17.1 Hz, ^3J = 10.3 Hz, ^3J = 5.7 Hz, 1H, -CH=), 5.43 (ddt, ^3J = 17.2 Hz, ^2J = ^4J = 1.5 Hz, 1H, =$CH_{2,\,trans}$), 5.30 (ddt, ^3J = 10.5 Hz, ^2J = ^4J = 1.3 Hz, 1H, =$CH_{2,\,cis}$), 4.81 (ddd, ^3J = 5.7 Hz, ^4J = 1.4 Hz, ^4J = 1.4 Hz, 2H, -OCH_2-), 2.33 (s, 3H, -OAc). ^{13}C NMR (101 MHz, acetone-d_6): δ = 168.76 (-(**C**=O)-CH_3), 163.98 (d, $^4J_{C\text{-}F}$ = 3.6 Hz, Ar-(**C**=O)), 155.91 (d, $^1J_{C\text{-}F}$ = 247.5 Hz, C3), 139.49 (d, $^2J_{C\text{-}F}$ = 14.5 Hz, C2), 133.19 (C2′), 127.78 (d, $^3J_{C\text{-}F}$ = 7.9 Hz, C5), 127.54 (d, $^4J_{C\text{-}F}$ = 3.6 Hz, C6), 126.81 (C1), 121.53 (d, $^2J_{C\text{-}F}$ = 19.5 Hz, C4), 118.93 (C3′), 66.60 (C1′), 20.47 (-OAc). HR-ESI-MS (*m/z*): 261.0526 ([M+Na]$^+$, calcd for $C_{12}H_{11}FO_4Na$: 261.0534).

Prop-2-en-1-yl 2-acetoxy-4-fluorobenzoate (4-F-ASA-Propene)

Colorless oil; yield: 61%. ^{1}H NMR (400 MHz, acetone-d_6): δ = 8.11 (dd, ^3J = 8.9 Hz, $^4J_{H\text{-}F}$ = 6.5 Hz, 1H, Ar-H6), 7.21 (ddd, ^3J = 8.9 Hz, $^3J_{H\text{-}F}$ = 8.1 Hz, ^4J = 2.6 Hz, 1H, Ar-H5), 7.08 (dd, $^3J_{H\text{-}F}$ = 9.3 Hz, ^4J = 2.6 Hz, 1H, Ar-H3), 6.05 (ddt, ^3J = 17.3 Hz, ^3J = 10.5 Hz, ^3J = 5.7 Hz, 1H, -CH=), 5.42 (ddt, ^3J = 17.2 Hz, ^2J = ^4J = 1.6 Hz, 1H, =$CH_{2,\,trans}$), 5.28 (ddt, ^3J = 10.4 Hz, ^2J = ^4J = 1.4 Hz, 1H, =$CH_{2,\,cis}$), 4.78 (ddd, ^3J = 5.7 Hz, ^4J = 1.5 Hz, ^4J = 1.5 Hz, 2H, -OCH_2-), 2.28 (s, 3H, -OAc). ^{13}C NMR (101 MHz, acetone-d_6): δ = 169.40 (-(**C**=O)-CH_3), 166.19 (d, $^1J_{C\text{-}F}$ = 253.6 Hz, C4), 163.89 (Ar-(**C**=O)), 153.52 (d, $^3J_{C\text{-}F}$ = 11.7 Hz, C2), 134.51 (d, $^3J_{C\text{-}F}$ = 10.2 Hz, C6), 133.32 (C2′), 121.22 (d, $^4J_{C\text{-}F}$ = 3.6 Hz, C1), 118.76 (C3′), 114.04 (d, $^2J_{C\text{-}F}$ = 21.8 Hz, C5), 112.63 (d, $^2J_{C\text{-}F}$ = 24.0 Hz, C3), 66.33 (C1′), 20.93 (-OAc). HR-ESI-MS (*m/z*): 261.0526 ([M+Na]$^+$, calcd for $C_{12}H_{11}FO_4Na$: 261.0534).

Prop-2-en-1-yl 2-acetoxy-5-fluorobenzoate (5-F-ASA-Propene)

Colorless oil; yield: 67%. ^{1}H NMR (400 MHz, acetone-d_6): δ = 7.71 (dd, $^3J_{H\text{-}F}$ = 8.9 Hz, ^4J = 3.2 Hz, 1H, Ar-H6), 7.46 (ddd, ^3J = 9.0 Hz, $^3J_{H\text{-}F}$ = 7.8 Hz, ^4J = 3.2 Hz, 1H, Ar-H4), 7.26 (dd, ^3J = 9.0 Hz, $^4J_{H\text{-}F}$ = 4.7 Hz, 1H, Ar-H3), 6.06 (ddt, ^3J = 17.3 Hz, ^3J = 10.5 Hz, ^3J = 5.7 Hz, 1H, -CH=), 5.43 (ddt, ^3J = 17.4 Hz, ^2J = ^4J = 1.6 Hz, 1H, =$CH_{2,\,trans}$), 5.30 (ddt, ^3J = 10.4 Hz, ^2J = ^4J = 1.4 Hz, 1H, =$CH_{2,\,cis}$), 4.80 (ddd, ^3J = 5.8 Hz, ^4J = 1.4 Hz, ^4J = 1.4 Hz, 2H, -OCH_2-), 2.26 (s, 3H, -OAc). ^{13}C NMR (101 MHz, acetone-d_6): δ = 169.77 (-(**C**=O)-CH_3), 163.73 (d, $^4J_{C\text{-}F}$ = 2.5 Hz, Ar-(**C**=O)), 160.51 (d, $^1J_{C\text{-}F}$ = 244.1 Hz, C5), 147.76 (d, $^4J_{C\text{-}F}$ = 2.9 Hz, C2), 133.14 (C2′), 126.91 (d, $^3J_{C\text{-}F}$ = 8.6 Hz, C3), 126.03 (d, $^3J_{C\text{-}F}$ = 7.4 Hz, C1), 121.52 (d, $^2J_{C\text{-}F}$ = 23.3 Hz, C4), 118.96 (C3′), 118.39 (d, $^2J_{C\text{-}F}$ = 25.4 Hz, C6), 66.65 (C1′), 20.90 (-OAc). HR-ESI-MS (*m/z*): 261.0526 ([M+Na]$^+$, calcd for $C_{12}H_{11}FO_4Na$: 261.0534).

Prop-2-en-1-yl 2-acetoxy-6-fluorobenzoate (6-F-ASA-Propene)

Yellowish oil; yield: 24%. ^{1}H NMR (400 MHz, acetone-d_6): δ = 7.62 (ddd, ^3J = 8.4 Hz, ^3J = 8.4 Hz, $^4J_{H\text{-}F}$ = 6.2 Hz, 1H, Ar-H4), 7.20 (ddd, $^3J_{H\text{-}F}$ = 9.5 Hz, ^3J = 8.5 Hz, ^4J = 1.0 Hz, 1H, Ar-H5), 7.10 (ddd, ^3J = 8.3 Hz, ^4J = 1.0 Hz, $^5J_{H\text{-}F}$ = 1.0 Hz, 1H, Ar-H3), 6.04 (ddt, ^3J = 17.3 Hz, ^3J = 10.5 Hz, ^3J = 5.6 Hz, 1H, -CH=), 5.44 (ddt, ^3J = 17.3 Hz, ^2J = ^4J = 1.6 Hz, 1H, =$CH_{2,\,trans}$), 5.29 (ddt, ^3J = 10.5 Hz, ^2J = ^4J = 1.3 Hz, 1H, =$CH_{2,\,cis}$), 4.82 (ddd, ^3J = 5.7 Hz, ^4J = 1.5 Hz, ^4J = 1.5 Hz, 2H, -OCH_2-), 2.24 (s, 3H, -OAc). ^{13}C NMR (101 MHz, acetone-d_6): δ = 169.14 (-(**C**=O)-CH_3), 162.54 (Ar-(**C**=O)), 161.29 (d, $^1J_{C\text{-}F}$ = 252.7 Hz, C6), 150.81 (d, $^3J_{C\text{-}F}$ = 5.1 Hz, C2), 133.58 (d, $^3J_{C\text{-}F}$ = 10.2 Hz, C4), 132.99 (C2′), 120.46 (d, $^4J_{C\text{-}F}$ = 3.6 Hz, C3), 118.86 (C3′), 116.49 (d, $^2J_{C\text{-}F}$ = 17.4 Hz, C1), 114.50 (d, $^2J_{C\text{-}F}$ = 21.9 Hz, C5), 66.81 (C1′), 20.73 (-OAc). HR-ESI-MS (*m/z*): 261.0526 ([M+Na]$^+$, calcd for $C_{12}H_{11}FO_4Na$: 261.0534), 499.1162 ([2M+Na]$^+$, calcd for $(C_{12}H_{11}FO_4)_2Na$: 499.1175).

But-3-en-1-yl 2-acetoxy-3-fluorobenzoate (3-F-ASA-Butene)

Colorless oil; yield: 67%. ^{1}H NMR (400 MHz, acetone-d_6): δ = 7.80 (ddd, ^3J = 7.9 Hz, ^4J = 1.5 Hz, $^5J_{H\text{-}F}$ = 1.5 Hz, 1H, Ar-H6), 7.54 (ddd, $^3J_{H\text{-}F}$ = 9.9 Hz, ^3J = 8.3 Hz, ^4J = 1.6 Hz, 1H, Ar-H4), 7.42 (ddd, ^3J = 8.1 Hz, ^3J = 8.1 Hz, $^4J_{H\text{-}F}$ = 5.1 Hz, 1H, Ar-H5), 5.90 (ddt, ^3J = 17.1 Hz, ^3J = 10.3 Hz, ^3J = 6.7 Hz, 1H, -CH=), 5.18 (ddt, ^3J = 17.2 Hz, ^2J = ^4J = 1.7 Hz, 1H, =$CH_{2,\,trans}$), 5.09 (ddt, ^3J = 10.3 Hz, ^2J = 2.2 Hz, ^4J = 1.2 Hz, 1H, =$CH_{2,\,cis}$), 4.35 (t, ^3J = 6.7 Hz, 2H, -OCH_2-), 2.52 (dtdd, ^3J = 6.7 Hz, ^3J = 6.7 Hz, ^4J = 1.4 Hz, ^4J = 1.4 Hz, 2H, -CH_2-), 2.35 (s, 3H, -OAc). ^{13}C NMR (101 MHz, acetone-d_6): δ = 168.72 (-(**C**=O)-CH_3), 164.19 (d, $^4J_{C\text{-}F}$ = 3.0 Hz, Ar-(**C**=O)), 155.88 (d, $^1J_{C\text{-}F}$ = 247.2 Hz, C3), 139.49 (d, $^2J_{C\text{-}F}$ = 14.6 Hz, C2), 135.13 (C3′), 127.69 (d, $^3J_{C\text{-}F}$ = 7.5 Hz, C5), 127.47 (d, $^4J_{C\text{-}F}$ = 3.7 Hz, C6), 126.88 (C1), 121.39 (d, $^2J_{C\text{-}F}$ = 19.5 Hz, C4), 117.68 (C4′), 65.15 (C1′), 33.72 (C2′), 20.48 (-OAc). HR-ESI-MS (*m/z*): 275.0683 ($[M+Na]^+$, calcd for $C_{13}H_{13}FO_4Na$: 275.0690).

But-3-en-1-yl 2-acetoxy-4-fluorobenzoate (4-F-ASA-Butene)

Colorless oil; yield: 40%. ^{1}H NMR (400 MHz, acetone-d_6): δ = 8.08 (dd, ^3J = 8.9 Hz, $^4J_{H\text{-}F}$ = 6.5 Hz, 1H, Ar-H6), 7.19 (ddd, ^3J = 8.9 Hz, $^3J_{H\text{-}F}$ = 8.1 Hz, ^4J = 2.6 Hz, 1H, Ar-H5), 7.06 (dd, $^3J_{H\text{-}F}$ = 9.4 Hz, ^4J = 2.6 Hz, 1H, Ar-H3), 5.89 (ddt, ^3J = 17.0 Hz, ^3J = 10.3 Hz, ^3J = 6.7 Hz, 1H, -CH=), 5.17 (ddt, ^3J = 17.2 Hz, ^2J = ^4J = 1.7 Hz, 1H, =$CH_{2,\,trans}$), 5.08 (ddt, ^3J = 10.3 Hz, ^2J = 2.3 Hz, ^4J = 1.3 Hz, 1H, =$CH_{2,\,cis}$), 4.32 (t, ^3J = 6.7 Hz, 2H, -OCH_2-), 2.50 (dtdd, ^3J = 6.7 Hz, ^3J = 6.7 Hz, ^4J = 1.4 Hz, ^4J = 1.4 Hz, 2H, -CH_2-), 2.29 (s, 3H, -OAc). ^{13}C NMR (101 MHz, acetone-d_6): δ = 169.34 (-(**C**=O)-CH_3), 166.12 (d, $^1J_{C\text{-}F}$ = 253.5 Hz, C4), 164.10 (Ar-(**C**=O)), 153.53 (d, $^3J_{C\text{-}F}$ = 11.7 Hz, C2), 135.20 (C3′), 134.41 (d, $^3J_{C\text{-}F}$ = 10.5 Hz, C6), 121.29 (d, $^4J_{C\text{-}F}$ = 3.6 Hz, C1), 117.62 (C4′), 113.95 (d, $^2J_{C\text{-}F}$ = 21.7 Hz, C5), 112.59 (d, $^2J_{C\text{-}F}$ = 24.1 Hz, C3), 64.87 (C1′), 33.75 (C2′), 20.95 (-OAc). HR-ESI-MS (*m/z*): 275.0683 ($[M+Na]^+$, calcd for $C_{13}H_{13}FO_4Na$: 275.0690).

But-3-en-1-yl 2-acetoxy-5-fluorobenzoate (5-F-ASA-Butene)

Colorless oil; yield: 86%. ^{1}H NMR (400 MHz, acetone-d_6): δ = 7.69 (dd, $^3J_{H\text{-}F}$ = 8.9 Hz, ^4J = 3.2 Hz, 1H, Ar-H6), 7.45 (ddd, ^3J = 8.9 Hz, $^3J_{H\text{-}F}$ = 7.8 Hz, ^4J = 3.2 Hz, 1H, Ar-H4), 7.25 (dd, ^3J = 9.0 Hz, $^4J_{H\text{-}F}$ = 4.8 Hz, 1H, Ar-H3), 5.90 (ddt, ^3J = 17.1 Hz, ^3J = 10.3 Hz, ^3J = 6.8 Hz, 1H, -CH=), 5.18 (ddt, ^3J = 17.2 Hz, ^2J = ^4J = 1.7 Hz, 1H, =$CH_{2,\,trans}$), 5.09 (ddt, ^3J = 10.3 Hz, ^2J = 2.2 Hz, ^4J = 1.2 Hz, 1H, =$CH_{2,\,cis}$), 4.33 (t, ^3J = 6.7 Hz, 2H, -OCH_2-), 2.52 (dtdd, ^3J = 6.7 Hz, ^3J = 6.7 Hz, ^4J = 1.4 Hz, ^4J = 1.4 Hz, 2H, -CH_2-), 2.28 (s, 3H, -OAc). ^{13}C NMR (101 MHz, acetone-d_6): δ = 169.72 (-(**C**=O)-CH_3), 163.90 (d, $^4J_{C\text{-}F}$ = 2.0 Hz, Ar-(**C**=O)), 160.46 (d, $^1J_{C\text{-}F}$ = 243.8 Hz, C5), 147.80 (d, $^4J_{C\text{-}F}$ = 3.0 Hz, C2), 135.16 (C3′), 126.88 (d, $^3J_{C\text{-}F}$ = 8.3 Hz, C3), 126.09 (d, $^3J_{C\text{-}F}$ = 7.4 Hz, C1), 121.42 (d, $^2J_{C\text{-}F}$ = 23.4 Hz, C4), 118.31 (d, $^2J_{C\text{-}F}$ = 25.3 Hz, C6), 117.67 (C4′), 65.16 (C1′), 33.70 (C2′), 20.91 (-OAc). HR-ESI-MS (*m/z*): 275.0685 ($[M+Na]^+$, calcd for $C_{13}H_{13}FO_4Na$: 275.0690).

But-3-en-1-yl 2-acetoxy-6-fluorobenzoate (6-F-ASA-Butene)

Colorless oil; yield: 56%. ^{1}H NMR (400 MHz, acetone-d_6): δ = 7.60 (ddd, ^3J = 8.4 Hz, ^3J = 8.4 Hz, $^4J_{H\text{-}F}$ = 6.2 Hz, 1H, Ar-H4), 7.18 (ddd, $^3J_{H\text{-}F}$ = 9.5 Hz, ^3J = 8.4 Hz, ^4J = 1.0 Hz, 1H, Ar-H5), 7.09 (ddd, ^3J = 8.2 Hz, ^4J = 1.0 Hz, $^5J_{H\text{-}F}$ = 1.0 Hz, 1H, Ar-H3), 5.88 (ddt, ^3J = 17.1 Hz, ^3J = 10.3 Hz, ^3J = 6.7 Hz, 1H, -CH=), 5.17 (ddt, ^3J = 17.2 Hz, ^2J = ^4J = 1.7 Hz, 1H, =$CH_{2,\,trans}$), 5.08 (ddt, ^3J = 10.3 Hz, ^2J = 2.0 Hz, ^4J = 1.3 Hz, 1H, =$CH_{2,\,cis}$), 4.36 (t, ^3J = 6.7 Hz, 2H, -OCH_2-), 2.49 (dtdd, ^3J = 6.7 Hz, ^3J = 6.7 Hz, ^4J = 1.4 Hz, ^4J = 1.4 Hz, 2H, -CH_2-), 2.26 (s, 3H, -OAc). ^{13}C NMR (101 MHz, acetone-d_6): δ = 169.12 (-(**C**=O)-CH_3), 162.90 (Ar-(**C**=O)), 161.22 (d, $^1J_{C\text{-}F}$ = 253.9 Hz, C6), 150.76 (d, $^3J_{C\text{-}F}$ = 5.3 Hz, C2), 134.97 (C3′), 133.42 (d, $^3J_{C\text{-}F}$ = 10.2 Hz, C4), 120.39 (d, $^4J_{C\text{-}F}$ = 3.0 Hz, C3), 117.67 (C4′), 116.67 (d, $^2J_{C\text{-}F}$ = 17.5 Hz, C1), 114.44 (d, $^2J_{C\text{-}F}$ = 21.9 Hz, C5), 65.55 (C1′), 33.68 (C2′), 20.75 (-OAc). HR-ESI-MS (*m/z*): 275.0682 ($[M+Na]^+$, calcd for $C_{13}H_{13}FO_4Na$: 275.0690).

Prop-2-en-1-yl 2-acetoxy-3-chlorobenzoate (3-Cl-ASA-Propene)

Colorless crystals; yield: 48%. ^{1}H NMR (400 MHz, acetone-d_6): δ = 7.98 (dd, ^3J = 7.9 Hz, ^4J = 1.6 Hz, 1H, Ar-H6), 7.80 (dd, ^3J = 8.1 Hz, ^4J = 1.6 Hz, 1H, Ar-H4), 7.44 (dd, ^3J = 8.0 Hz, ^4J = 8.0 Hz, 1H, Ar-H5), 6.06 (ddt, ^3J = 17.2 Hz, ^3J = 10.5 Hz, ^3J = 5.7 Hz, 1H, -CH=), 5.43 (ddt, ^3J = 17.2 Hz, ^2J = 1.6 Hz, ^4J = 1.6 Hz, 1H, =$CH_{2,\,trans}$), 5.30 (ddt, ^3J = 10.5 Hz, ^2J = 1.4 Hz, ^4J = 1.4 Hz, 1H, =$CH_{2,\,cis}$), 4.81 (ddd, ^3J = 5.7 Hz, ^4J = 1.4 Hz, ^4J = 1.4 Hz, 2H, -OCH_2-), 2.34 (s, 3H, -OAc). ^{13}C NMR (101 MHz, acetone-d_6): δ = 168.52 (-(**C**=O)-CH_3), 163.97 (Ar-(**C**=O)), 147.90 (C2), 135.10 (C4), 133.16 (C2′), 131.03 (C6), 129.55 (C3), 127.85 (C5), 126.66 (C1), 118.94 (C3′), 66.65 (C1′), 20.57 (-OAc).

Prop-2-en-1-yl 2-acetoxy-4-chlorobenzoate (4-Cl-ASA-Propene)

Colorless crystals; yield: 36%. ^{1}H NMR (400 MHz, acetone-d_6): δ = 8.03 (d, ^3J = 8.5 Hz, 1H, Ar-H6), 7.45 (dd, ^3J = 8.5 Hz, ^4J = 2.1 Hz, 1H, Ar-H5), 7.32 (d, ^4J = 2.1 Hz, 1H, Ar-H3), 6.05 (ddt, ^3J = 16.3 Hz, ^3J = 11.2 Hz,

^{3}J = 5.7 Hz, 1H, -CH=), 5.42 (ddt, ^{3}J = 17.2 Hz, ^{2}J = 1.6 Hz, ^{4}J = 1.6 Hz, 1H, =$CH_{2,\,trans}$), 5.29 (ddt, ^{3}J = 10.4 Hz, ^{2}J = 1.4 Hz, ^{4}J = 1.4 Hz, 1H, =$CH_{2,\,cis}$), 4.79 (ddd, ^{3}J = 5.8 Hz, ^{4}J = 1.4 Hz, ^{4}J = 1.4 Hz, 2H, -OCH_2-), 2.28 (s, 3H, -OAc). ^{13}C NMR (101 MHz, acetone-d_6): δ = 169.43 (-(**C**=O)-CH_3), 163.97 (Ar-(**C**=O)), 152.40 (C2), 139.56 (C4), 133.64 (C6), 133.22 (C2′), 127.16 (C5), 125.30 (C3), 123.47 (C1), 118.87 (C3′), 66.47 (C1′), 20.92 (-OAc).

Prop-2-en-1-yl 2-acetoxy-5-chlorobenzoate (5-Cl-ASA-Propene)

Colorless crystals; yield: 39%. ^{1}H NMR (400 MHz, acetone-d_6): δ = 7.97 (d, ^{4}J = 2.7 Hz, 1H, Ar-H6), 7.69 (dd, ^{3}J = 8.7 Hz, ^{4}J = 2.7 Hz, 1H, Ar-H4), 7.26 (d, ^{3}J = 8.7 Hz, 1H, Ar-H3), 6.06 (ddt, ^{3}J = 16.5 Hz, ^{3}J = 10.5 Hz, ^{3}J = 5.7 Hz, 1H, -CH=), 5.43 (ddt, ^{3}J = 17.1 Hz, ^{2}J = 1.6 Hz, ^{4}J = 1.6 Hz, 1H, =$CH_{2,\,trans}$), 5.30 (ddt, ^{3}J = 10.4 Hz, ^{2}J = 1.4 Hz, ^{4}J = 1.4 Hz, 1H, =$CH_{2,\,cis}$), 4.80 (ddd, ^{3}J = 5.8 Hz, ^{4}J = 1.5 Hz, ^{4}J = 1.5 Hz, 2H, -OCH_2-), 2.27 (s, 3H, -OAc). ^{13}C NMR (101 MHz, acetone-d_6): δ = 169.57 (-(**C**=O)-CH_3), 163.65 (Ar-(**C**=O)), 150.33 (C2), 134.58 (C4), 133.13 (C2′), 131.73 and 131.68 (C1 and C6), 126.86 and 126.17 (C3 and C5), 119.05 (C3′), 66.72 (C1′), 20.91 (-OAc).

Prop-2-en-1-yl 2-acetoxy-6-chlorobenzoate (6-Cl-ASA-Propene)

Colorless crystals; yield: 36%. ^{1}H NMR (400 MHz, acetone-d_6): δ = 7.54 (dd, ^{3}J = 8.2 Hz, ^{3}J = 8.2 Hz, 1H, Ar-H4), 7.43 (dd, ^{3}J = 8.1 Hz, ^{4}J = 1.0 Hz, 1H, Ar-H5), 7.25 (dd, ^{3}J = 8.2 Hz, ^{4}J = 1.0 Hz, 1H, Ar-H3), 6.05 (ddt, ^{3}J = 17.2 Hz, ^{3}J = 10.4 Hz, ^{3}J = 5.8 Hz, 1H, -CH=), 5.45 (ddt, ^{3}J = 17.2 Hz, ^{2}J = 1.6 Hz, ^{4}J = 1.6 Hz, 1H, =$CH_{2,\,trans}$), 5.31 (ddt, ^{3}J = 10.5 Hz, ^{2}J = 1.3 Hz, ^{4}J = 1.3 Hz, 1H, =$CH_{2,\,cis}$), 4.85 (ddd, ^{3}J = 5.8 Hz, ^{4}J = 1.4 Hz, ^{4}J = 1.4 Hz, 2H, -OCH_2-), 2.24 (s, 3H, -OAc). ^{13}C NMR (101 MHz, acetone-d_6): δ = 168.89 (-(**C**=O)-CH_3), 164.20 (Ar-(**C**=O)), 149.78 (C2), 132.88 (C2′), 132.30 (C4), 132.01 (C6), 128.30 (C1), 127.90 (C5), 123.21 (C3), 119.21 (C3′), 67.04 (C1′), 20.65 (-OAc).

But-3-en-1-yl 2-acetoxy-3-chlorobenzoate (3-Cl-ASA-Butene)

Colorless crystals; yield: 66%. ^{1}H NMR (400 MHz, acetone-d_6): δ = 7.95 (dd, ^{3}J = 7.9 Hz, ^{4}J = 1.6 Hz, 1H, Ar-H6), 7.78 (dd, ^{3}J = 8.0 Hz, ^{4}J = 1.6 Hz, 1H, Ar-H4), 7.42 (dd, ^{3}J = 8.0 Hz, ^{3}J = 8.0 Hz, 1H, Ar-H5), 5.90 (ddt, ^{3}J = 17.1 Hz, ^{3}J = 10.3 Hz, ^{3}J = 6.7 Hz, 1H, -CH=), 5.18 (ddt, ^{3}J = 17.2 Hz, ^{2}J = 1.7 Hz, ^{4}J = 1.7 Hz, 1H, =$CH_{2,\,trans}$), 5.09 (ddt, ^{3}J = 10.3 Hz, ^{2}J = 2.0 Hz, ^{4}J = 1.1 Hz, 1H, =$CH_{2,\,cis}$), 4.35 (t, ^{3}J = 6.7 Hz, 2H, -OCH_2-), 2.51 (dtdd, ^{3}J = 6.7 Hz, ^{3}J = 6.7 Hz, ^{4}J = 1.5 Hz, ^{4}J = 1.5 Hz, 2H, -CH_2-), 2.35 (s, 3H, -OAc). ^{13}C NMR (101 MHz, acetone-d_6): δ = 168.49 (-(**C**=O)-CH_3), 164.18 (Ar-(**C**=O)), 147.91 (C2), 135.13 (C3′), 134.98 (C4), 130.95 (C6), 129.52 (C3), 127.83 (C5), 126.74 (C1), 117.69 (C4′), 65.20 (C1′), 33.71 (C2′), 20.59 (-OAc).

But-3-en-1-yl 2-acetoxy-4-chlorobenzoate (4-Cl-ASA-Butene)

Colorless crystals; yield: 54%. ^{1}H NMR (400 MHz, acetone-d_6): δ = 8.01 (d, ^{3}J = 8.5 Hz, 1H, Ar-H6), 7.44 (dd, ^{3}J = 8.5 Hz, ^{4}J = 2.1 Hz, 1H, Ar-H5), 7.31 (d, ^{4}J = 2.1 Hz, 1H, Ar-H3), 5.89 (ddt, ^{3}J = 17.1 Hz, ^{3}J = 10.3 Hz, ^{3}J = 6.8 Hz, 1H, -CH=), 5.17 (ddt, ^{3}J = 17.2 Hz, ^{2}J = 1.7 Hz, ^{4}J = 1.7 Hz, 1H, =$CH_{2,\,trans}$), 5.08 (ddt, ^{3}J = 10.3 Hz, ^{2}J = 2.1 Hz, ^{4}J = 1.3 Hz, 1H, =$CH_{2,\,cis}$), 4.33 (t, ^{3}J = 6.7 Hz, 2H, -OCH_2-), 2.50 (dtdd, ^{3}J = 6.7 Hz, ^{3}J = 6.7 Hz, ^{4}J = 1.4 Hz, ^{4}J = 1.4 Hz, 2H, -CH_2-), 2.30 (s, 3H, -OAc). ^{13}C NMR (101 MHz, acetone-d_6): δ = 169.40 (-(**C**=O)-CH_3), 164.18 (Ar-(**C**=O)), 152.43 (C2), 139.44 (C4), 135.15 (C3′), 133.56 (C6), 127.10 (C5), 125.27 (C3), 123.56 (C1), 117.66 (C4′), 65.01 (C1′), 33.72 (C2′), 20.93 (-OAc).

But-3-en-1-yl 2-acetoxy-5-chlorobenzoate (5-Cl-ASA-Butene)

Colorless crystals; yield: 32%. ^{1}H NMR (400 MHz, acetone-d_6): δ = 7.95 (d, ^{4}J = 2.7 Hz, 1H, Ar-H6), 7.68 (dd, ^{3}J = 8.6 Hz, ^{4}J = 2.7 Hz, 1H, Ar-H4), 7.25 (d, ^{3}J = 8.6 Hz, 1H, Ar-H3), 5.90 (ddt, ^{3}J = 17.1 Hz, ^{3}J = 10.3 Hz, ^{3}J = 6.8 Hz, 1H, -CH=), 5.19 (ddt, ^{3}J = 17.2 Hz, ^{2}J = 1.6 Hz, ^{4}J = 1.6 Hz, 1H, =$CH_{2,\,trans}$), 5.09 (ddt, ^{3}J = 10.3 Hz, ^{2}J = 2.1 Hz, ^{4}J = 1.1 Hz, 1H, =$CH_{2,\,cis}$), 4.34 (t, ^{3}J = 6.7 Hz, 2H, -OCH_2-), 2.52 (dtdd, ^{3}J = 6.7 Hz, ^{3}J = 6.7 Hz, ^{4}J = 1.4 Hz, ^{4}J = 1.4 Hz, 2H, -CH_2-), 2.29 (s, 3H, -OAc). ^{13}C NMR (101 MHz, acetone-d_6): δ = 168.63 (-(**C**=O)-CH_3), 162.93 (Ar-(**C**=O)), 149.46 (C2), 134.24 (C3′), 133.58 (C4), 130.77 (C6), 130.71 (C5), 125.94 (C3), 125.34 (C1), 116.78 (C4′), 64.31 (C1′), 32.79 (C2′), 20.03 (-OAc).

But-3-en-1-yl 2-acetoxy-6-chlorobenzoate (6-Cl-ASA-Butene)

Colorless crystals; yield: 41%. ^{1}H NMR (400 MHz, acetone-d_6): δ 7.53 (dd, ^{3}J = 8.2 Hz, ^{4}J = 8.2 Hz, 1H, Ar-H4), 7.42 (dd, ^{3}J = 8.2 Hz, ^{4}J = 1.0 Hz, 1H, Ar-H5), 7.25 (dd, ^{3}J = 8.3 Hz, ^{4}J = 1.0 Hz, 1H, Ar-H3), 5.88 (ddt, ^{3}J = 17.1 Hz, ^{3}J = 10.3 Hz, ^{3}J = 6.7 Hz, 1H, -CH=), 5.17 (ddt, ^{3}J = 17.2 Hz, ^{2}J = 1.7 Hz, ^{4}J = 1.7 Hz, 1H, =$CH_{2,\,trans}$), 5.09 (ddt, ^{3}J = 10.3 Hz, ^{2}J = 1.5 Hz, ^{4}J = 1.5 Hz, 1H, =$CH_{2,\,cis}$), 4.39 (t, ^{3}J = 6.7 Hz, 2H, -OCH_2-), 2.51 (dtdd, ^{3}J = 6.7 Hz, ^{3}J = 6.7 Hz, ^{4}J = 1.4 Hz, ^{4}J = 1.4 Hz, 2H, -CH_2-), 2.25 (s, 3H, -OAc). ^{13}C NMR (101 MHz, acetone-

d_6): δ = 168.87 (-(**C**=O)-CH_3), 164.52 (Ar-(**C**=O)), 149.70 (C2), 134.94 (C3′), 132.17 (C4), 131.95 (C6), 128.50 (C1), 127.83 (C5), 123.15 (C3), 117.73 (C4′), 65.79 (C1′), 33.61 (C2′), 20.69 (-OAc).

Prop-2-en-1-yl 2-acetoxy-3-methylbenzoate (3-M-ASA-Propene)

Pale yellow oil; yield: 40%. ^{1}H NMR (400 MHz, acetone-d_6): δ = 7.88-7.80 (m, 1H, Ar-H6), 7.53 (dqd, ^{3}J = 7.5 Hz, ^{4}J = 1.5 Hz, ^{4}J = 0.9 Hz, 1H, Ar-H4), 7.28 (dd, ^{3}J = 7.7 Hz, ^{3}J = 7.7 Hz, 1H, Ar-H5), 6.06 (ddt, ^{3}J = 17.3 Hz, ^{3}J = 10.4 Hz, ^{3}J = 5.7 Hz, 1H, -CH=), 5.42 (ddt, ^{3}J = 17.2 Hz, ^{2}J = ^{4}J = 1.6 Hz, 1H, =$CH_{2,\,trans}$), 5.28 (ddt, ^{3}J = 10.5 Hz, ^{2}J = ^{4}J = 1.4 Hz, 1H, =$CH_{2,\,cis}$), 4.77 (ddd, ^{3}J = 5.7 Hz, ^{4}J = 1.5 Hz, ^{4}J = 1.5 Hz, 2H, -OCH_2-), 2.30 (s, 3H, -OAc), 2.21 (s_{br}, 3H, -CH_3). ^{13}C NMR (101 MHz, acetone-d_6): δ = 169.24 (-(**C**=O)-CH_3), 164.94 (Ar-(**C**=O)), 150.21 (C2), 136.14 (C4), 133.48 (C2′), 133.23 (C3), 129.94 (C6), 126.46 (C5), 124.48 (C1), 118.57 (C3′), 66.16 (C1′), 20.81 (-OAc), 16.13 (-CH_3). HR-ESI-MS (*m/z*): 257.0785 ([M+Na]$^+$, calcd for $C_{13}H_{14}O_4Na$: 257.0784), 491.1674 ([2M+Na]$^+$, calcd for $(C_{13}H_{14}O_4)_2Na$: 491.1676).

Prop-2-en-1-yl 2-acetoxy-4-methylbenzoate (4-M-ASA-Propene)

Pale yellow oil; yield: 32%. ^{1}H NMR (400 MHz, acetone-d_6): δ = 7.91 (d, ^{3}J = 8.0 Hz, 1H, Ar-H6), 7.21 (dqd, ^{3}J = 7.9 Hz, ^{4}J = 1.7 Hz, ^{4}J = 0.8 Hz, 1H, Ar-H5), 7.05-6.95 (m, 1H, Ar-H3), 6.05 (ddt, ^{3}J = 17.2 Hz, ^{3}J = 10.4 Hz, ^{3}J = 5.7 Hz, 1H, -CH=), 5.41 (ddt, ^{3}J = 17.2 Hz, ^{2}J = ^{4}J = 1.6 Hz, 1H, =$CH_{2,\,trans}$), 5.27 (ddt, ^{3}J = 10.5 Hz, ^{2}J = ^{4}J = 1.4 Hz, 1H, =$CH_{2,\,cis}$), 4.76 (ddd, ^{3}J = 5.7 Hz, ^{4}J = 1.5 Hz, ^{4}J = 1.5 Hz, 2H, -OCH_2-), 2.40 (s_{br}, 3H, -CH_3), 2.25 (s, 3H, -OAc). ^{13}C NMR (101 MHz, acetone-d_6): δ = 169.69 (-(**C**=O)-CH_3), 164.65 (Ar-(**C**=O)), 151.80 (C2), 146.13 (C4), 133.54 (C2′), 132.24 (C6), 127.52 (C5), 125.30 (C3), 121.55 (C1), 118.51 (C3′), 66.01 (C1′), 21.25 (-CH_3), 21.03 (-OAc). HR-ESI-MS (*m/z*): 257.0786 ([M+Na]$^+$, calcd for $C_{13}H_{14}O_4Na$: 257.0784).

Prop-2-en-1-yl 2-acetoxy-5-methylbenzoate (5-M-ASA-Propene)

Pale yellow oil; yield: 54%. ^{1}H NMR (400 MHz, acetone-d_6): δ = 7.81 (d, ^{4}J = 2.3 Hz, 1H, Ar-H6), 7.21 (dq, ^{3}J = 8.0 Hz, ^{4}J = 0.8 Hz, 1H, Ar-H4), 7.06 (d, ^{3}J = 8.2 Hz, 1H, Ar-H3), 6.05 (ddt, ^{3}J = 17.3 Hz, ^{3}J = 10.5 Hz, ^{3}J = 5.7 Hz, 1H, -CH=), 5.42 (ddt, ^{3}J = 17.2 Hz, ^{2}J = ^{4}J = 1.7 Hz, 1H, =$CH_{2,\,trans}$), 5.28 (ddt, ^{3}J = 10.4 Hz, ^{2}J = ^{4}J = 1.4 Hz, 1H, =$CH_{2,\,cis}$), 4.77 (ddd, ^{3}J = 5.7 Hz, ^{4}J = 1.5 Hz, ^{4}J = 1.5 Hz, 2H, -OCH_2-), 2.38 (s_{br}, 3H, -CH_3), 2.24 (s, 3H, -OAc). ^{13}C NMR (101 MHz, acetone-d_6): δ = 169.79 (-(**C**=O)-CH_3), 164.85 (Ar-(**C**=O)), 149.50 (C2), 136.64 (C5), 135.26 (C4), 133.47 (C2′), 132.44 (C6), 124.63 (C3), 124.12 (C1), 118.63 (C3′), 66.16 (C1′), 20.99 (-OAc), 20.64 (-CH_3). HR-ESI-MS (*m/z*): 257.0787 ([M+Na]$^+$, calcd for $C_{13}H_{14}O_4Na$: 257.0784), 491.1679 ([2M+Na]$^+$, calcd for $(C_{13}H_{14}O_4)_2Na$: 491.1676).

Prop-2-en-1-yl 2-acetoxy-6-methylbenzoate (6-M-ASA-Propene)

Pale yellow oil; yield: 82%. ^{1}H NMR (400 MHz, acetone-d_6): δ = 7.40 (dd, ^{3}J = 7.9 Hz, ^{3}J = 7.9 Hz, 1H, Ar-H4), 7.18 (d, ^{3}J = 7.7 Hz, 1H, Ar-H5), 7.03 (d, ^{3}J = 8.1 Hz, 1H, Ar-H3), 6.05 (ddt, ^{3}J = 17.3 Hz, ^{3}J = 10.5 Hz, ^{3}J = 5.8 Hz, 1H, -CH=), 5.43 (ddt, ^{3}J = 17.3 Hz, ^{2}J = ^{4}J = 1.6 Hz, 1H, =$CH_{2,\,trans}$), 5.29 (ddt, ^{3}J = 10.5 Hz, ^{2}J = ^{4}J = 1.4 Hz, 1H, =$CH_{2,\,cis}$), 4.80 (ddd, ^{3}J = 5.8 Hz, ^{4}J = 1.4 Hz, ^{4}J = 1.4 Hz, 2H, -OCH_2-), 2.37 (s_{br}, 3H, -CH_3), 2.21 (s, 3H, -OAc). ^{13}C NMR (101 MHz, acetone-d_6): δ = 169.22 (-(**C**=O)-CH_3), 166.57 (Ar-(**C**=O)), 149.43 (C2), 138.56 (C6), 133.29 (C2′), 131.40 (C4), 128.66 (C5), 127.71 (C1), 121.62 (C3), 118.97 (C3′), 66.43 (C1′), 20.81 (-OAc), 19.85 (-CH_3). HR-ESI-MS (*m/z*): 257.0811 ([M+Na]$^+$, calcd for $C_{13}H_{14}O_4Na$: 257.0784).

But-3-en-1-yl 2-acetoxy-3-methylbenzoate (3-M-ASA-Butene)

Colorless oil; yield: 64% ^{1}H NMR (400 MHz, acetone-d_6): δ = 7.81 (dd, ^{3}J = 7.8 Hz, ^{4}J = 1.1 Hz, 1H, Ar-H6), 7.52 (dq, ^{3}J = 7.6 Hz, ^{4}J = 0.8 Hz, 1H, Ar-H4), 7.27 (dd, ^{3}J = 7.7 Hz, ^{3}J = 7.7 Hz, 1H, Ar-H5), 5.90 (ddt, ^{3}J = 17.1 Hz, ^{3}J = 10.3 Hz, ^{3}J = 6.8 Hz, 1H, -CH=), 5.17 (ddt, ^{3}J = 17.2 Hz, ^{2}J = 1.7 Hz, ^{4}J = 1.7 Hz, 1H, =$CH_{2,\,trans}$), 5.08 (ddt, ^{3}J = 10.3 Hz, ^{2}J = 2.2 Hz, ^{4}J = 1.3 Hz, 1H, =$CH_{2,\,cis}$), 4.31 (t, ^{3}J = 6.7 Hz, 2H, -OCH_2-), 2.50 (dtdd, ^{3}J = 6.6 Hz, ^{3}J = 6.6 Hz, ^{4}J = 1.3 Hz, ^{4}J = 1.3 Hz, 2H, -CH_2-), 2.31 (s, 3H, -OAc), 2.21 (s_{br}, 3H, -CH_3). ^{13}C NMR (101 MHz, acetone-d_6): δ = 169.21 (-(**C**=O)-CH_3), 165.15 (Ar-(**C**=O)), 150.24 (C2), 136.02 (C4), 135.29 (C3′), 133.18 (C3), 129.87 (C6), 126.40 (C5), 124.57 (C1), 117.56 (C4′), 64.69 (C1′), 33.80 (C2′), 20.82 (-OAc), 16.12 (-CH_3). HR-ESI-MS (*m/z*): 392.1378 ([3M+H+K]$^{2+}$, calcd for $C_{42}H_{49}O_{12}K$: 392.1422).

But-3-en-1-yl 2-acetoxy-4-methylbenzoate (4-M-ASA-Butene)

Colorless oil; yield: 34%. ^{1}H NMR (400 MHz, acetone-d_6): δ = 7.89 (d, ^{3}J = 8.0 Hz, 1H, Ar-H6), 7.19 (dd, ^{3}J = 8.0 Hz, ^{4}J = 0.8 Hz, 1H, Ar-H5), 7.00 (dd, ^{4}J = 1.8 Hz, ^{4}J = 0.9 Hz, 1H, Ar-H3), 5.89 (ddt, ^{3}J = 17.0 Hz, ^{3}J = 10.3 Hz, ^{3}J = 6.7 Hz, 1H, -CH=), 5.17 (ddt, ^{3}J = 10.3 Hz, ^{2}J = 1.7 Hz, ^{4}J = 1.7 Hz, 1H, =$CH_{2,\,trans}$), 5.08 (ddt, ^{3}J = 10.3 Hz, ^{2}J = 2.2 Hz, ^{4}J = 1.3 Hz, 1H, =$CH_{2,\,cis}$), 4.29 (t, ^{3}J = 6.7 Hz, 2H, -OCH_2-), 2.49 (dtdd, ^{3}J = 6.8 Hz, ^{3}J= 6.8 Hz, ^{4}J = 1.4 Hz, ^{4}J = 1.4 Hz, 2H, -CH_2-), 2.39 (s_{br}, 3H, -CH_3), 2.27 (s, 3H, -OAc). ^{13}C NMR (101 MHz,

acetone-d_6): δ = 169.63 (-(**C**=O)-CH_3), 164.85 (Ar-(**C**=O)), 151.81 (C2), 145.96 (C4), 135.28 (C3′), 132.17 (C6), 127.44 (C5), 125.26 (C3), 121.63 (C1), 117.54 (C4′), 64.54 (C1′), 33.80 (C2′), 21.25 (-CH_3), 21.04 (-OAc). HR-ESI-MS (*m*/*z*): 271.0939 ($[M+Na]^+$, calcd for $C_{14}H_{16}O_4Na$: 271.0941), 392.1378 ($[3M+H+K]^{2+}$, calcd for $C_{42}H_{49}O_{12}K$: 392.1422).

But-3-en-1-yl 2-acetoxy-5-methylbenzoate (5-M-ASA-Butene)

Colorless oil; yield: 54%. ^{1}H NMR (400 MHz, acetone-d_6): δ = 7.79 (d, ^4J = 2.2 Hz, 1H, Ar-H6), 7.44 (dd, ^3J = 8.3 Hz, ^4J = 2.3 Hz, 1H, Ar-H4), 7.05 (d, ^3J = 8.2 Hz, 1H, Ar-H3), 5.90 (ddt, ^3J = 17.0 Hz, ^3J = 10.3 Hz, ^3J = 6.7 Hz, 1H, -CH=), 5.17 (ddt, ^3J = 17.2 Hz, ^2J = 1.7 Hz, ^4J = 1.7 Hz, 1H, =$CH_{2,\,trans}$), 5.08 (ddt, ^3J = 10.3 Hz, ^2J = 1.4 Hz, ^4J = 1.4 Hz, 1H, =$CH_{2,\,cis}$), 4.30 (t, ^3J = 6.8 Hz, 2H, -OCH_2-), 2.50 (dtdd, ^3J = 6.7 Hz, ^3J = 6.7 Hz, ^4J = 1.4 Hz, ^4J = 1.4 Hz, 2H, -CH_2-), 2.37 (s_{br}, 3H, -CH_3), 2.26 (s, 3H, -OAc). ^{13}C NMR (101 MHz, acetone-d_6): δ = 169.75 (-(**C**=O)-CH_3), 165.08 (Ar-(**C**=O)), 149.50 (C2), 136.53 (C5), 135.24 (C3′), 135.13 (C4), 132.39 (C6), 124.59 (C3), 124.22 (C1), 117.58 (C4′), 64.70 (C1′), 33.80 (C2′), 21.01 (-OAc), 20.66 (-CH_3). HR-ESI-MS (*m*/*z*): 271.0939 ($[M+Na]^+$, calcd for $C_{14}H_{16}O_4Na$: 271.0941), 392.1377 ($[3M+H+K]^{2+}$, calcd for $C_{42}H_{49}O_{12}K$: 392.1422), 519.1984 ($[2M+Na]^+$, calcd for $C_{28}H_{32}O_8Na$: 519.1989).

But-3-en-1-yl 2-acetoxy-6-methylbenzoate (6-M-ASA-Butene)

Colorless oil; yield 68%: ^{1}H NMR (400 MHz, acetone-d_6): δ = 7.38 (dd, ^3J = 7.9 Hz, ^3J = 7.9 Hz, 1H, Ar-H4), 7.17 (d, ^3J = 7.8 Hz, 1H, Ar-H5), 7.02 (d, ^3J = 8.2 Hz, 1H, Ar-H3), 5.88 (ddt, ^3J = 17.1 Hz, ^3J = 10.3 Hz, ^3J = 6.7 Hz, 1H, -CH=), 5.17 (ddt, ^3J = 17.2 Hz, ^2J = 1.7 Hz, ^4J = 1.7 Hz, 1H, =$CH_{2,\,trans}$), 5.08 (ddt, ^3J = 10.3 Hz, ^2J = 1.4 Hz, ^4J = 1.4 Hz, 1H, =$CH_{2,\,cis}$), 4.35 (t, ^3J = 6.7 Hz, 2H, -OCH_2-), 2.49 (dtdd, ^3J = 6.7 Hz, ^3J = 6.7 Hz, ^4J = 1.4 Hz, ^4J = 1.4 Hz, 2H, -CH_2-), 2.36 (s_{br}, 3H, -CH_3), 2.22 (s, 3H, -OAc). ^{13}C NMR (101 MHz, acetone-d_6): δ = 169.18 (-(**C**=O)-CH_3), 166.90 (Ar-(**C**=O)), 149.35 (C2), 138.34 (C6), 135.23 (C3′), 131.26 (C4), 128.58 (C5), 127.98 (C1), 121.53 (C3), 117.62 (C4′), 65.02 (C1′), 33.78 (C2′), 20.83 (-OAc), 19.94 (-CH_3). HR-ESI-MS (*m*/*z*): 271.0939 ($[M+Na]^+$, calcd for $C_{14}H_{16}O_4Na$: 271.0941), 519.1984 ($[2M+Na]^+$, calcd for $C_{28}H_{32}O_8Na$: 519.1989).

Electropherograms of the complexes

The conditions are given in the "Experimental section" of the article.

Supporting Figure S1: Electropherogram of 3-F-Prop-PtCl$_3$

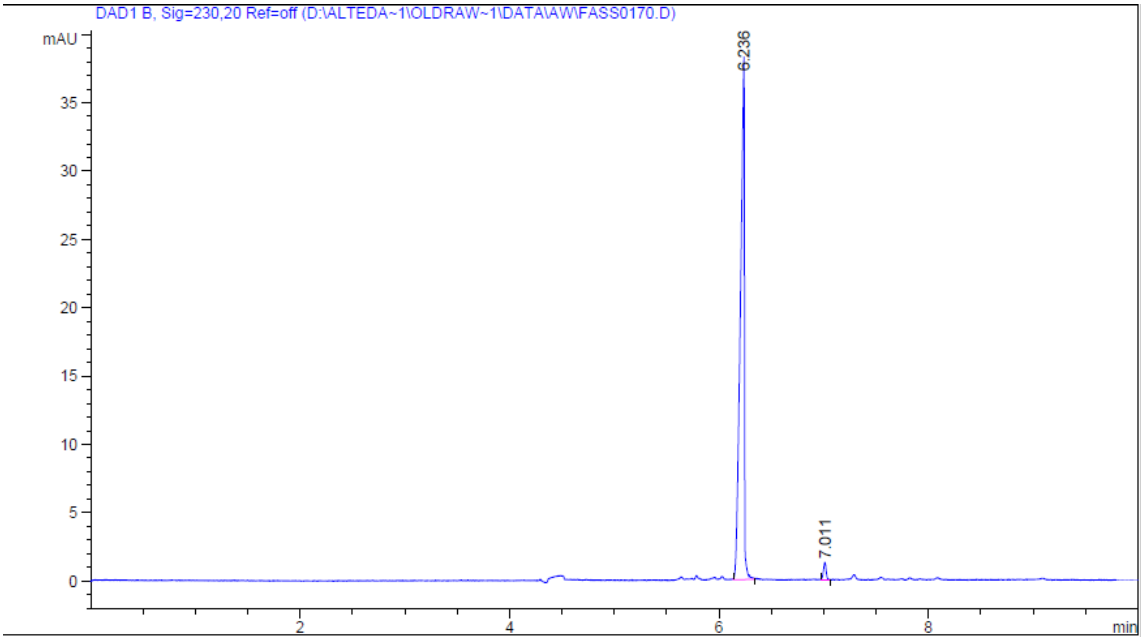

Supporting Figure S2: Electropherogram of 4-F-Prop-PtCl$_3$

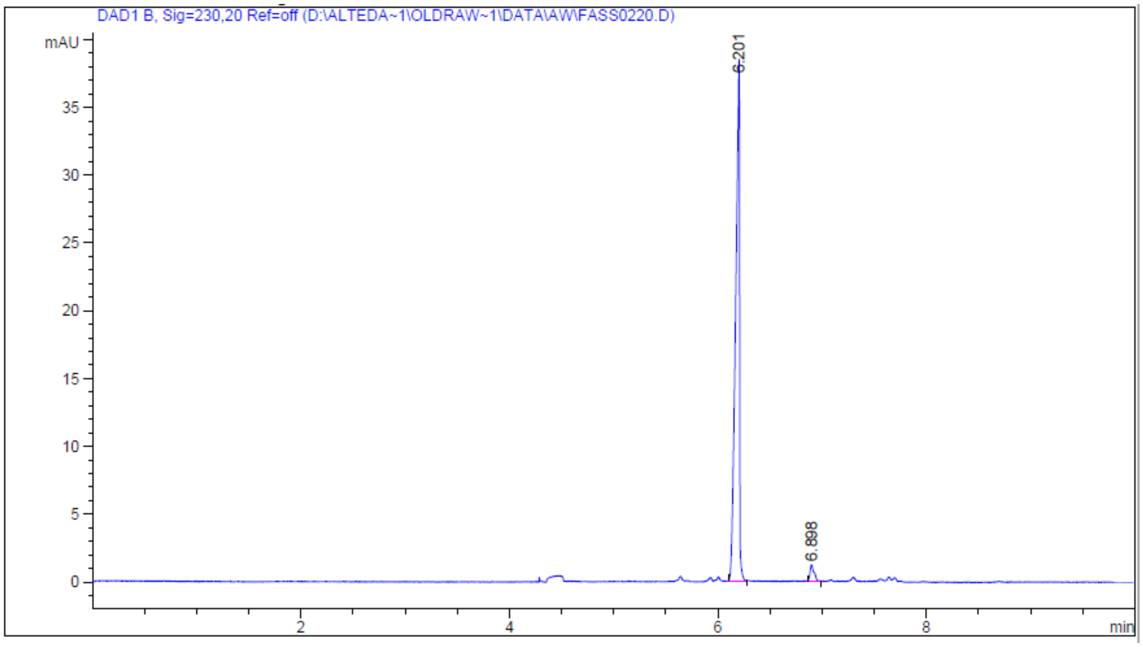

Supporting Figure S3: Electropherogram of 5-F-Prop-PtCl$_3$

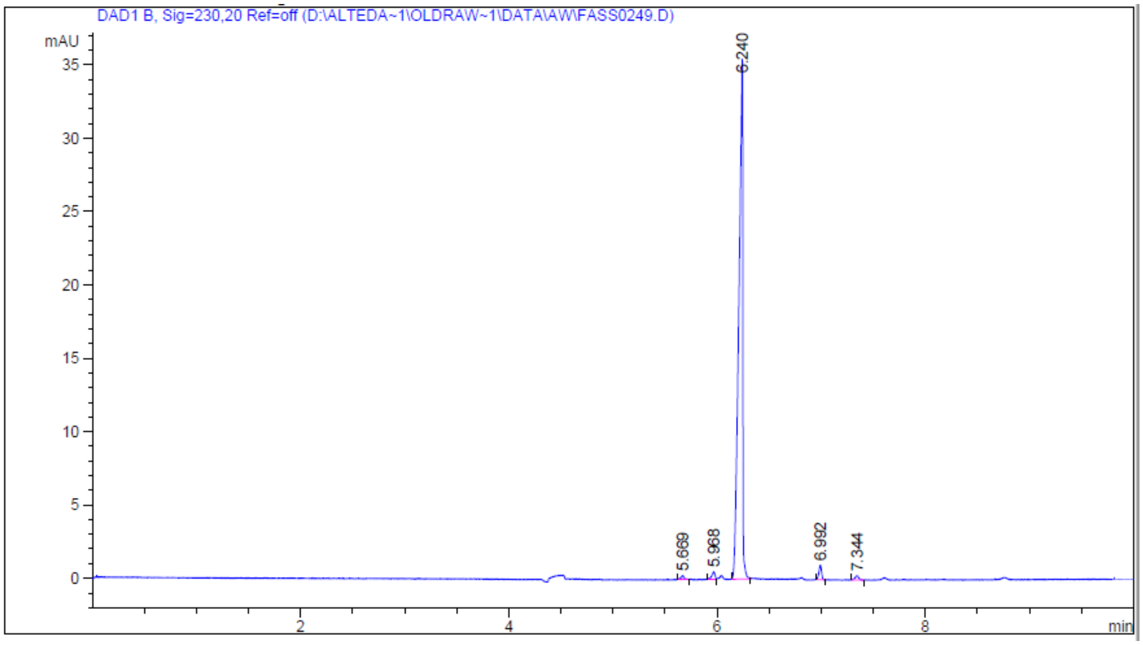

Supporting Figure S4: Electropherogram of 6-F-Prop-PtCl$_3$

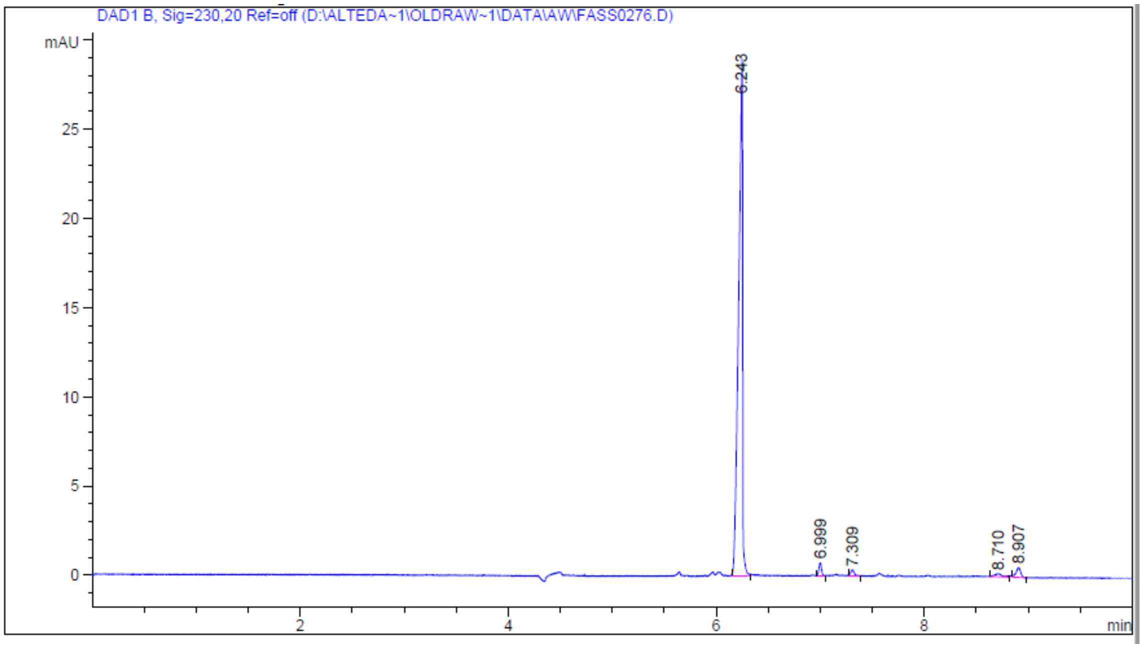

Supporting Figure S5: Electropherogram of 3-F-But-PtCl$_3$

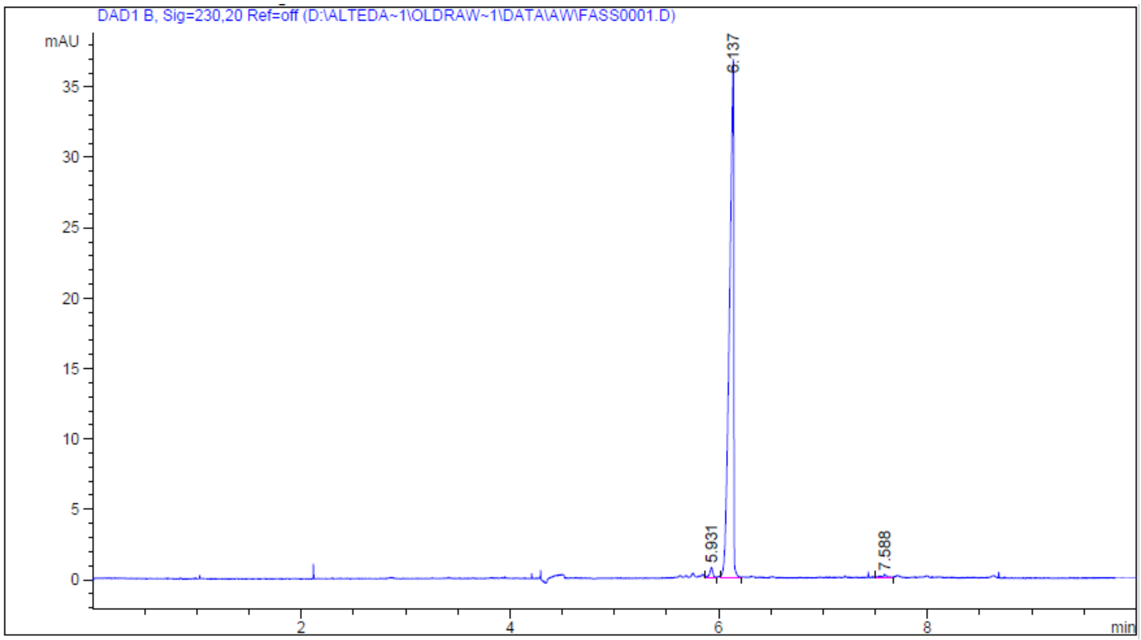

Supporting Figure S6: Electropherogram of 4-F-But-PtCl$_3$

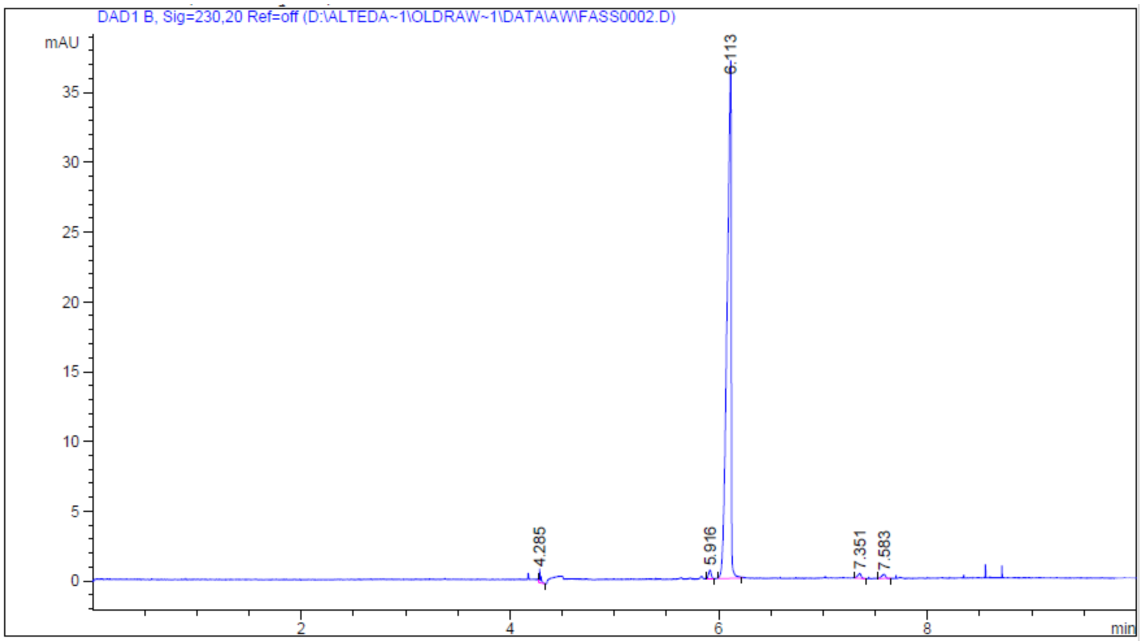

Supporting Figure S7: Electropherogram of 5-F-But-$PtCl_3$

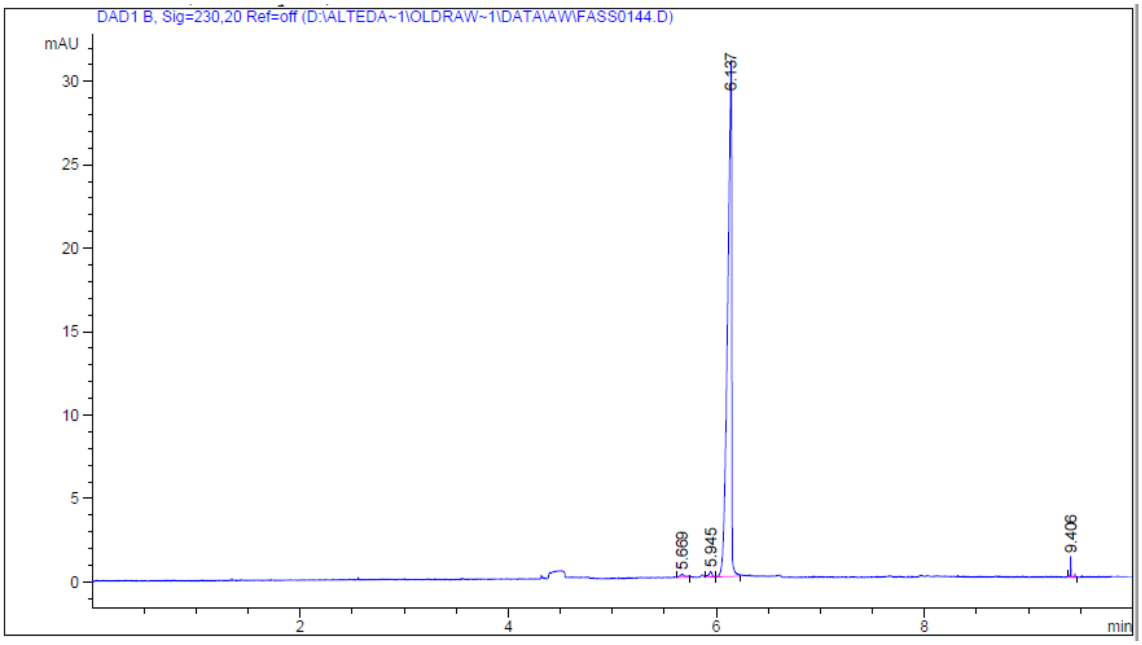

Supporting Figure S8: Electropherogram of 6-F-But-$PtCl_3$

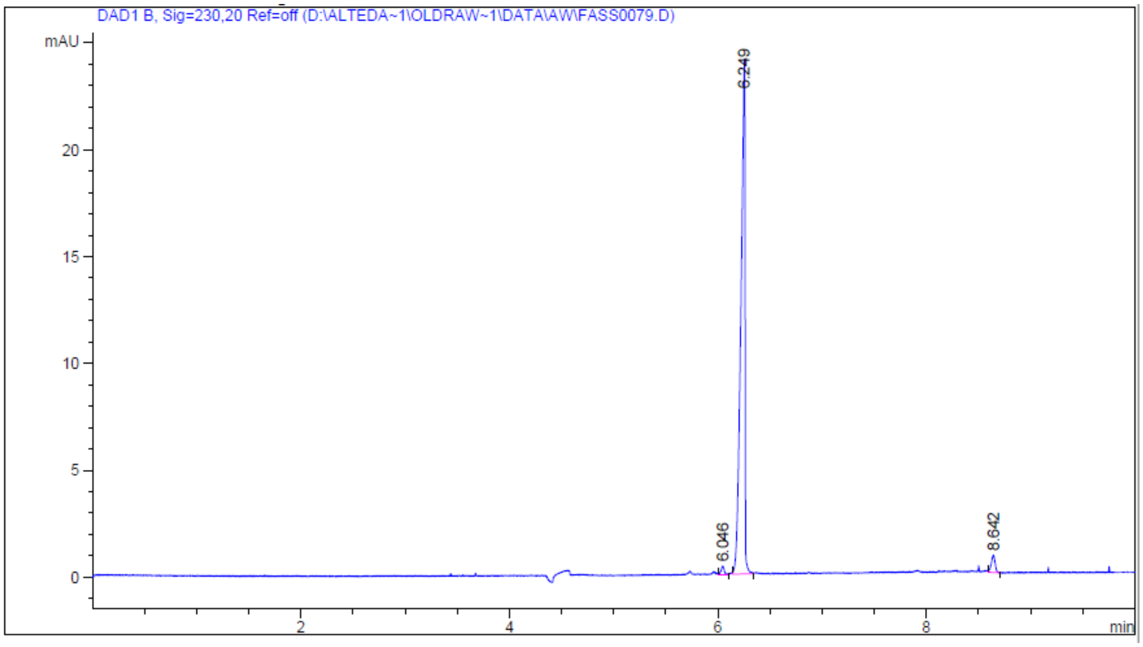

Supporting Figure S9: Electropherogram of 3-Cl-Prop-PtCl₃

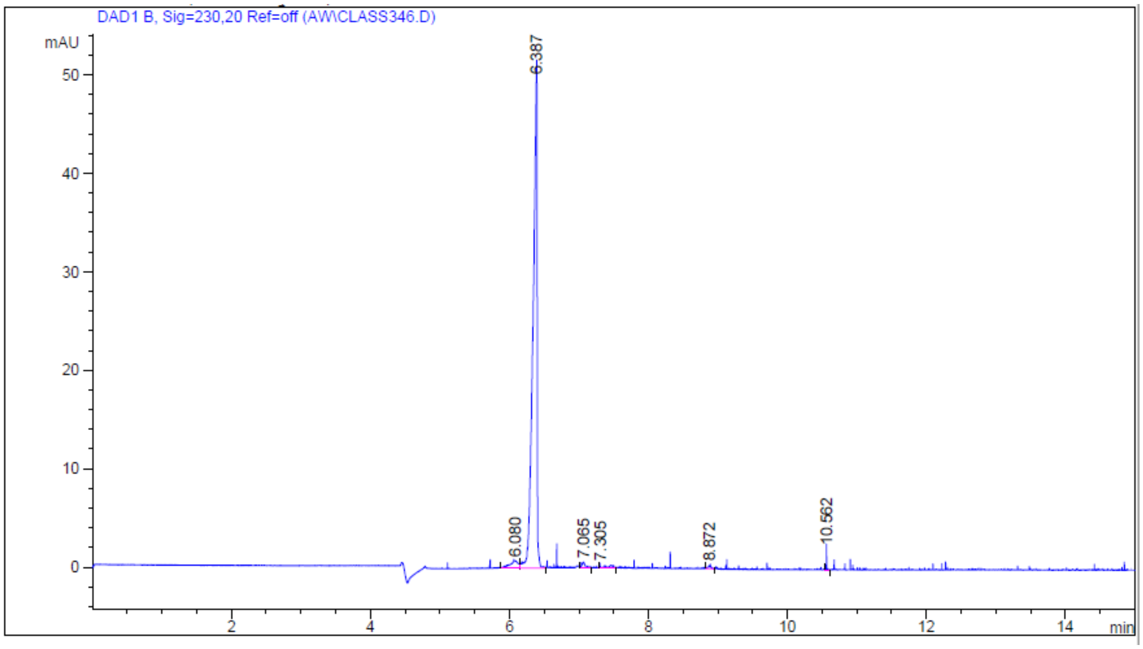

Supporting Figure S10: Electropherogram of 4-Cl-Prop-PtCl₃

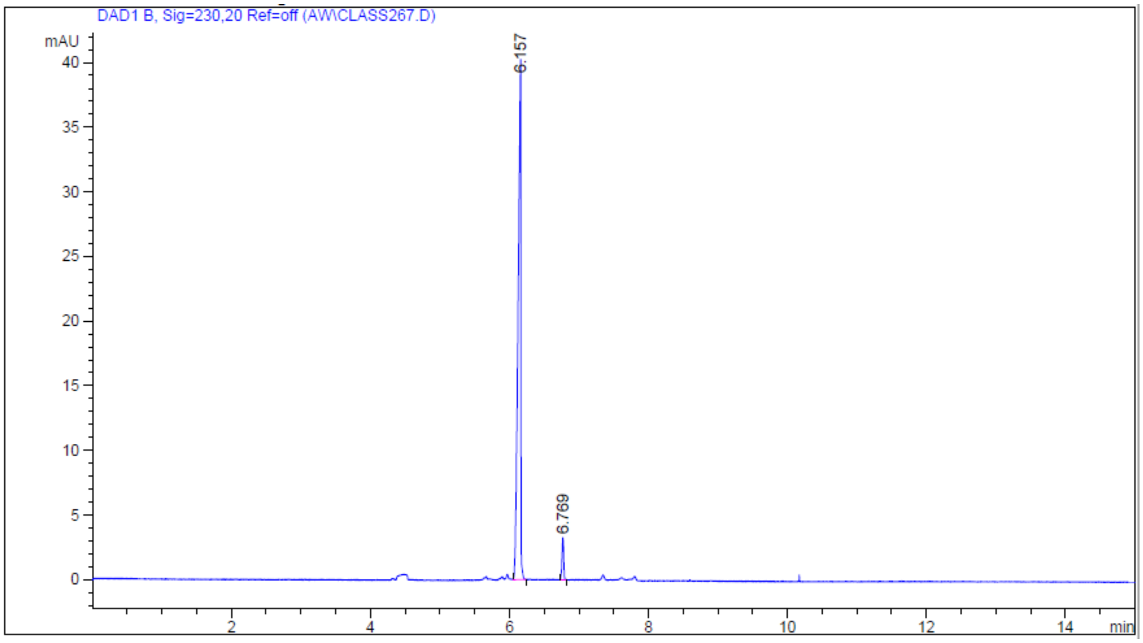

Supporting Figure S11: Electropherogram of 5-Cl-Prop-$PtCl_3$

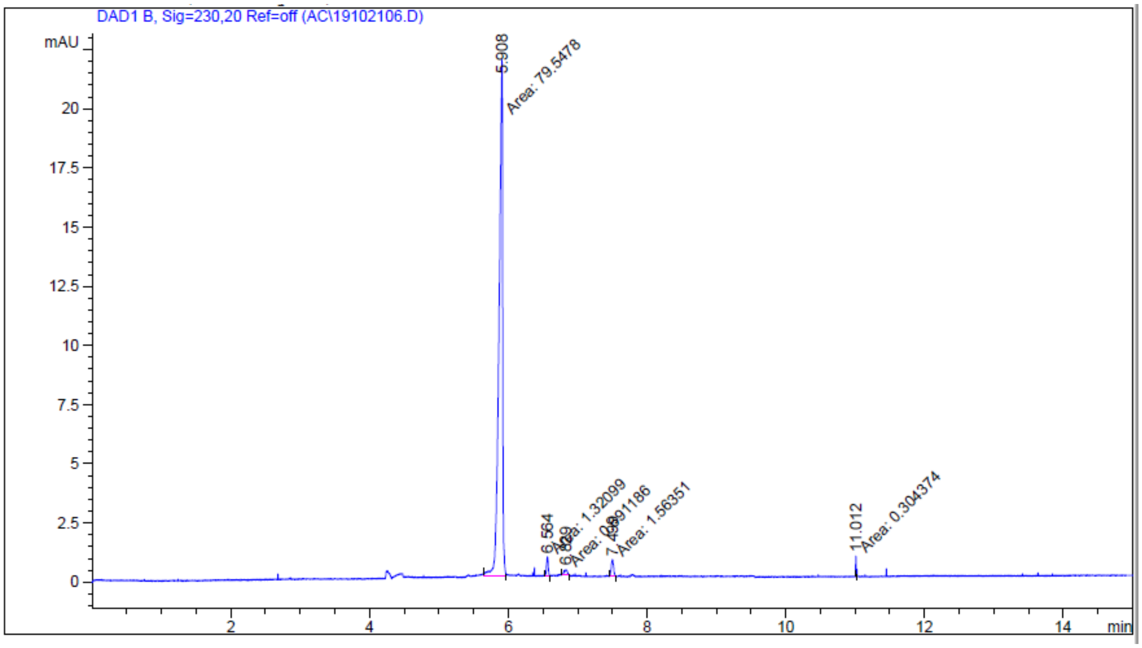

Supporting Figure S12: Electropherogram of 6-Cl-Prop-$PtCl_3$

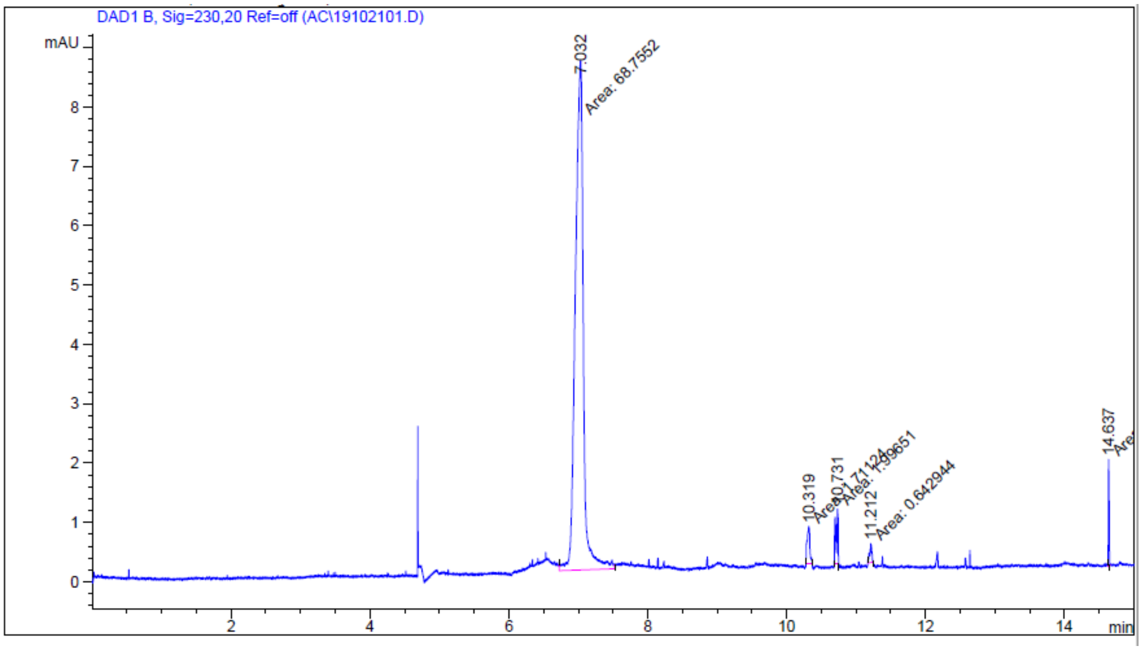

Supporting Figure S13: Electropherogram of 3-Cl-But-$PtCl_3$

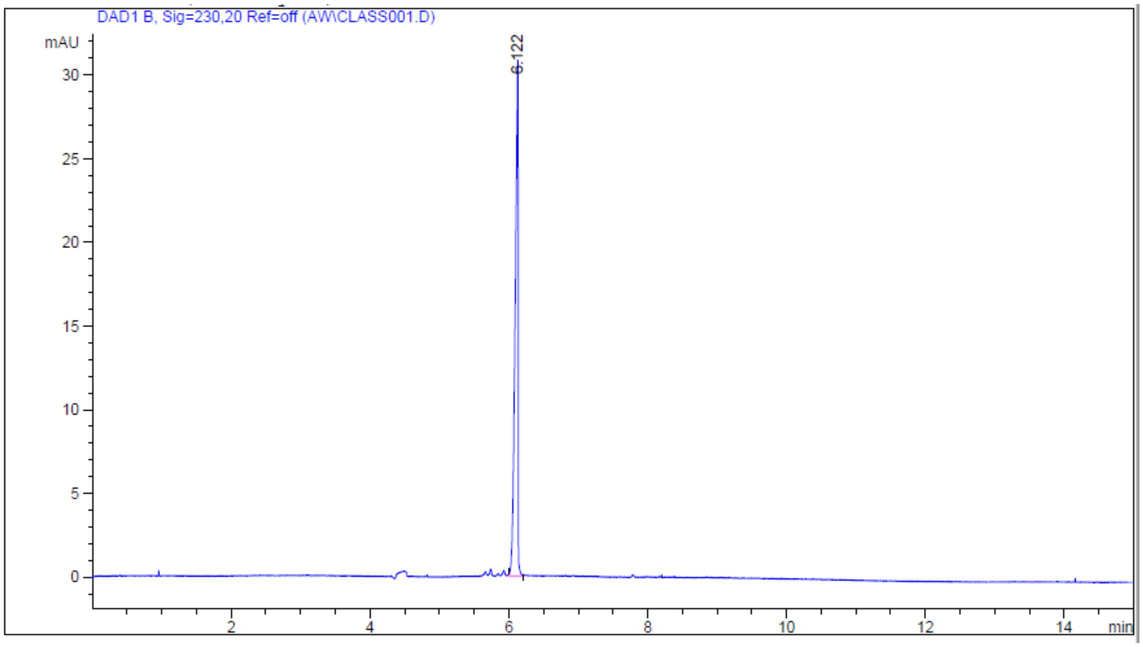

Supporting Figure S14: Electropherogram of 4-Cl-But-$PtCl_3$

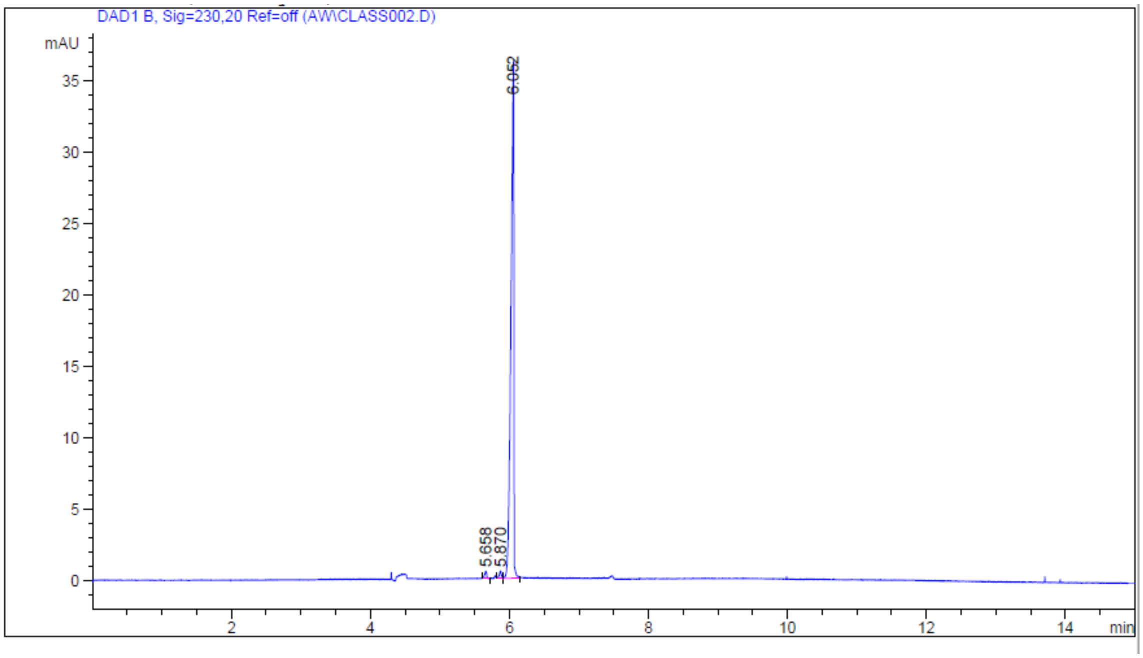

Supporting Figure S15: Electropherogram of 5-Cl-But-$PtCl_3$

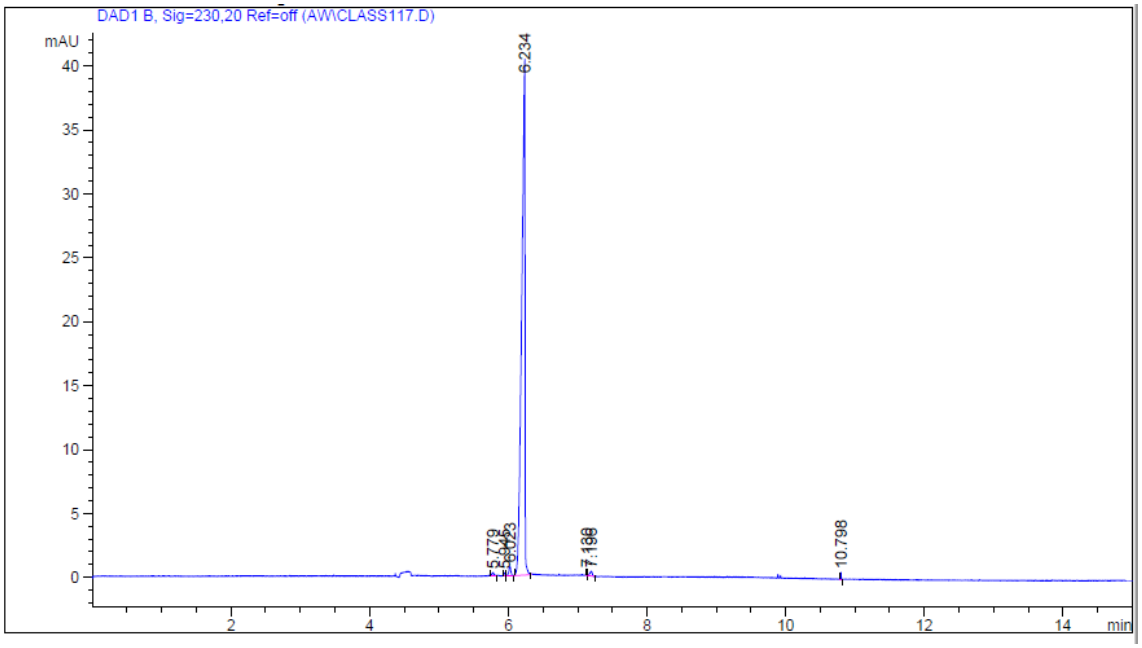

Supporting Figure S16: Electropherogram of 6-Cl-But-$PtCl_3$

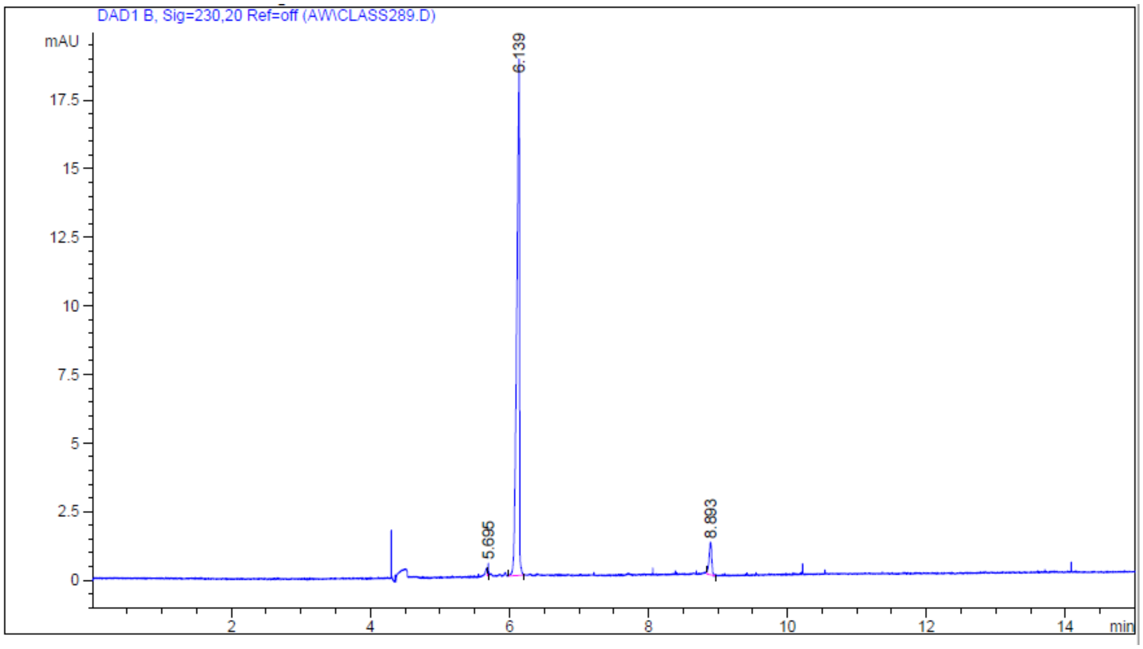

Supporting Figure S17: Electropherogram of 3-CH_3-Prop-$PtCl_3$

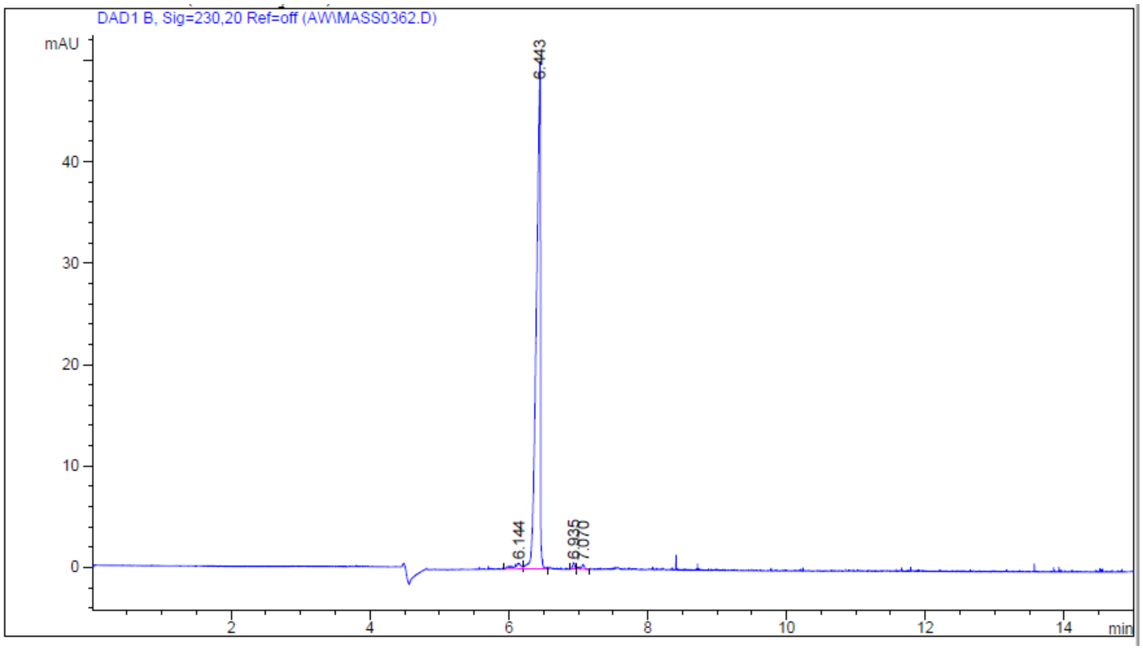

Supporting Figure S18: Electropherogram of 4-CH_3-Prop-$PtCl_3$

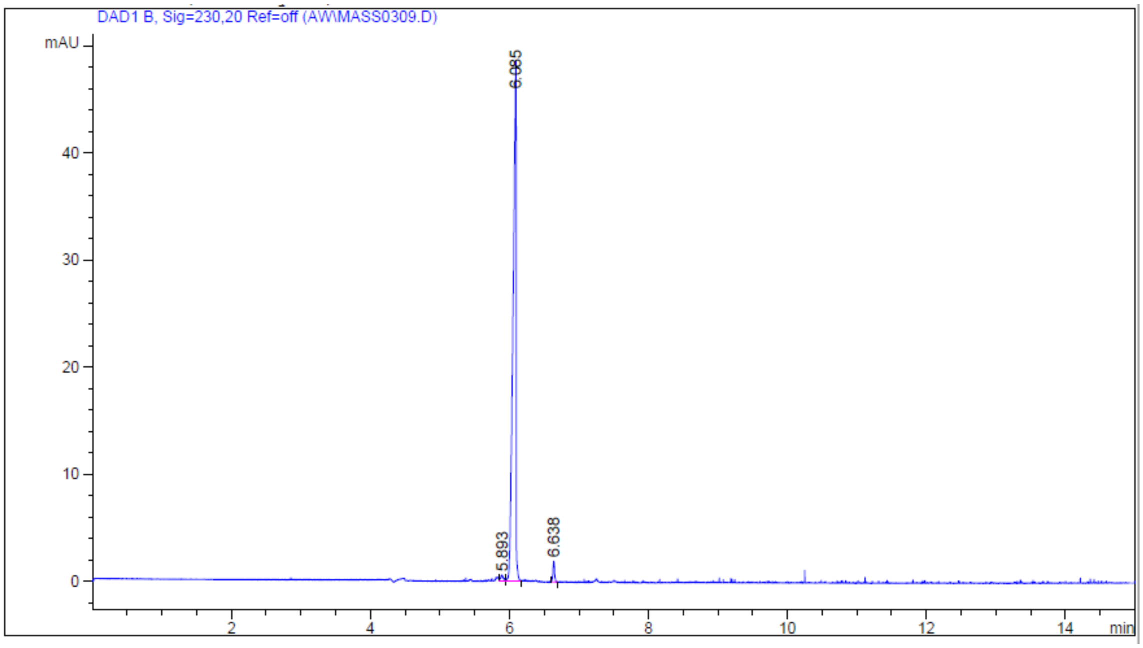

Supporting Figure S19: Electropherogram of 5-CH_3-Prop-$PtCl_3$

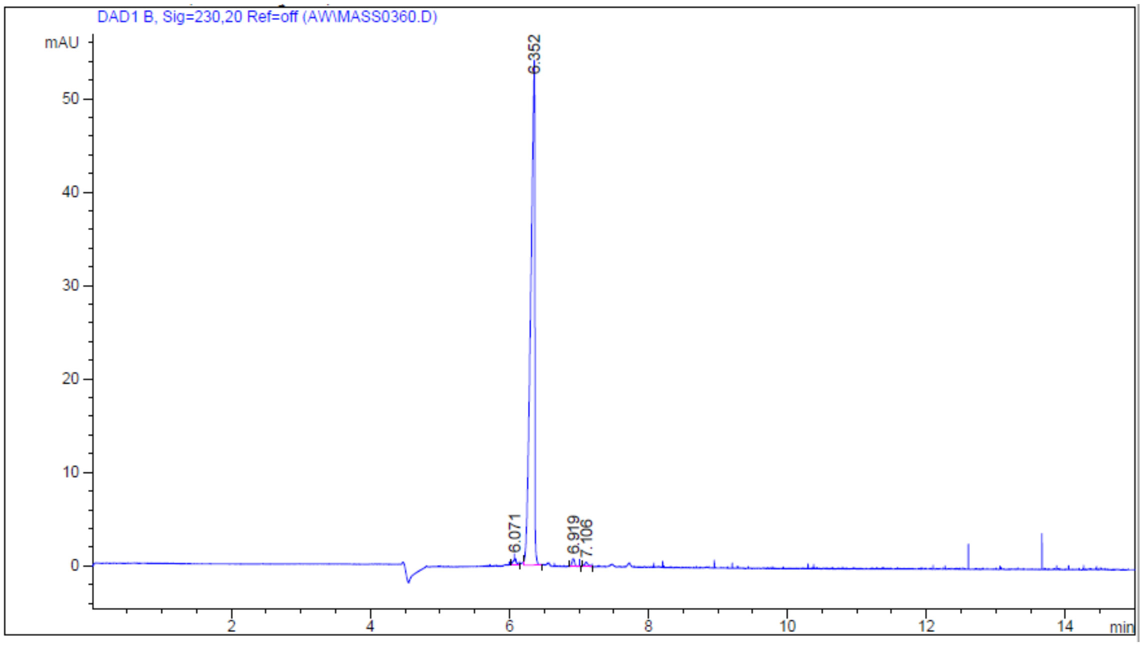

Supporting Figure S20: Electropherogram of 6-CH_3-Prop-$PtCl_3$

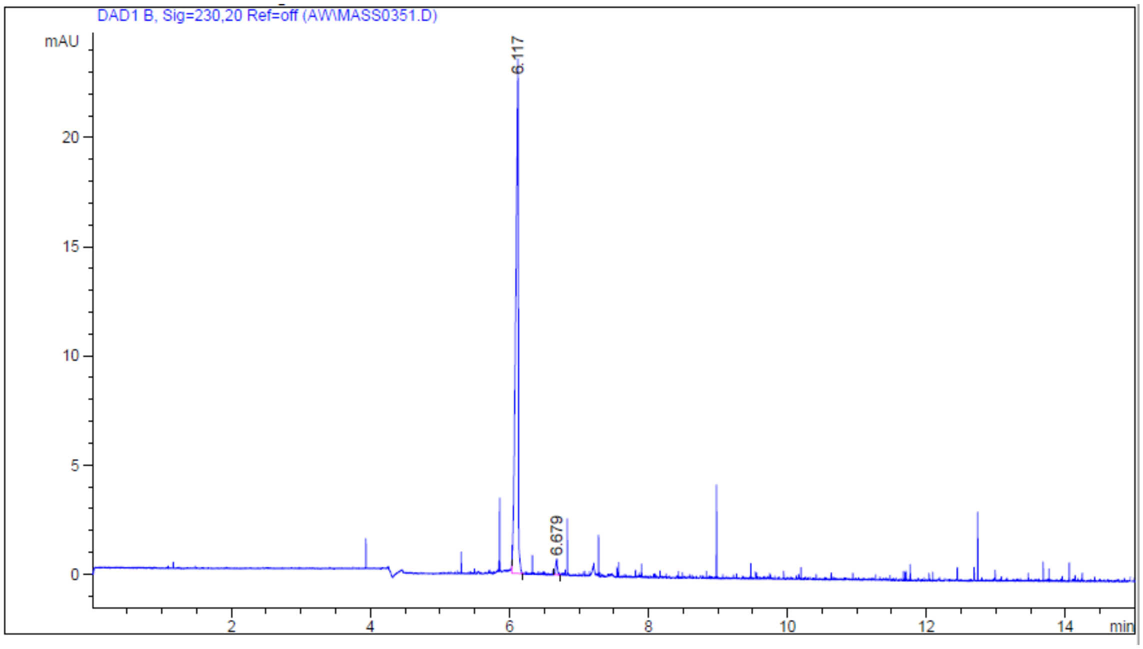

Supporting Figure S21: Electropherogram of 3-CH_3-But-$PtCl_3$

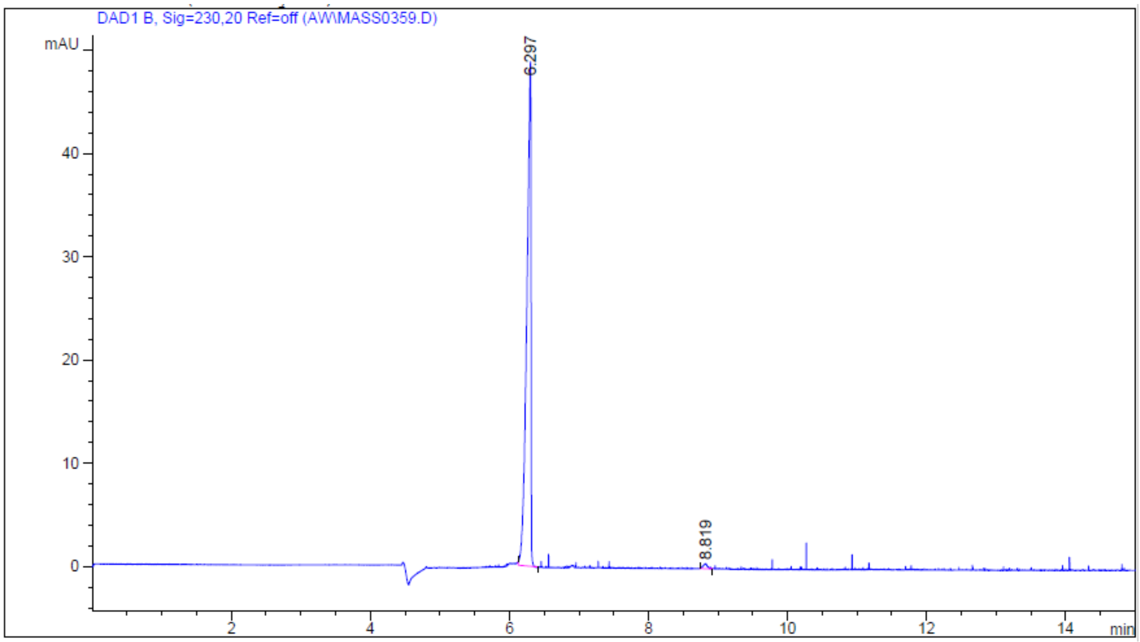

Supporting Figure S22: Electropherogram of 4-CH_3-But-$PtCl_3$

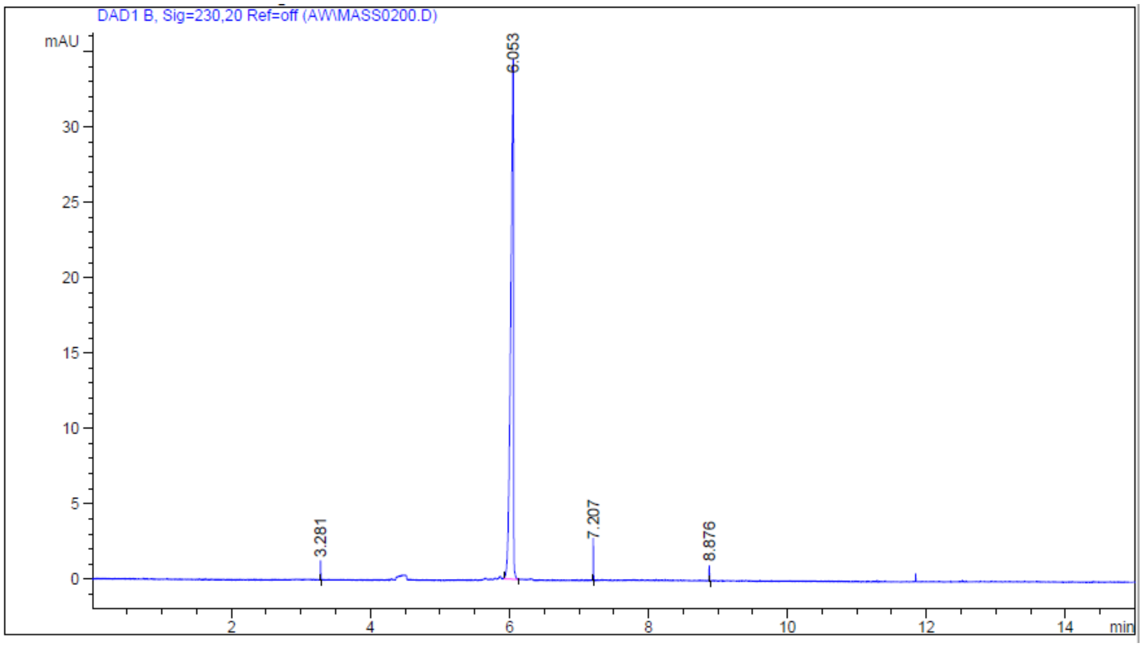

Supporting Figure S23: Electropherogram of 5-CH_3-But-$PtCl_3$

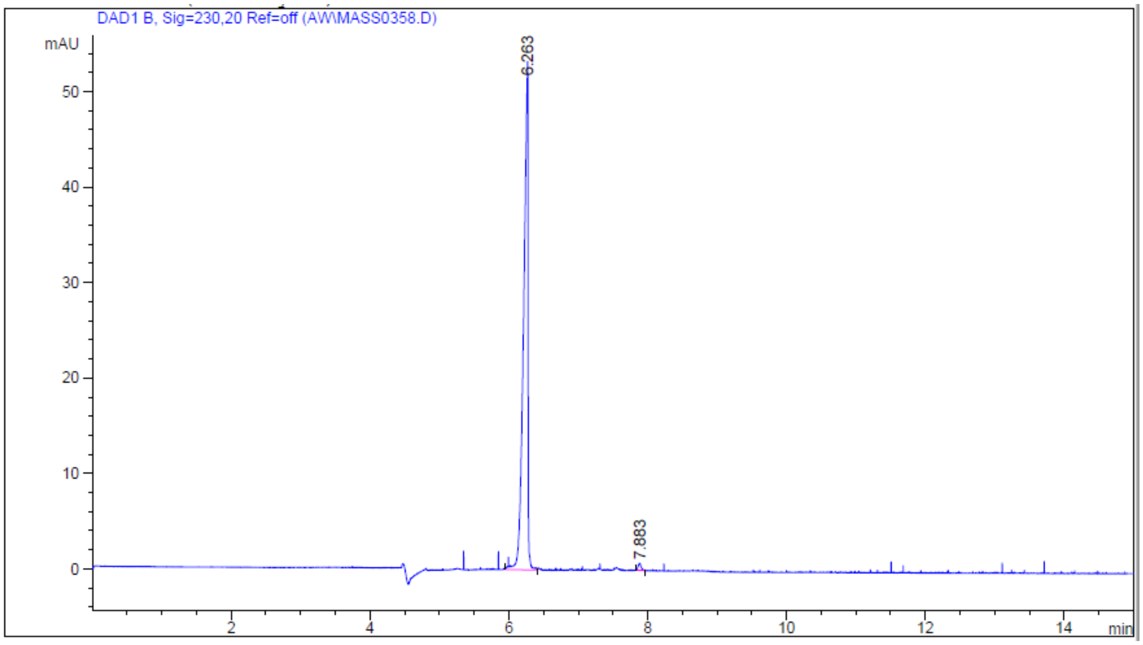

Supporting Figure S24: Electropherogram of 6-CH_3-But-$PtCl_3$

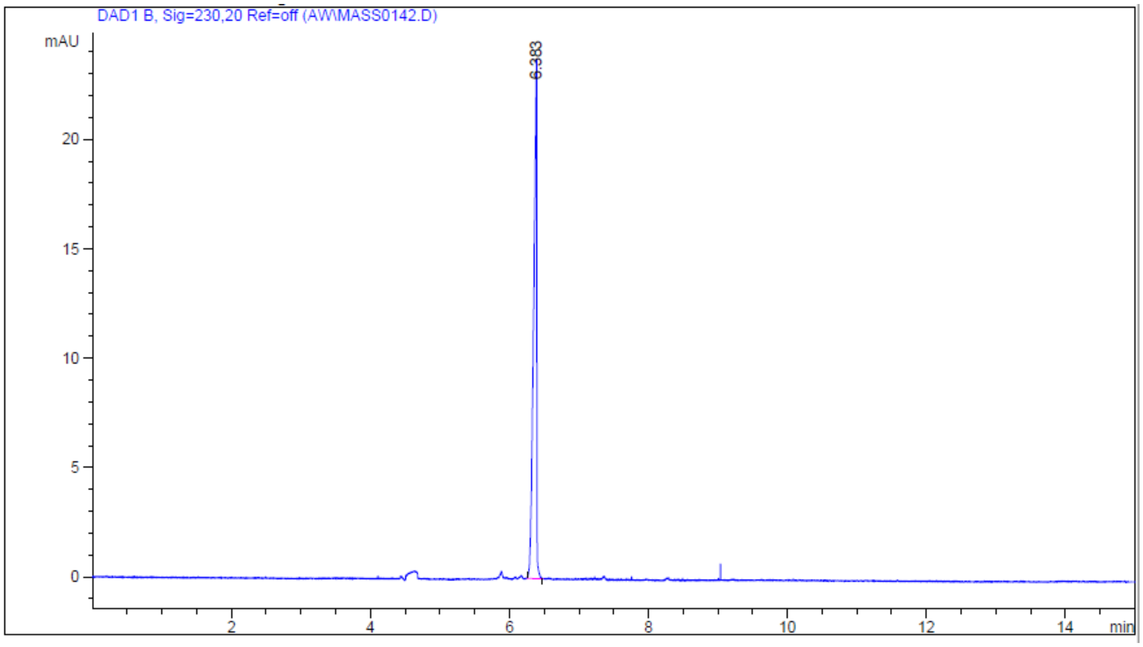

NMR spectra of the ligands and complexes

The conditions are given in the "Experimental section" of the article.

Supporting Figure S25: ¹H and ¹³C NMR spectra of 3-F-ASA-Prop

¹H NMR spectrum of 3-F-ASA-Prop

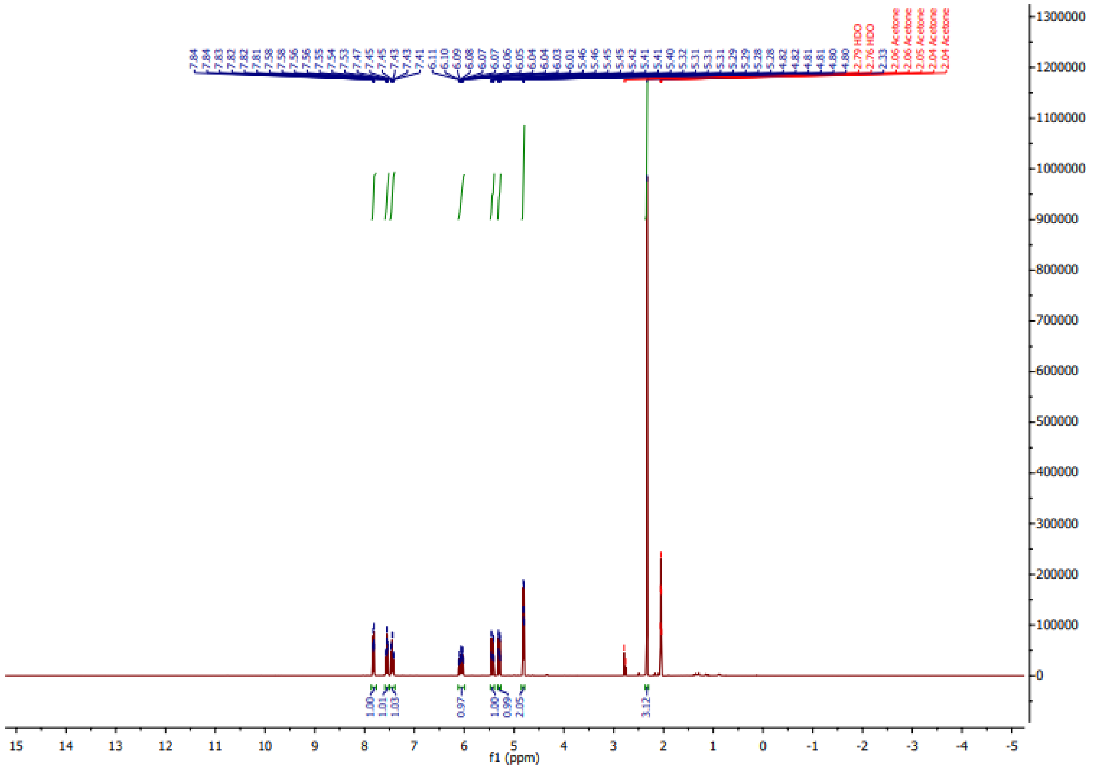

¹³C NMR spectrum of 3-F-ASA-Prop

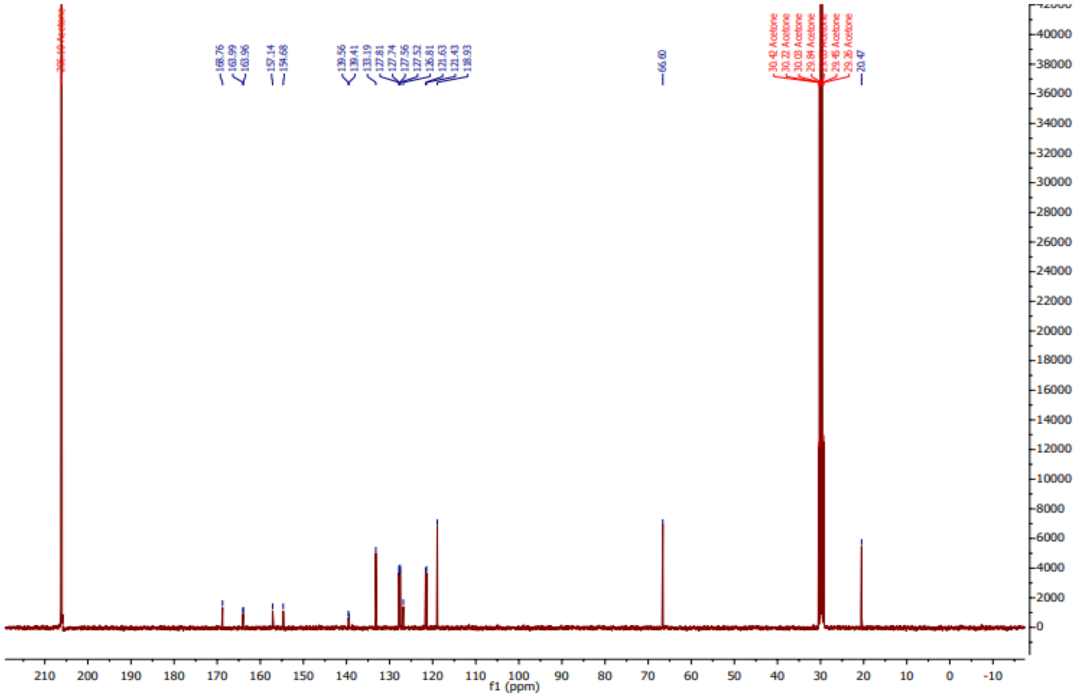

__**Supporting Figure S26: ^{1}H and ^{13}C NMR spectra of 3-F-ASA-But**__

^{1}H NMR spectrum of 3-F-ASA-But

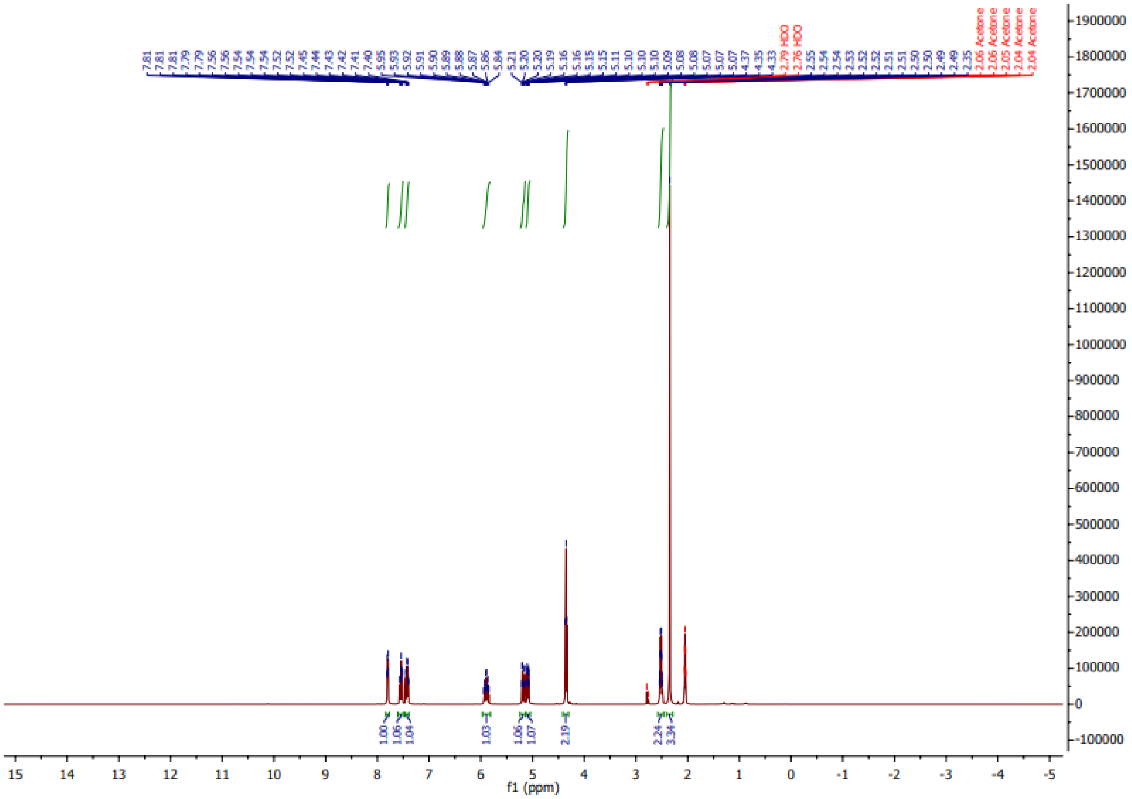

^{13}C NMR spectrum of 3-F-ASA-But

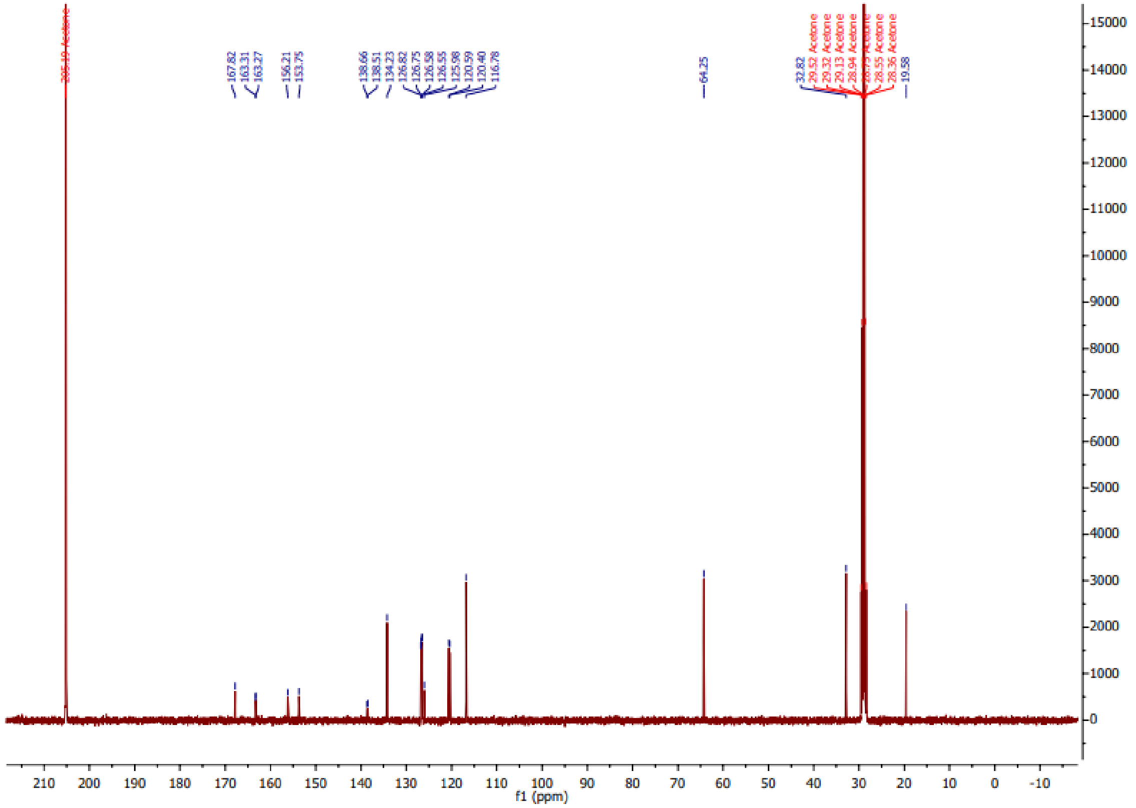

Supporting Figure S27: ^{1}H and ^{13}C NMR spectra of 4-F-ASA-Prop

^{1}H NMR spectrum of 4-F-ASA-Prop

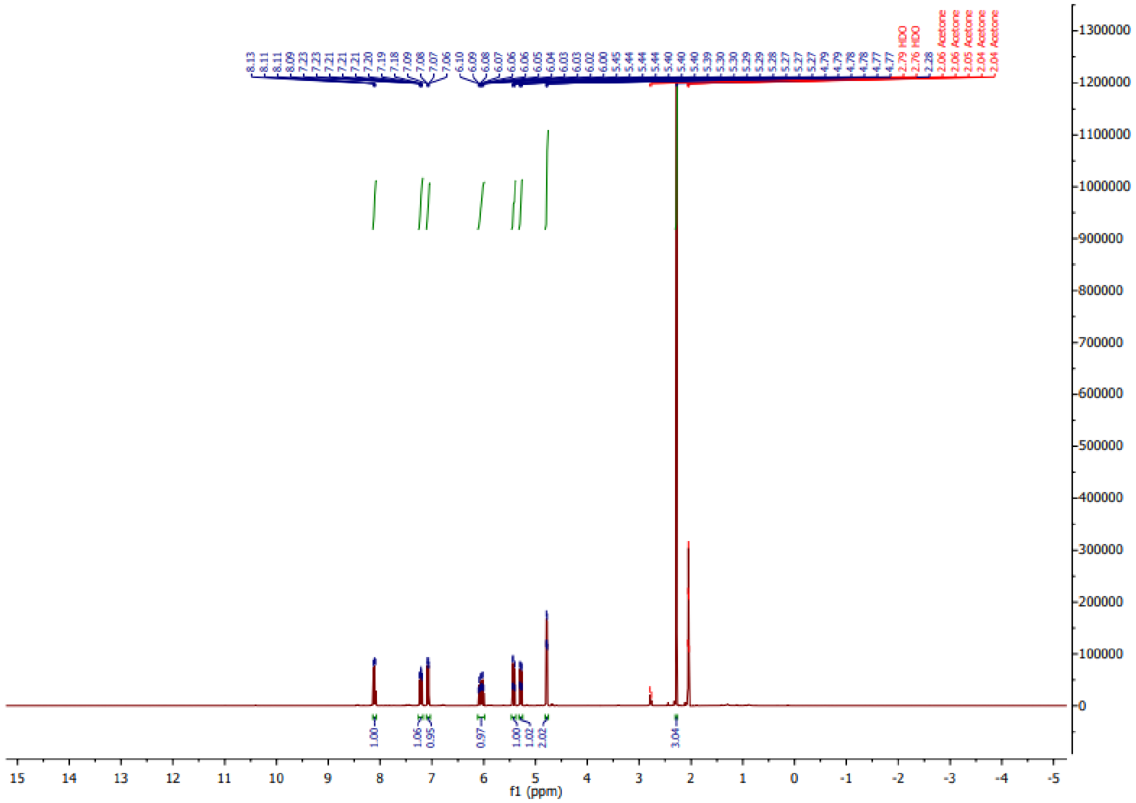

^{13}C NMR spectrum of 4-F-ASA-Prop

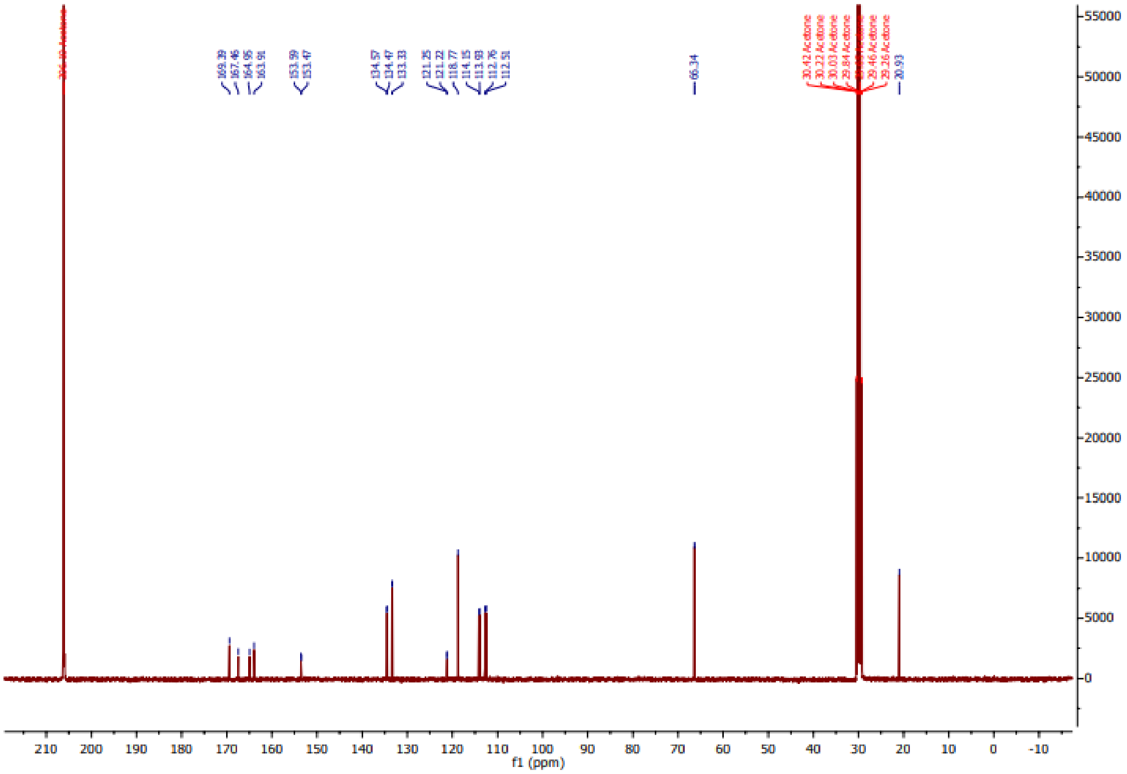

Supporting Figure S28: ^{1}H and ^{13}C NMR spectra of 4-F-ASA-But

^{1}H NMR spectrum of 4-F-ASA-But

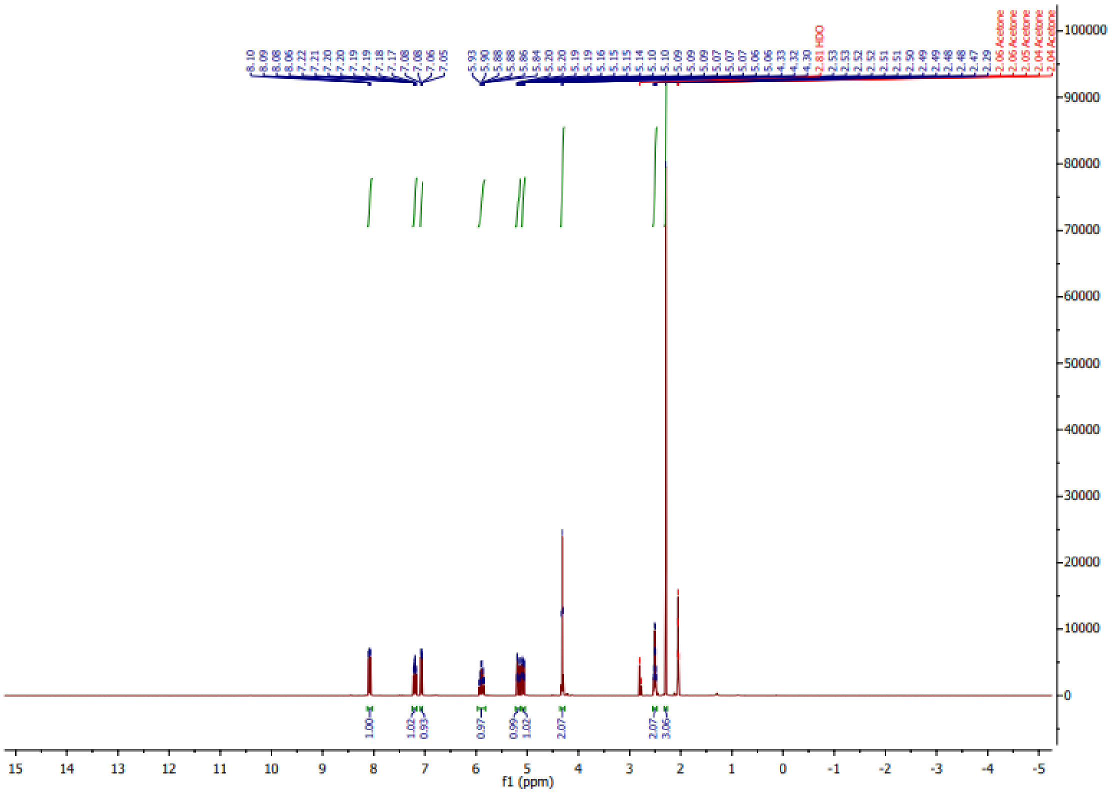

^{13}C NMR spectrum of 4-F-ASA-But

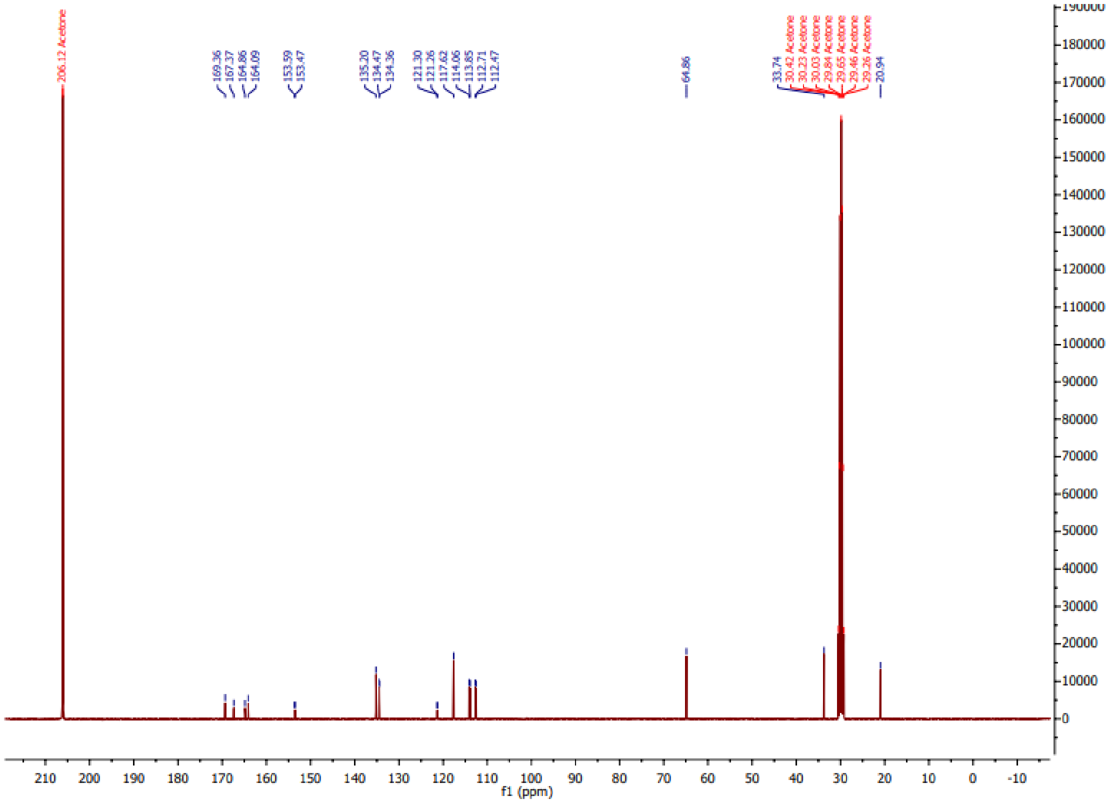

Supporting Figure S29: ^1H and ^{13}C NMR spectra of 5-F-ASA-Prop

^1H NMR spectrum of 5-F-ASA-Prop

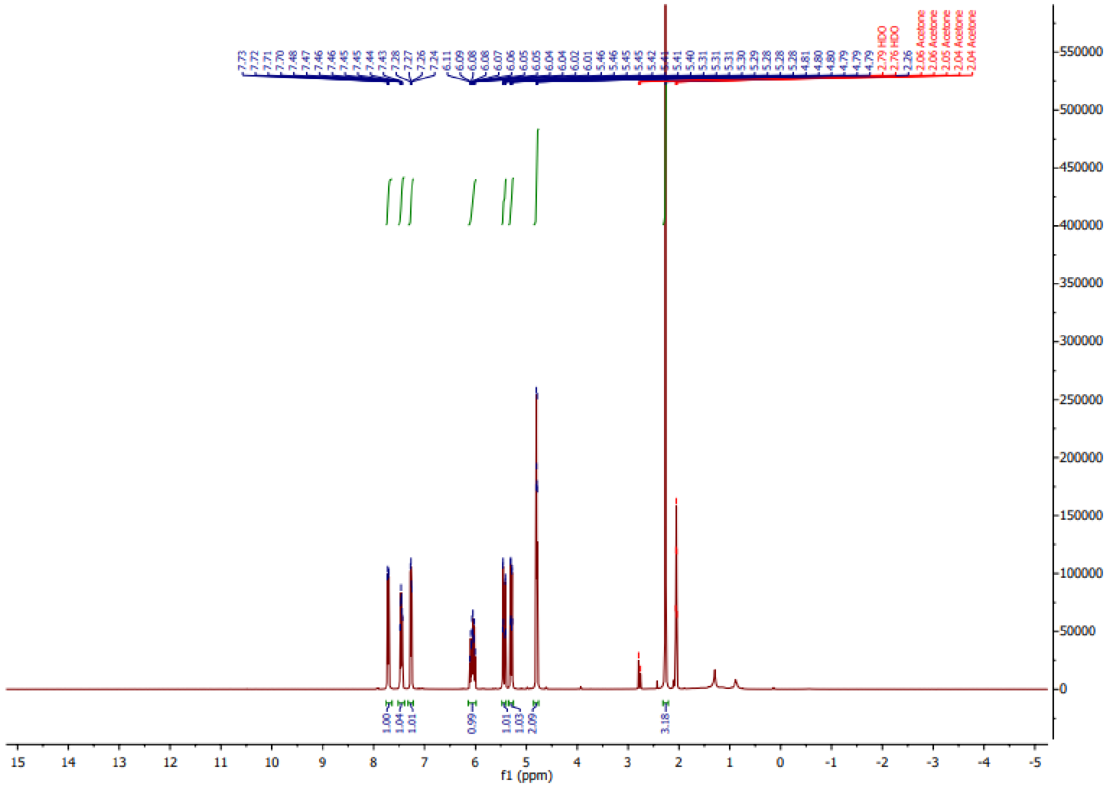

^{13}C NMR spectrum of 5-F-ASA-Prop

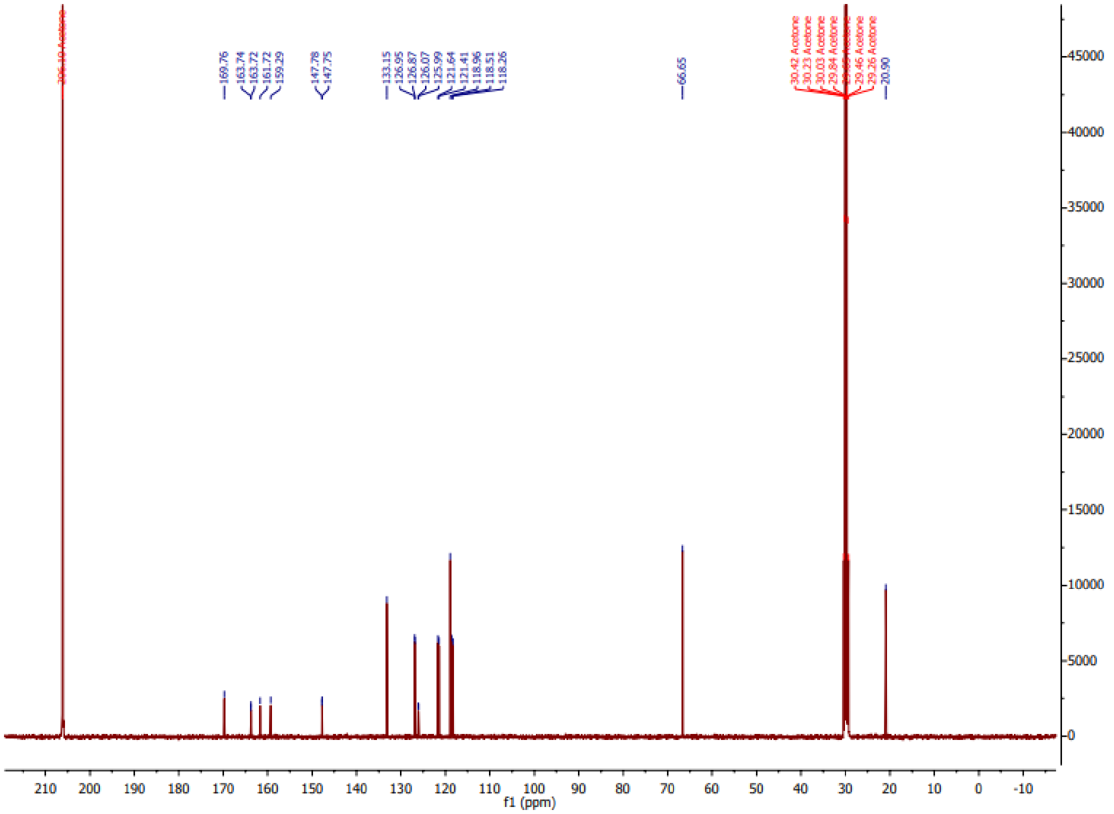

Supporting Figure S30: ^{1}H and ^{13}C NMR spectra of 5-F-ASA-But

^{1}H NMR spectrum of 5-F-ASA-But

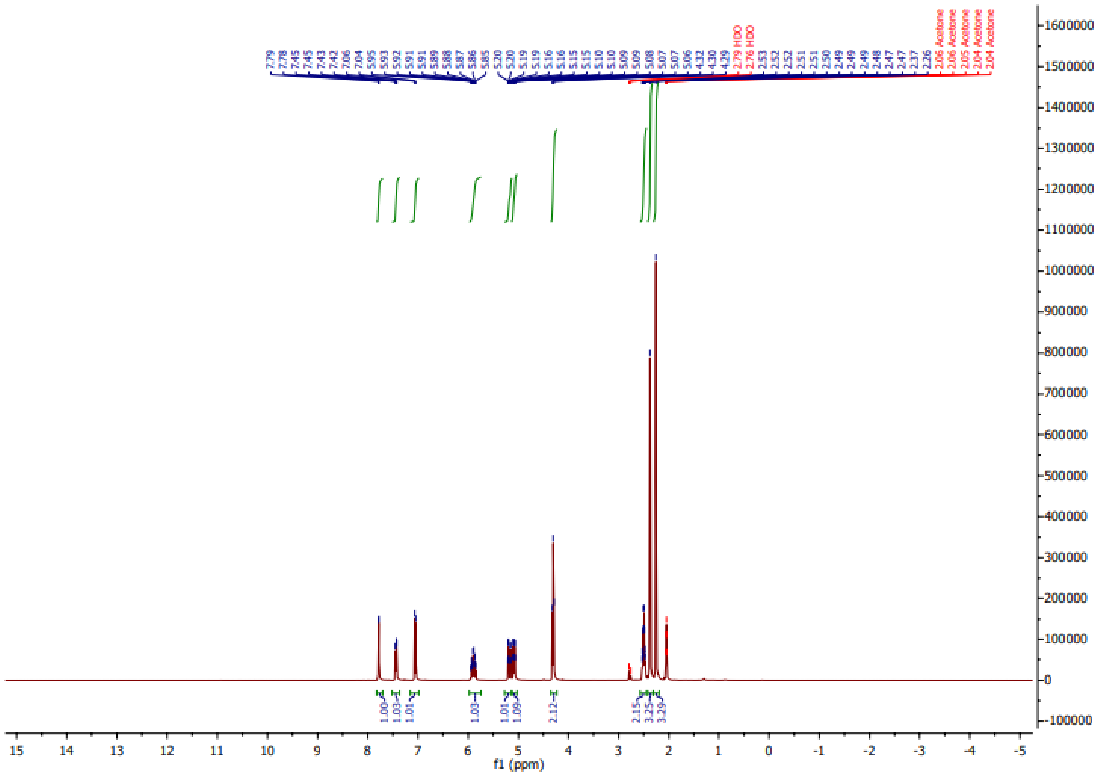

^{13}C NMR spectrum of 5-F-ASA-But

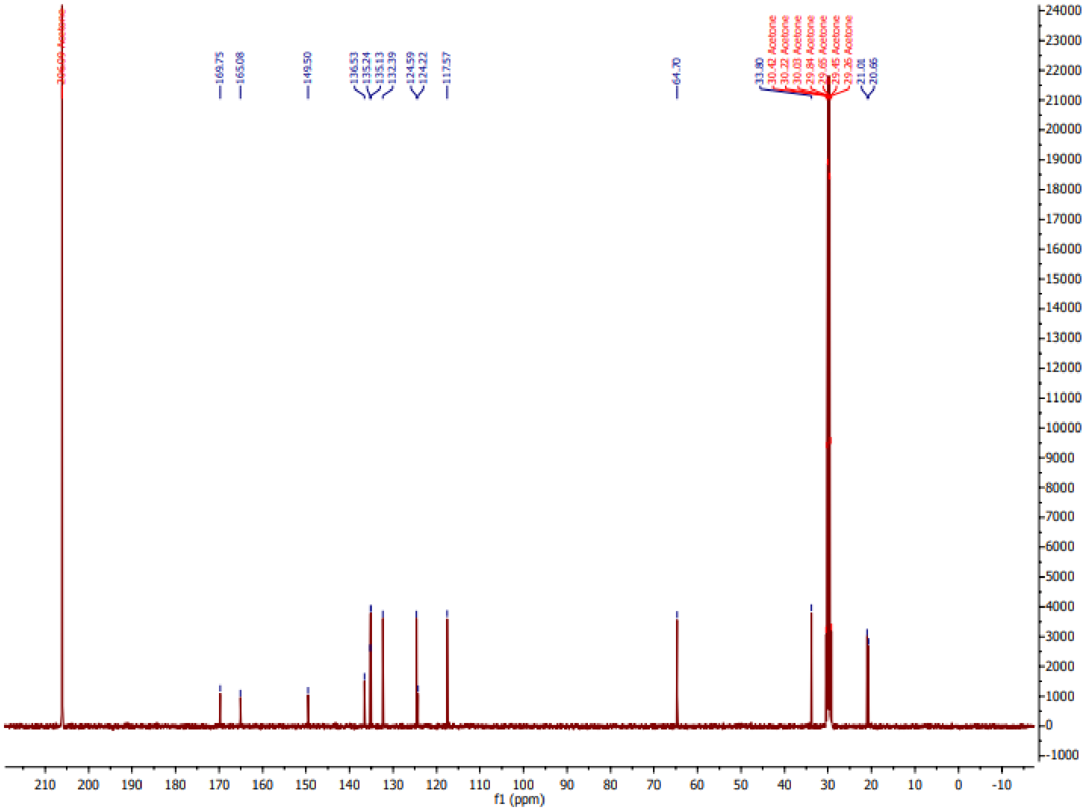

Supporting Figure S31: ^{1}H and ^{13}C NMR spectra of 6-F-ASA-Prop

^{1}H NMR spectrum of 6-F-ASA-Prop

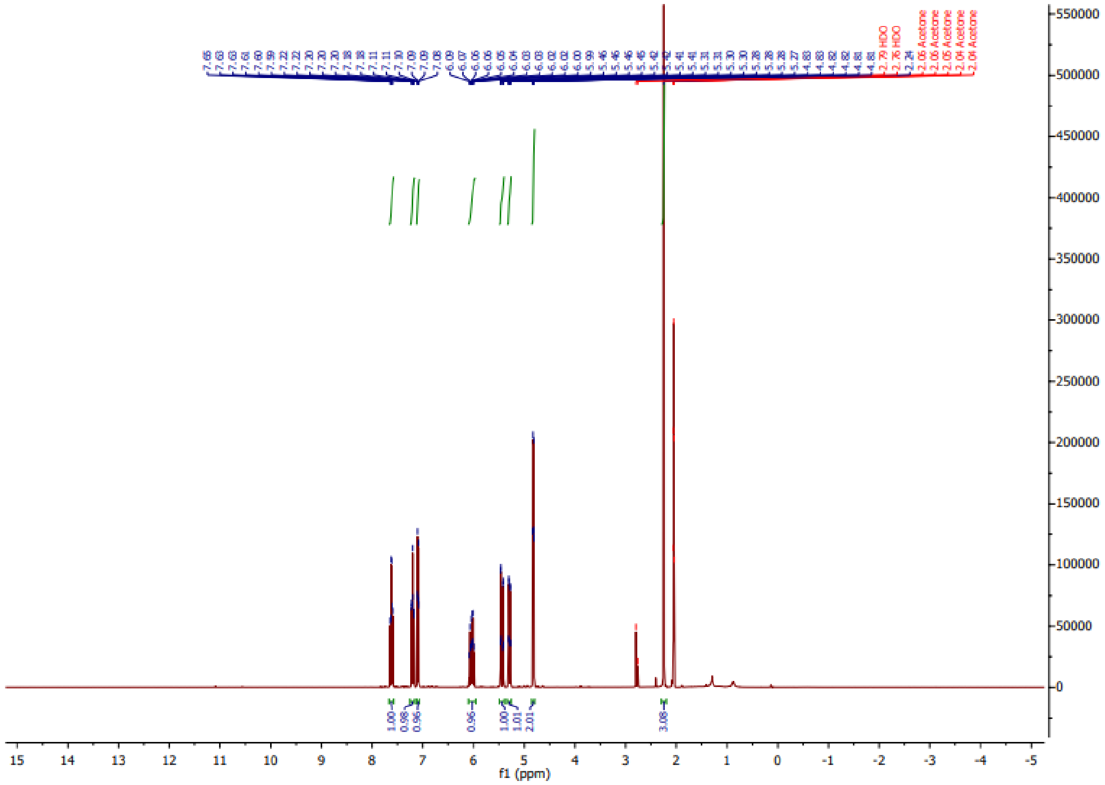

^{13}C NMR spectrum of 6-F-ASA-Prop

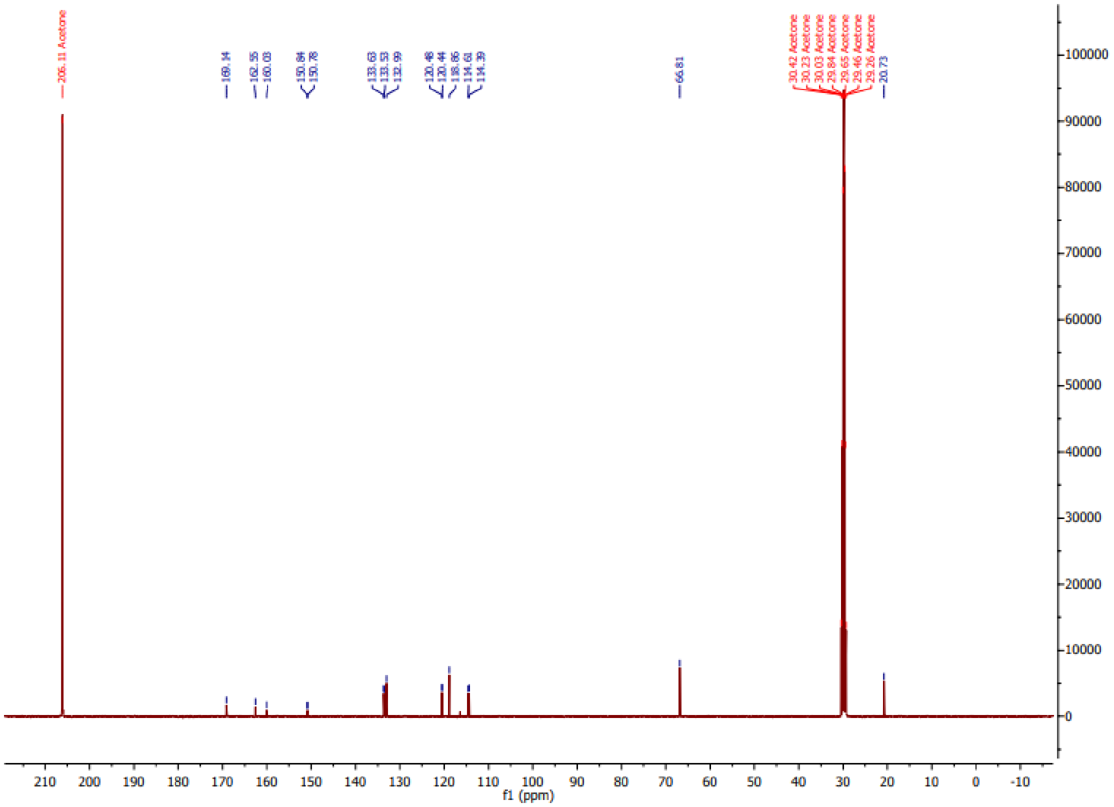

Supporting Figure S32: ^{1}H and ^{13}C NMR spectra of 6-F-ASA-But

^{1}H NMR spectrum of 6-F-ASA-But

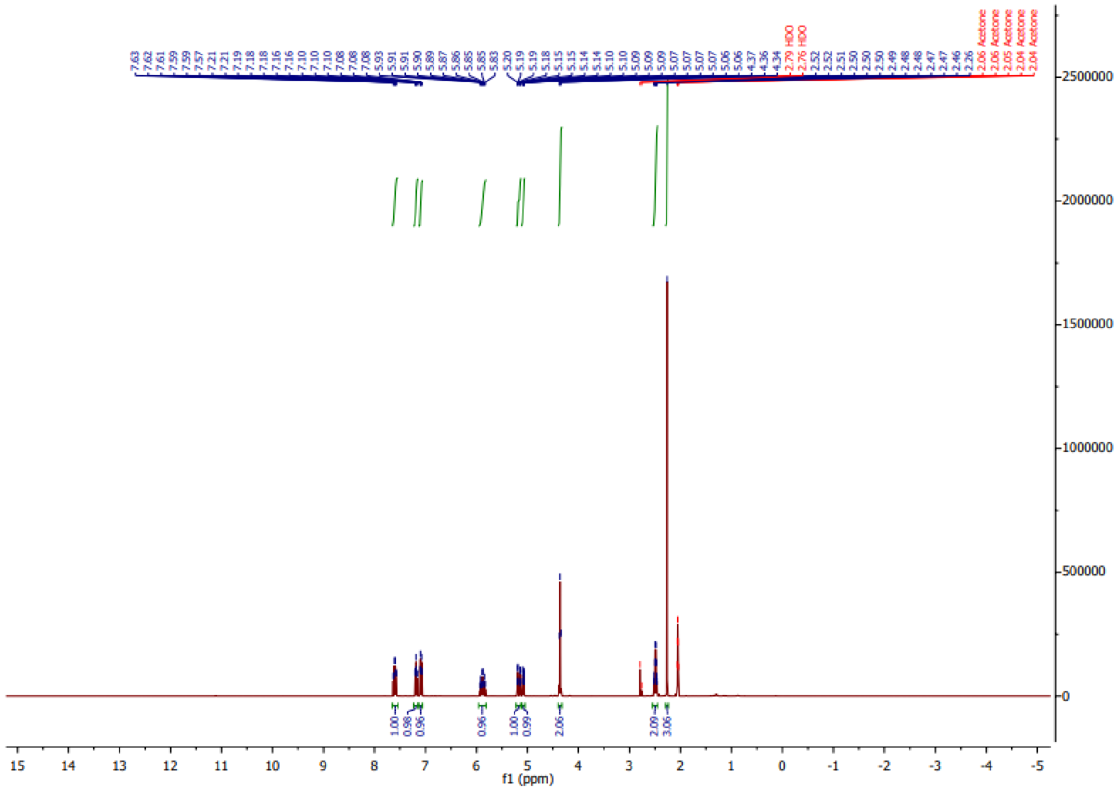

^{13}C NMR spectrum of 6-F-ASA-But

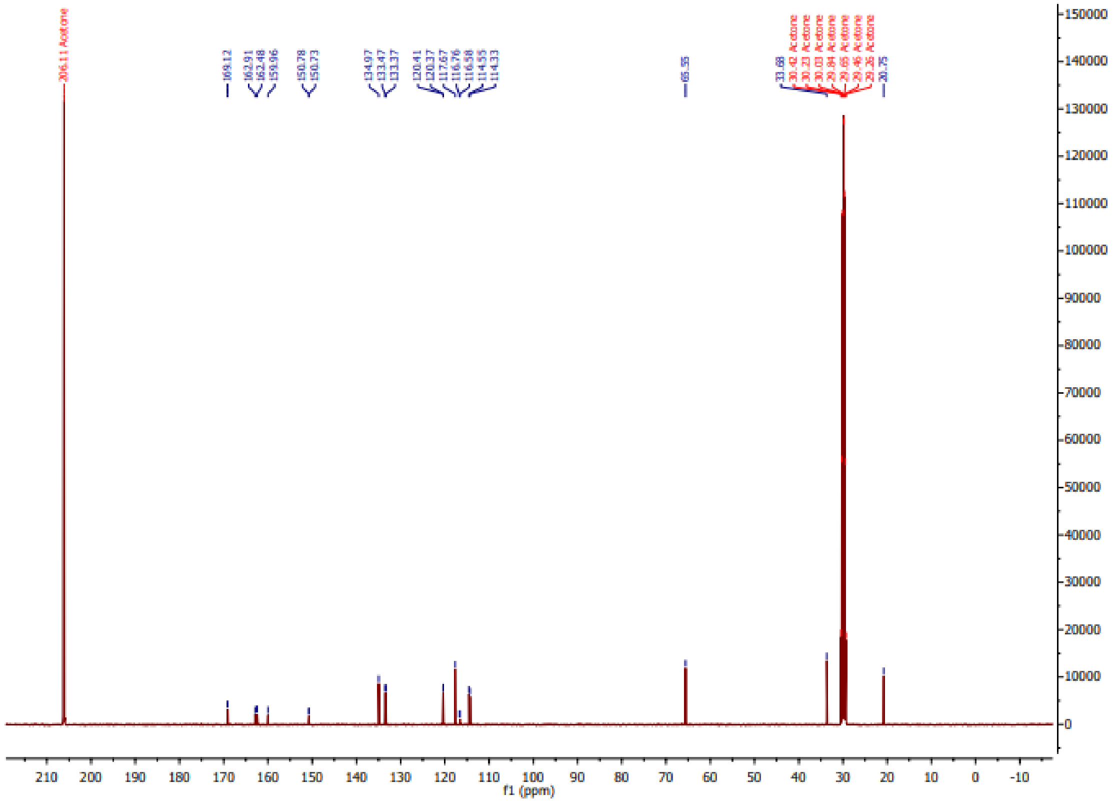

Supporting Figure S33: ^{1}H and ^{13}C NMR spectra of 3-Cl-ASA-Prop

^{1}H NMR spectrum of 3-Cl-ASA-Prop

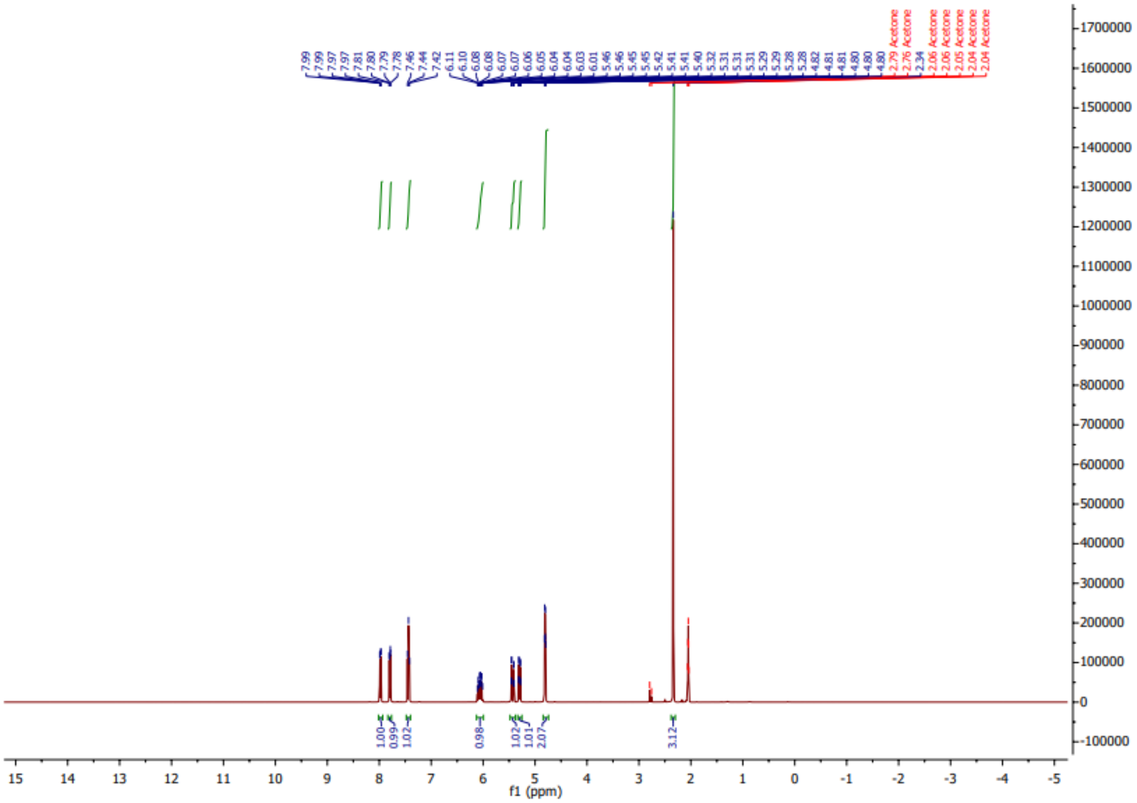

^{13}C NMR spectrum of 3-Cl-ASA-Prop

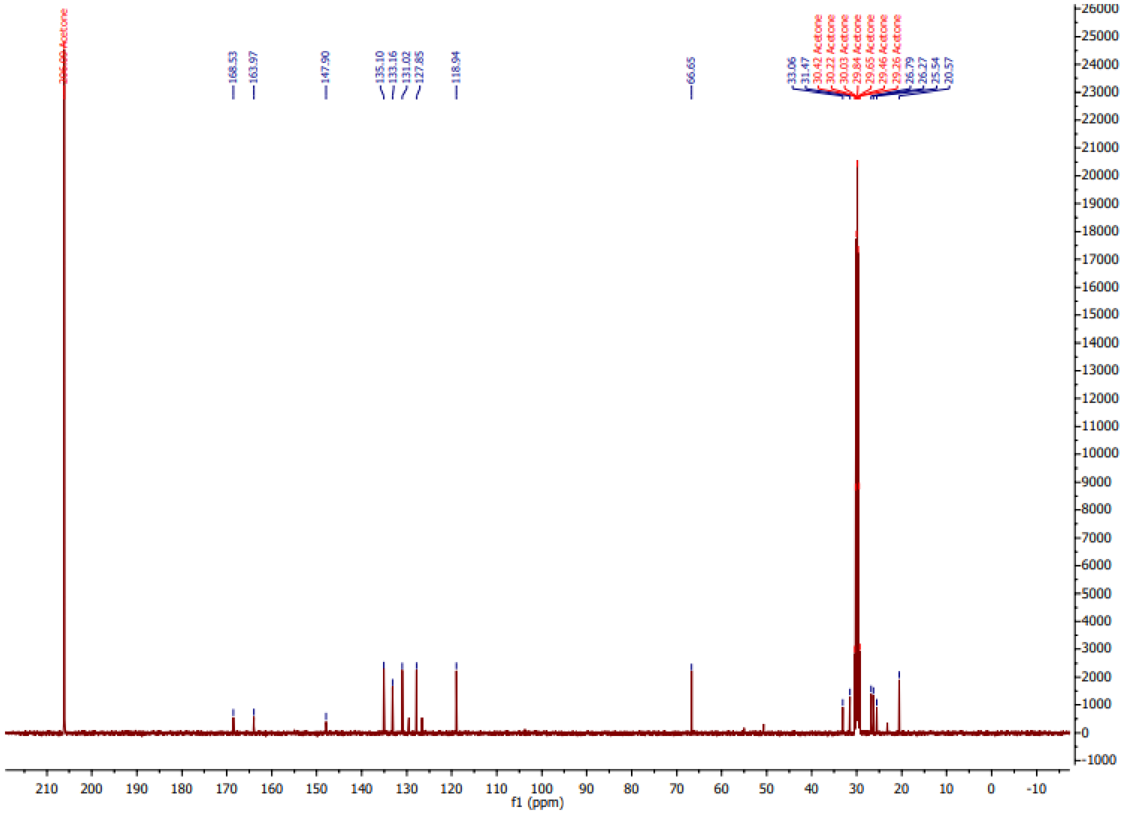

Supporting Figure S34: ^{1}H and ^{13}C NMR spectra of 3-Cl-ASA-But

^{1}H NMR spectrum of 3-Cl-ASA-But

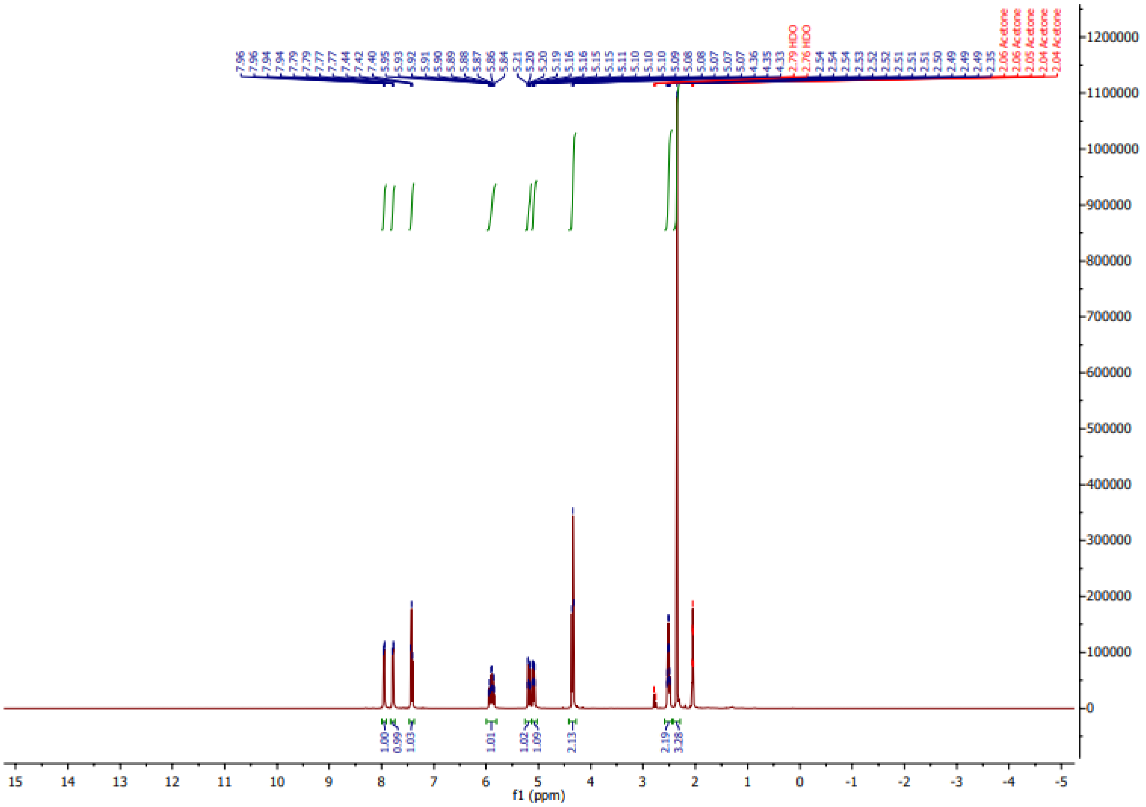

^{13}C NMR spectrum of 3-Cl-ASA-But

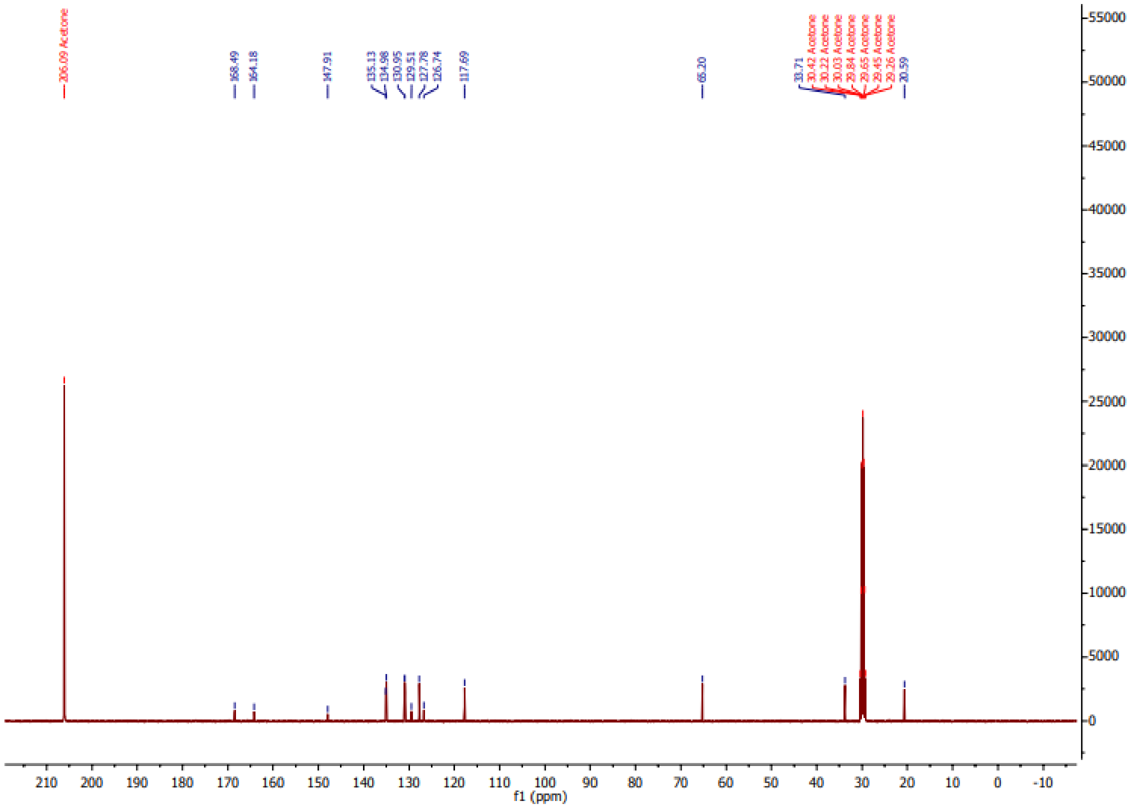

Supporting Figure S35: ^{1}H and ^{13}C NMR spectra of 4-Cl-ASA-Prop

^{1}H NMR spectrum of 4-Cl-ASA-Prop

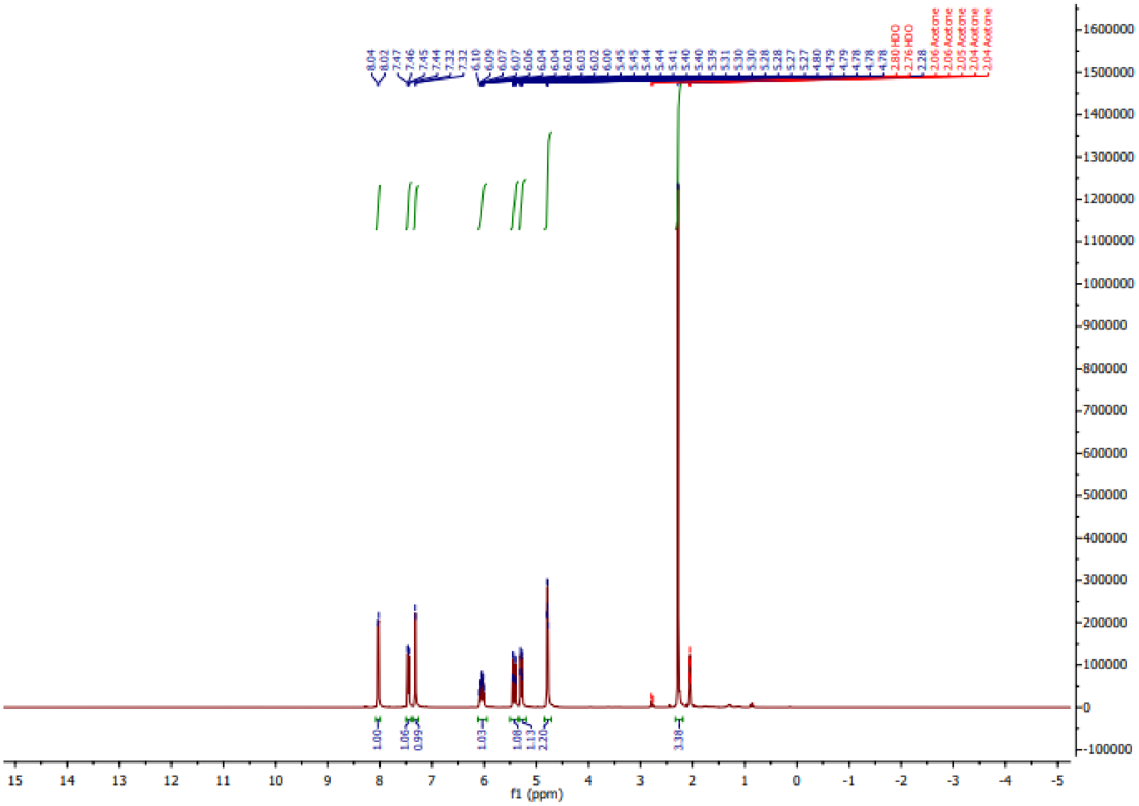

^{13}C NMR spectrum of 4-Cl-ASA-Prop

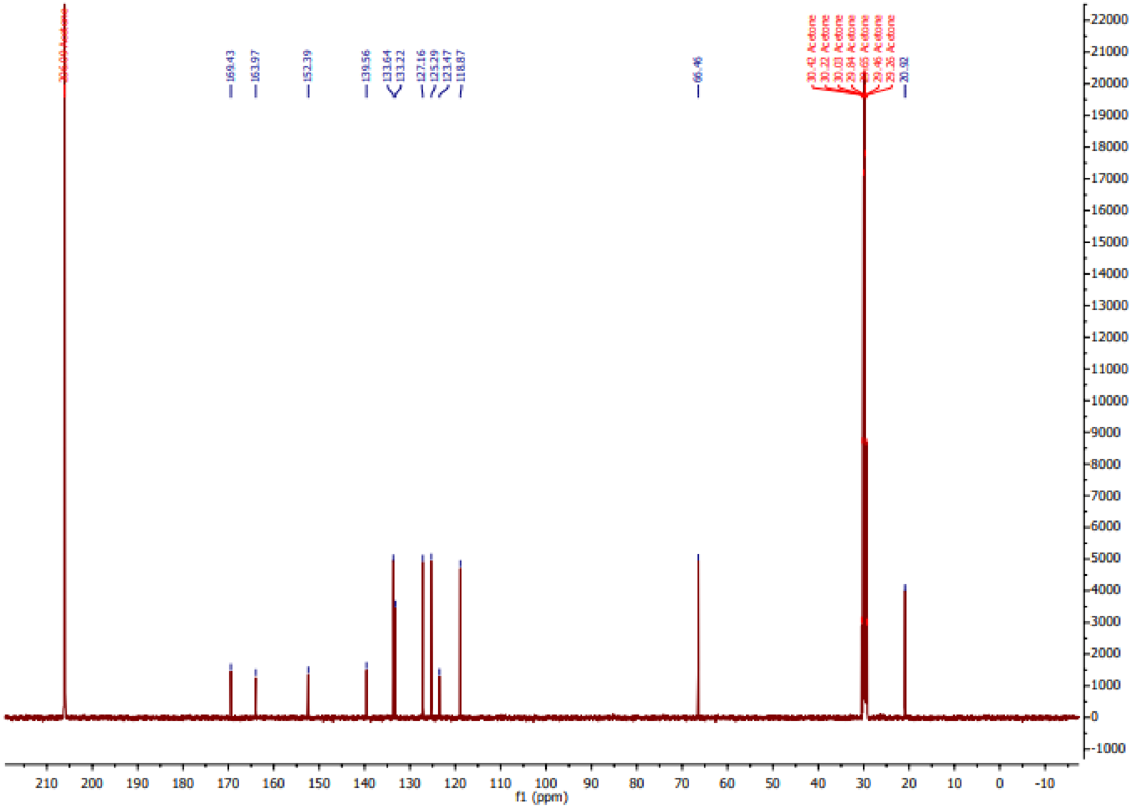

Supporting Figure S36: ^{1}H and ^{13}C NMR spectra of 4-Cl-ASA-But

^{1}H NMR spectrum of 4-Cl-ASA-But

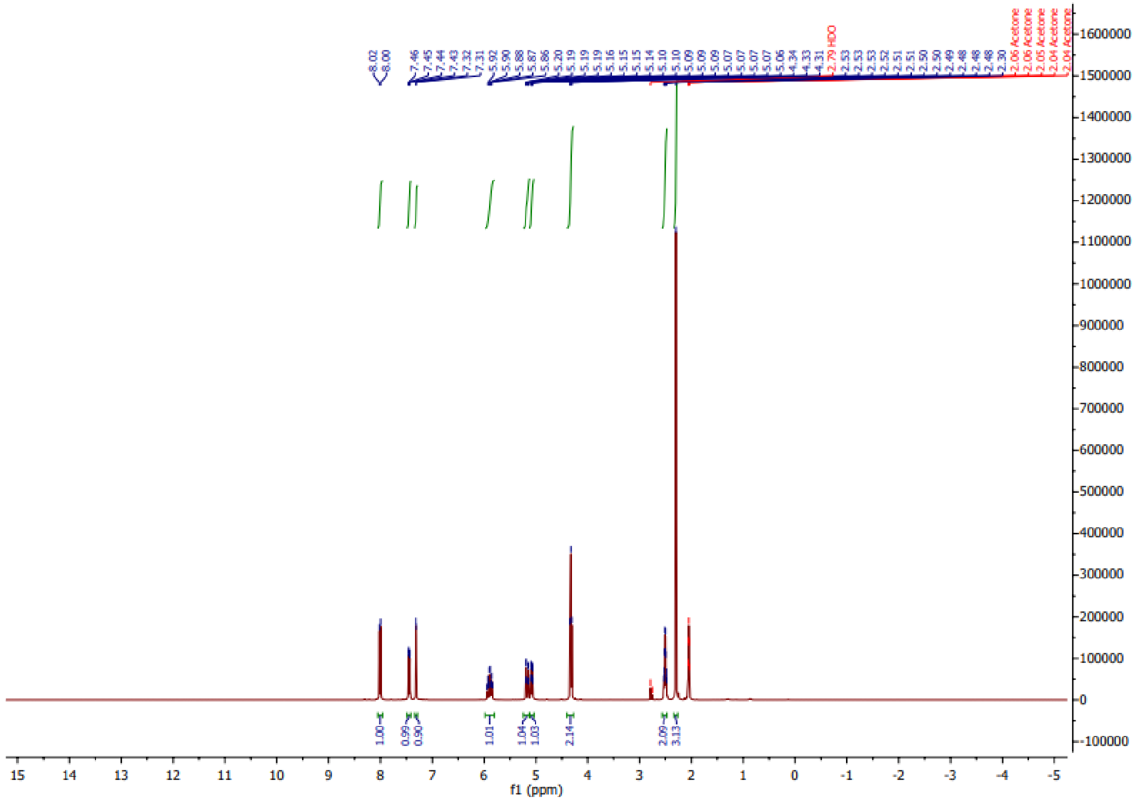

^{13}C NMR spectrum of 4-Cl-ASA-But

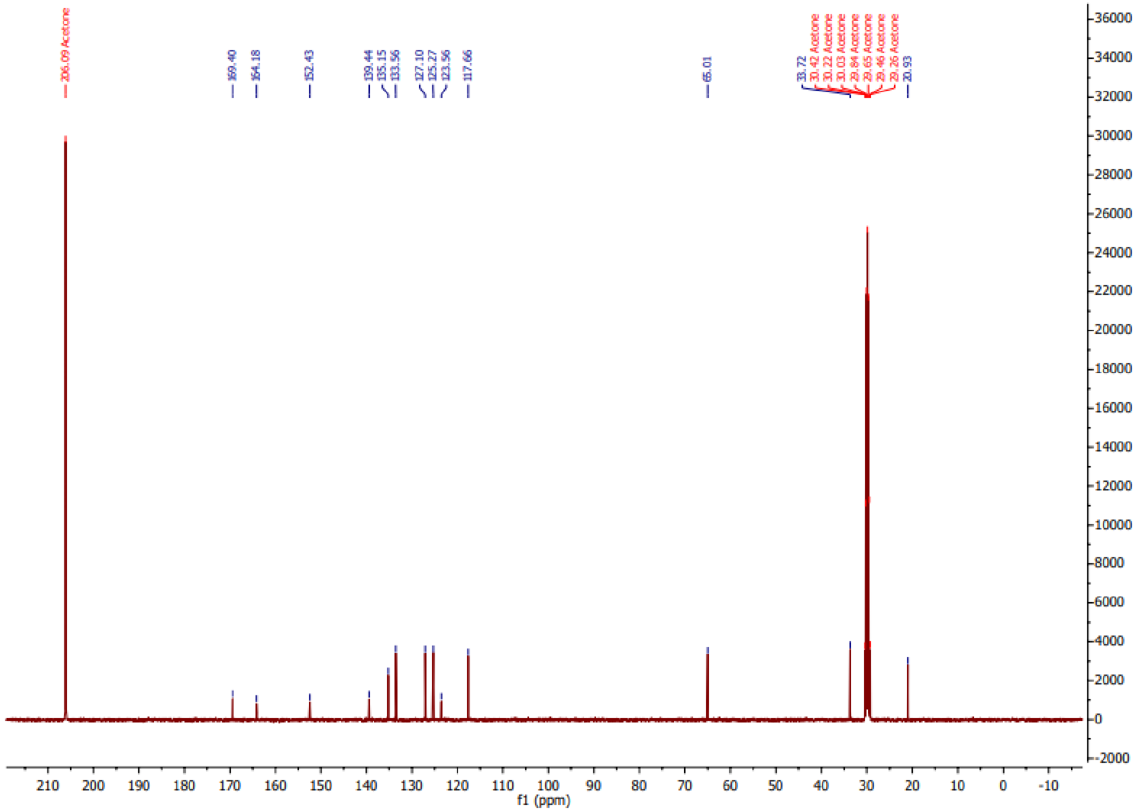

Supporting Figure S37: ^{1}H and ^{13}C NMR spectra of 5-Cl-ASA-Prop

^{1}H NMR spectrum of 5-Cl-ASA-Prop

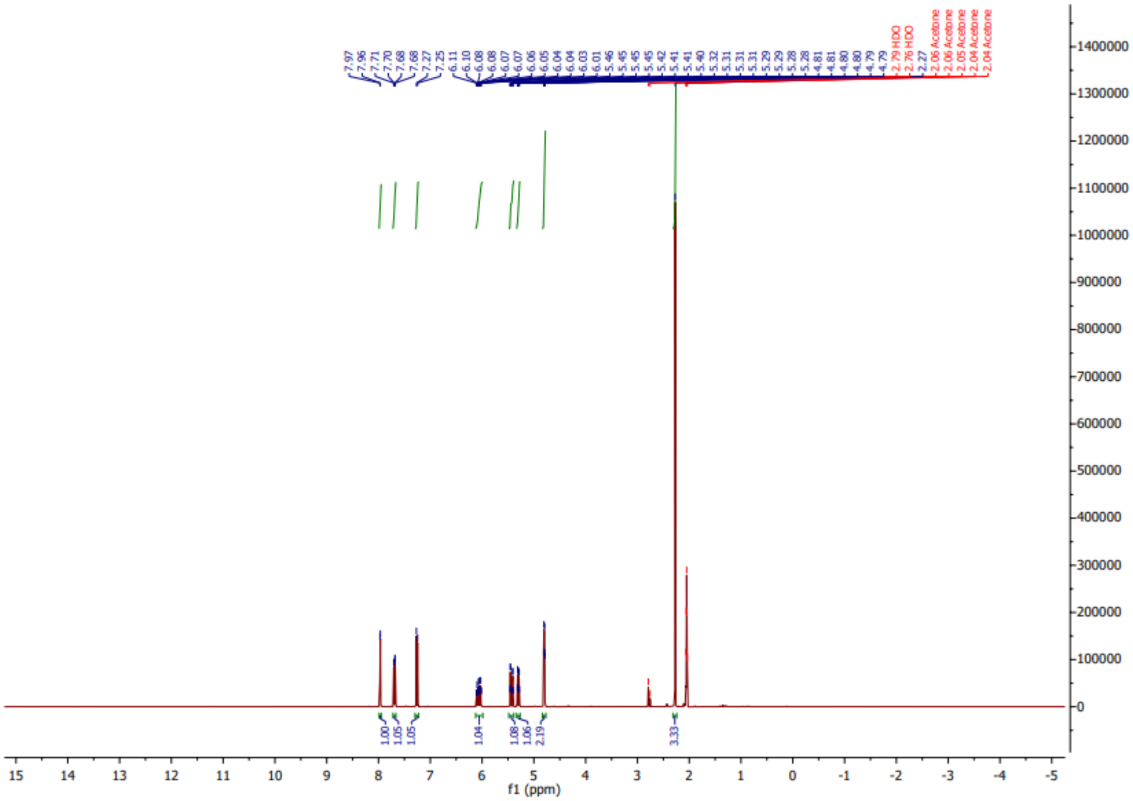

^{13}C NMR spectrum of 5-Cl-ASA-Prop

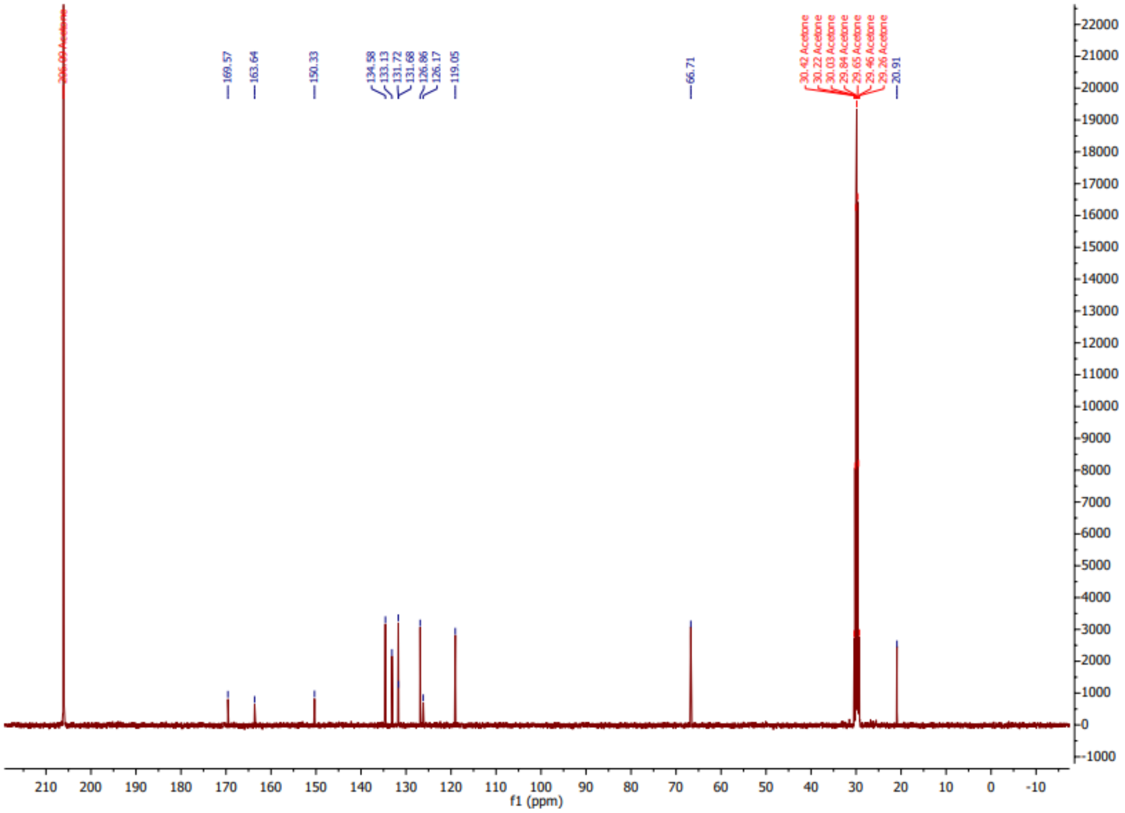

<u>Supporting Figure S38: ^{1}H and ^{13}C NMR spectra of 5-Cl-ASA-But</u>

^{1}H NMR spectrum of 5-Cl-ASA-But

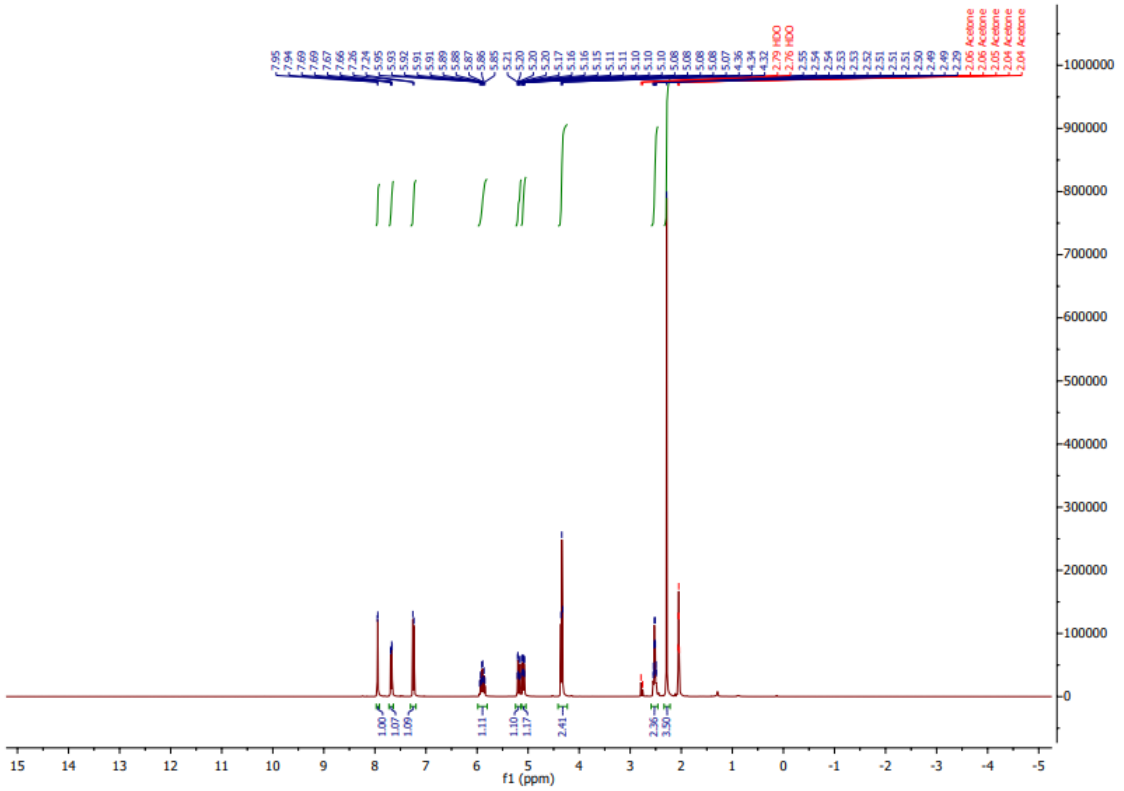

^{13}C NMR spectrum of 5-Cl-ASA-But

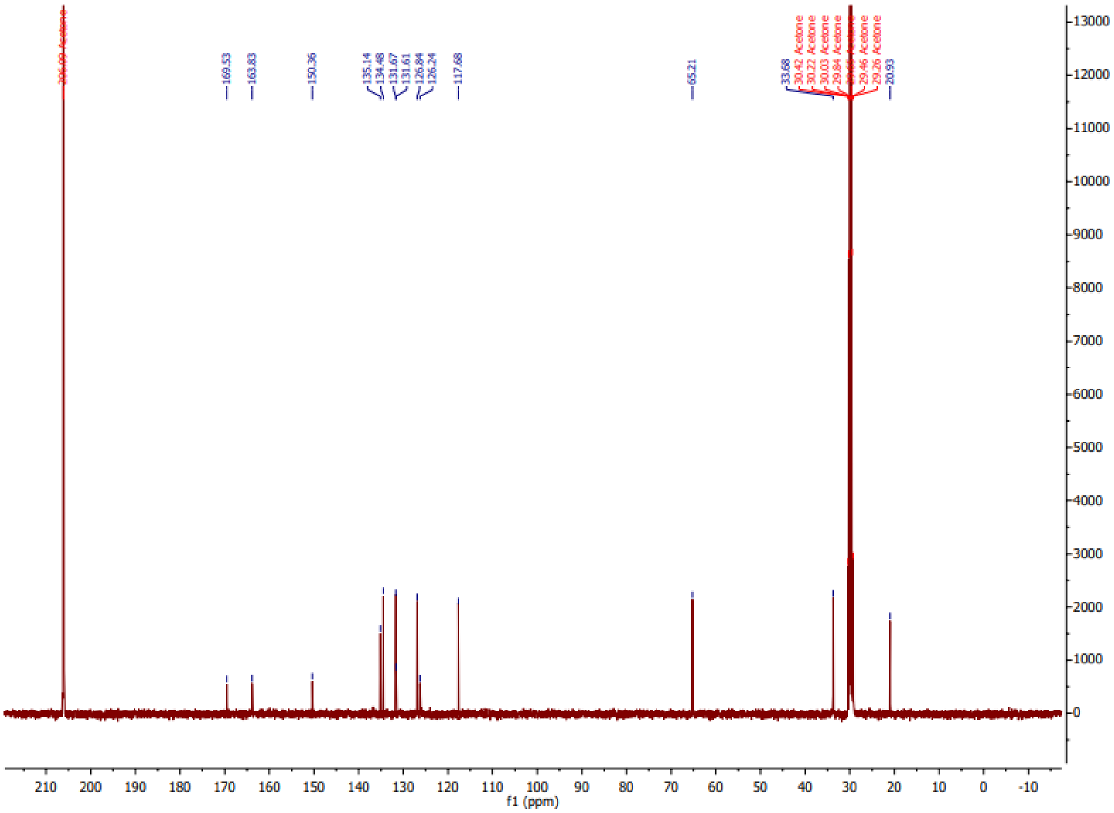

Supporting Figure S39: ^{1}H and ^{13}C NMR spectra of 6-Cl-ASA-Prop

^{1}H NMR spectrum of 6-Cl-ASA-Prop

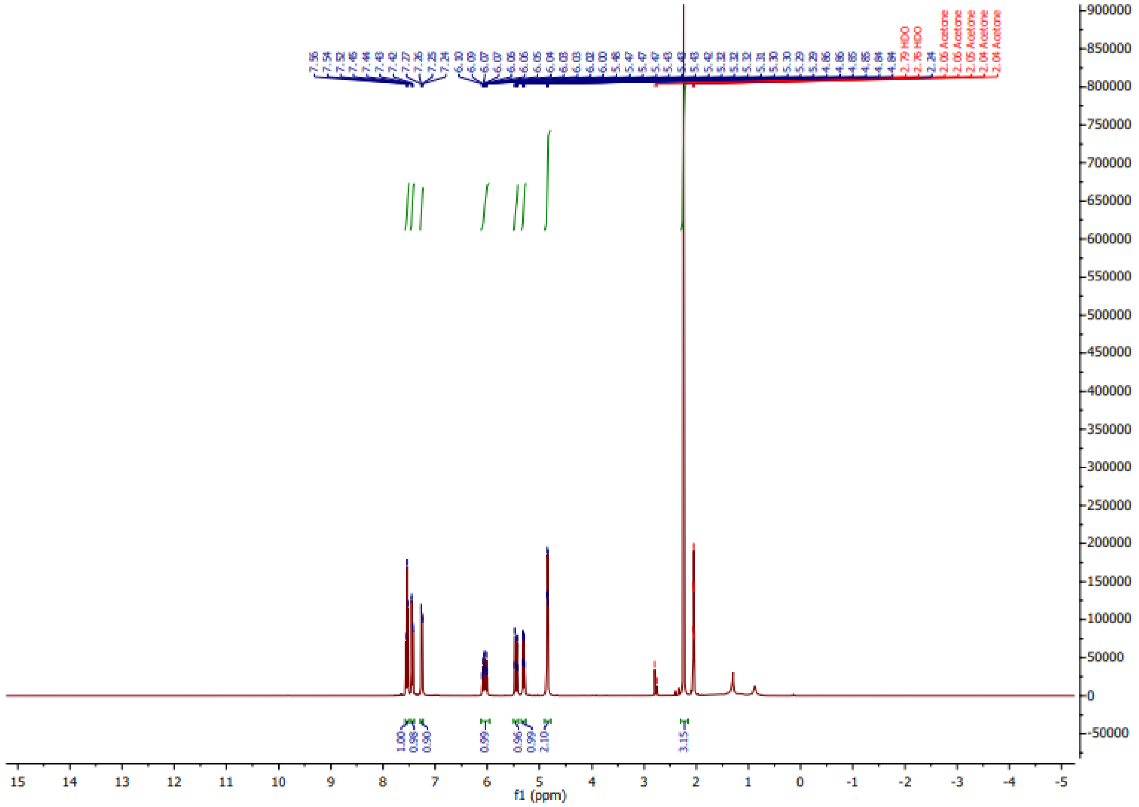

^{13}C NMR spectrum of 6-Cl-ASA-Prop

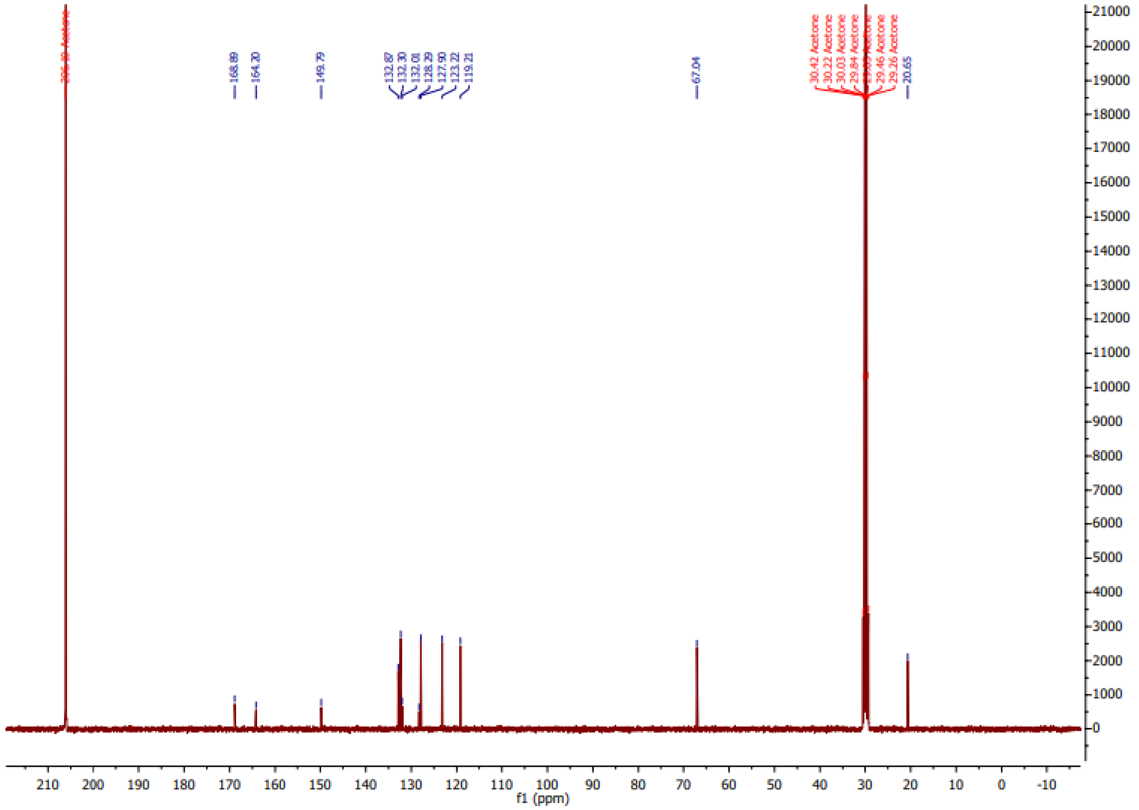

Supporting Figure S40: ^{1}H and ^{13}C NMR spectra of 6-Cl-ASA-But

^{1}H NMR spectrum of 6-Cl-ASA-But

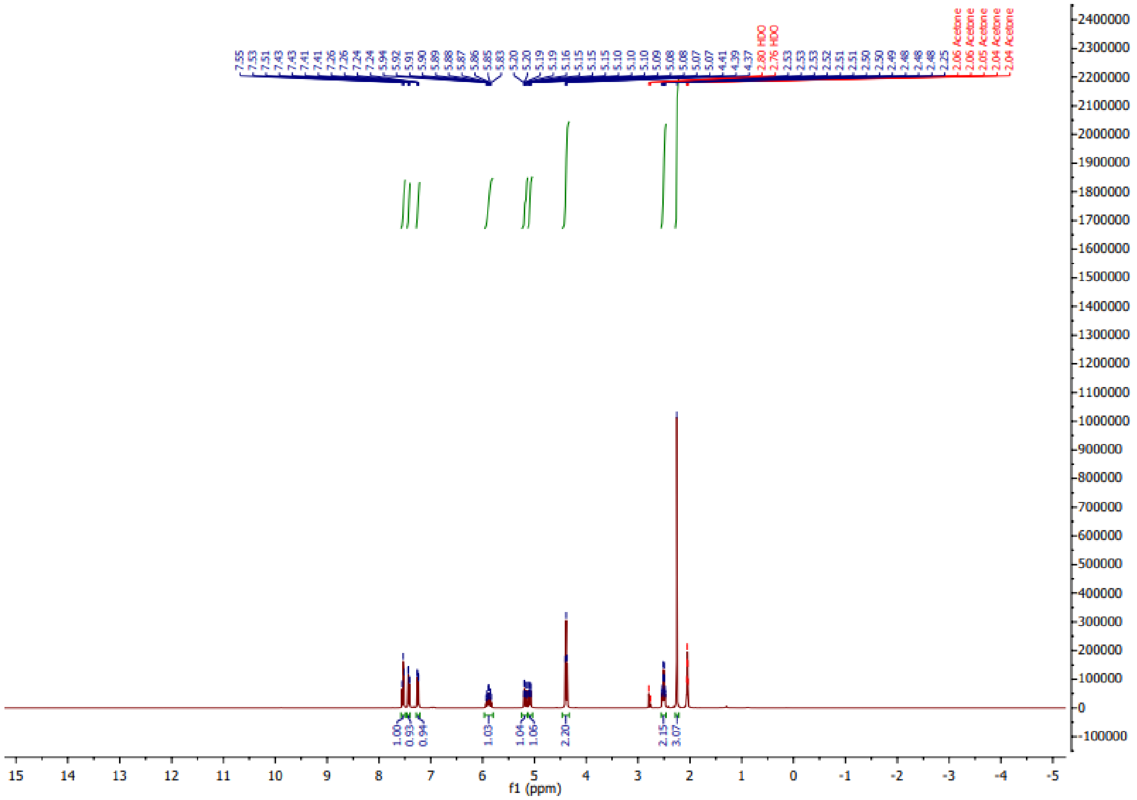

^{13}C NMR spectrum of 6-Cl-ASA-But

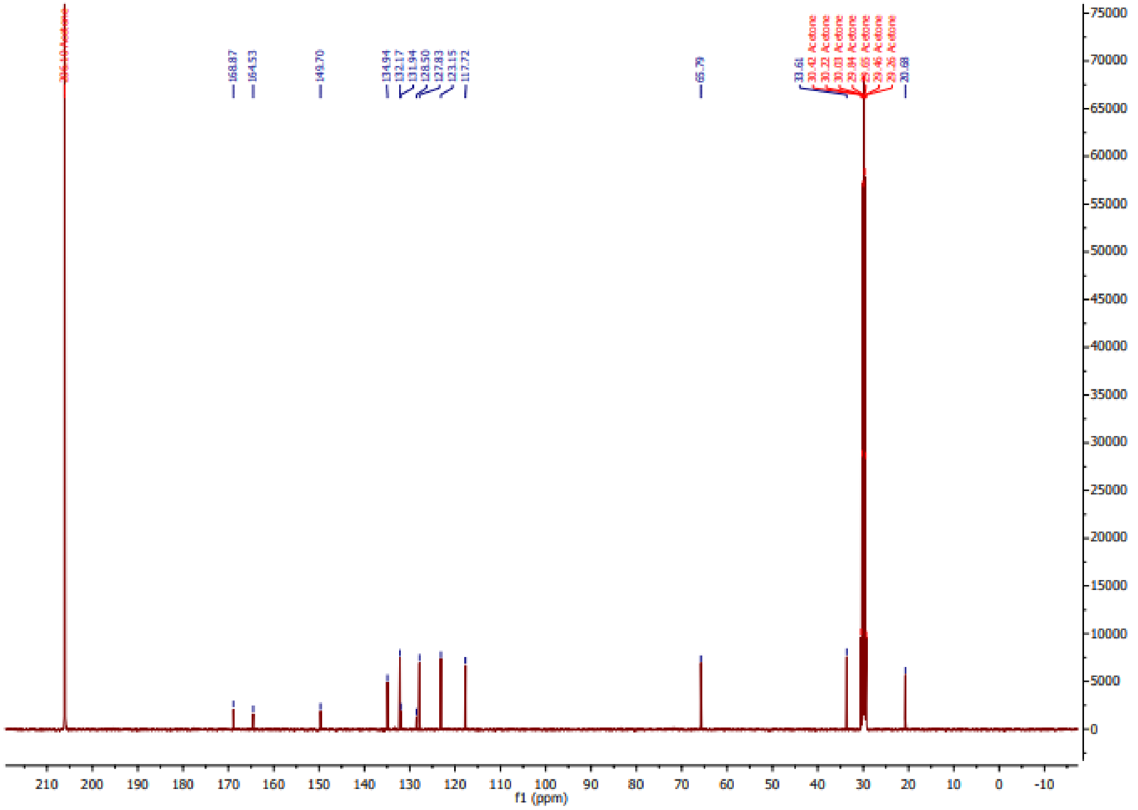

Supporting Figure S41: ^{1}H and ^{13}C NMR spectra of 3-CH_3-ASA-Prop

^{1}H NMR spectrum of 3-CH_3-ASA-Prop

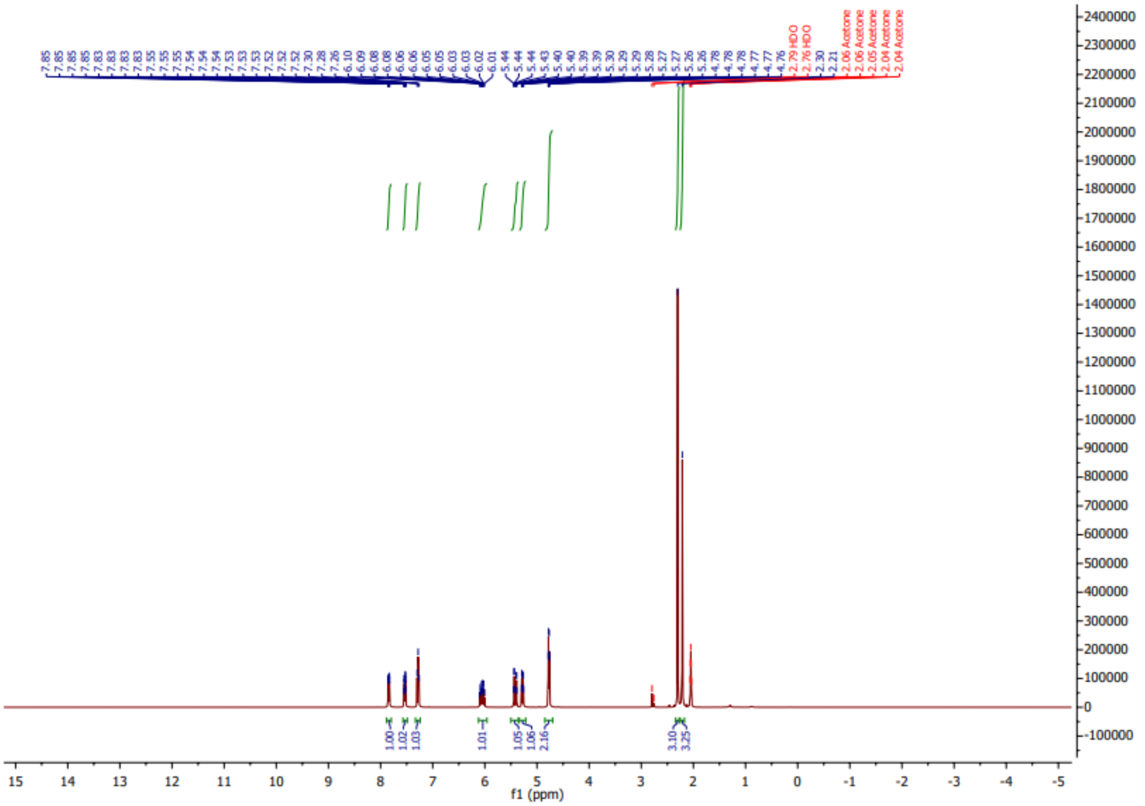

^{13}C NMR spectrum of 3-CH_3-ASA-Prop

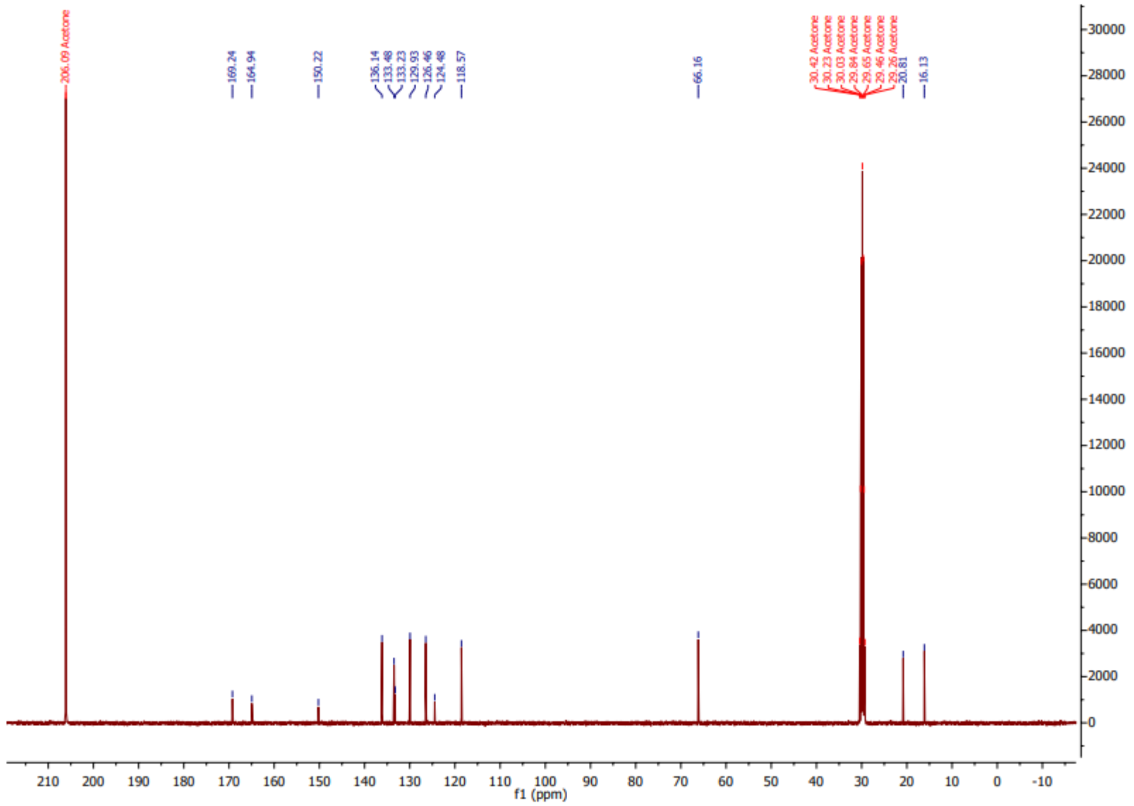

Supporting Figure S42: ^{1}H and ^{13}C NMR spectra of 3-CH_3-ASA-But

^{1}H NMR spectrum of 3-CH_3-ASA-But

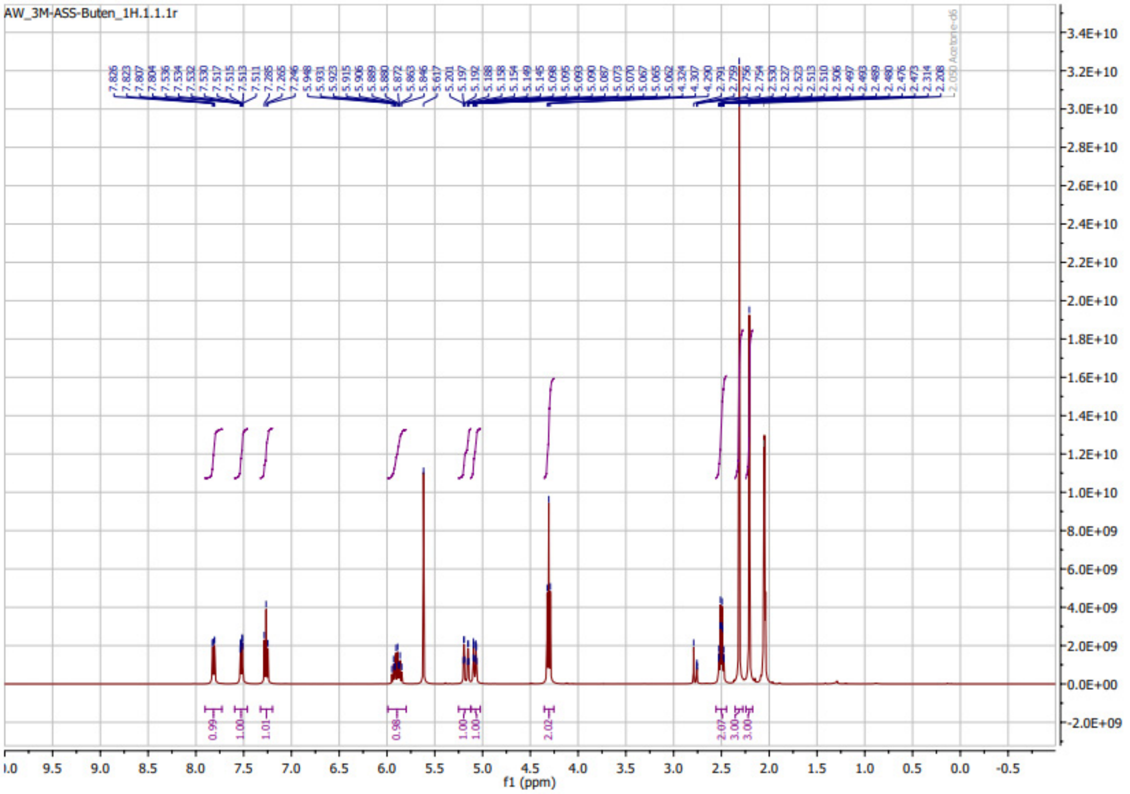

^{13}C NMR spectrum of 3-CH_3-ASA-But

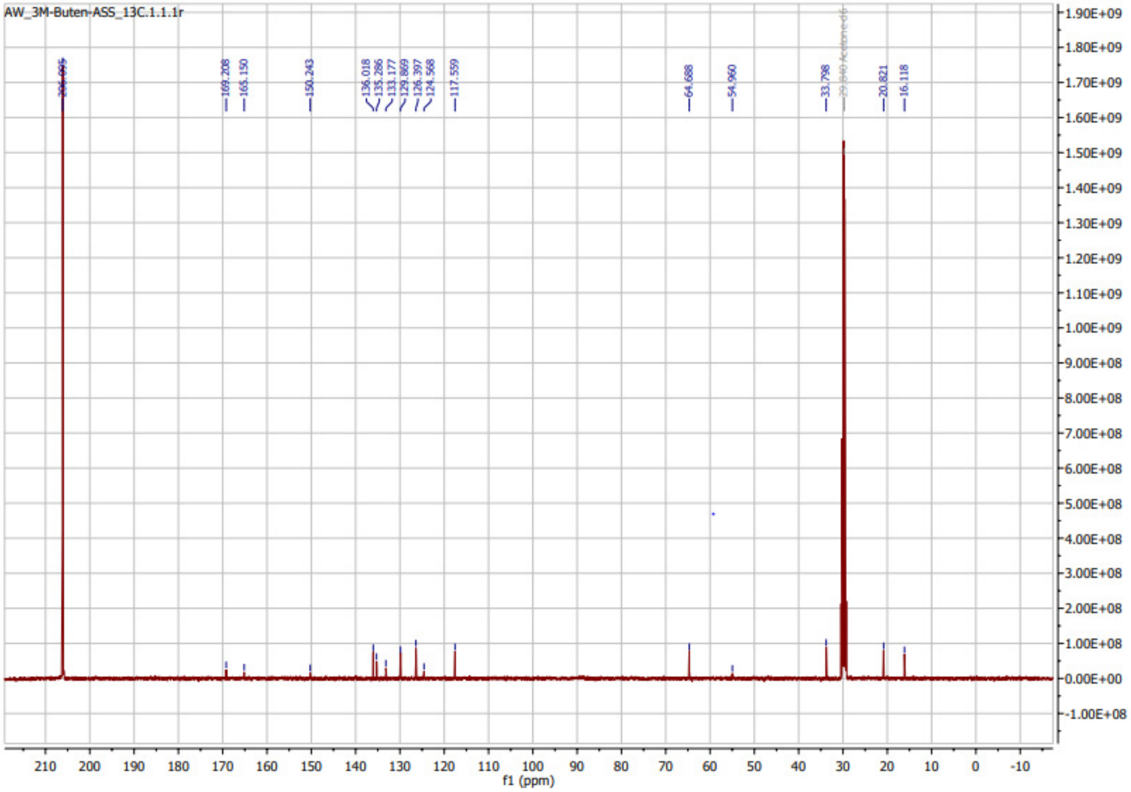

Supporting Figure S43: ^{1}H and ^{13}C NMR spectra of 4-CH_3-ASA-Prop

^{1}H NMR spectrum of 4-CH_3-ASA-Prop

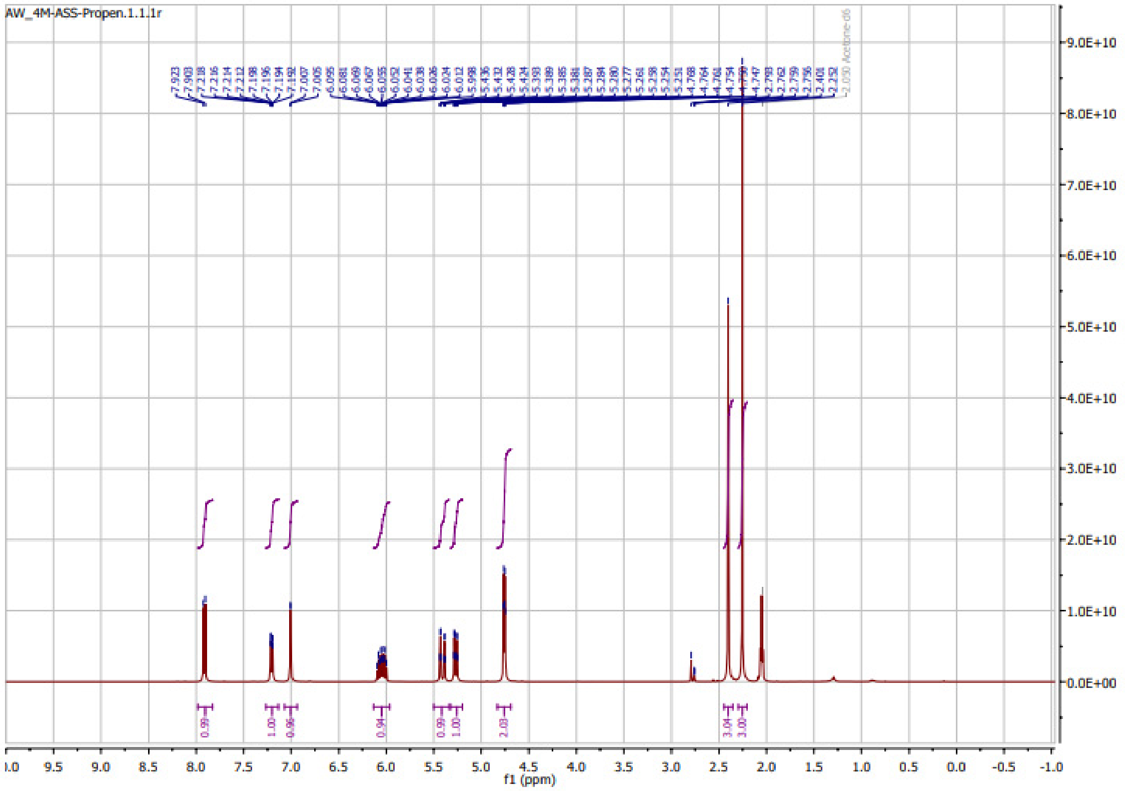

^{13}C NMR spectrum of 4-CH_3-ASA-Prop

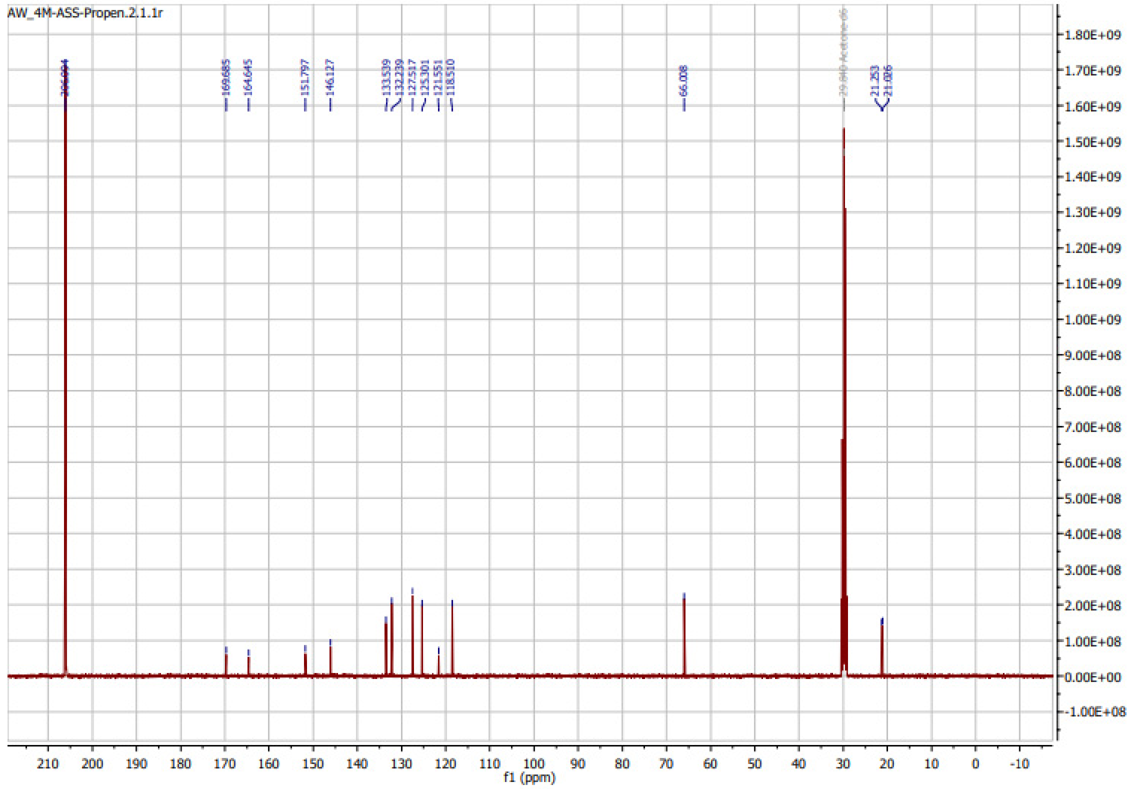

Supporting Figure S44: ^{1}H NMR spectrum of 4-CH_3-ASA-But

^{1}H NMR spectrum of 4-CH_3-ASA-But

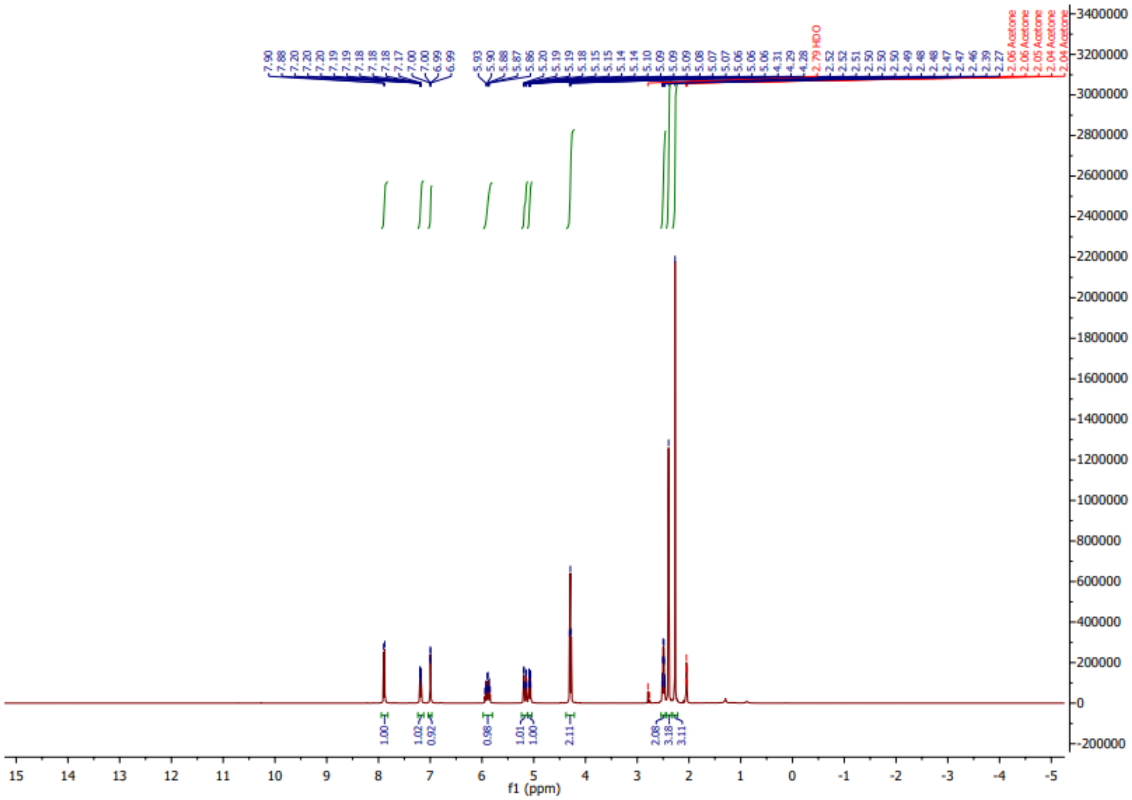

Supporting Figure S45: ^{1}H and ^{13}C NMR spectra of 5-CH_3-ASA-Prop

^{1}H NMR spectrum of 5-CH_3-ASA-Prop

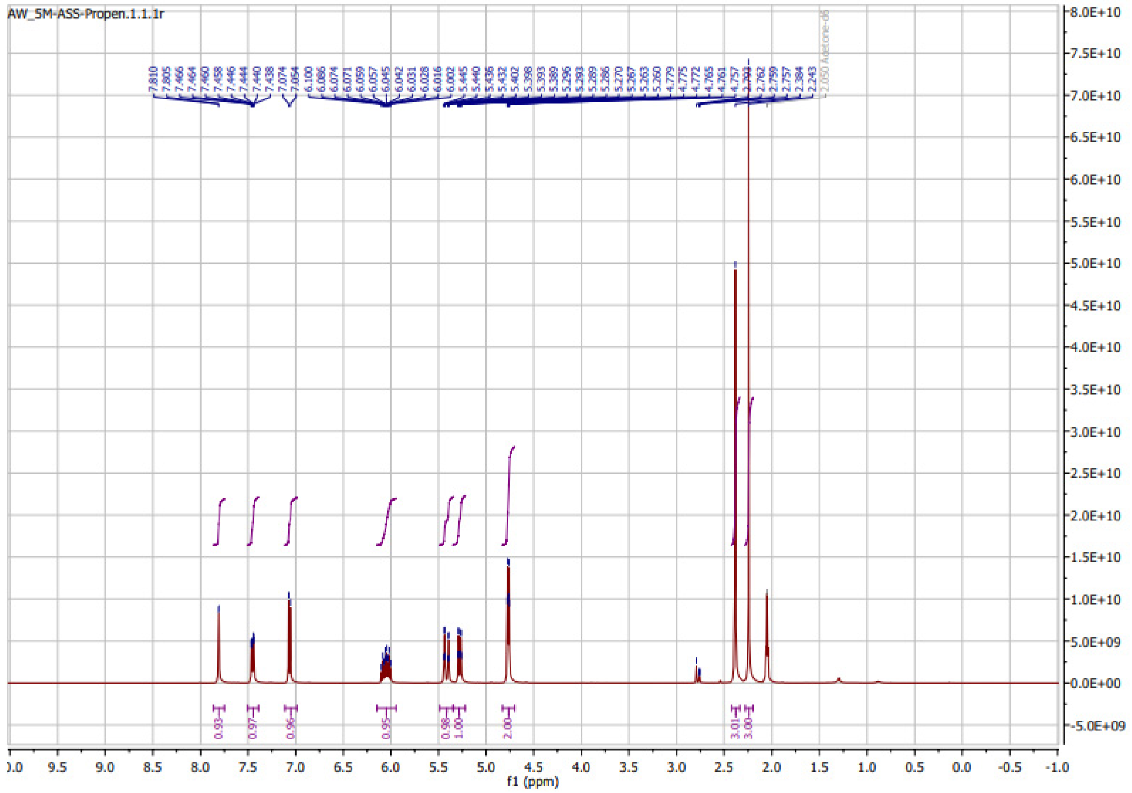

^{13}C NMR spectrum of 5-CH_3-ASA-Prop

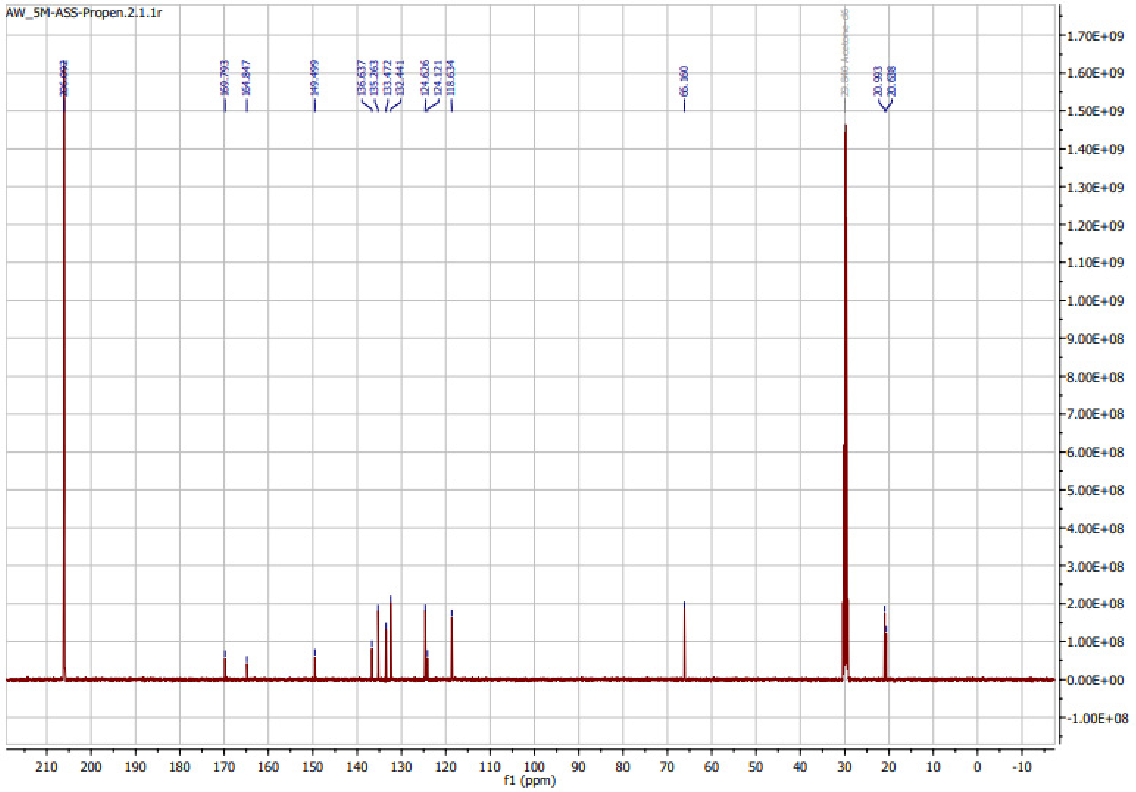

Supporting Figure S46: ^{1}H and ^{13}C NMR spectra of 5-CH_3-ASA-But

^{1}H NMR spectrum of 5-CH_3-ASA-But

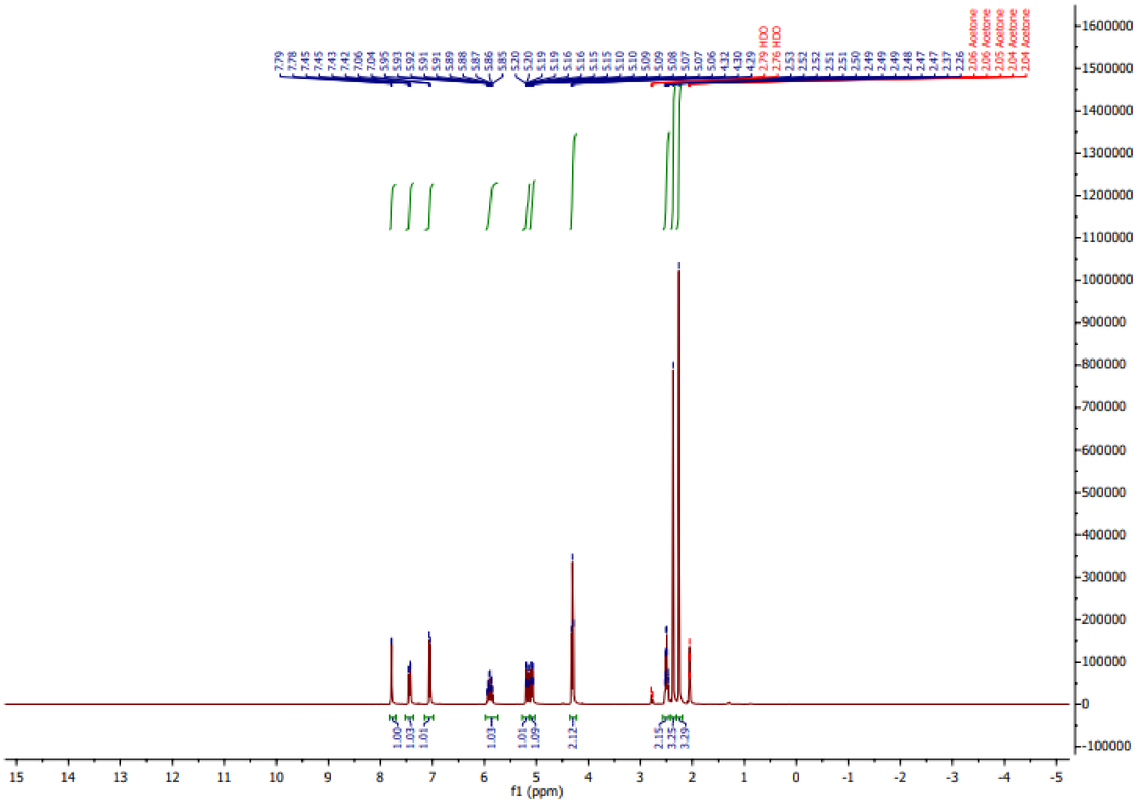

^{13}C NMR spectrum of 5-CH_3-ASA-But

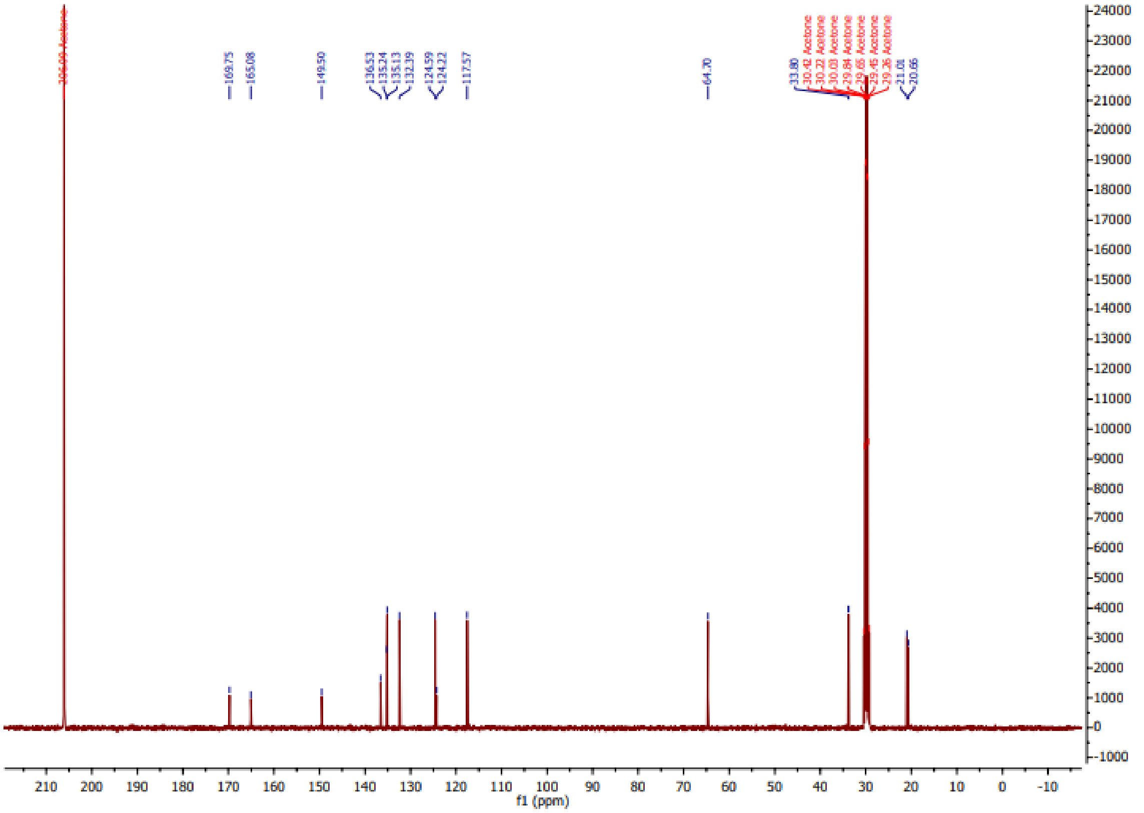

Supporting Figure S47: ^{1}H and ^{13}C NMR spectra of 6-CH_3-ASA-Prop

^{1}H NMR spectrum of 6-CH_3-ASA-Prop

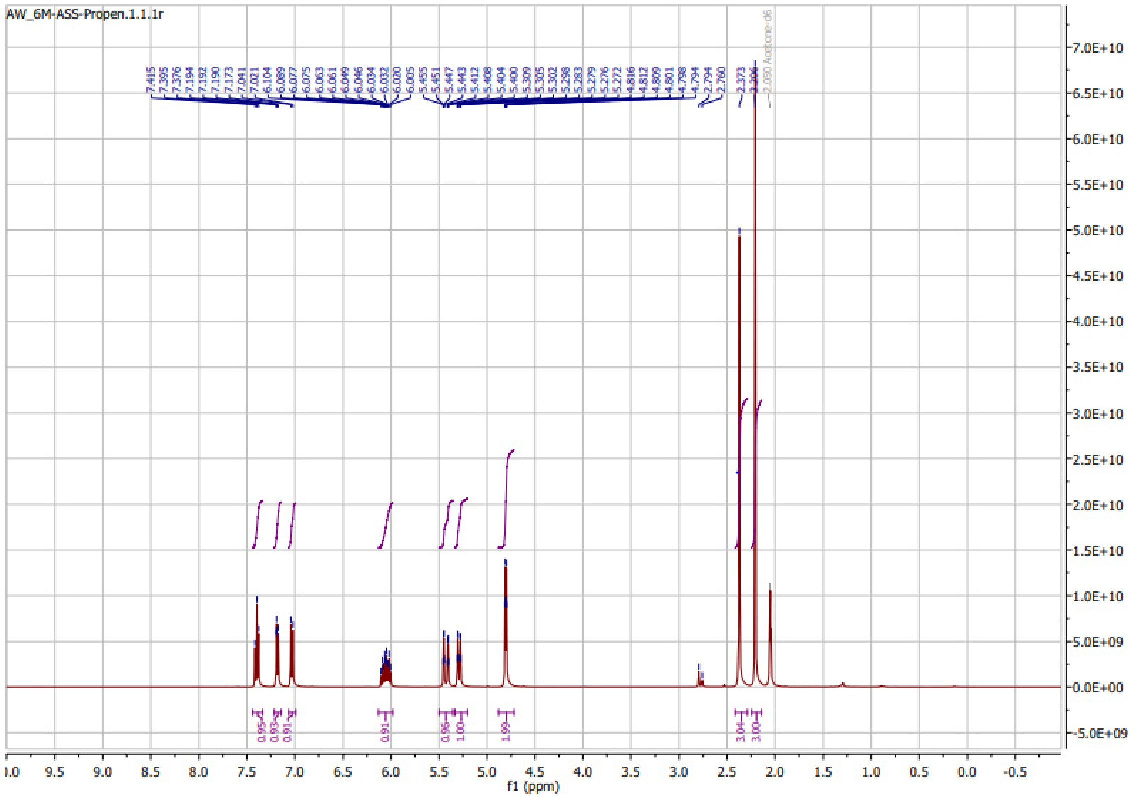

^{13}C NMR spectrum of 6-CH_3-ASA-Prop

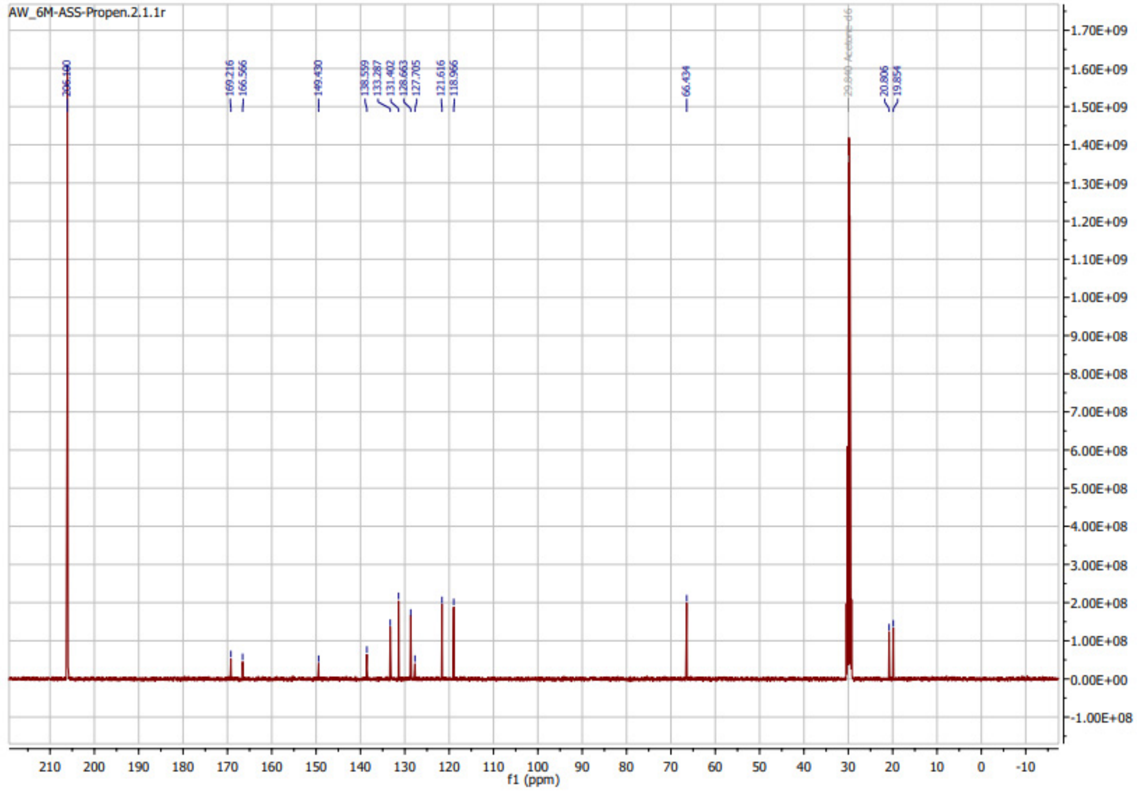

Supporting Figure S48: ^{1}H and ^{13}C NMR spectra of 6-CH3-ASA-But

^{1}H NMR spectrum of 6-CH_3-ASA-But

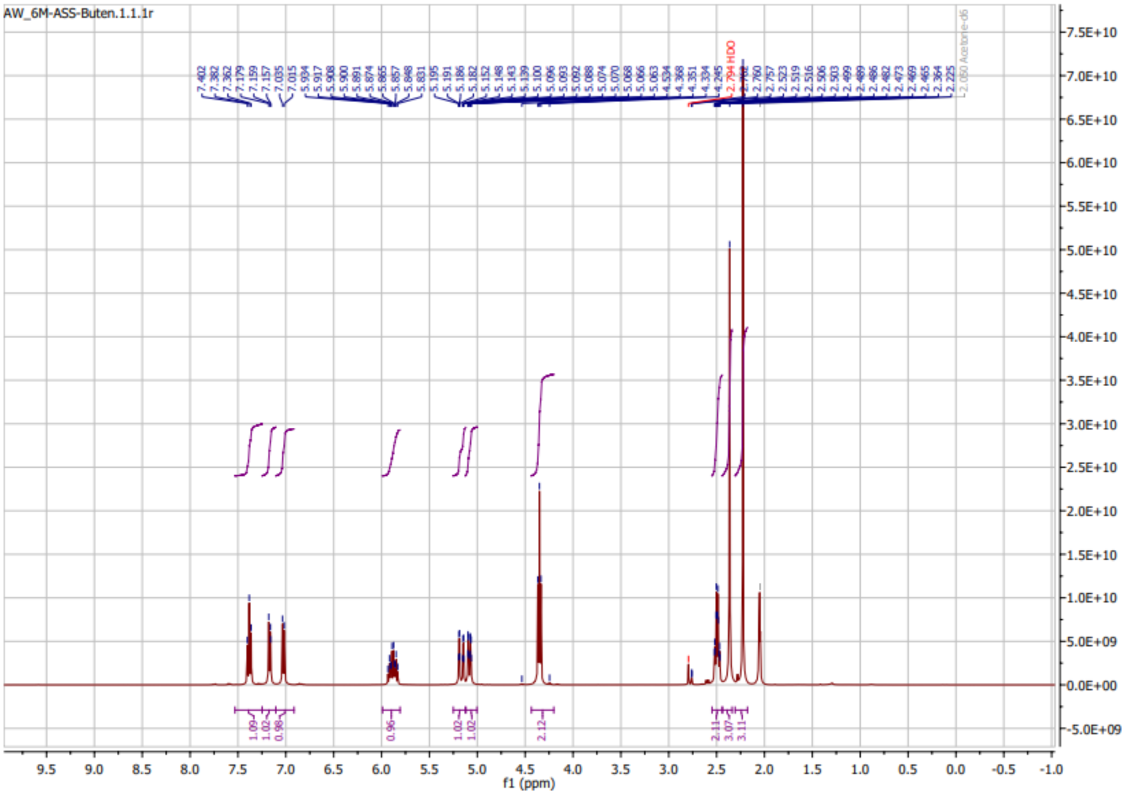

^{13}C NMR spectrum of 6-CH_3-ASA-But

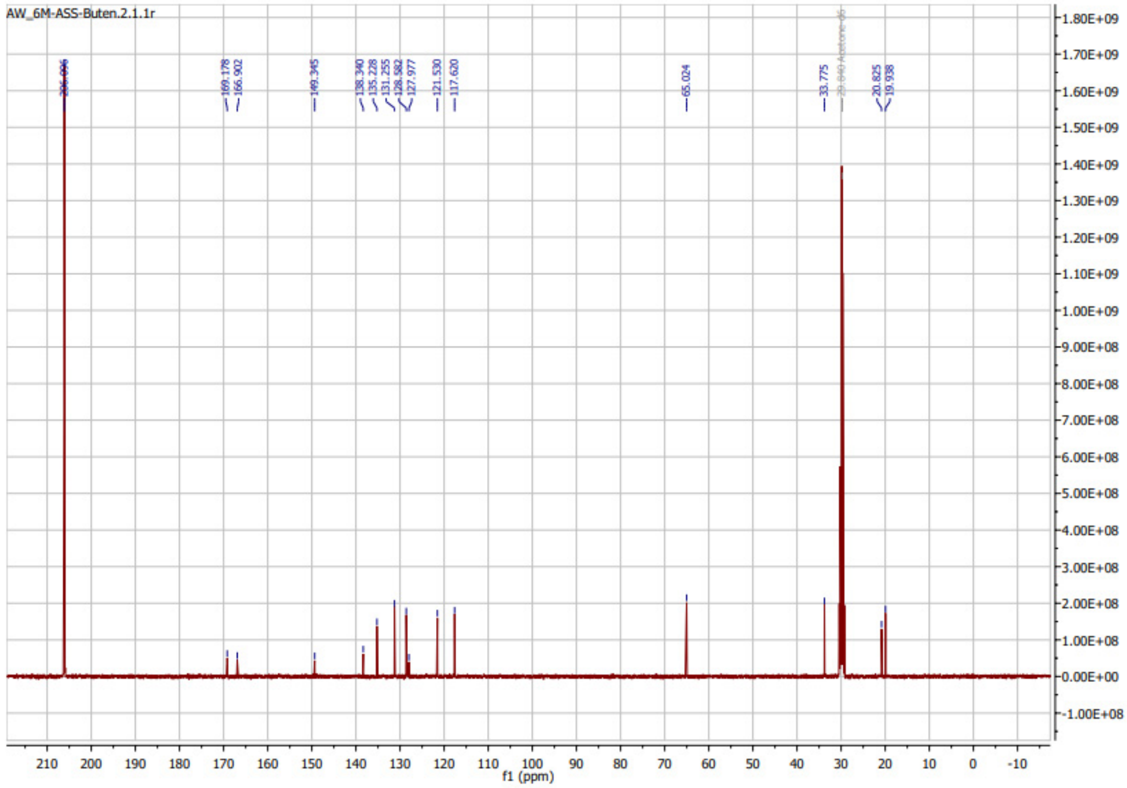

Supporting Figure S49: ^{1}H and ^{13}C NMR spectra of 3-F-ASA-Prop-PtCl$_3$

^{1}H NMR spectrum of 3-F-ASA-Prop-PtCl$_3$

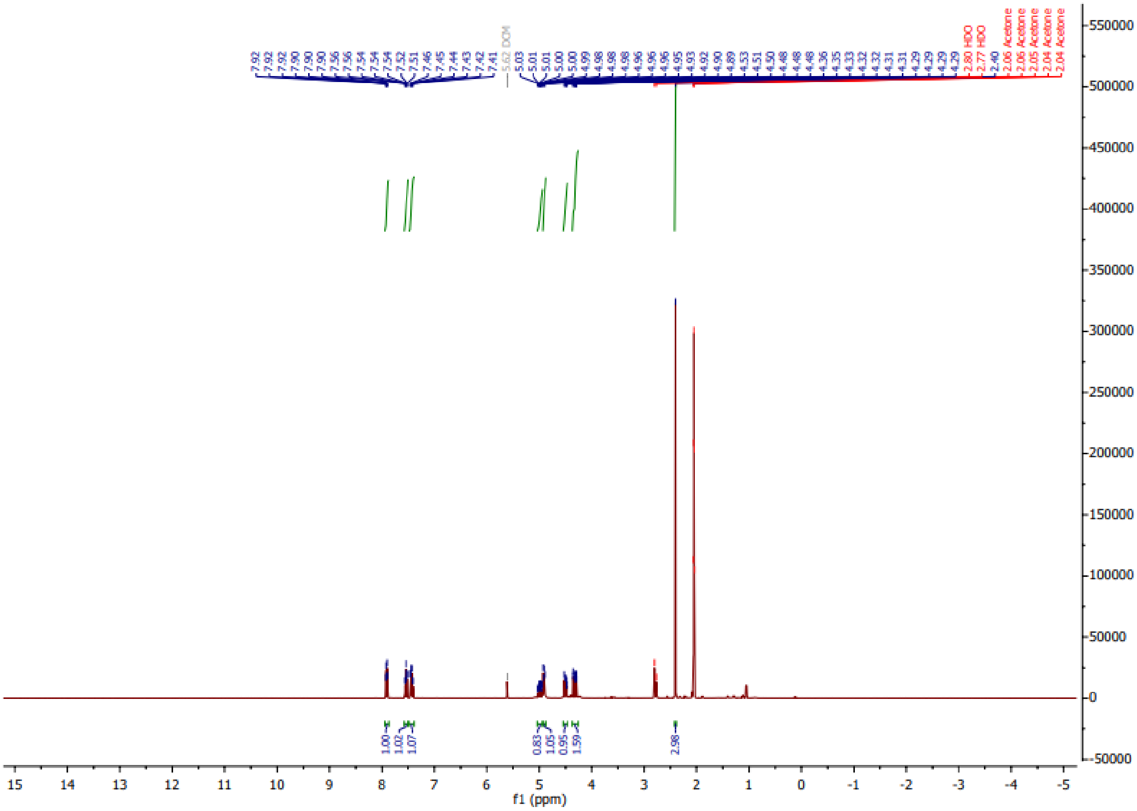

^{13}C NMR spectrum of 3-F-ASA-Prop-PtCl$_3$

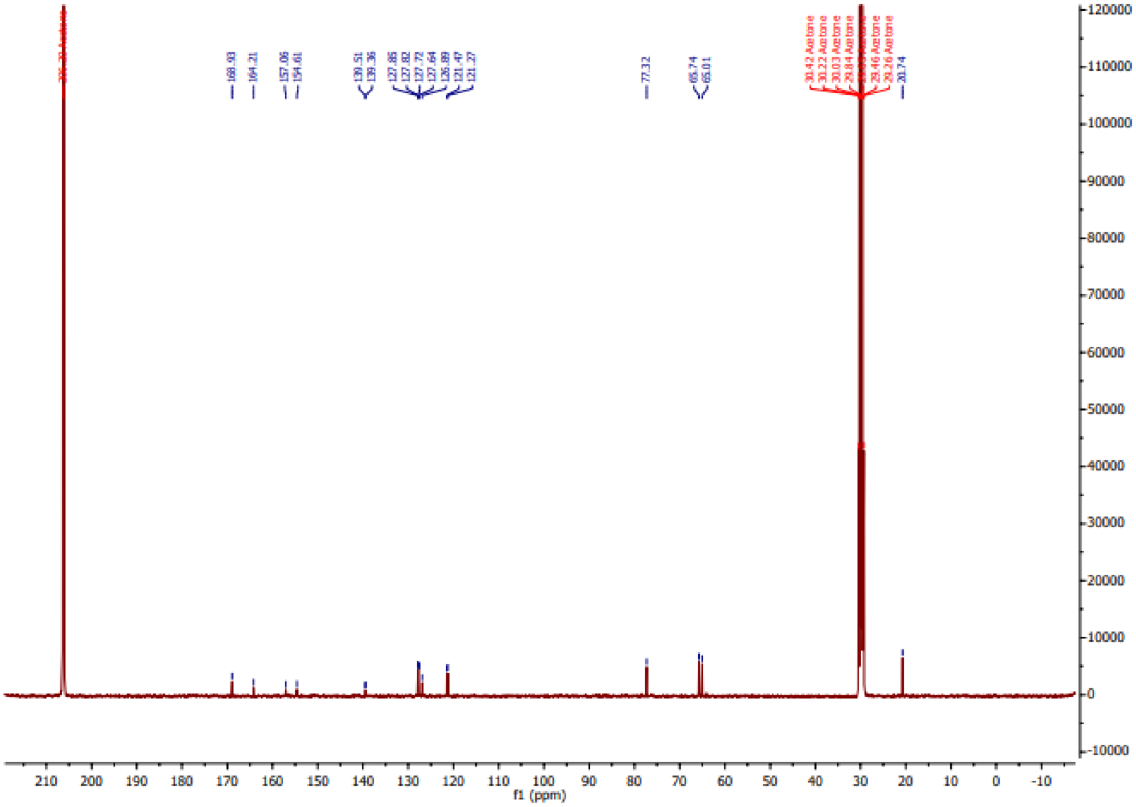

Supporting Figure S50: ^{1}H and ^{13}C NMR spectra of 3-F-ASA-But-PtCl$_3$

^{1}H NMR spectrum of 3-F-ASA-But-PtCl$_3$

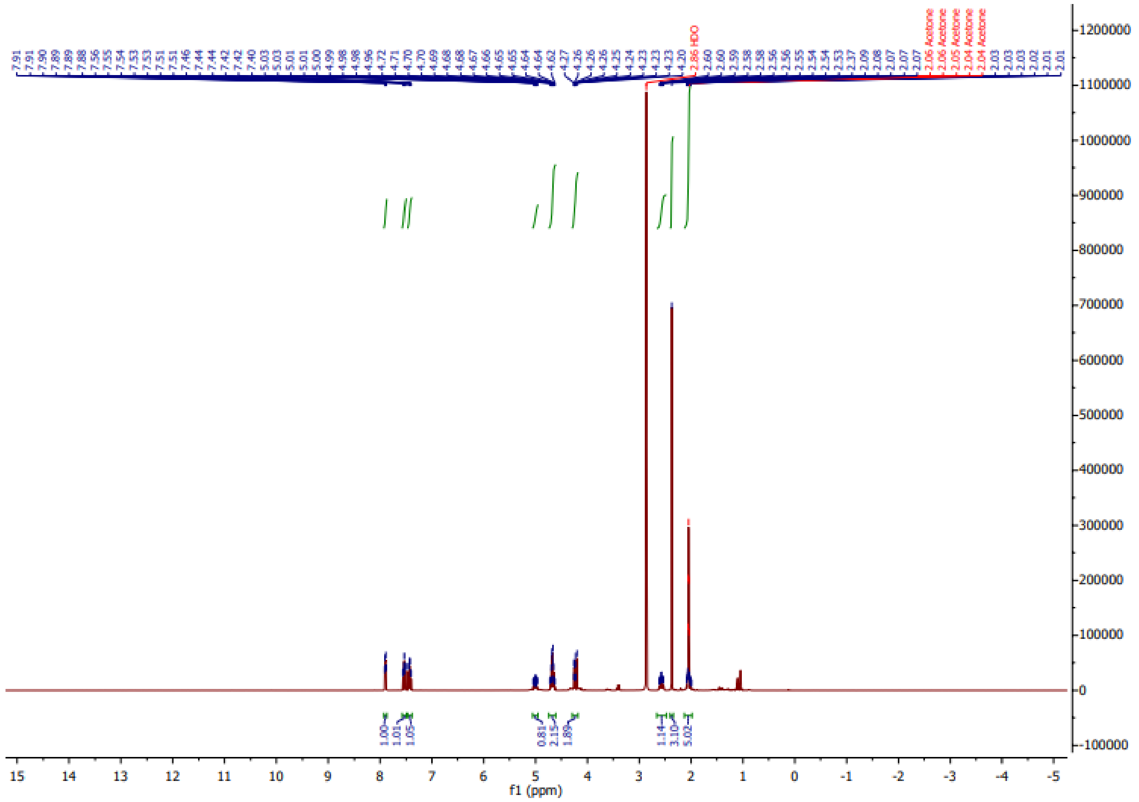

^{13}C NMR spectrum of 3-F-ASA-But-PtCl$_3$

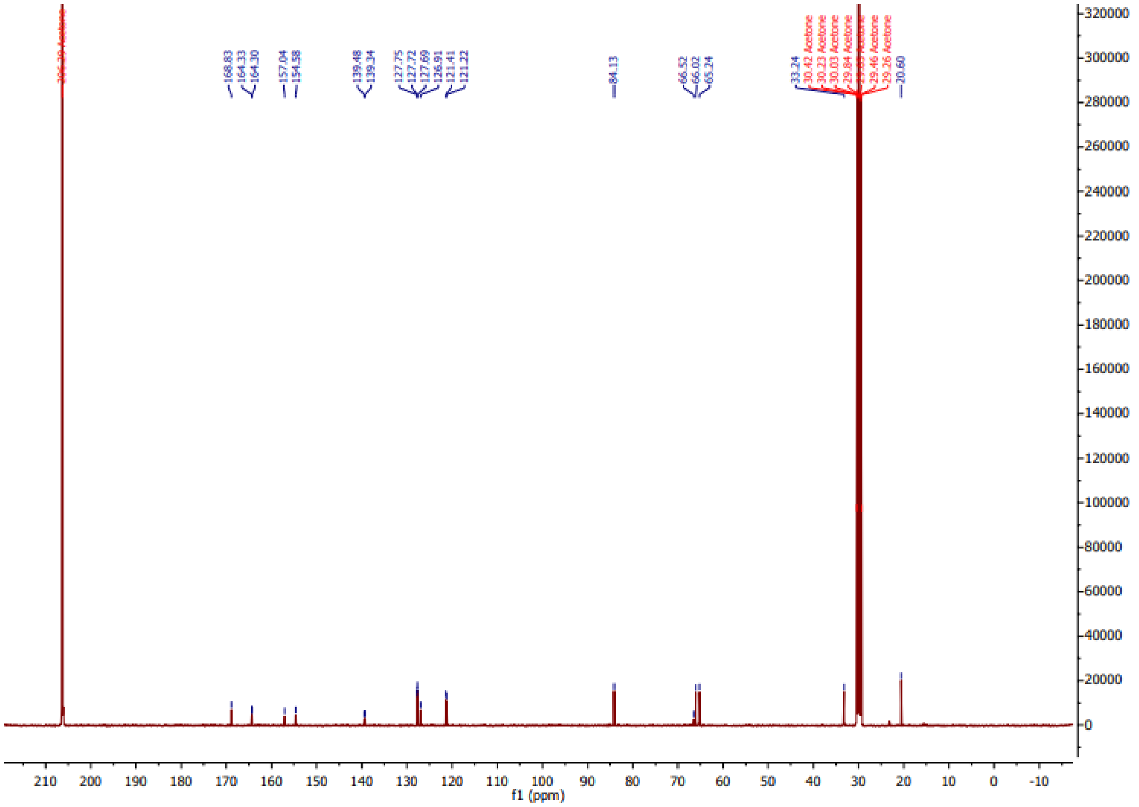

Supporting Figure S51: ^{1}H and ^{13}C NMR spectra of 4-F-ASA-Prop-PtCl$_3$

^{1}H NMR spectrum of 4-F-ASA-Prop-PtCl$_3$

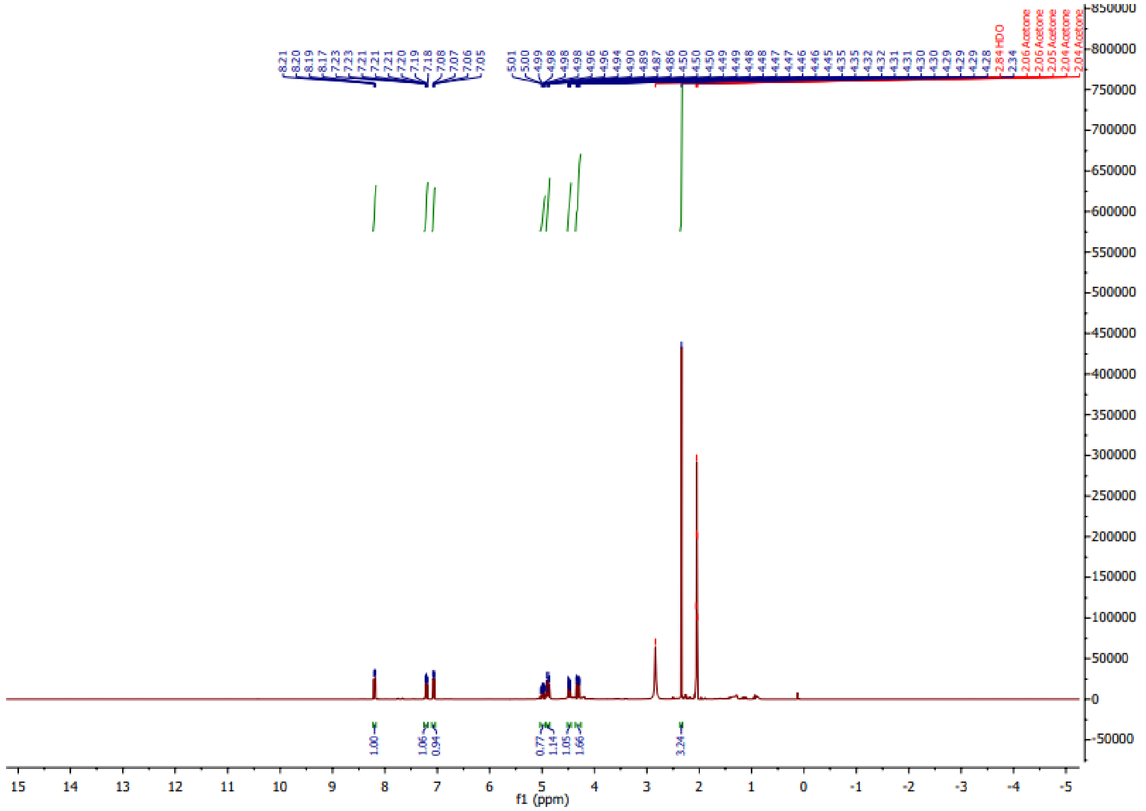

^{13}C NMR spectrum of 4-F-ASA-Prop-PtCl$_3$

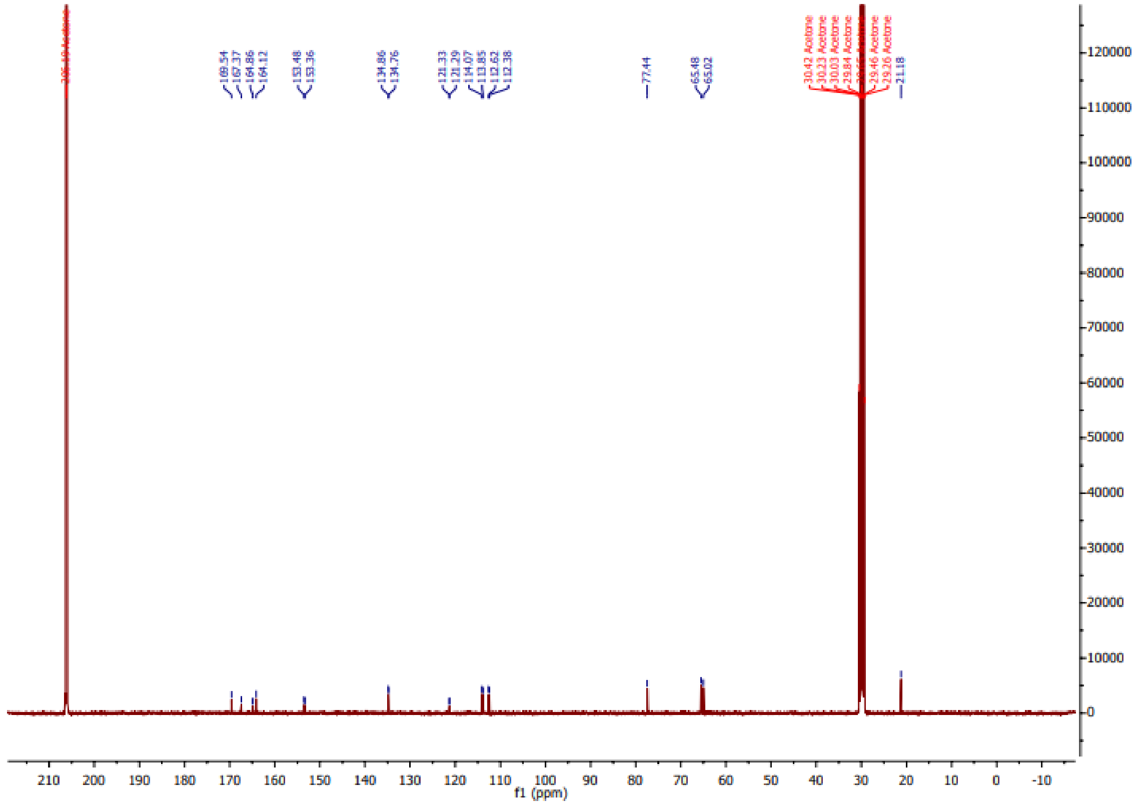

Supporting Figure S52: ^{1}H and ^{13}C NMR spectra of 4-F-ASA-But-$PtCl_3$

^{1}H NMR spectrum of 4-F-ASA-But-$PtCl_3$

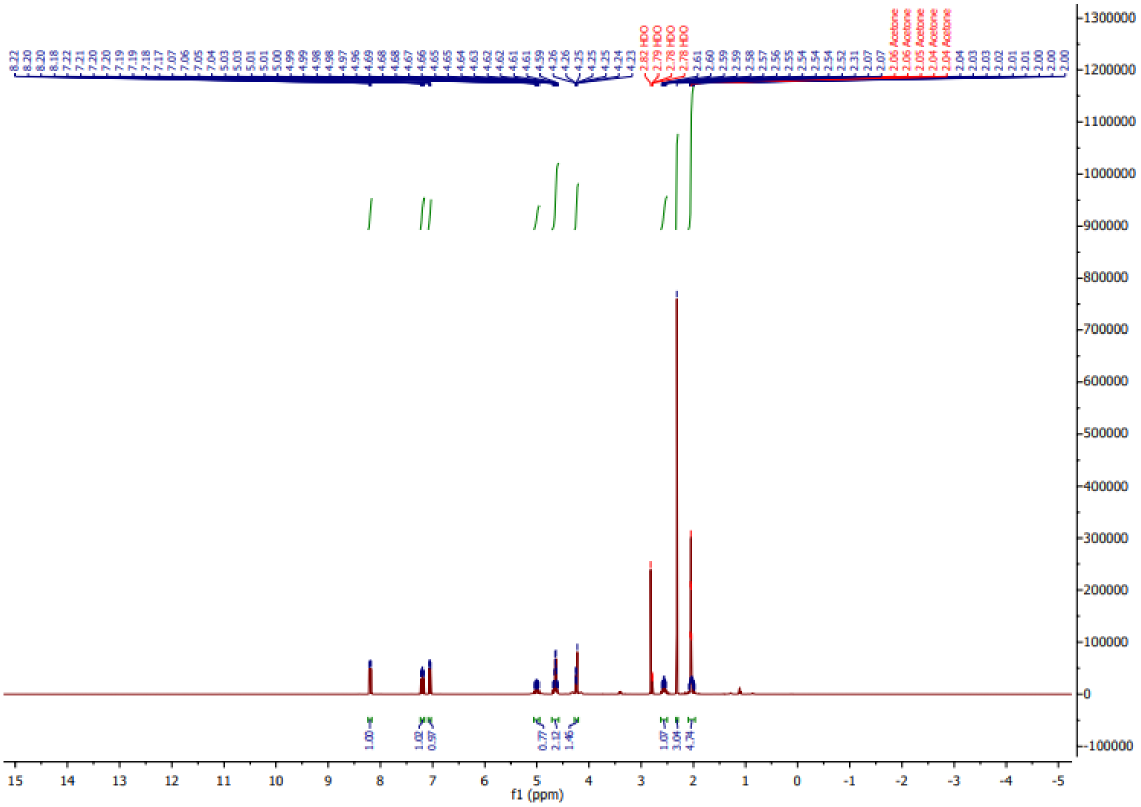

^{13}C NMR spectrum of 4-F-ASA-But-$PtCl_3$

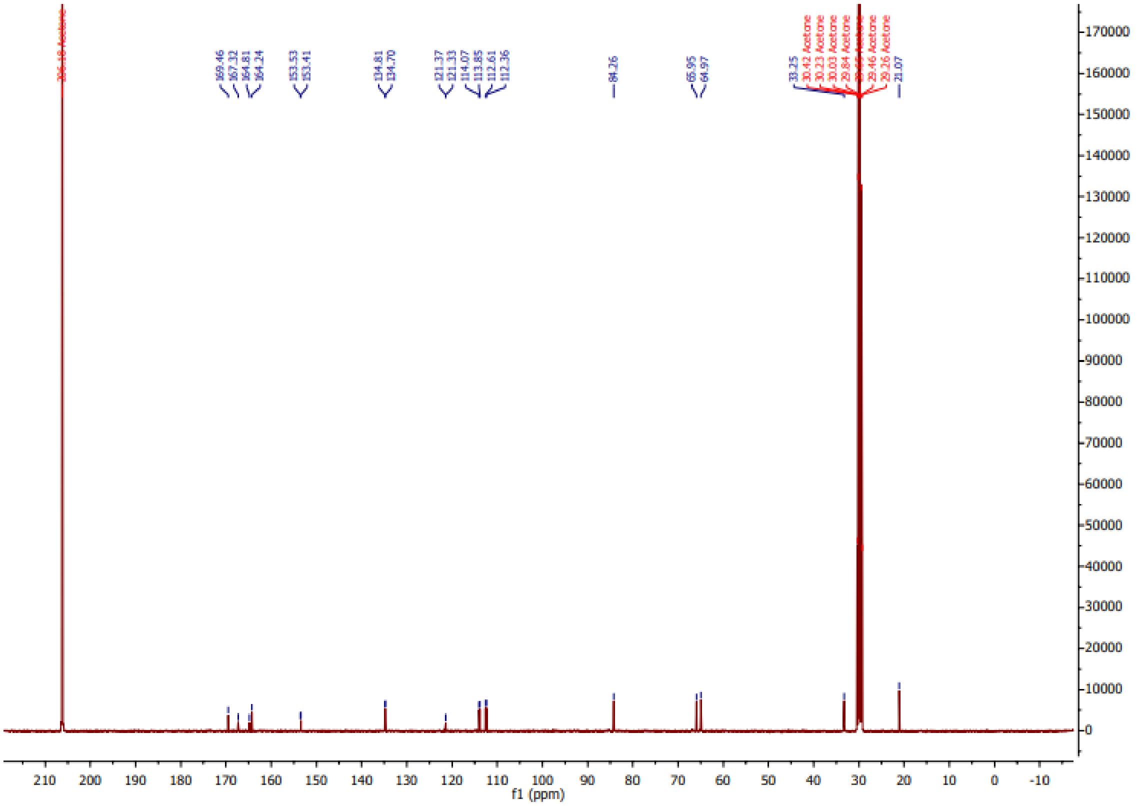

Supporting Figure S53: ^{1}H and ^{13}C NMR spectra of 5-F-ASA-Prop-$PtCl_3$

^{1}H NMR spectrum of 5-F-ASA-Prop-$PtCl_3$

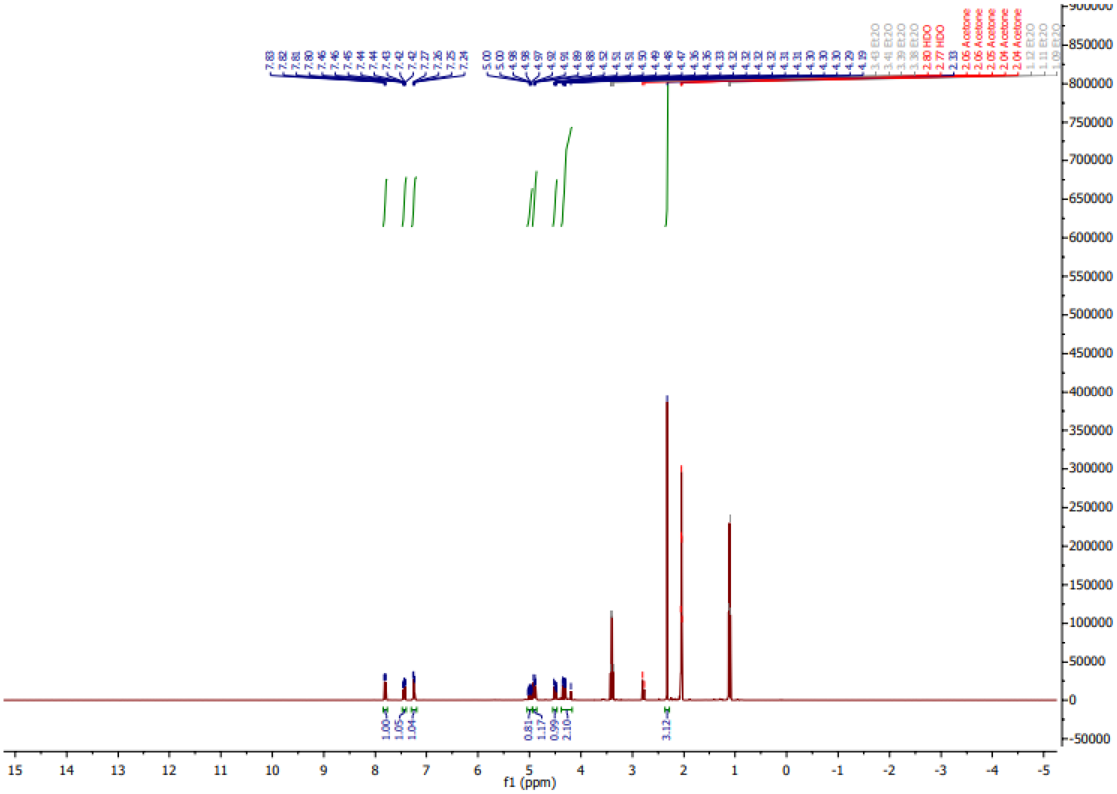

^{13}C NMR spectrum of 5-F-ASA-Prop-$PtCl_3$

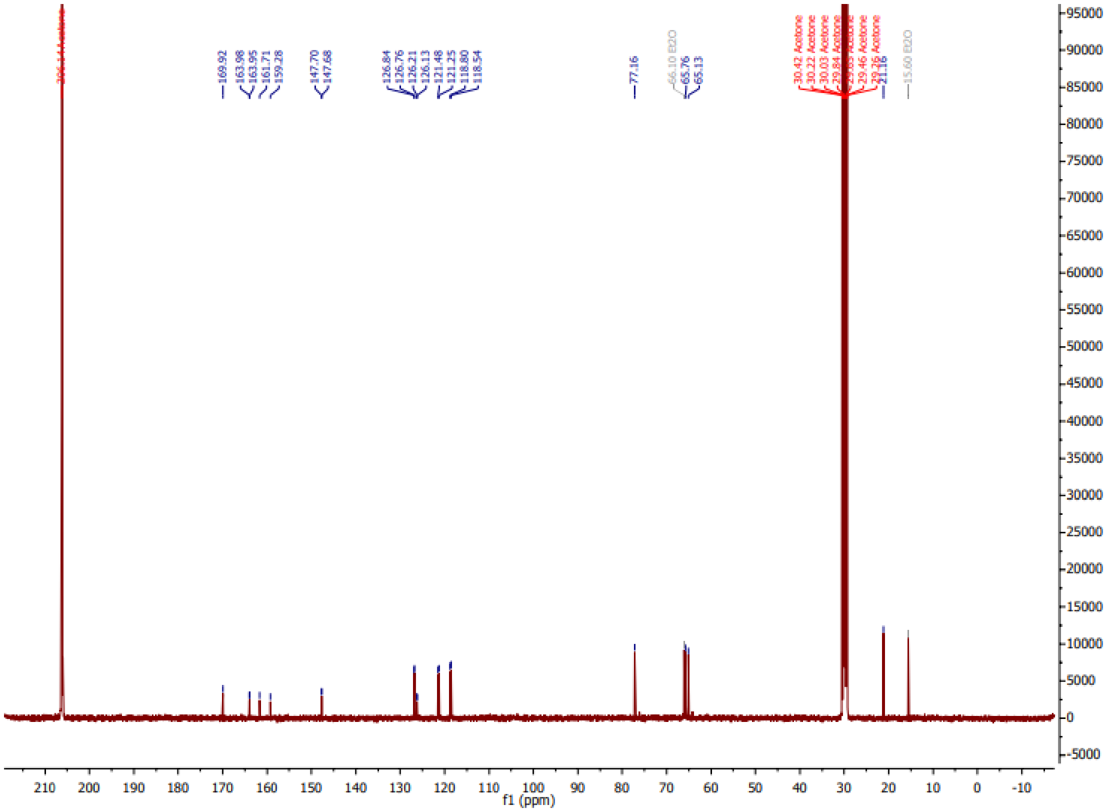

<u>Supporting Figure S54: ^{1}H and ^{13}C NMR spectra of 5-F-ASA-But-PtCl$_3$</u>

^{1}H NMR spectrum of 5-F-ASA-But-PtCl$_3$

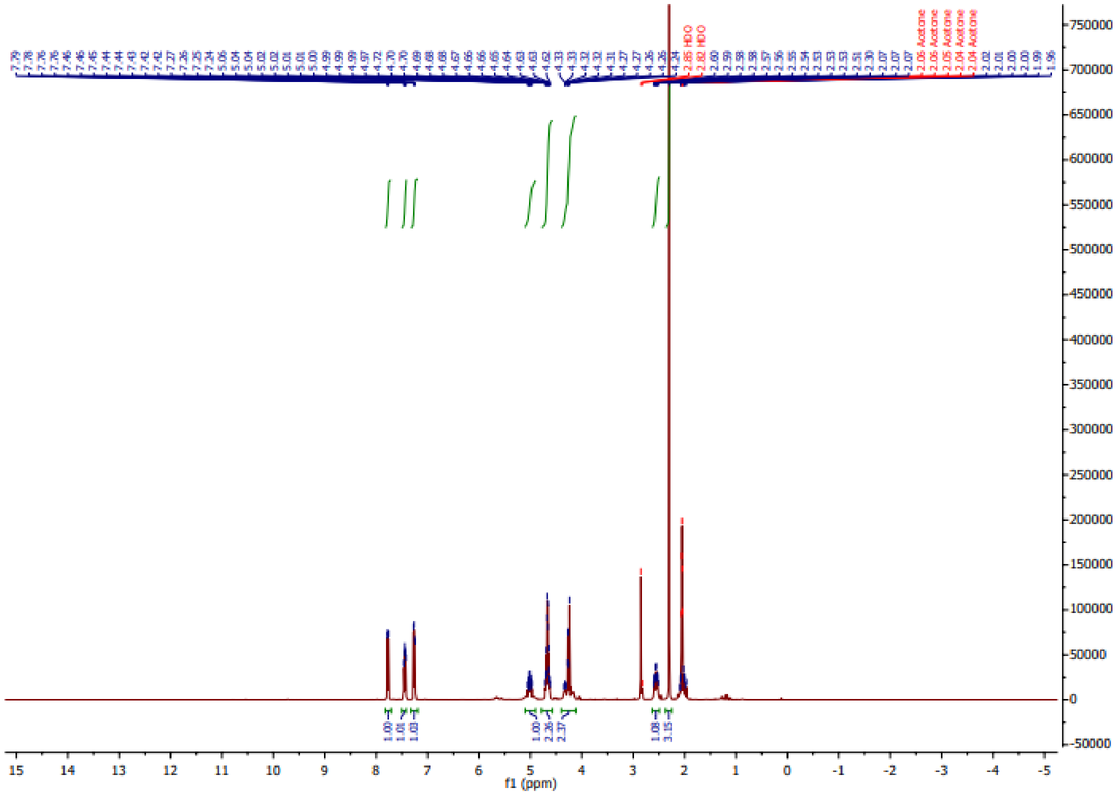

^{13}C NMR spectrum of 5-F-ASA-But-PtCl$_3$

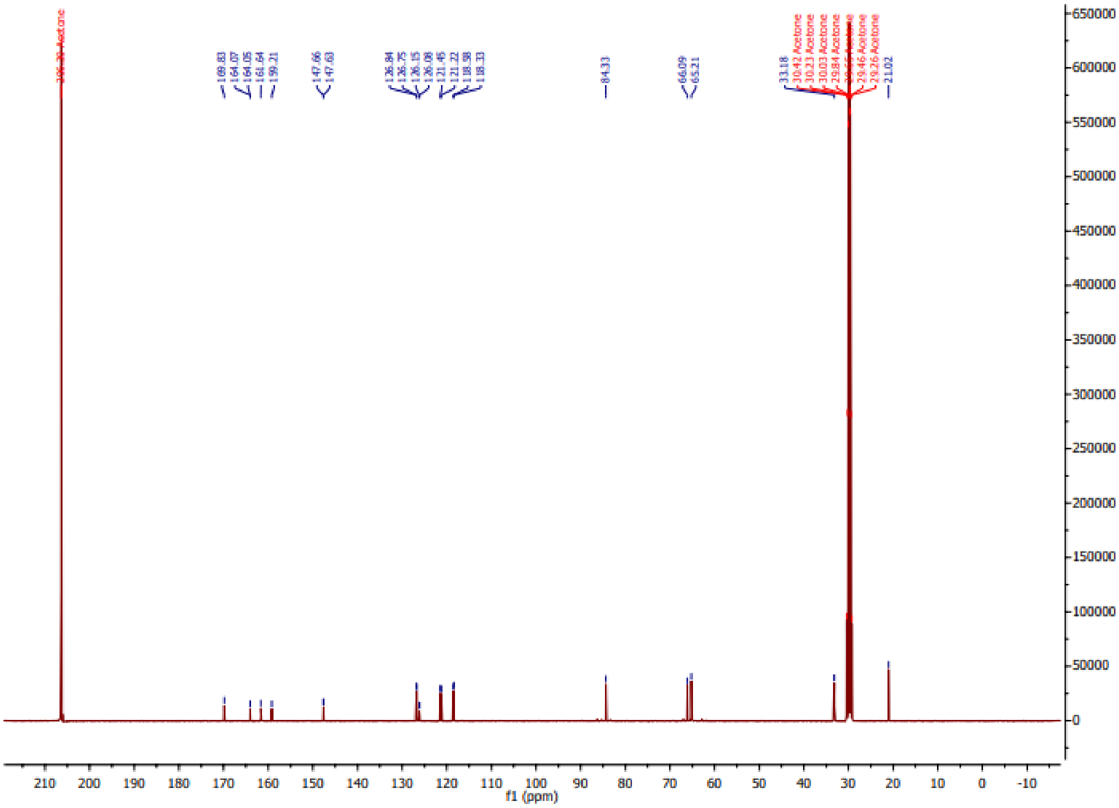

Supporting Figure S55: ^{1}H and ^{13}C NMR spectra of 6-F-ASA-Prop-PtCl$_3$

^{1}H NMR spectrum of 6-F-ASA-Prop-PtCl$_3$

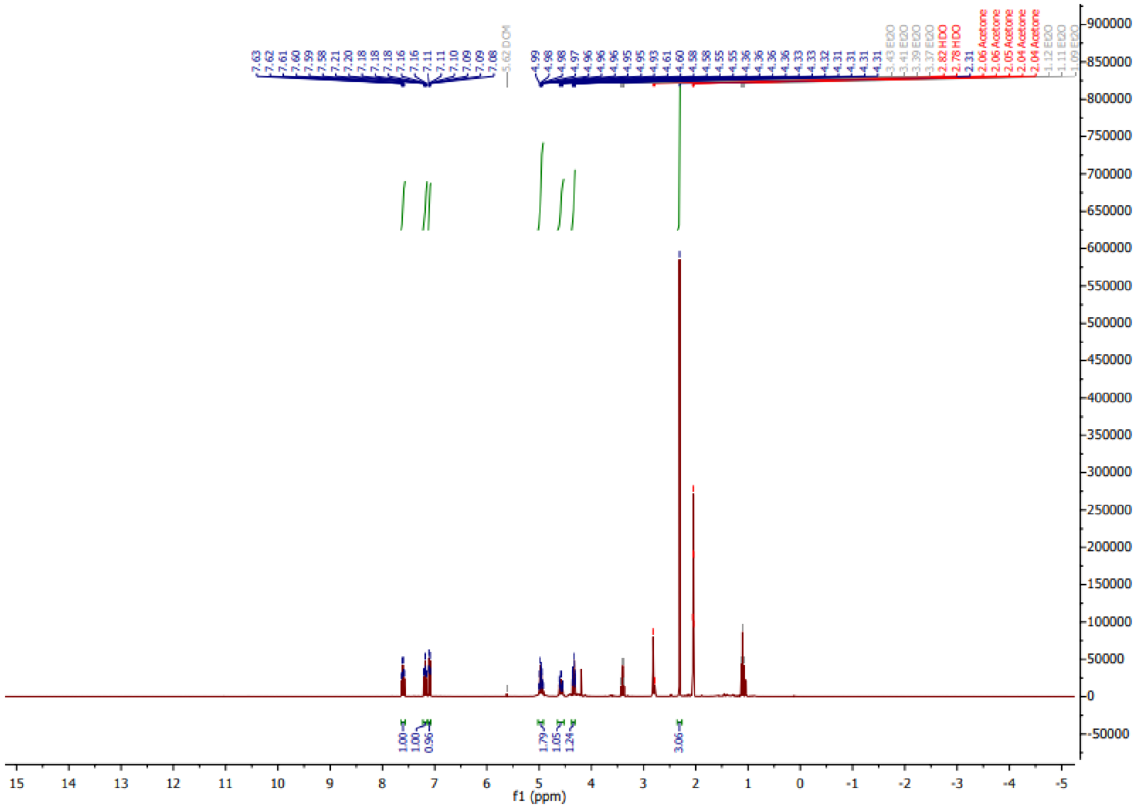

^{13}C NMR spectrum of 6-F-ASA-Prop-PtCl$_3$

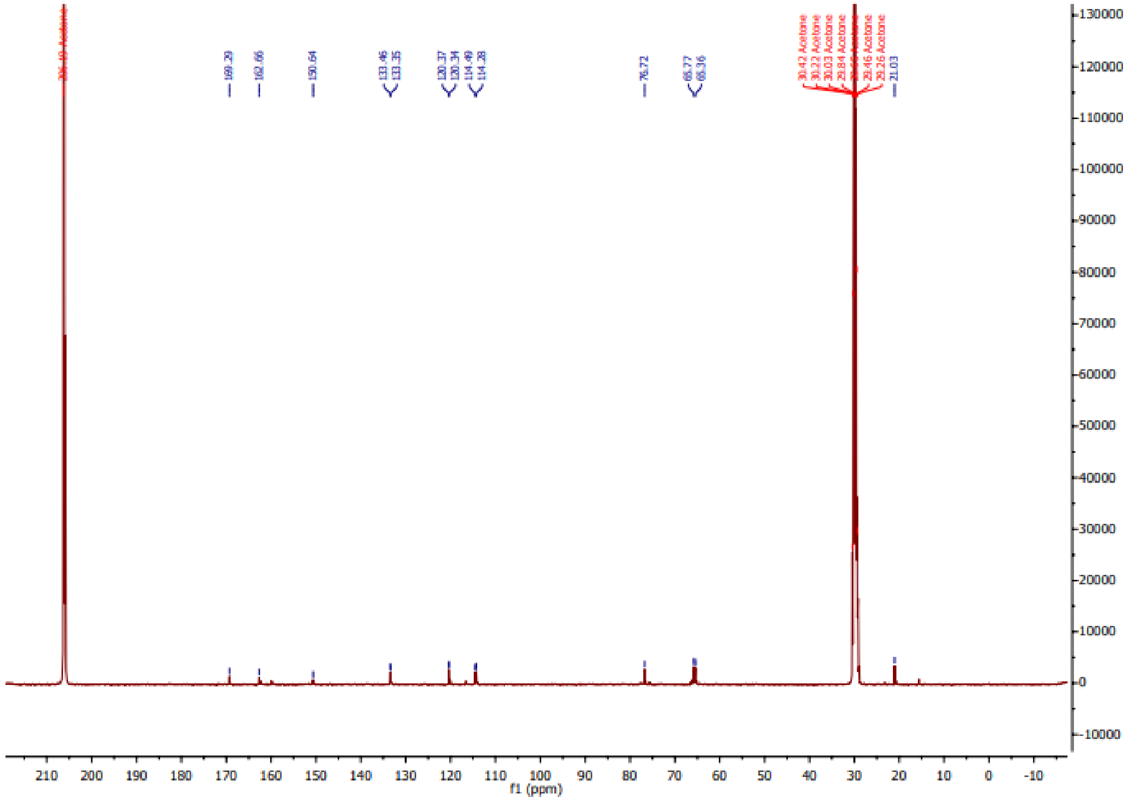

Supporting Figure S56: ^{1}H and ^{13}C NMR spectra of 6-F-ASA-But-PtCl$_3$

^{1}H NMR spectrum of 6-F-ASA-But-PtCl$_3$

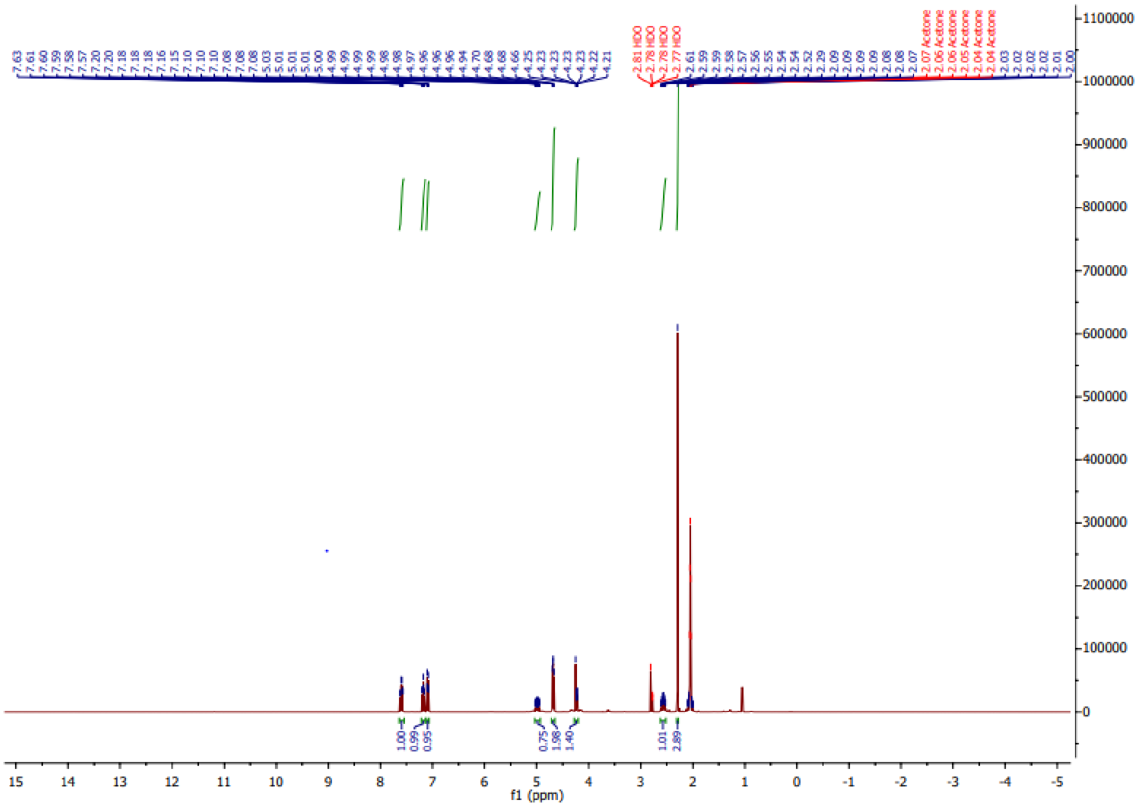

^{13}C NMR spectrum of 6-F-ASA-But-PtCl$_3$

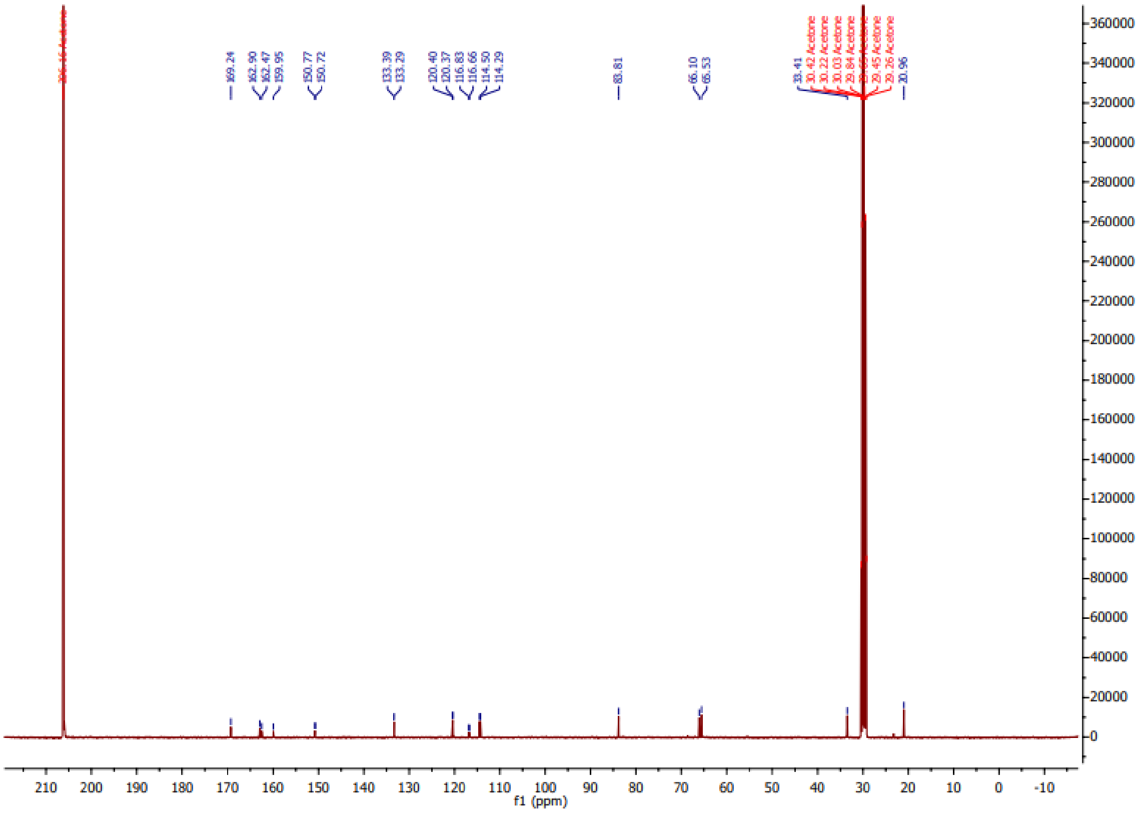

Supporting Figure S57: ^{1}H and ^{13}C NMR spectra of 3-Cl-ASA-Prop-$PtCl_3$

^{1}H NMR spectrum of 3-Cl-ASA-Prop-$PtCl_3$

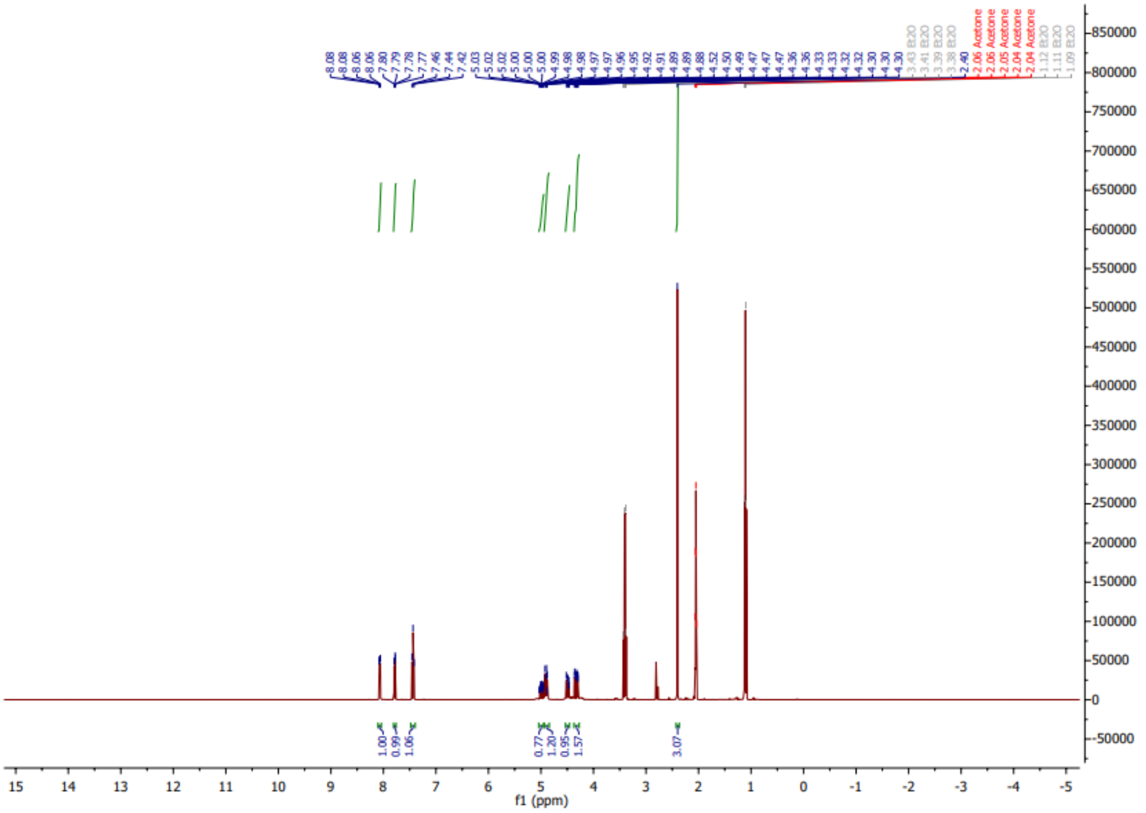

^{13}C NMR spectrum of 3-Cl-ASA-Prop-$PtCl_3$

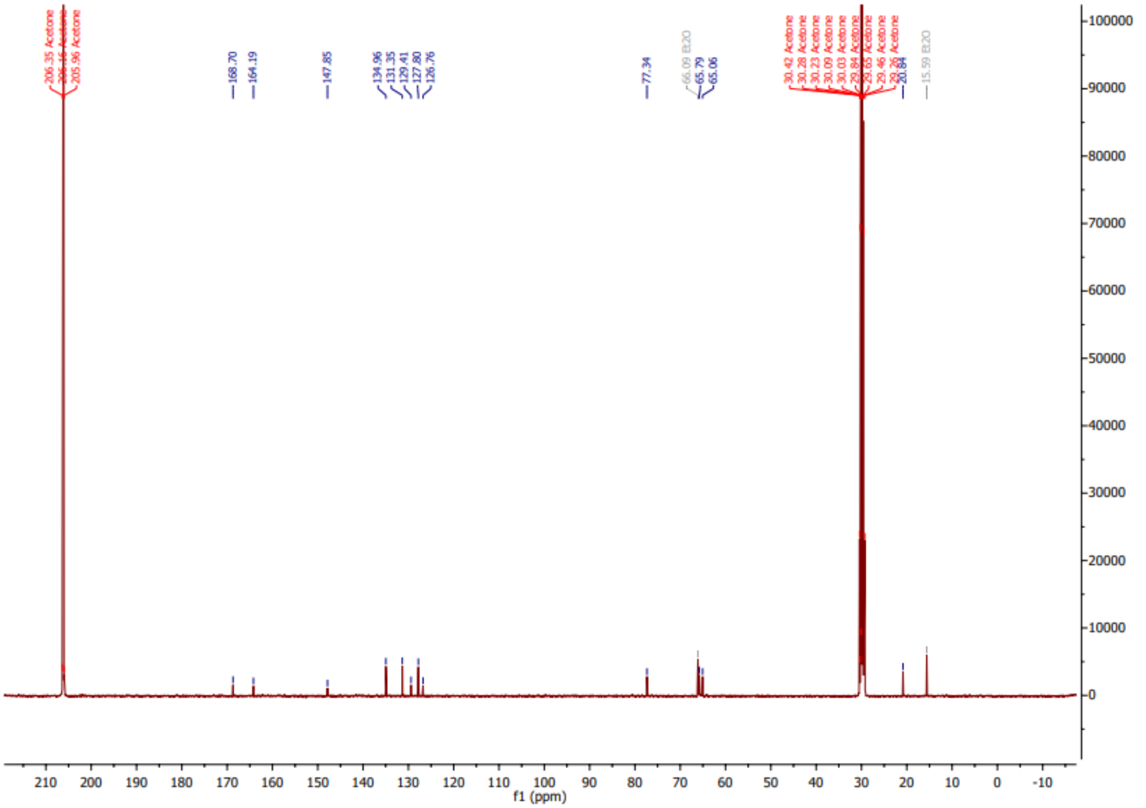

Supporting Figure S58: ^{1}H and ^{13}C NMR spectra of 3-Cl-ASA-But-PtCl$_3$

^{1}H NMR spectrum of 3-Cl-ASA-But-PtCl$_3$

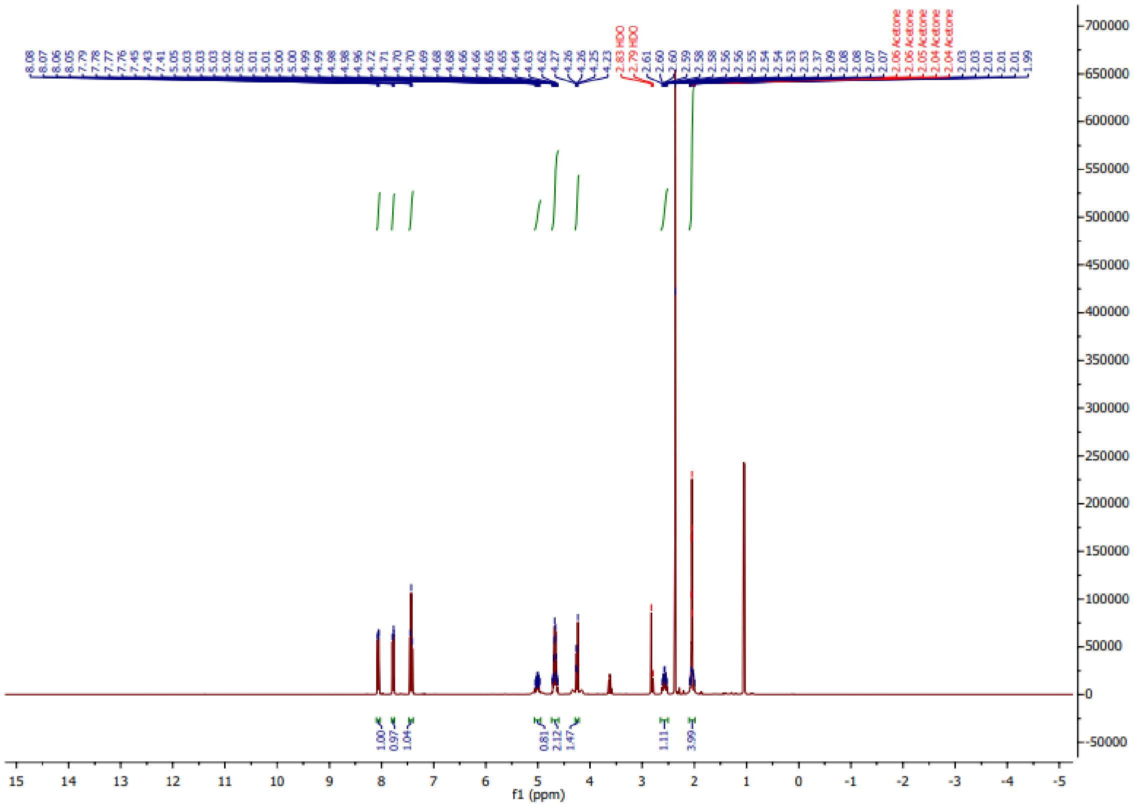

^{13}C NMR spectrum of 3-Cl-ASA-But-PtCl$_3$

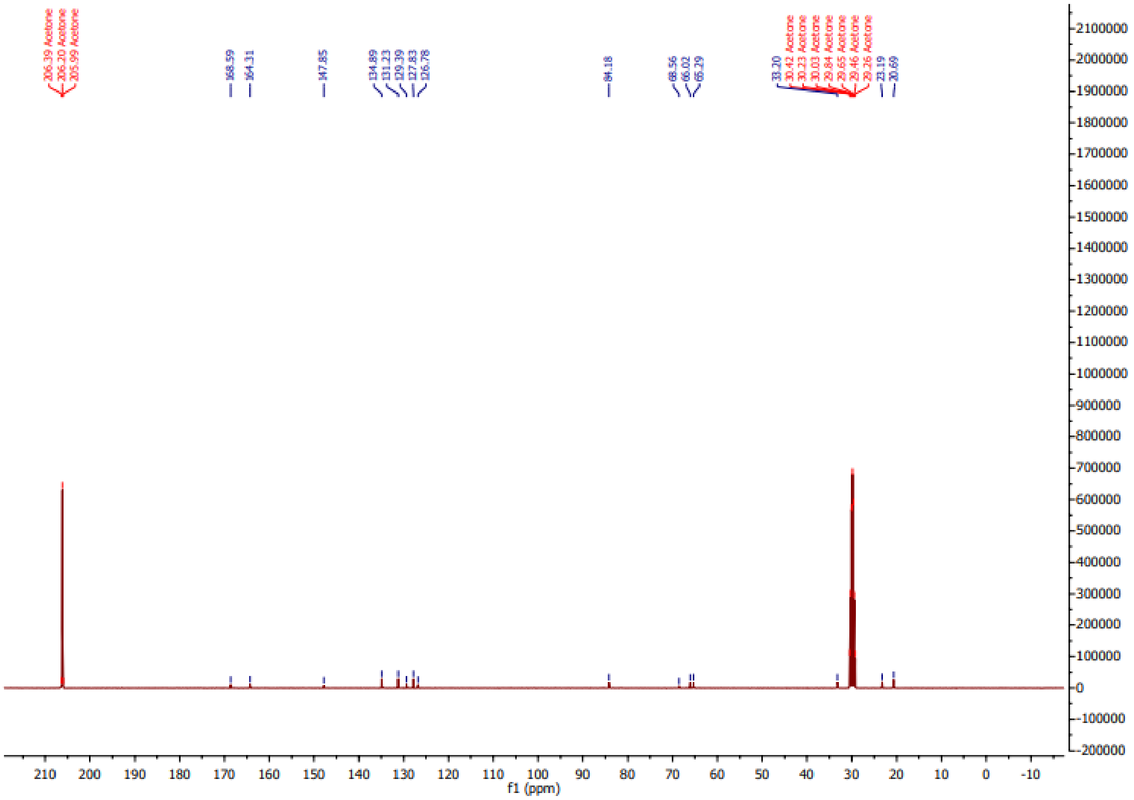

Supporting Figure S59: ^{1}H and ^{13}C NMR spectra of 4-Cl-ASA-Prop-PtCl$_3$

^{1}H NMR spectrum of 4-Cl-ASA-Prop-PtCl$_3$

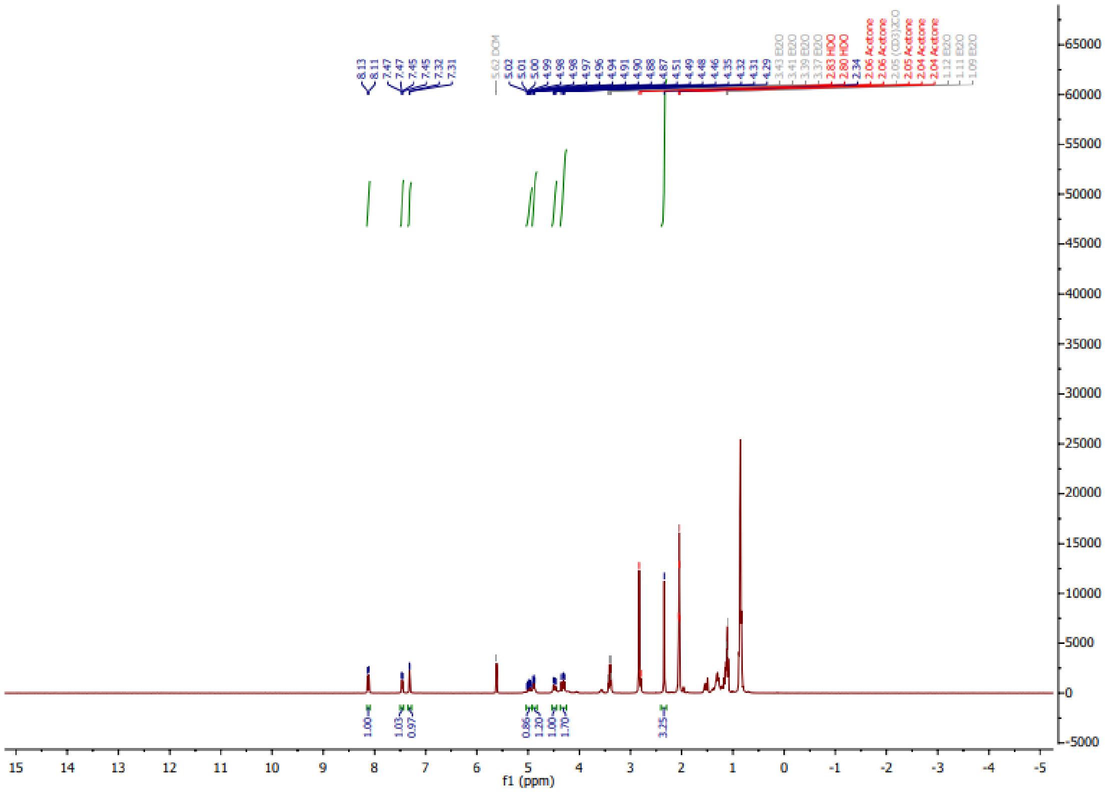

^{13}C NMR spectrum of 4-Cl-ASA-Prop-PtCl$_3$

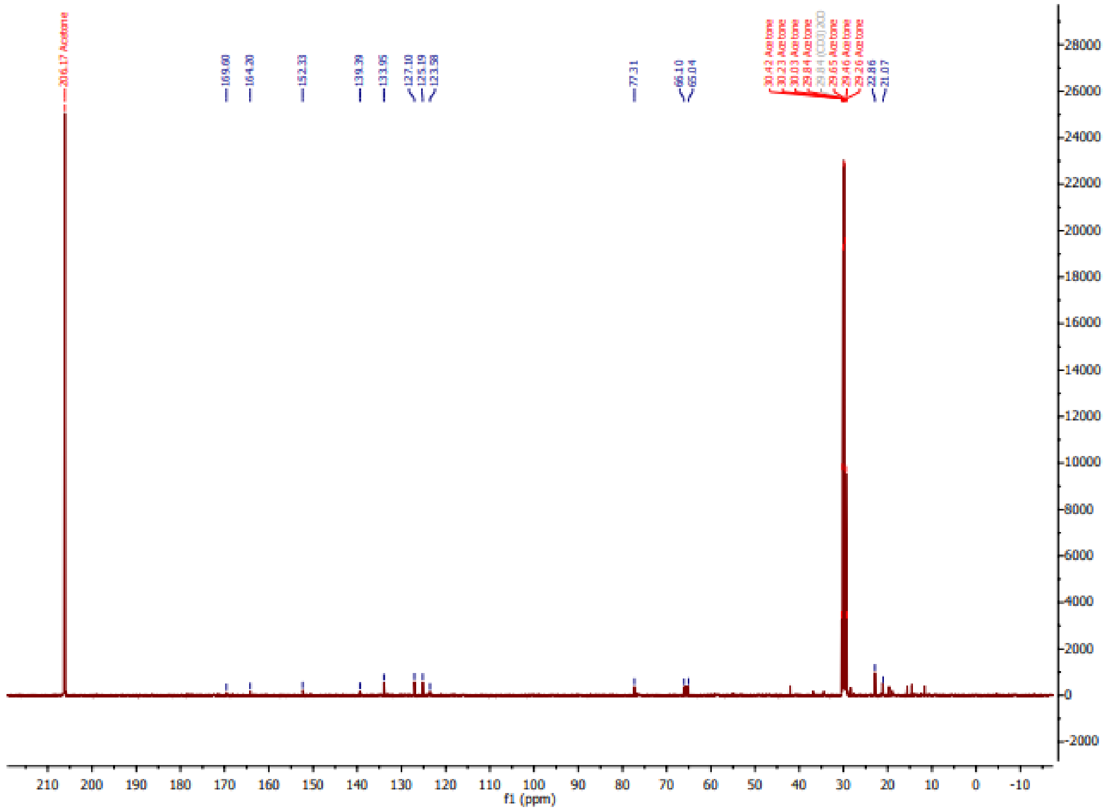

<u>Supporting Figure S60: ^{1}H and ^{13}C NMR spectra of 4-Cl-ASA-But-PtCl$_3$</u>

^{1}H NMR spectrum of 4-Cl-ASA-But-PtCl$_3$

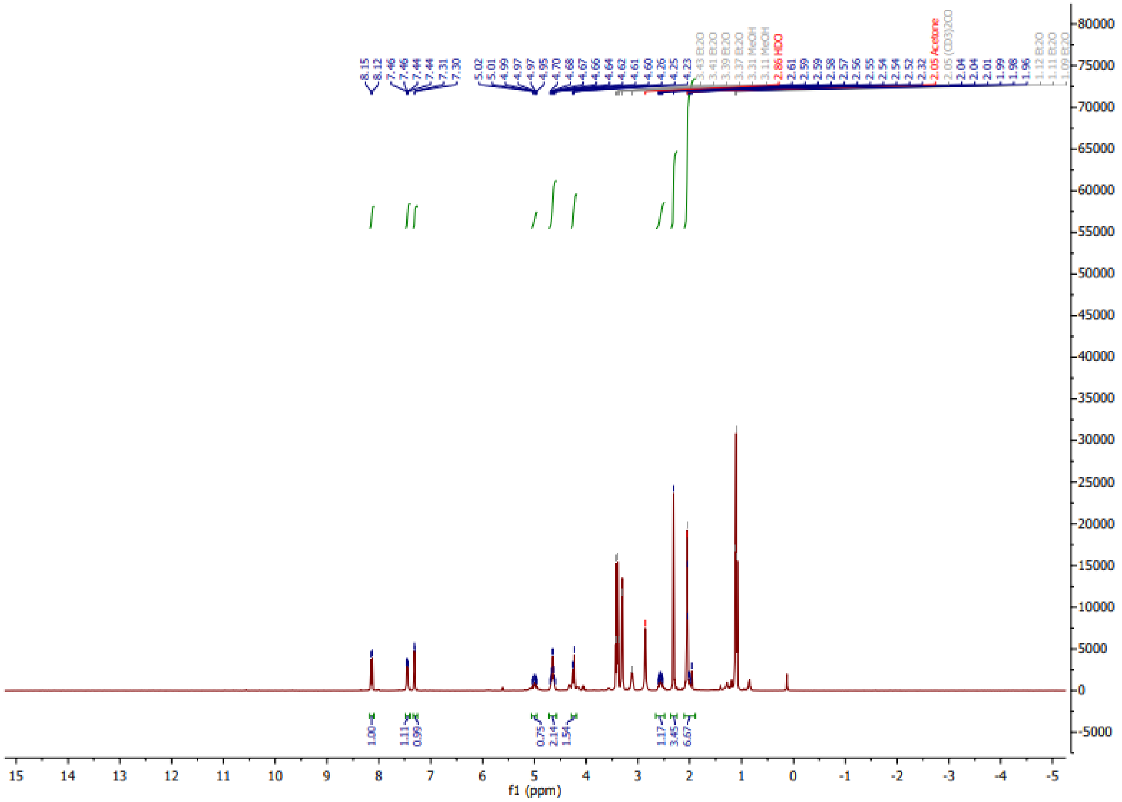

^{13}C NMR spectrum of 4-Cl-ASA-But-PtCl$_3$

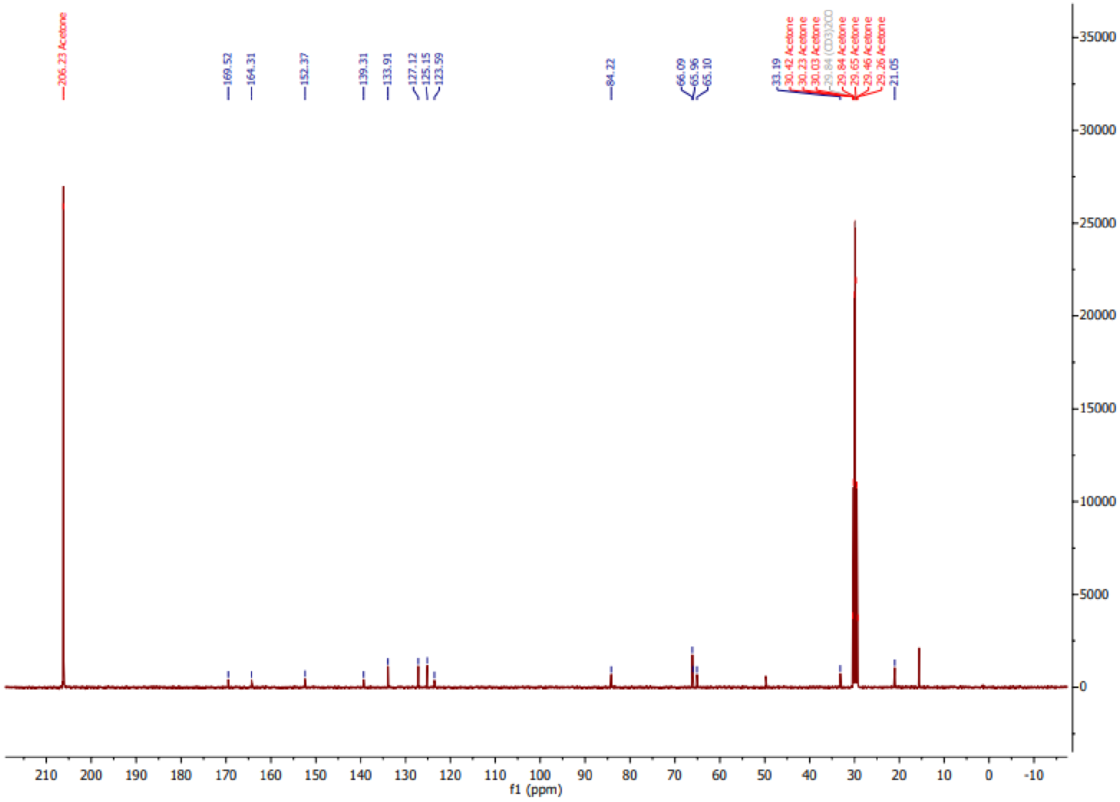

Supporting Figure S61: ^{1}H and ^{13}C NMR spectra of 5-Cl-ASA-Prop-$PtCl_3$

^{1}H NMR spectrum of 5-Cl-ASA-Prop-$PtCl_3$

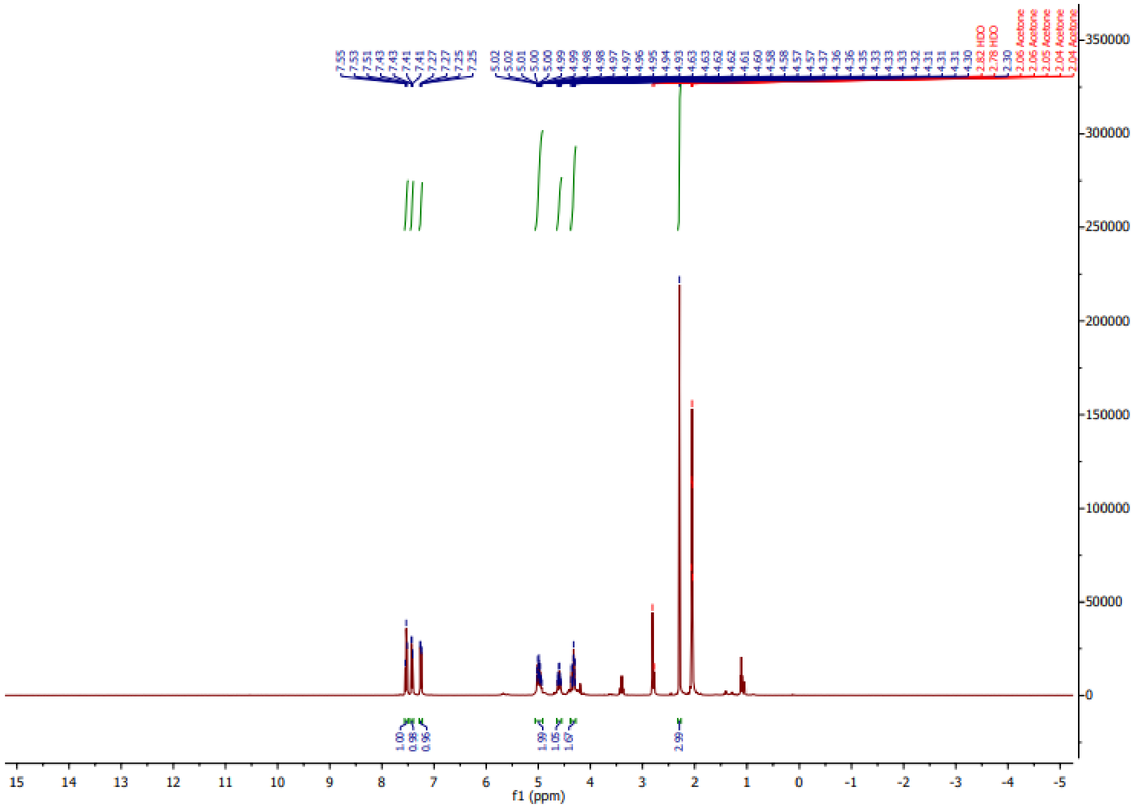

^{13}C NMR spectrum of 5-Cl-ASA-Prop-$PtCl_3$

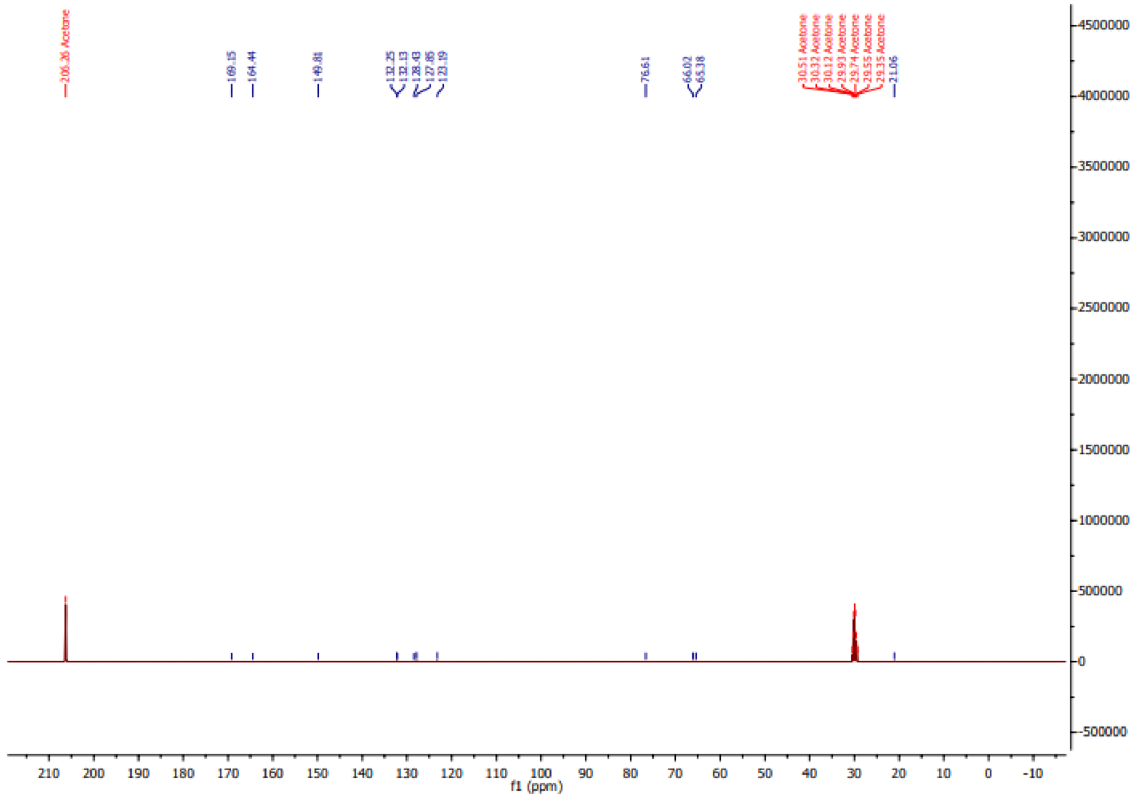

Supporting Figure S62: ^{1}H and ^{13}C NMR spectra of 5-Cl-ASA-But-$PtCl_3$

^{1}H NMR spectrum of 5-Cl-ASA-But-$PtCl_3$

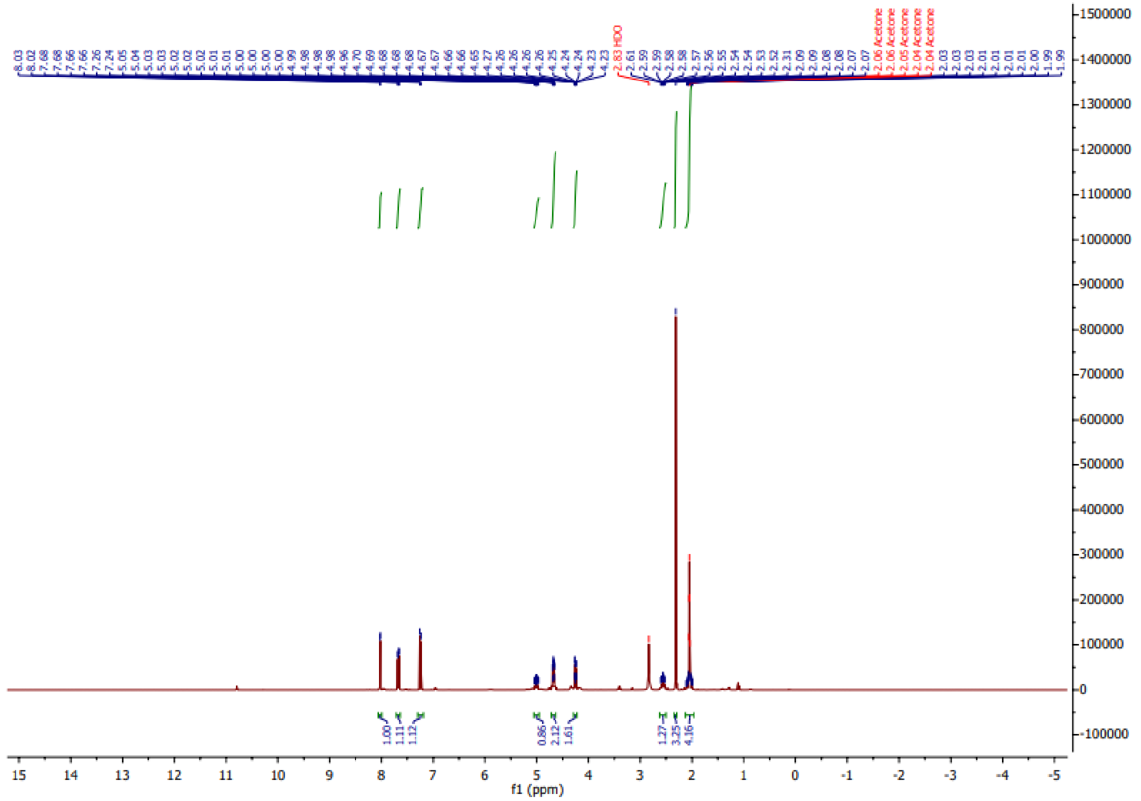

^{13}C NMR spectrum of 5-Cl-ASA-But-$PtCl_3$

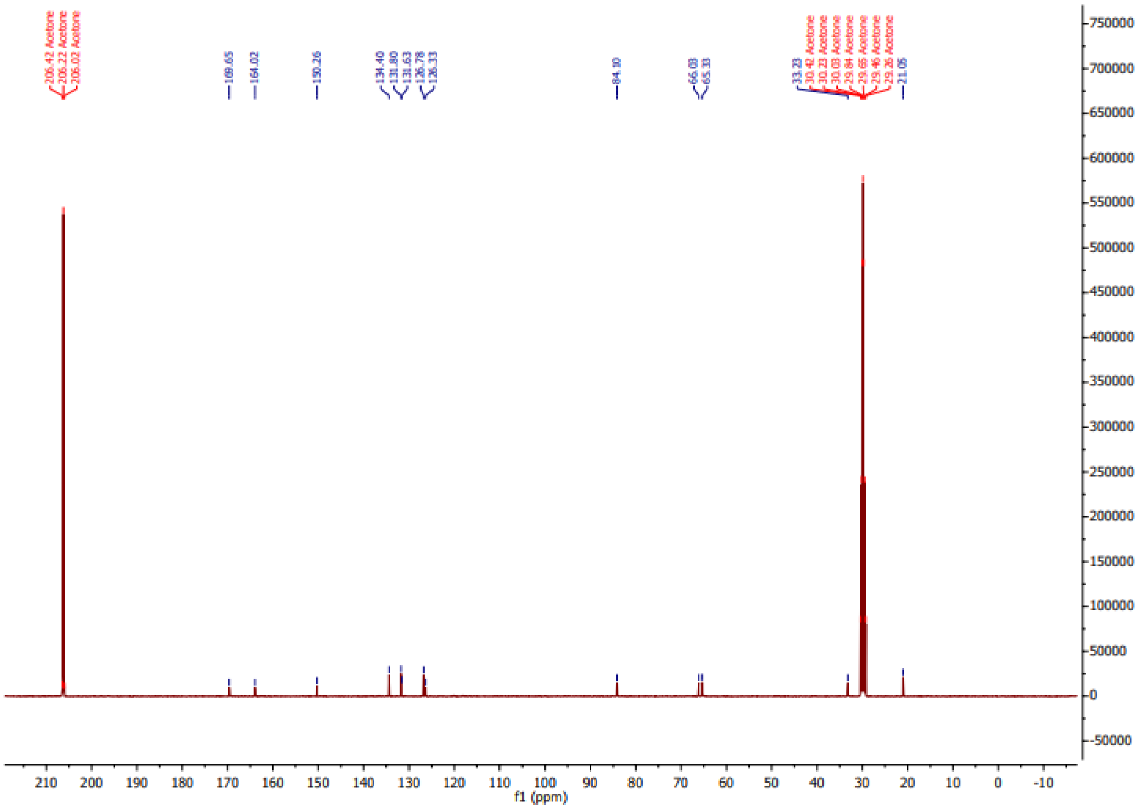

Supporting Figure S63: ^{1}H and ^{13}C NMR spectra of 6-Cl-ASA-Prop-PtCl$_3$

^{1}H NMR spectrum of 6-Cl-ASA-Prop-PtCl$_3$

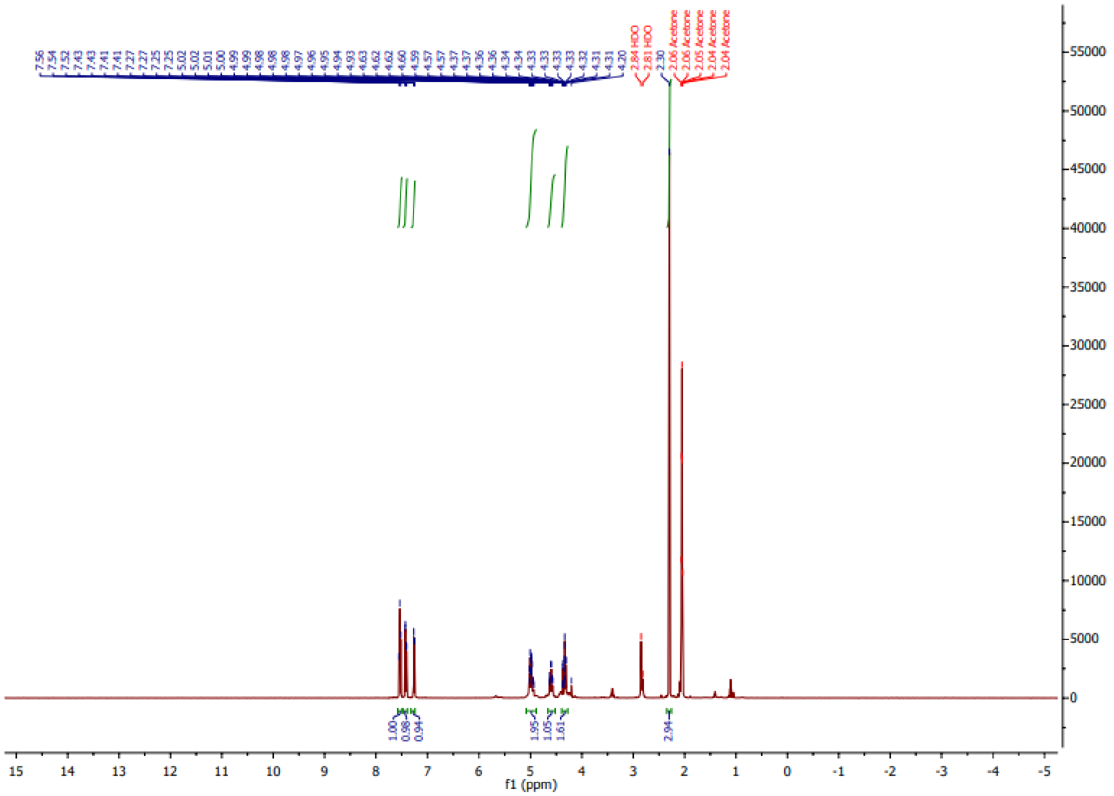

^{13}C NMR spectrum of 6-Cl-ASA-Prop-PtCl$_3$

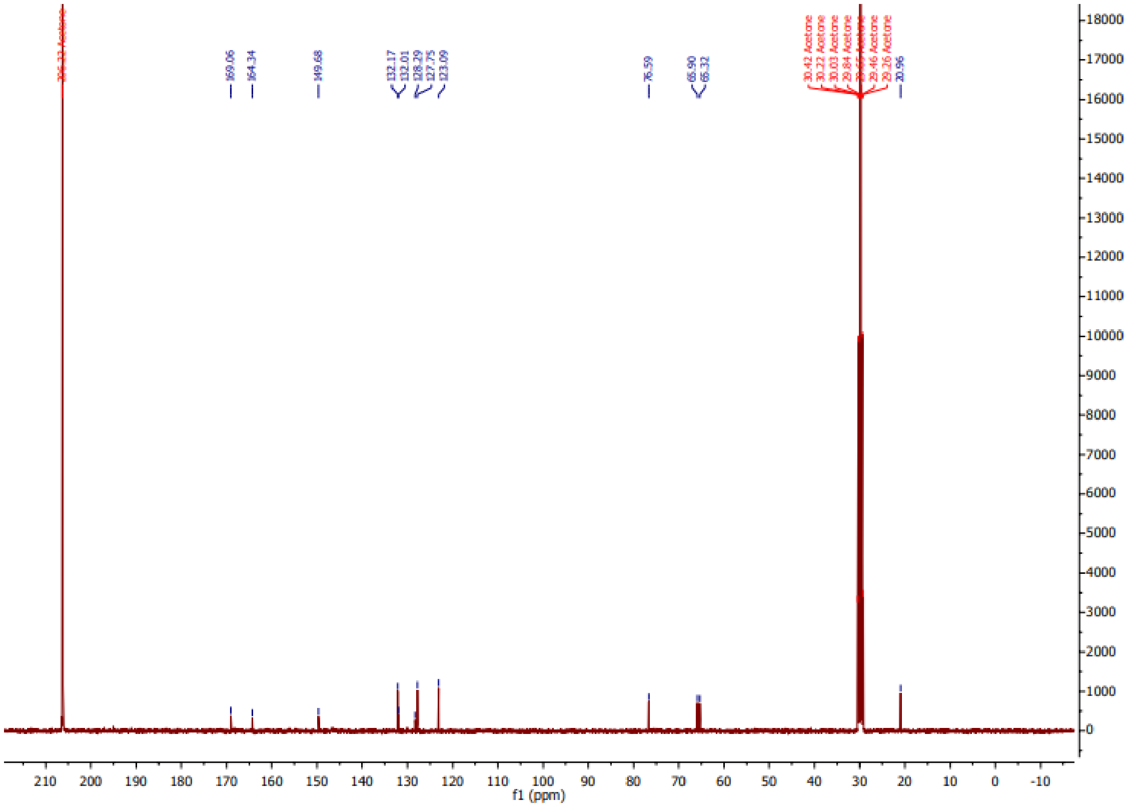

Supporting Figure S64: ^{1}H and ^{13}C NMR spectra of 6-Cl-ASA-But-PtCl$_3$

^{1}H NMR spectrum of 6-Cl-ASA-But-PtCl$_3$

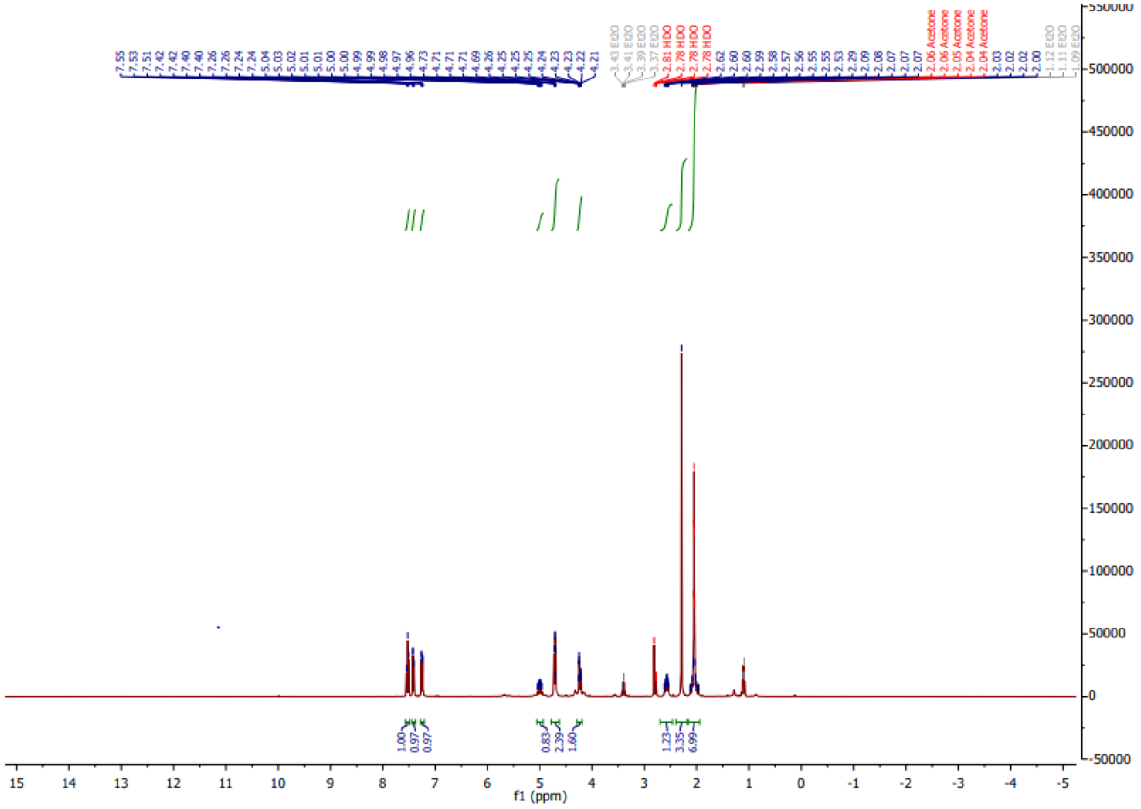

^{13}C NMR spectrum of 6-Cl-ASA-But-PtCl$_3$

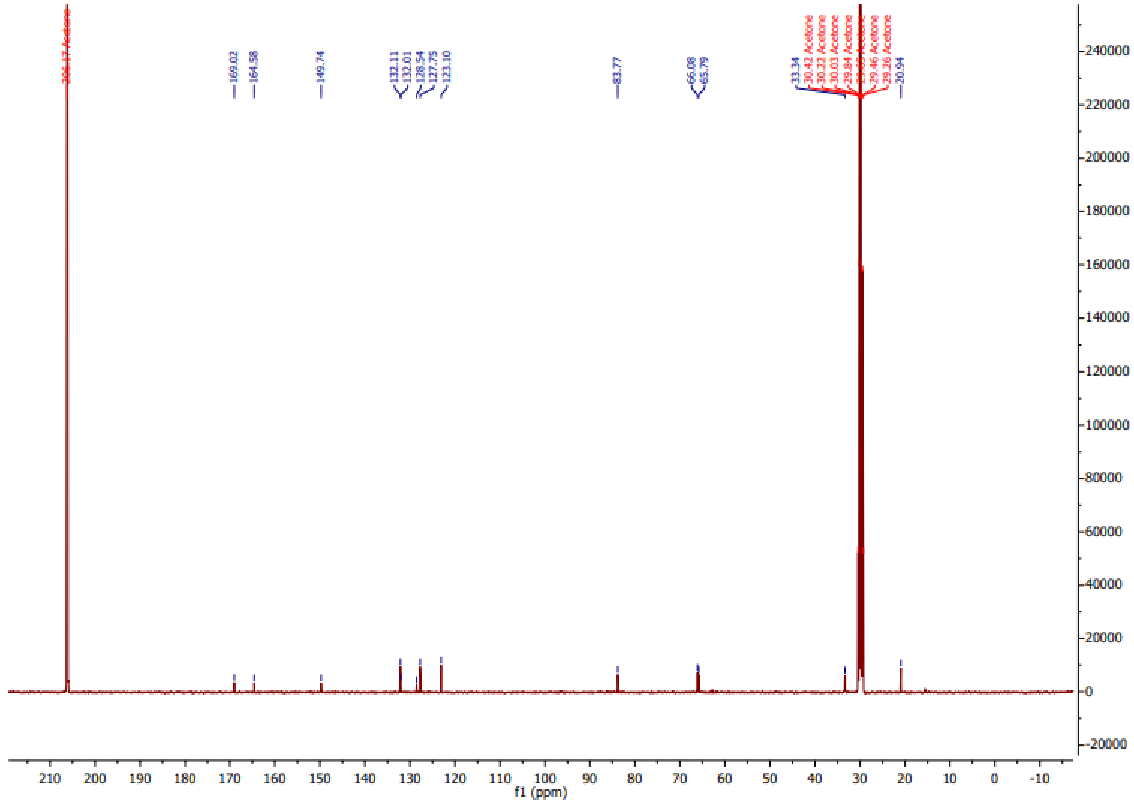

Supporting Figure S65: ^{1}H and ^{13}C NMR spectra of 3-CH_3-ASA-Prop-$PtCl_3$

^{1}H NMR spectrum of 3-CH_3-ASA-Prop-$PtCl_3$

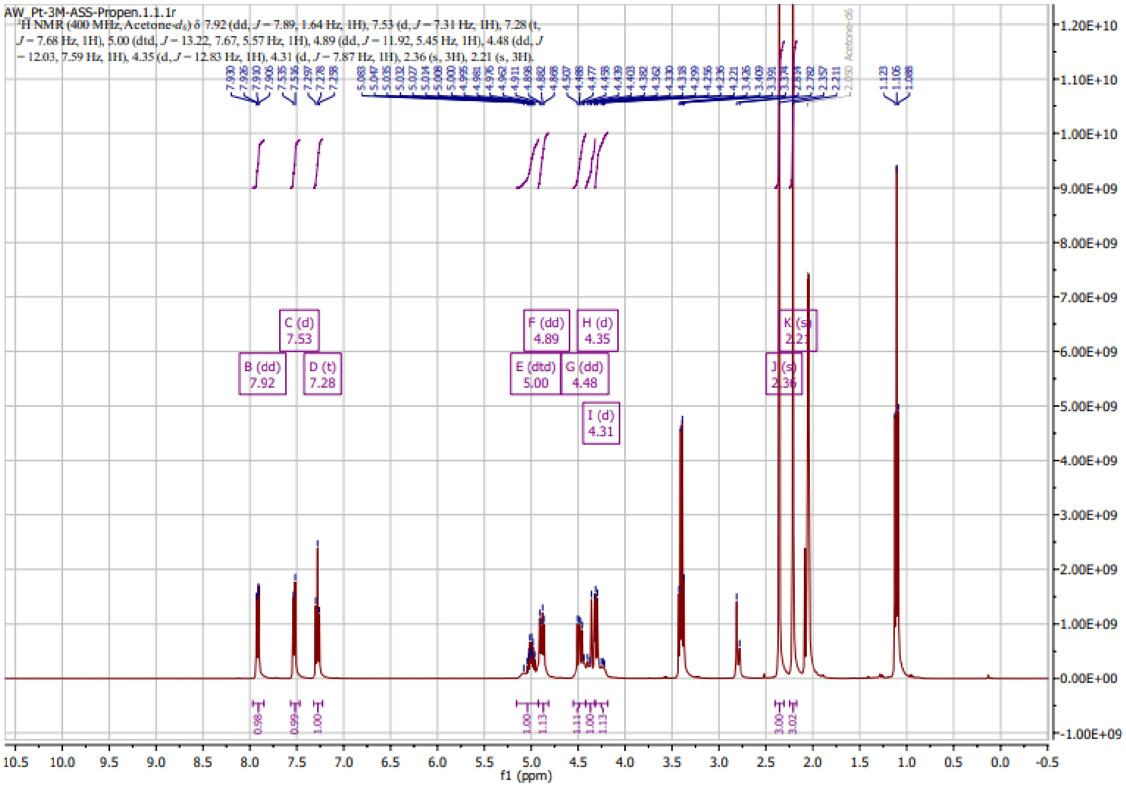

^{13}C NMR spectrum of 3-CH_3-ASA-Prop-$PtCl_3$

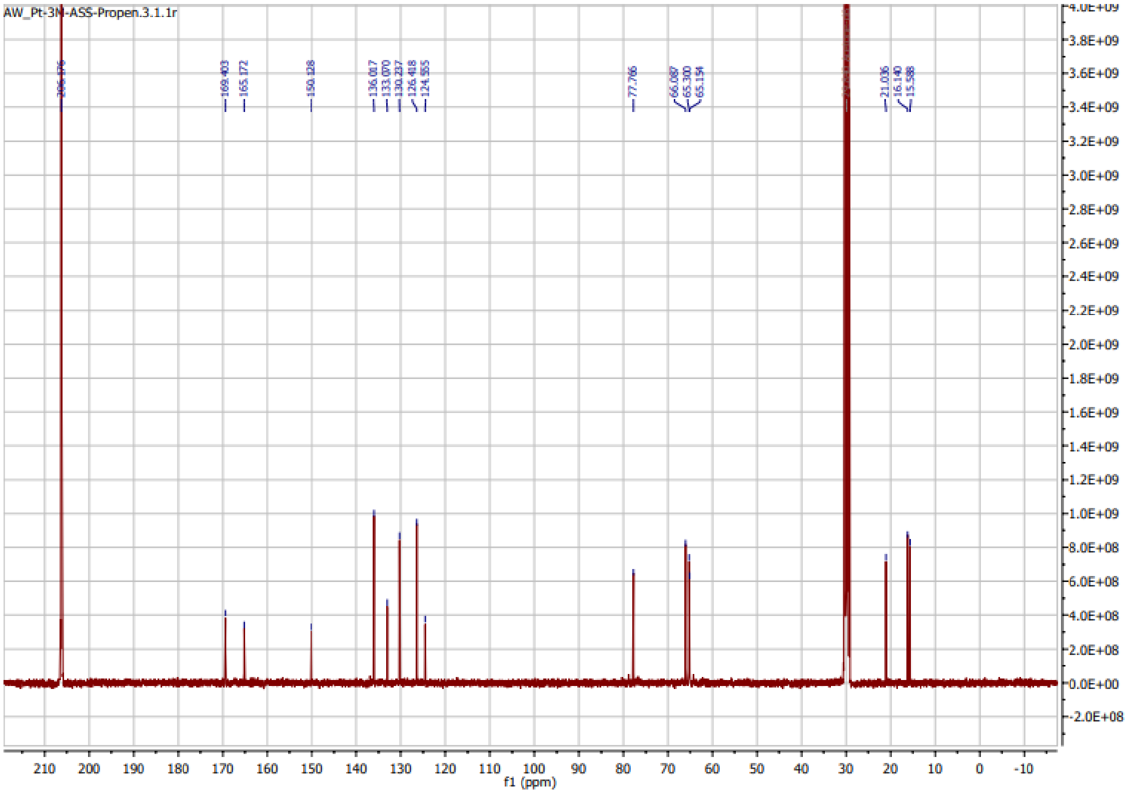

Supporting Figure S66: ^{1}H and ^{13}C NMR spectra of 3-CH$_3$-ASA-But-PtCl$_3$

^{1}H NMR spectrum of 3-CH$_3$-ASA-But-PtCl$_3$

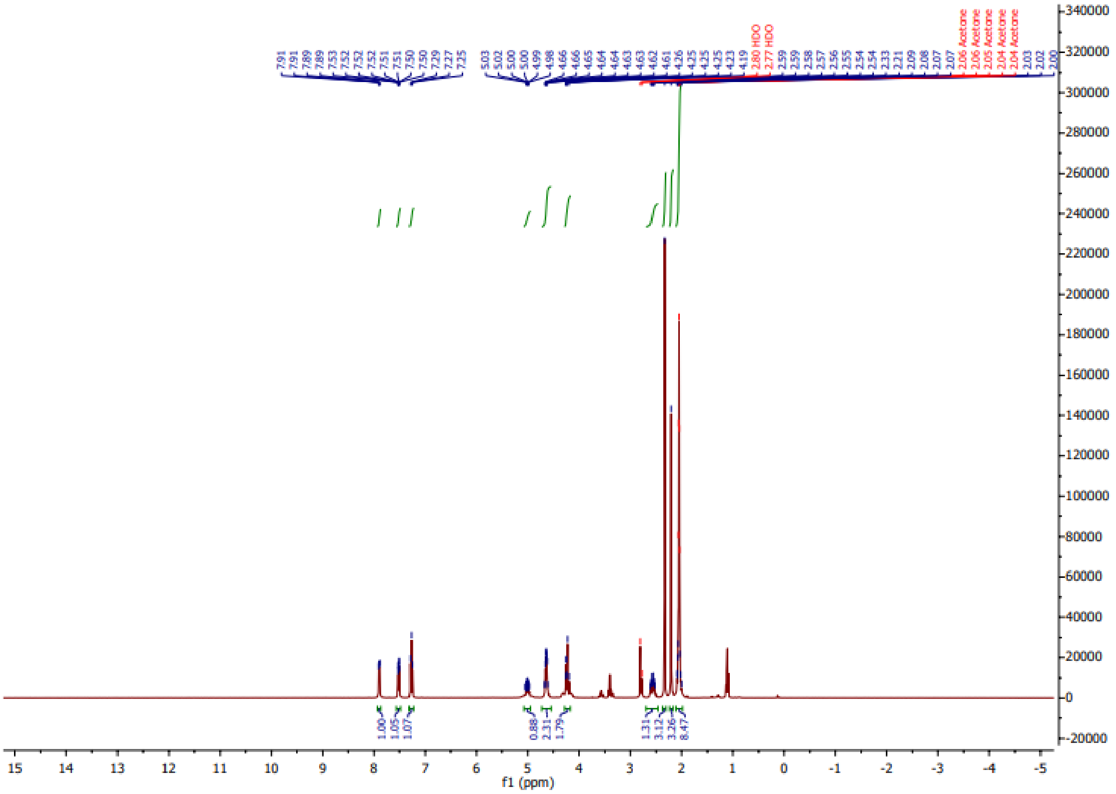

^{13}C NMR spectrum of 3-CH$_3$-ASA-But-PtCl$_3$

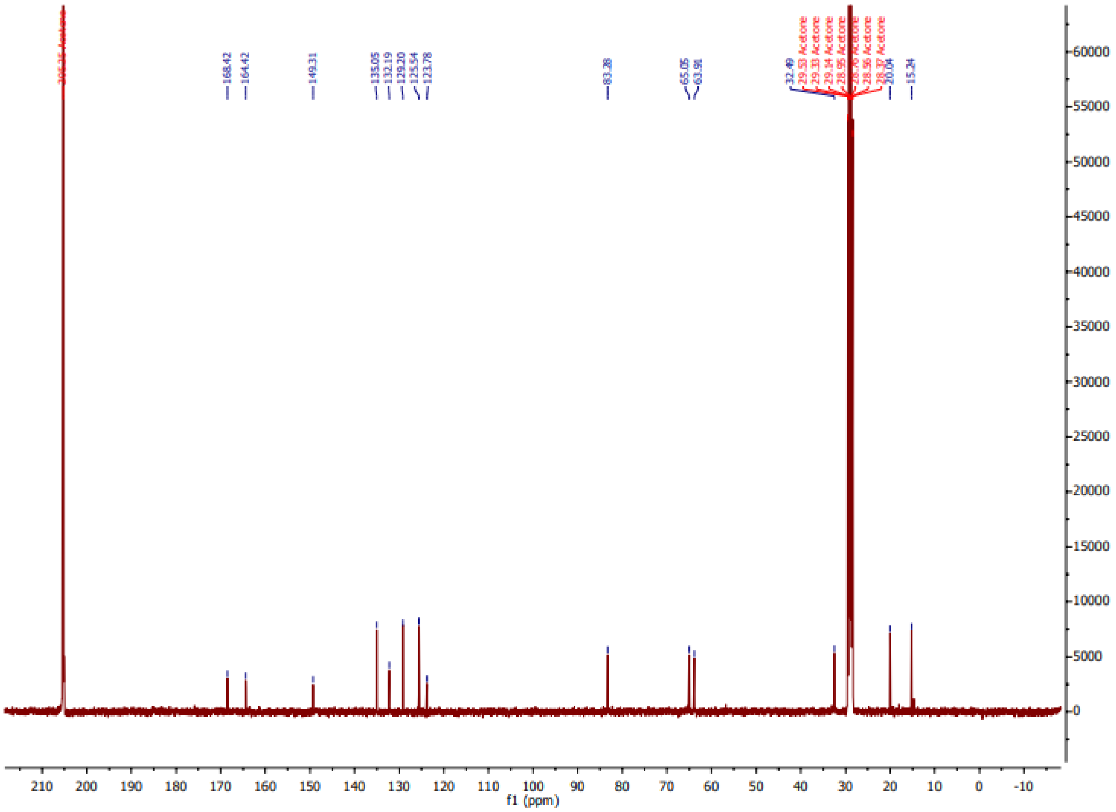

Supporting Figure S67: ^{1}H and ^{13}C NMR spectra of 4-CH_3-ASA-Prop-$PtCl_3$

^{1}H NMR spectrum of 4-CH_3-ASA-Prop-$PtCl_3$

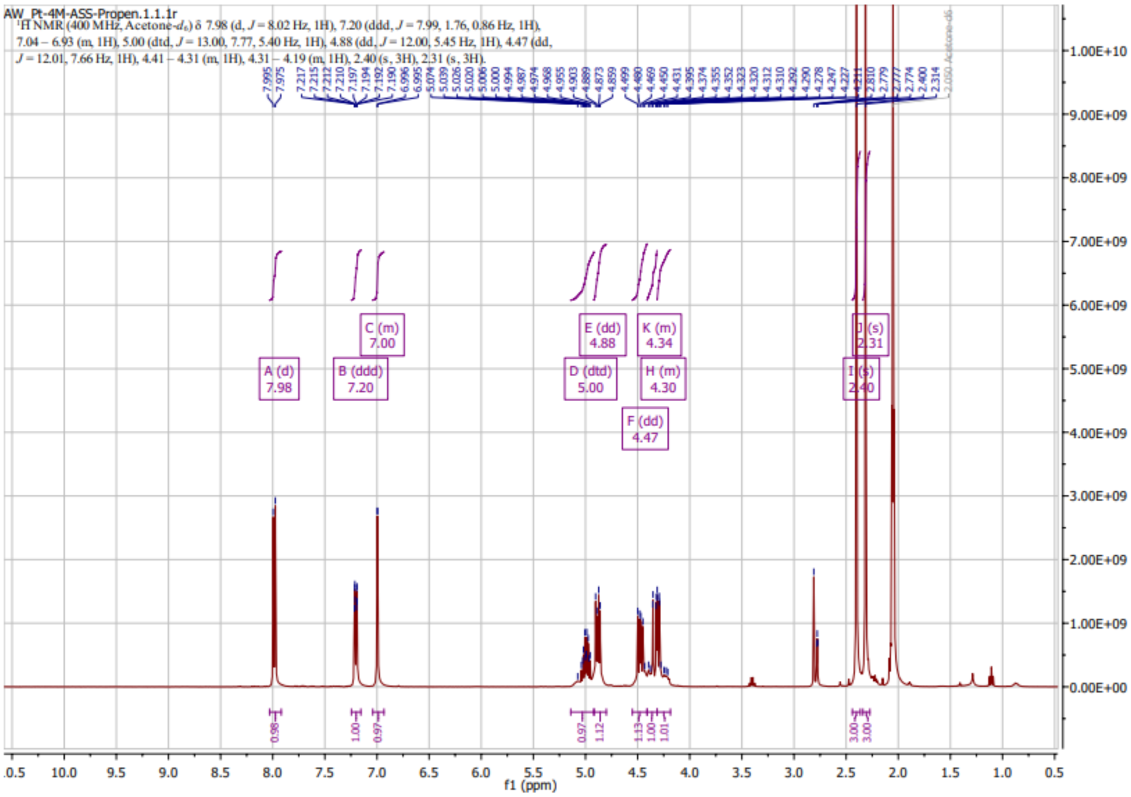

^{13}C NMR spectrum of 4-CH_3-ASA-Prop-$PtCl_3$

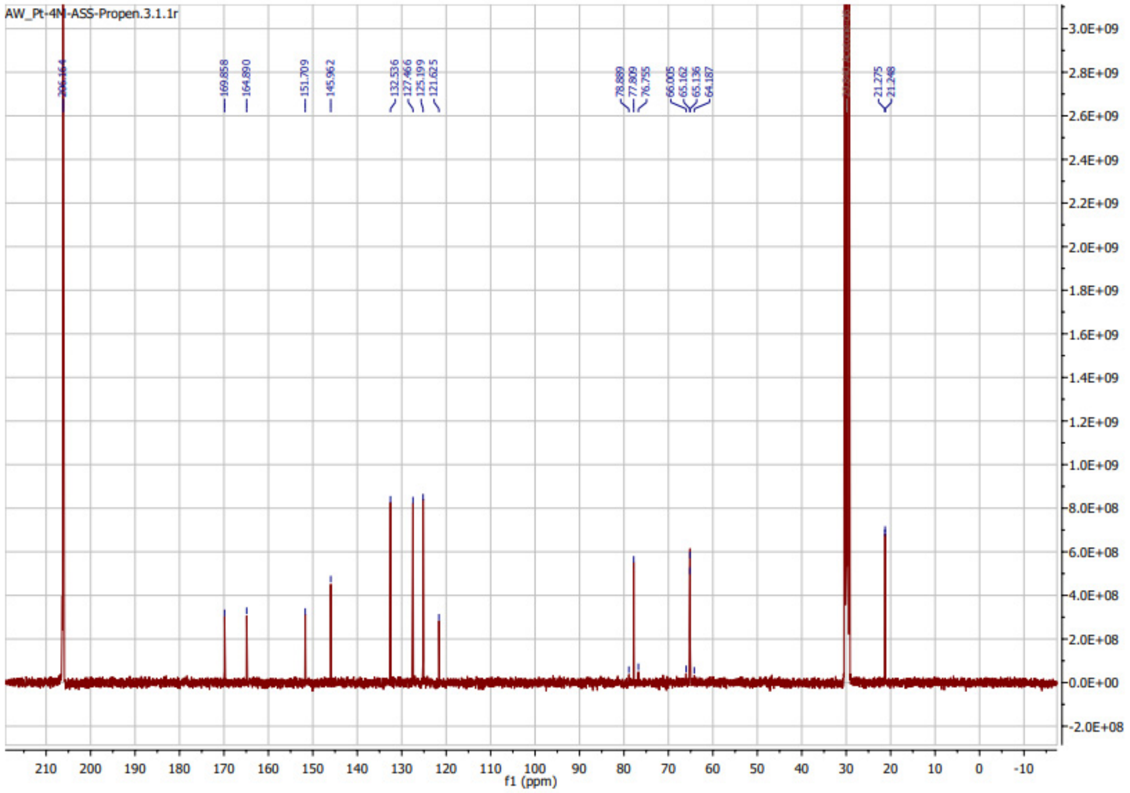

Supporting Figure S68: ^{1}H and ^{13}C NMR spectra of 4-CH_3-ASA-But-$PtCl_3$

^{1}H NMR spectrum of 4-CH_3-ASA-But-$PtCl_3$

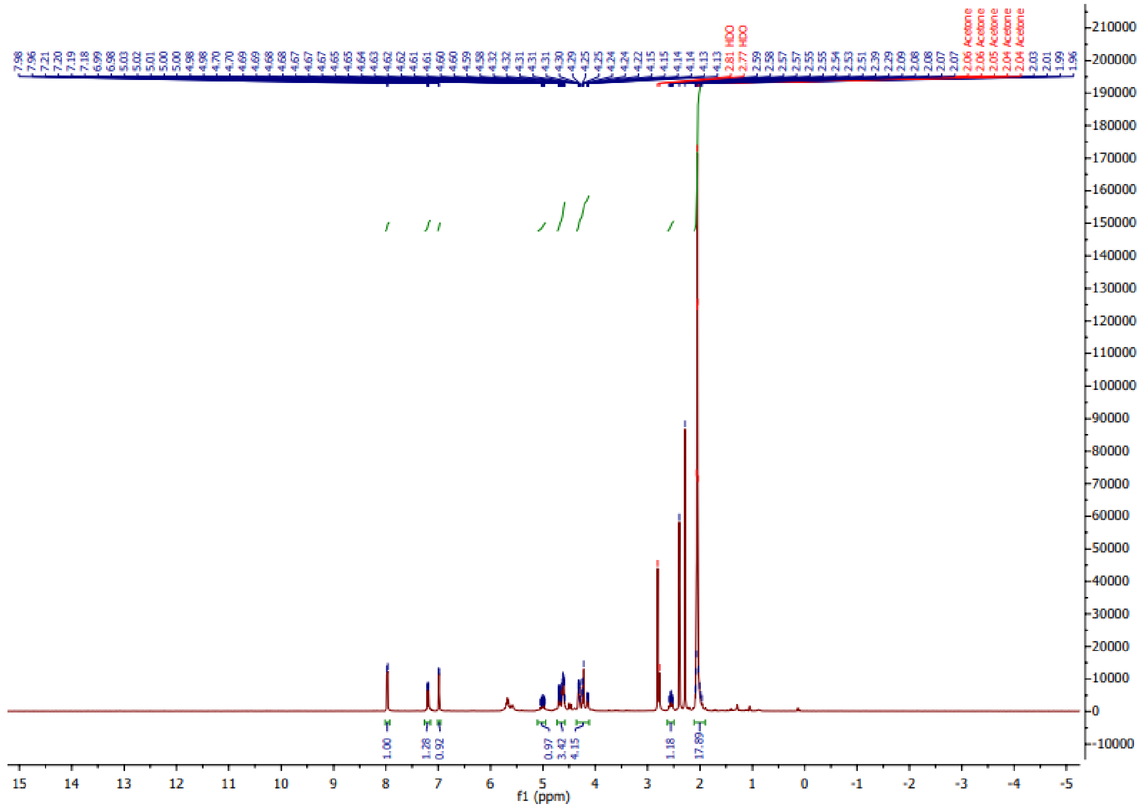

^{13}C NMR spectrum of 4-CH_3-ASA-But-$PtCl_3$

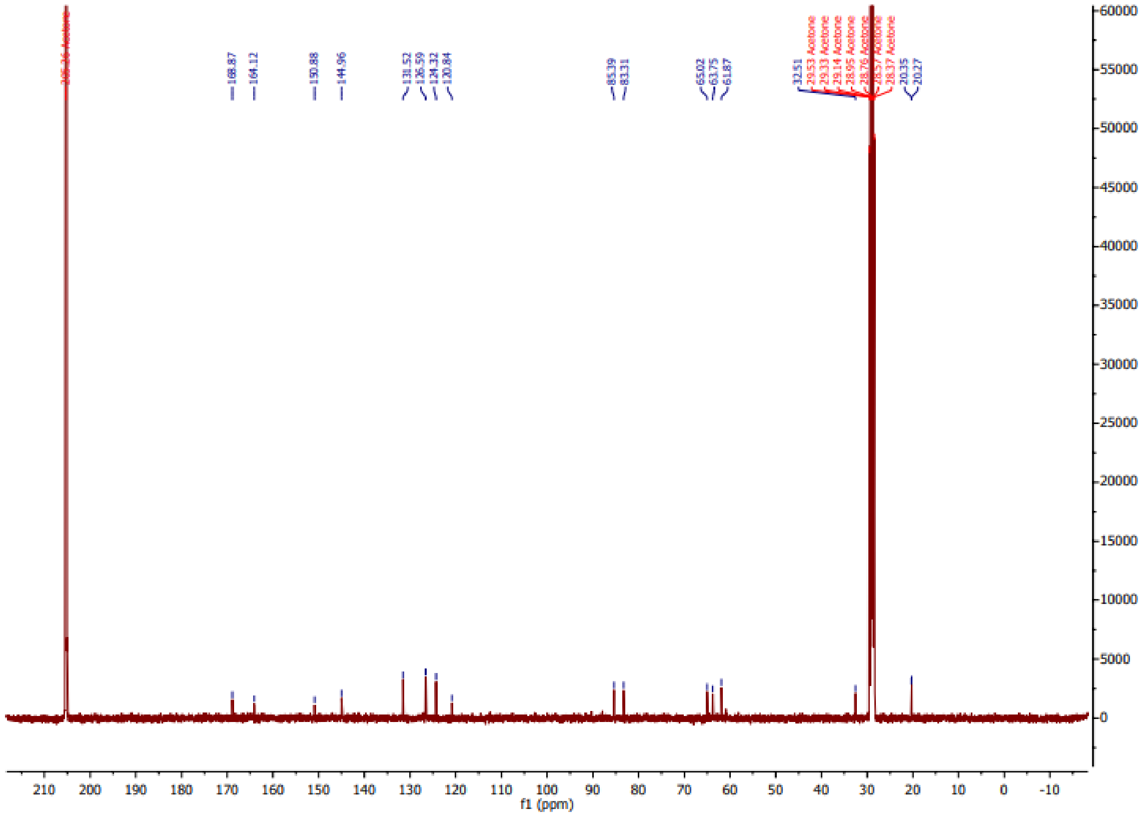

Supporting Figure S69: ¹H and ¹³C NMR spectra of 5-CH₃-ASA-Prop-PtCl₃

^{1}H NMR spectrum of 5-CH_3-ASA-Prop-$PtCl_3$

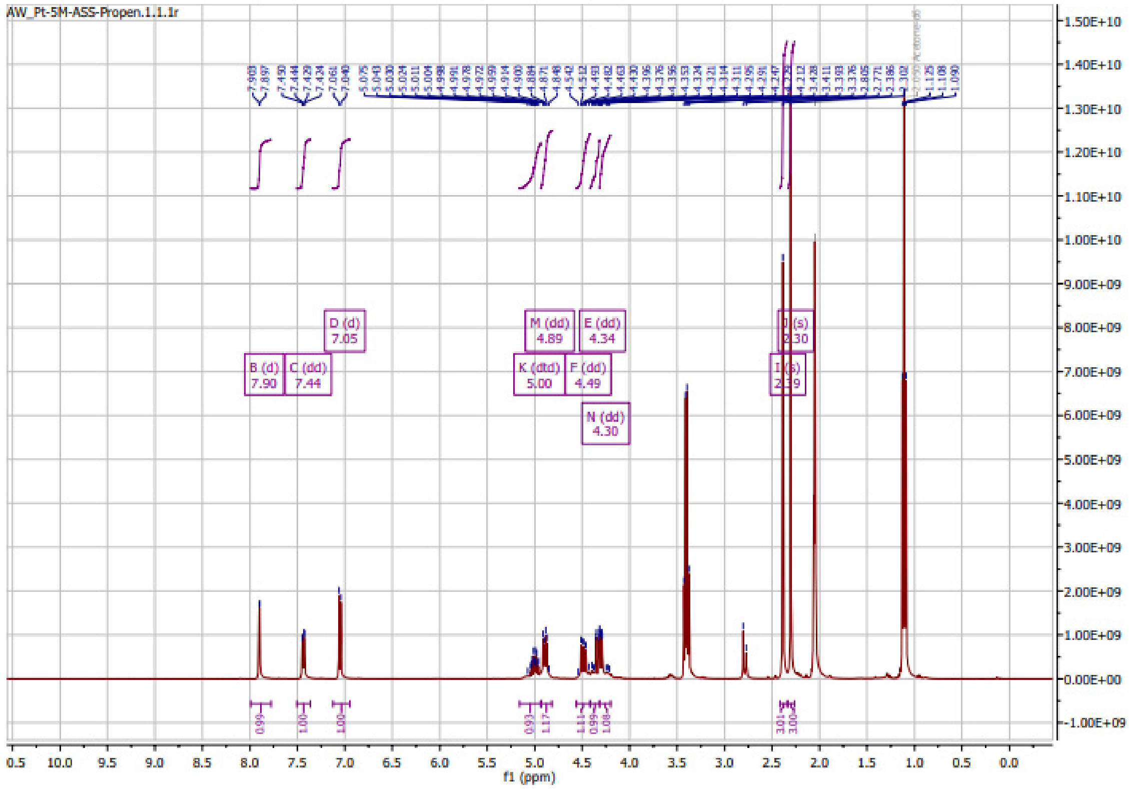

^{13}C NMR spectrum of 5-CH_3-ASA-Prop-$PtCl_3$

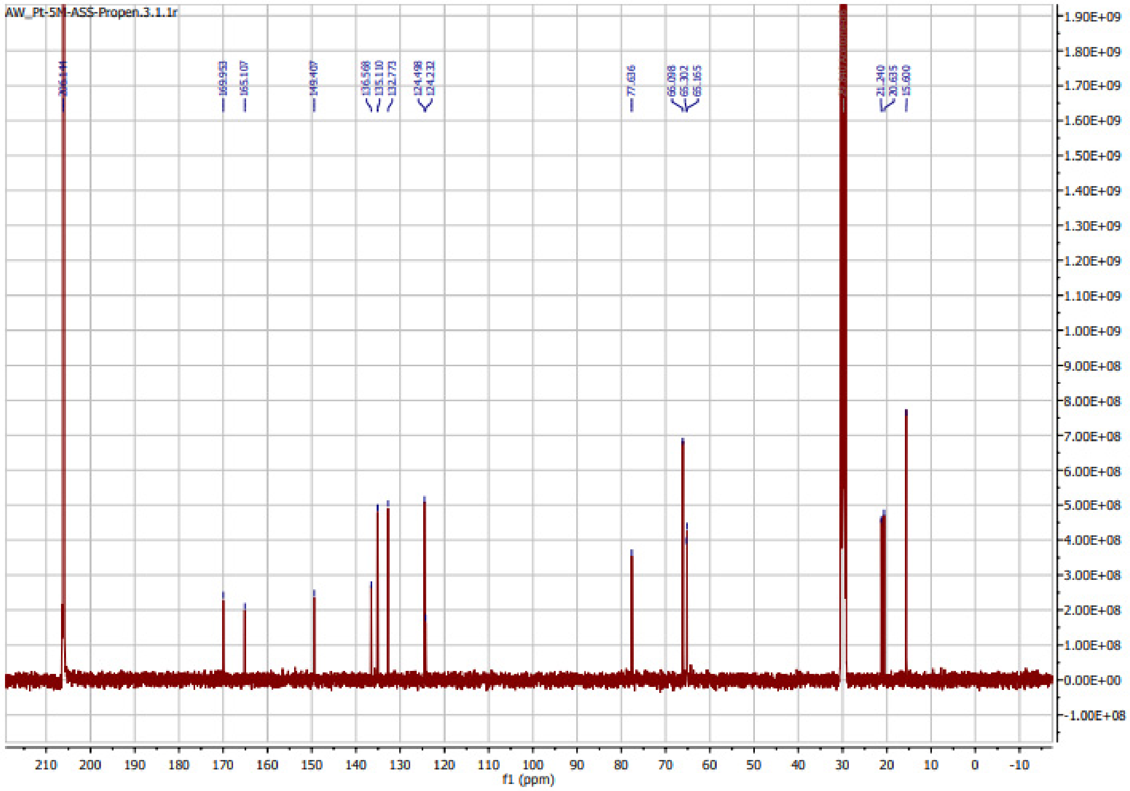

Supporting Figure S70: ^{1}H and ^{13}C NMR spectra of 5-CH_3-ASA-But-$PtCl_3$

^{1}H NMR spectrum of 5-CH_3-ASA-But-$PtCl_3$

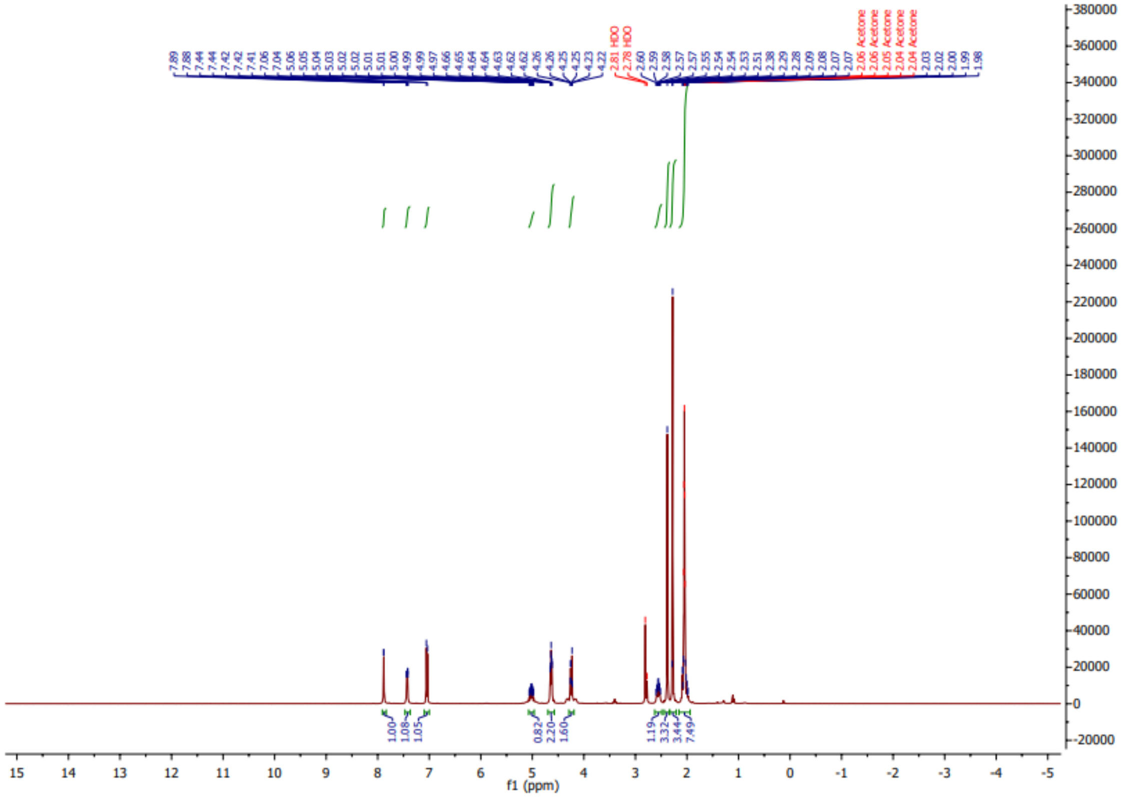

^{13}C NMR spectrum of 5-CH_3-ASA-But-$PtCl_3$

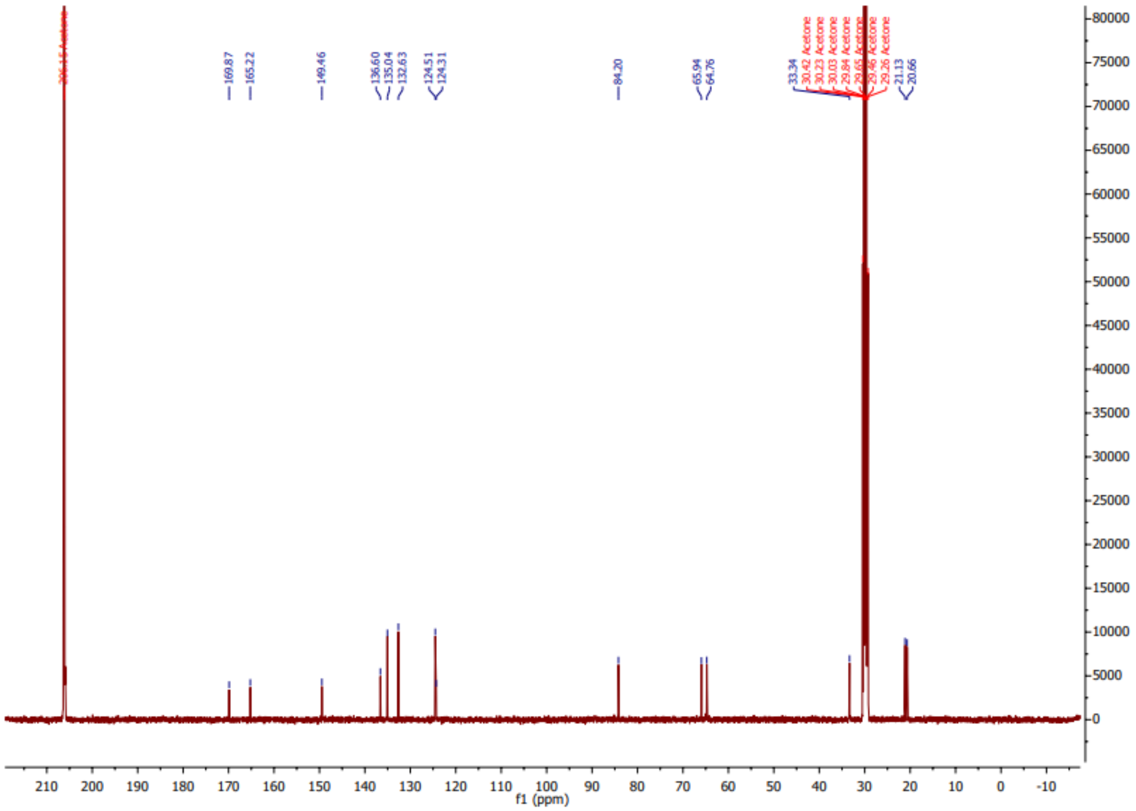

Supporting Figure S71: ^{1}H and ^{13}C NMR spectra of 6-CH_3-ASA-Prop-$PtCl_3$

^{1}H NMR spectrum of 6-CH_3-ASA-Prop-$PtCl_3$

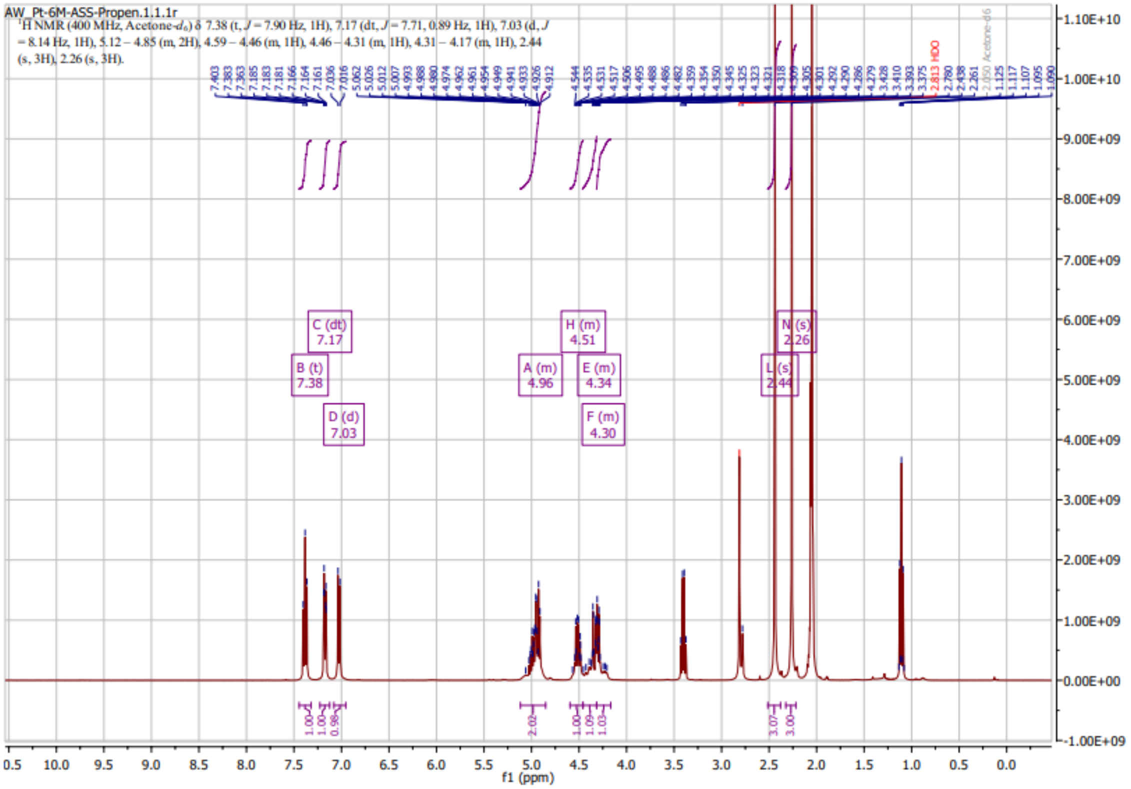

^{13}C NMR spectrum of 6-CH_3-ASA-Prop-$PtCl_3$

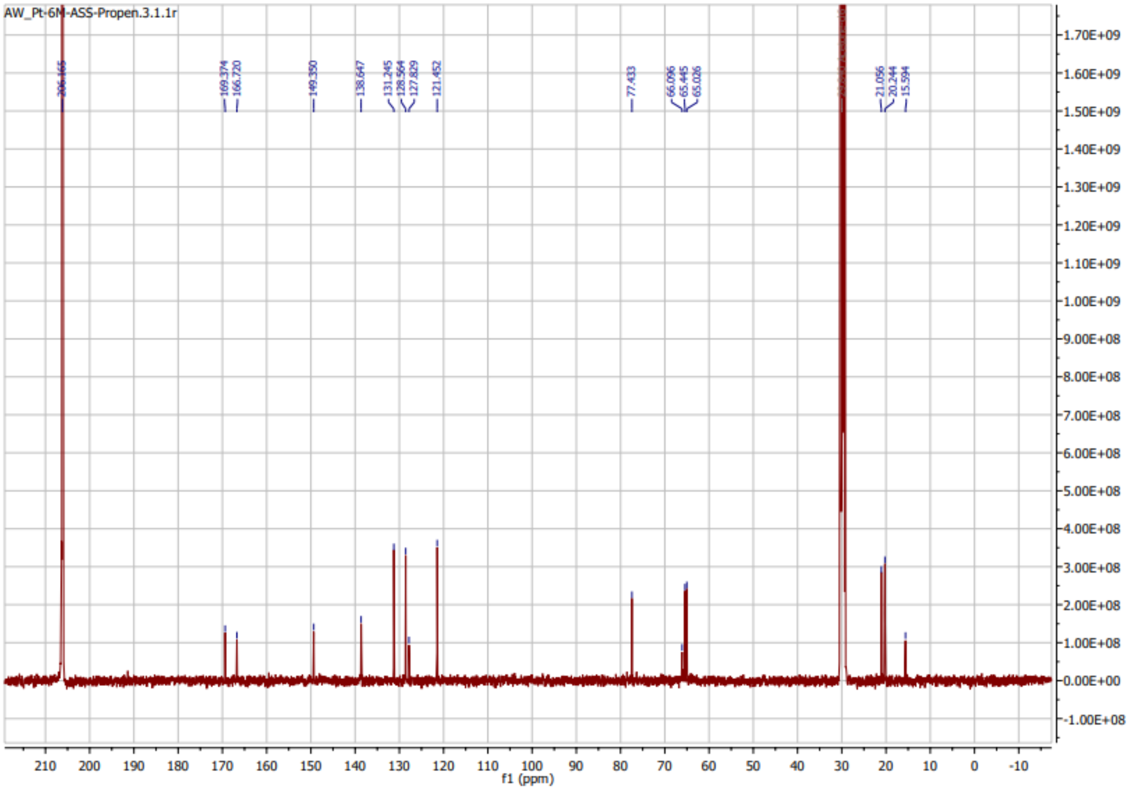

<u>Supporting Figure S72: ^{1}H and ^{13}C NMR spectra of 6-CH$_3$-ASA-But-PtCl$_3$</u>

^{1}H NMR spectrum of 6-CH$_3$-ASA-But-PtCl$_3$

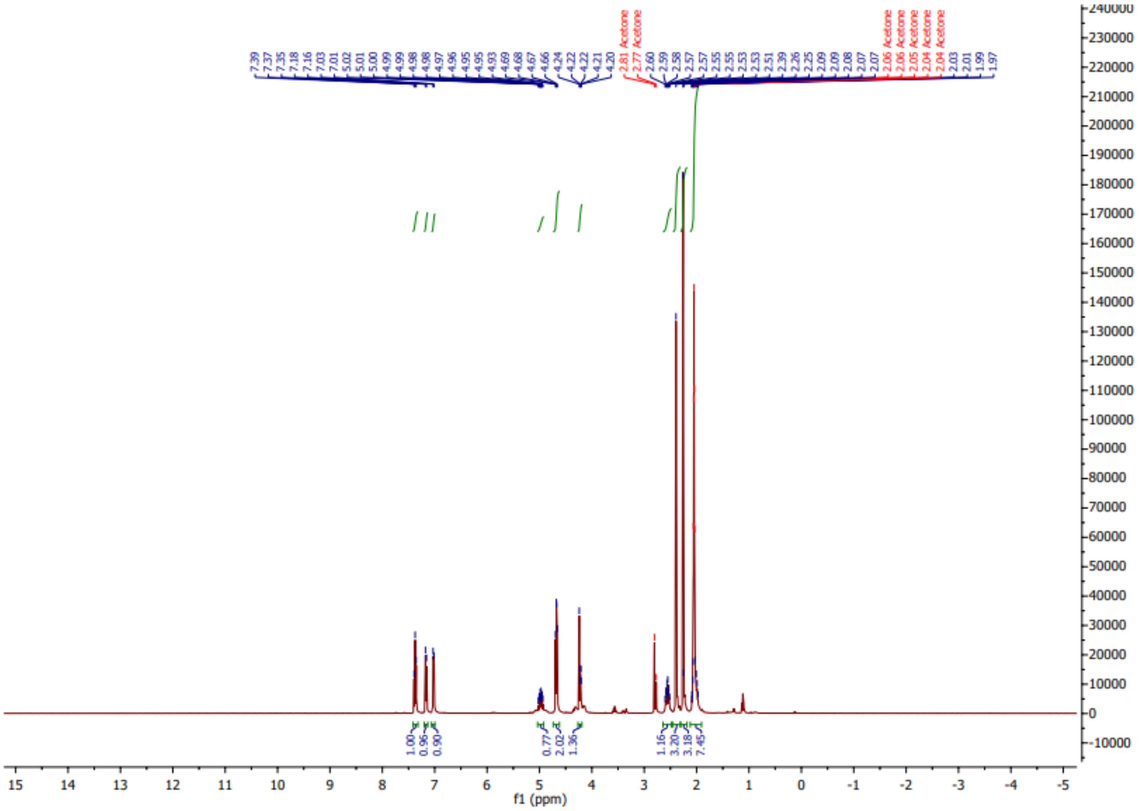

^{13}C NMR spectrum of 6-CH$_3$-ASA-But-PtCl$_3$

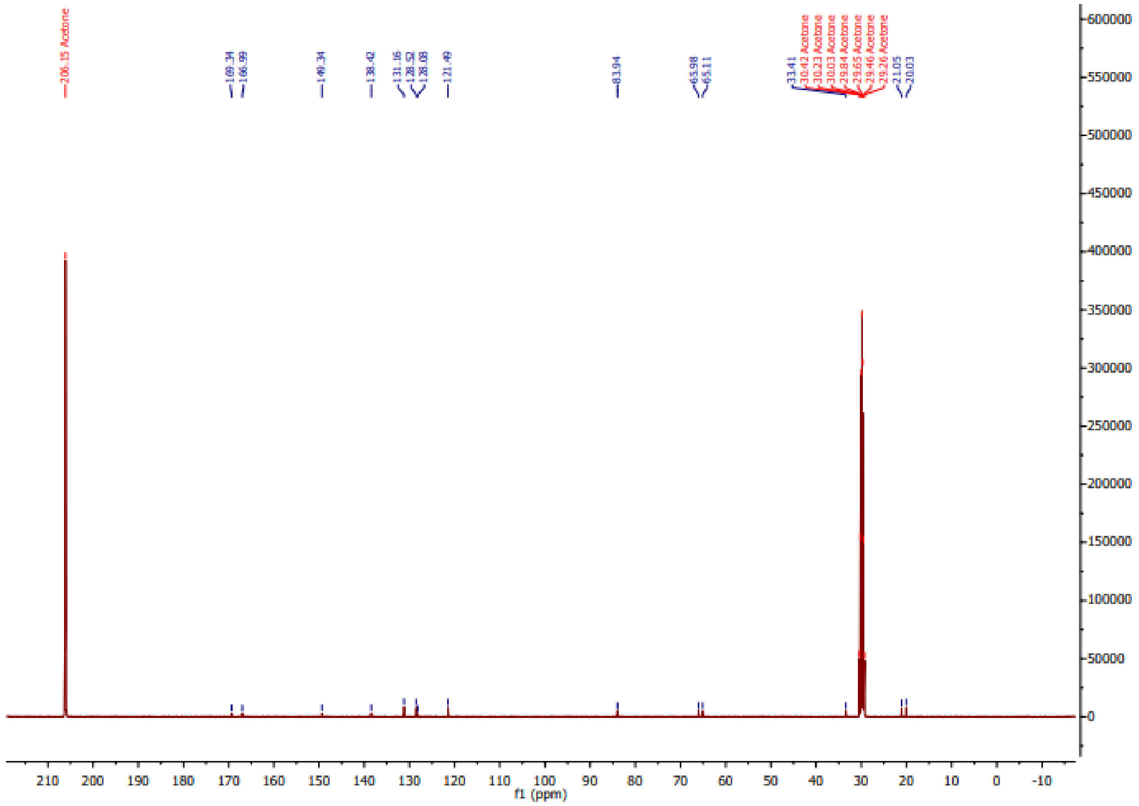

Supporting Figure S73: HR-ESI-MS spectrum of 3-F-ASA-Prop-PtCl3

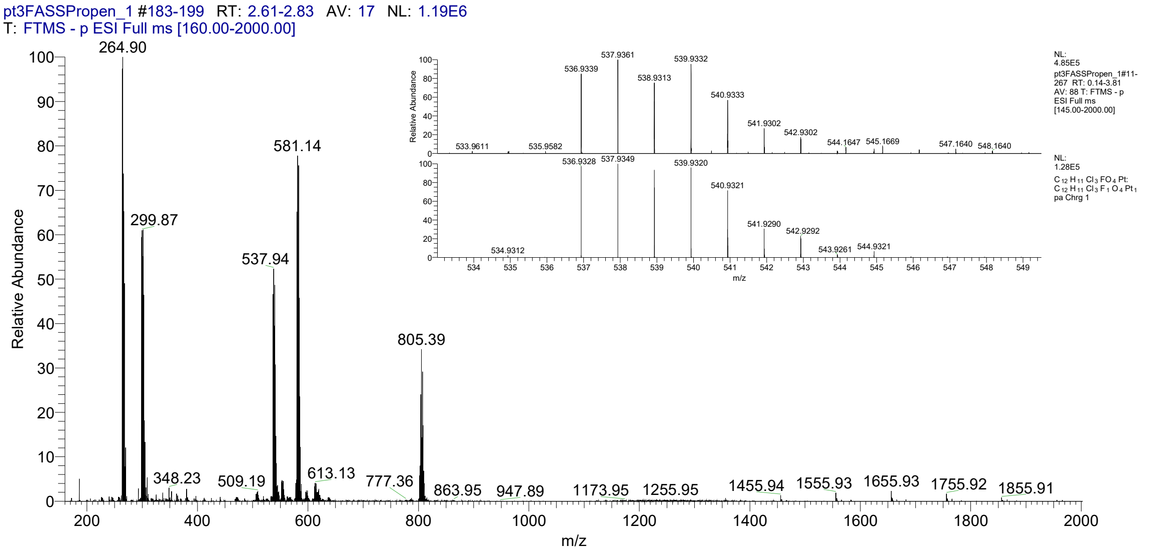

Supporting Figure S74: HR-ESI-MS spectrum of 4-F-ASA-Prop-PtCl3

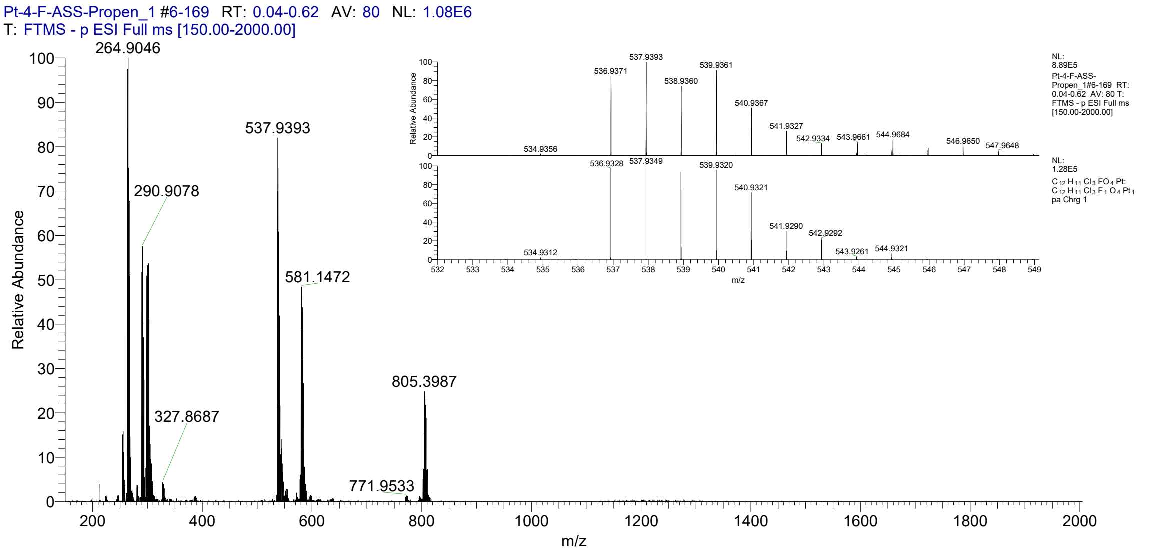

Supporting Figure S75: HR-ESI-MS spectrum of 5-F-ASA-Prop-PtCl$_3$

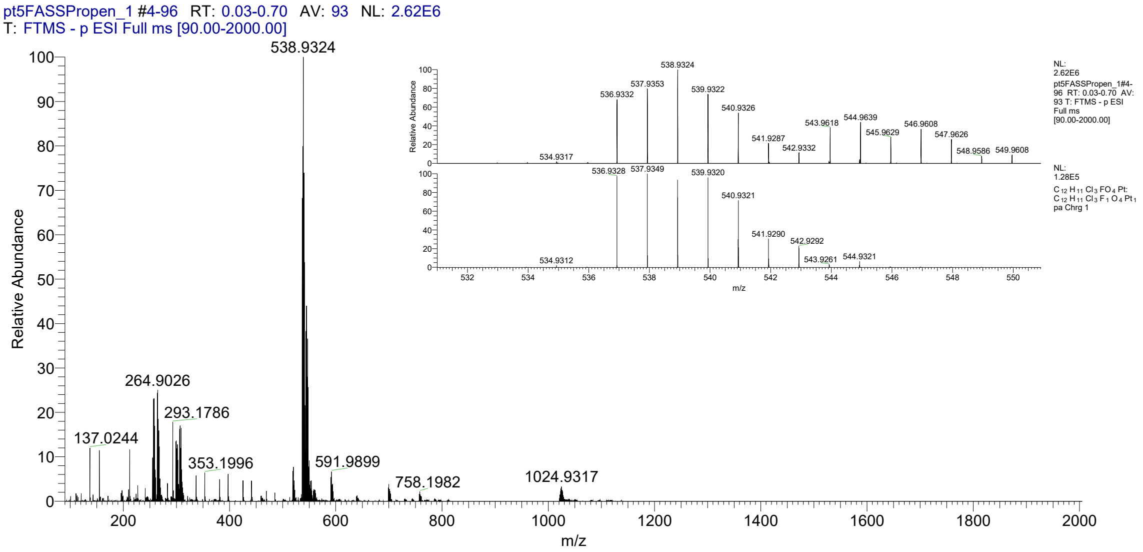

Supporting Figure S76: HR-ESI-MS spectrum of 6-F-ASA-Prop-PtCl$_3$

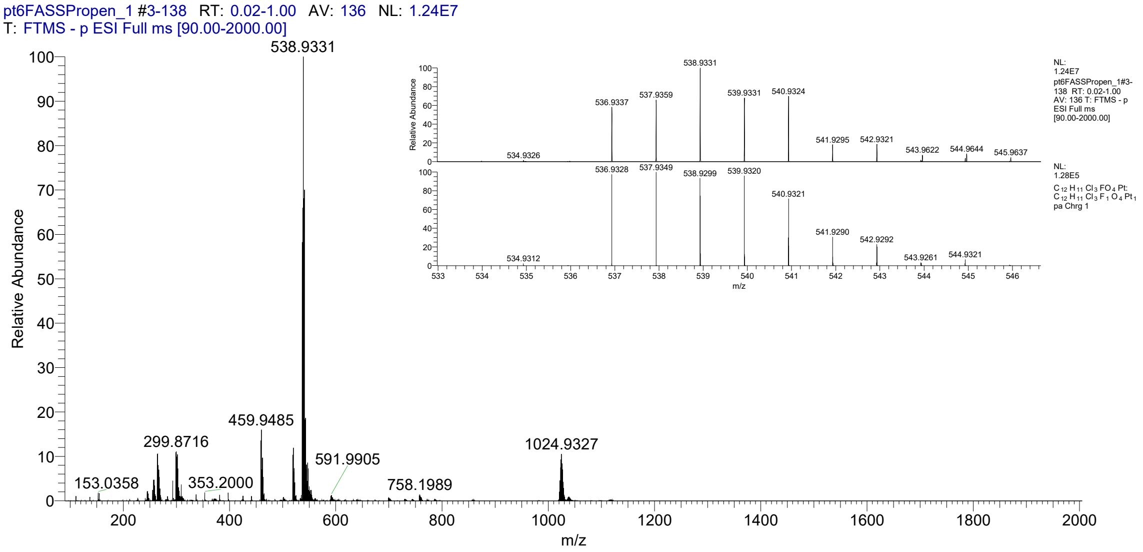

Supporting Figure S77: HR-ESI-MS spectrum of 3-F-ASA-But-PtCl$_3$

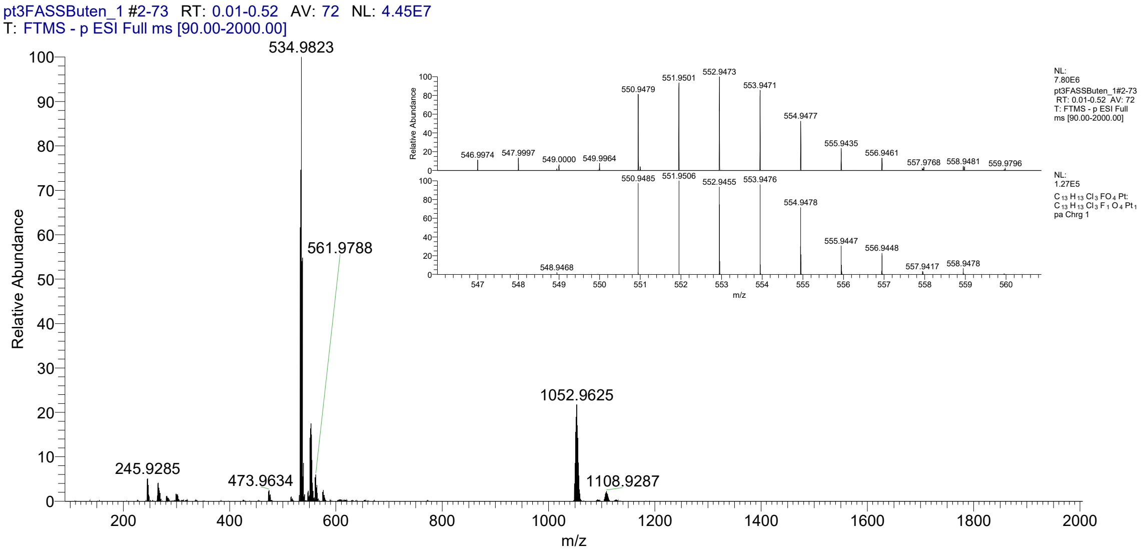

Supporting Figure S78: HR-ESI-MS spectrum of 4-F-ASA-But-PtCl$_3$

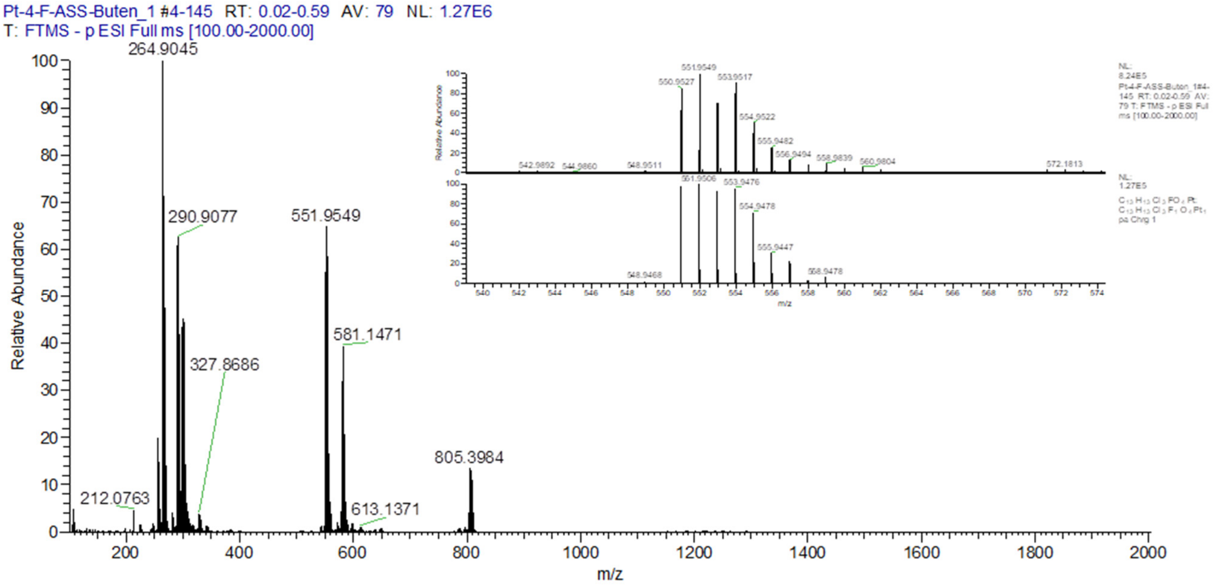

Supporting Figure S79: HR-ESI-MS spectrum of 5-F-ASA-But-PtCl$_3$

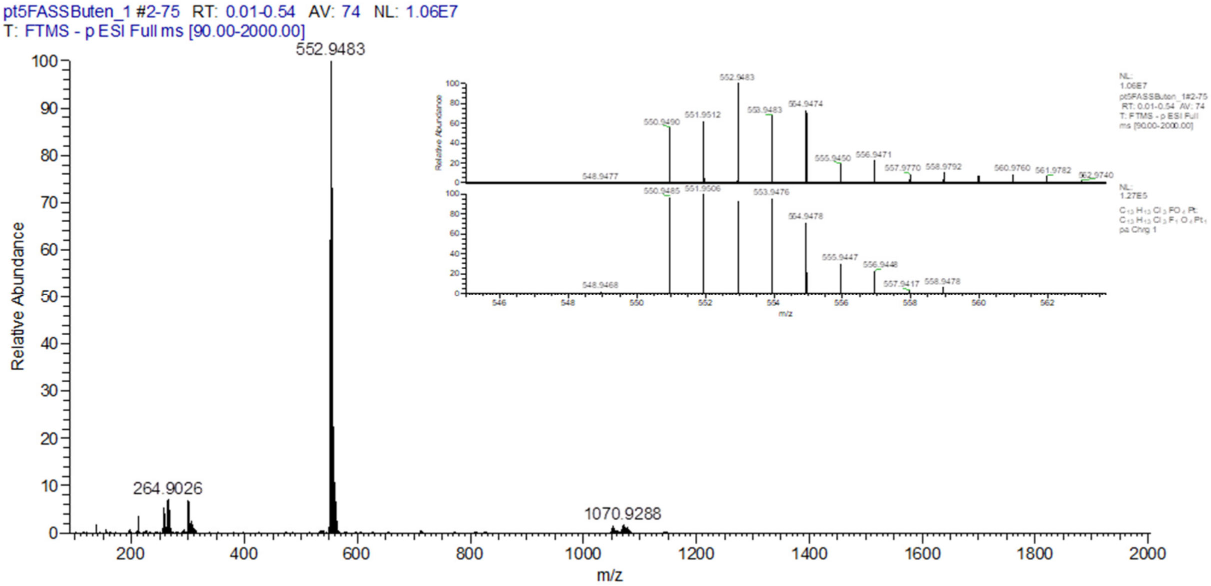

Supporting Figure S80: HR-ESI-MS spectrum of 6-F-ASA-But-PtCl$_3$

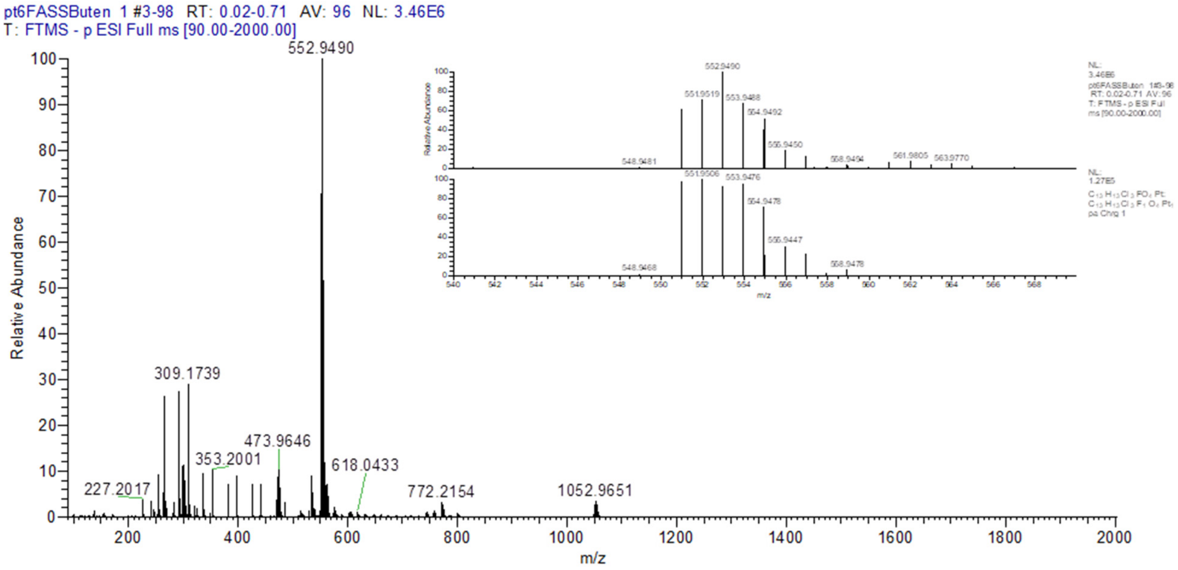

Supporting Figure S81: HR-ESI-MS spectrum of 3-Cl-ASA-Prop-$PtCl_3$

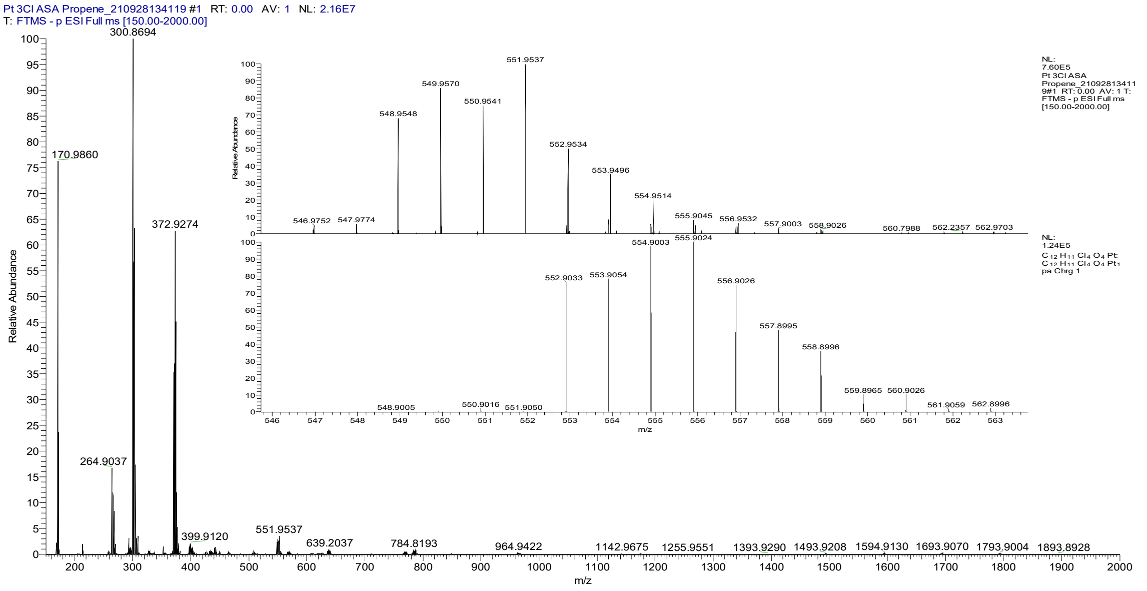

Supporting Figure S82: HR-ESI-MS spectrum of 4-Cl-ASA-Prop-$PtCl_3$

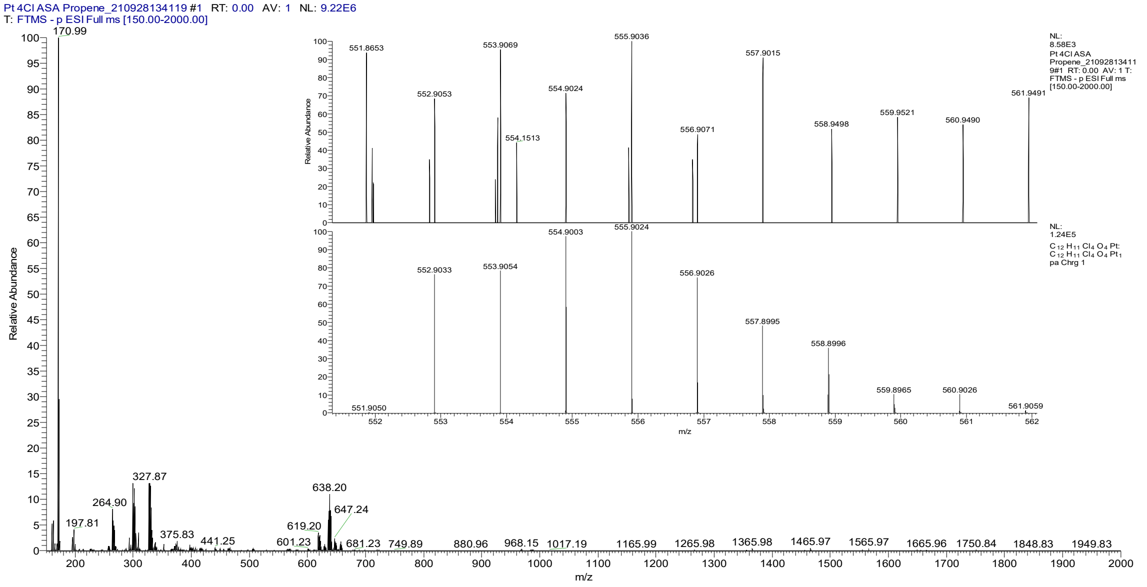

Supporting Figure S83: HR-ESI-MS spectrum of 5-Cl-ASA-Prop-PtCl₃

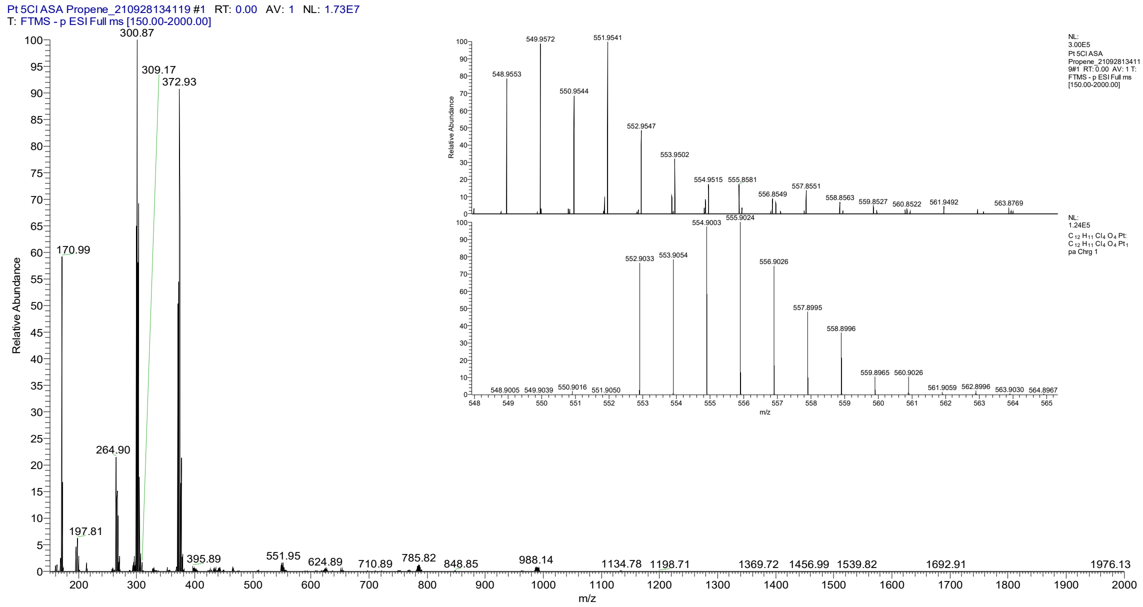

Supporting Figure S84: HR-ESI-MS spectrum of 6-Cl-ASA-Prop-PtCl₃

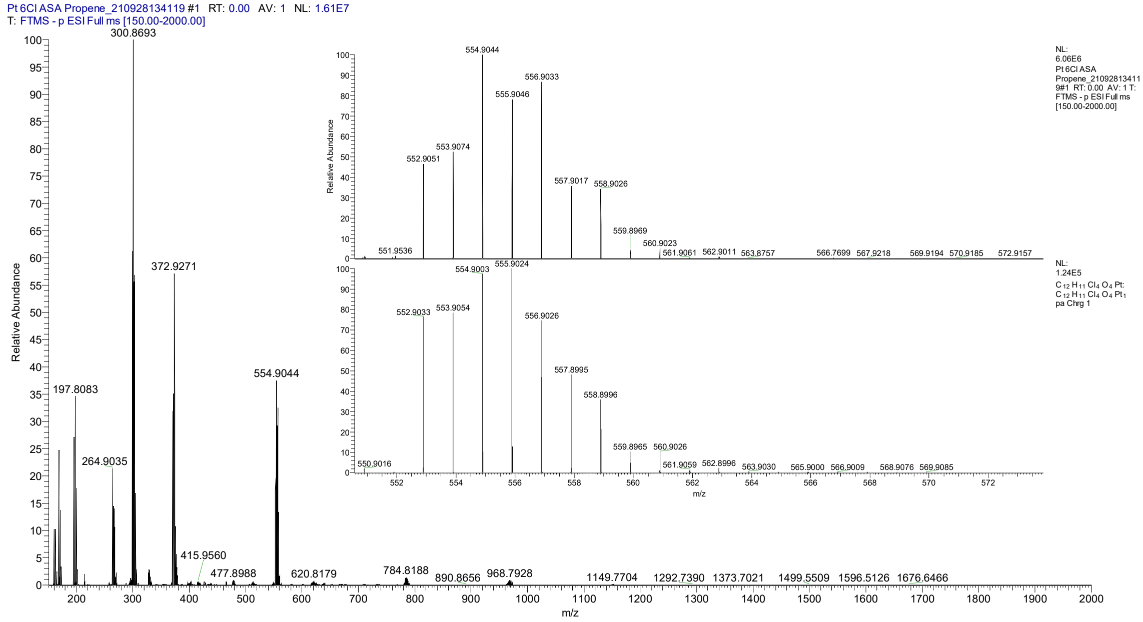

Supporting Figure S84: HR-ESI-MS spectrum of 3-Cl-ASA-But-$PtCl_3$

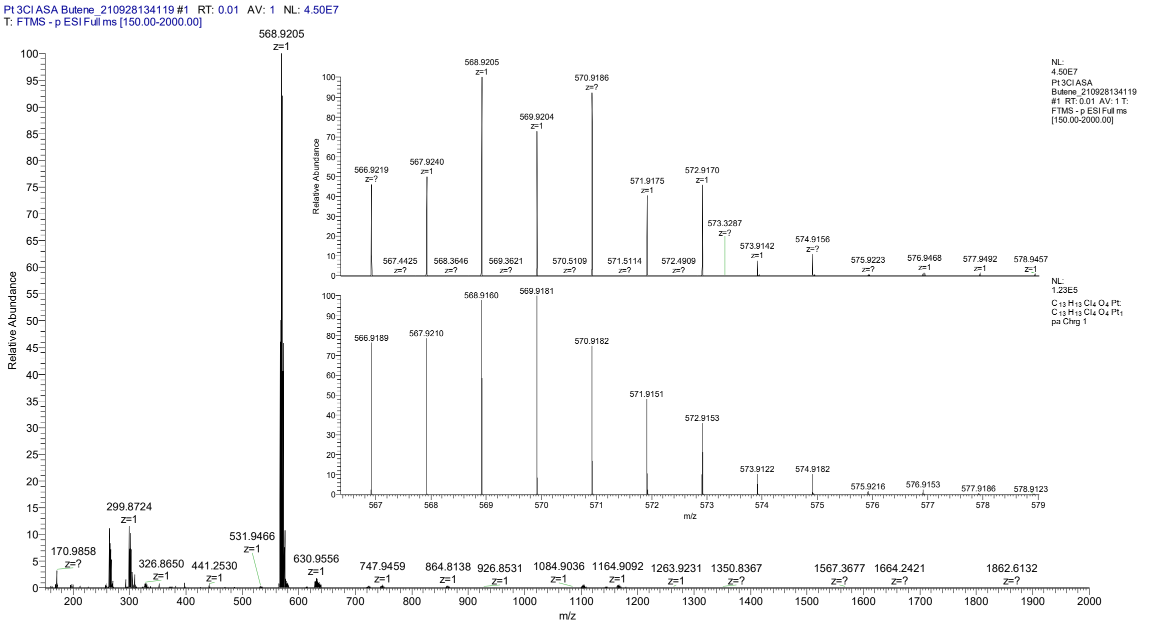

Supporting Figure S85: HR-ESI-MS spectrum of 4-Cl-ASA-But-$PtCl_3$

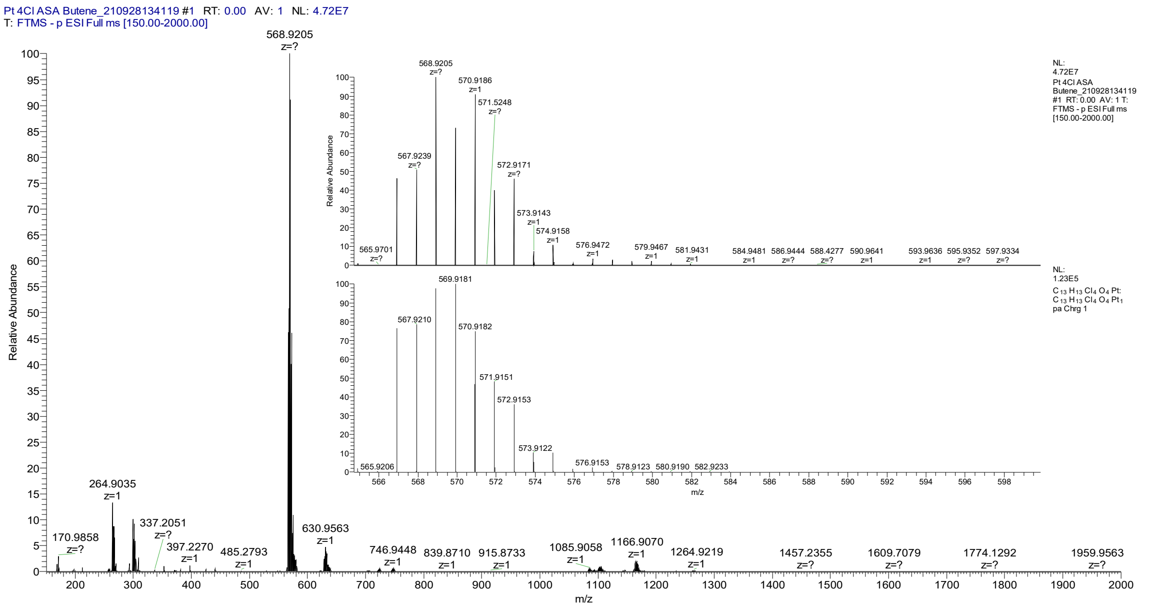

Supporting Figure S86: HR-ESI-MS spectrum of 5-Cl-ASA-But-PtCl$_3$

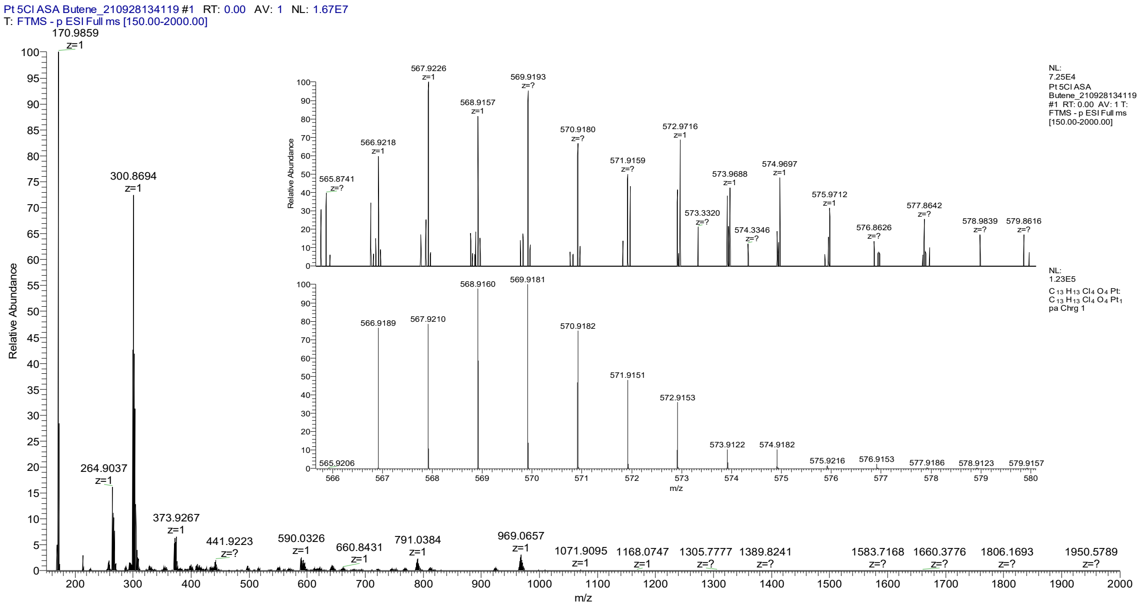

Supporting Figure S87: HR-ESI-MS spectrum of 6-Cl-ASA-But-PtCl$_3$

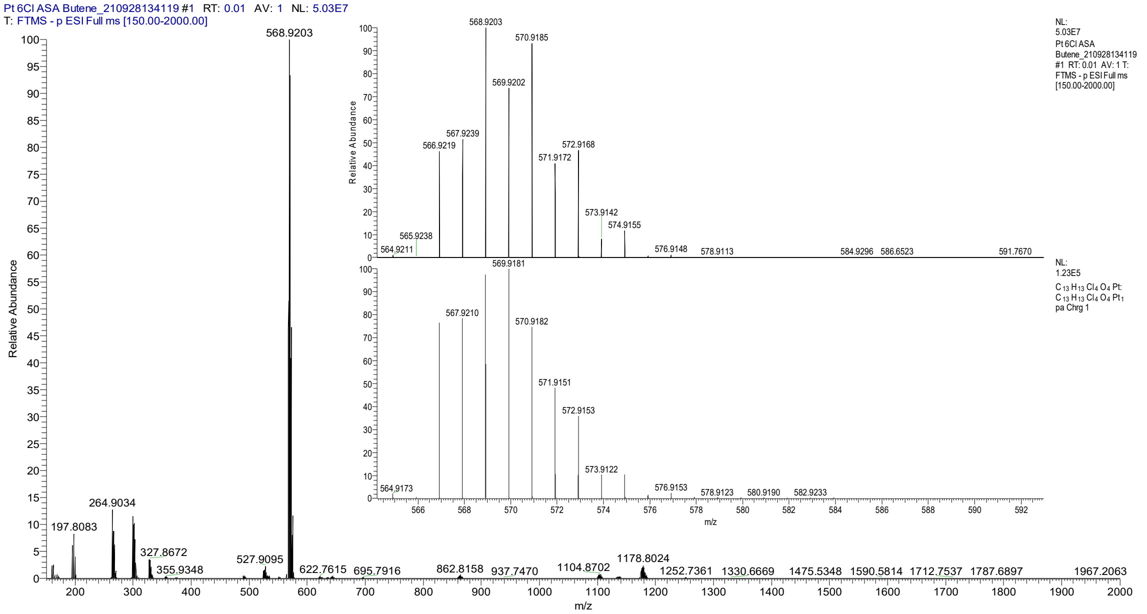

Supporting Figure S88: HR-ESI-MS spectrum of 3-CH_3-ASA-Prop-$PtCl_3$

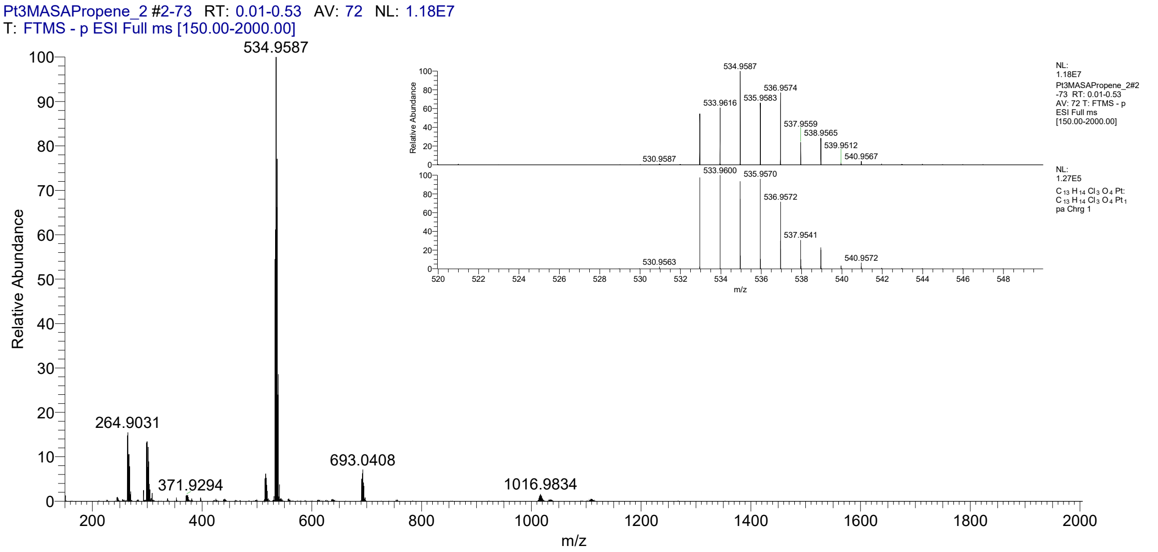

Supporting Figure S89 HR-ESI-MS spectrum of 4-CH_3-ASA-Prop-$PtCl_3$

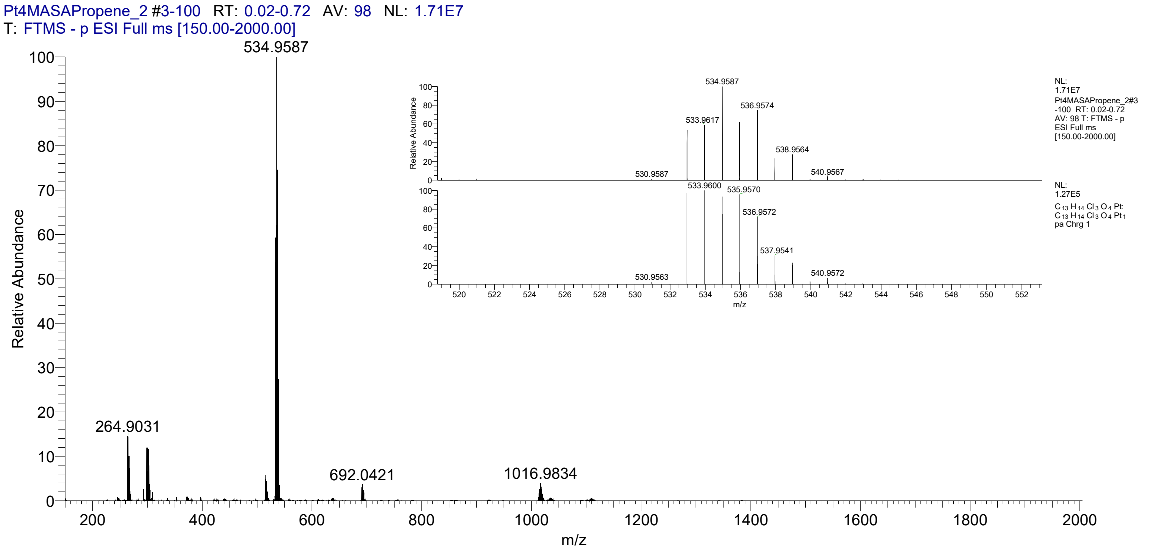

Supporting Figure S90: HR-ESI-MS spectrum of 5-CH_3-ASA-Prop-$PtCl_3$

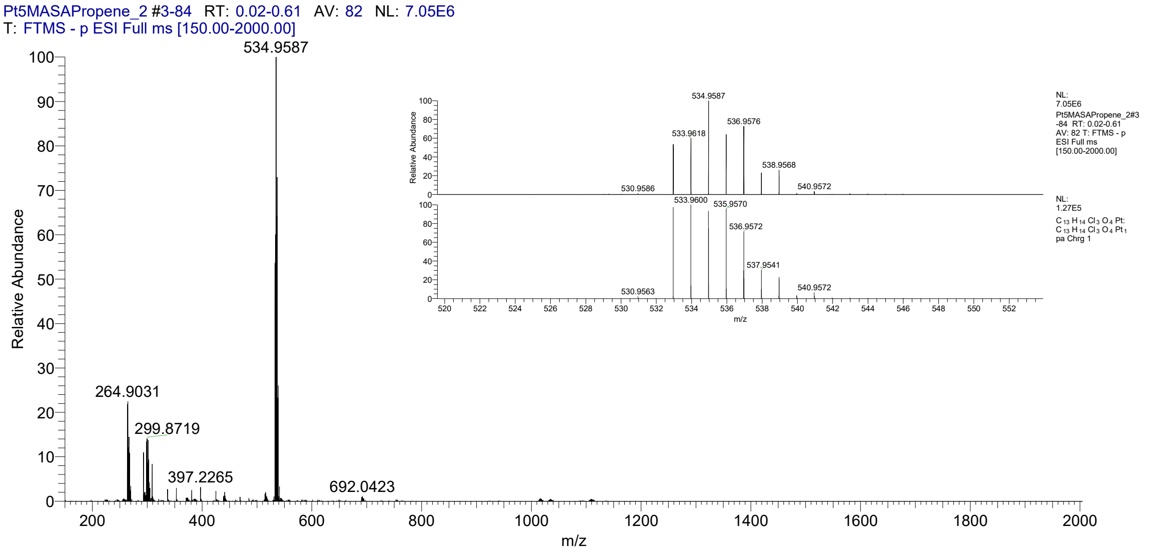

Supporting Figure S91: HR-ESI-MS spectrum of 6-CH_3-ASA-Prop-$PtCl_3$

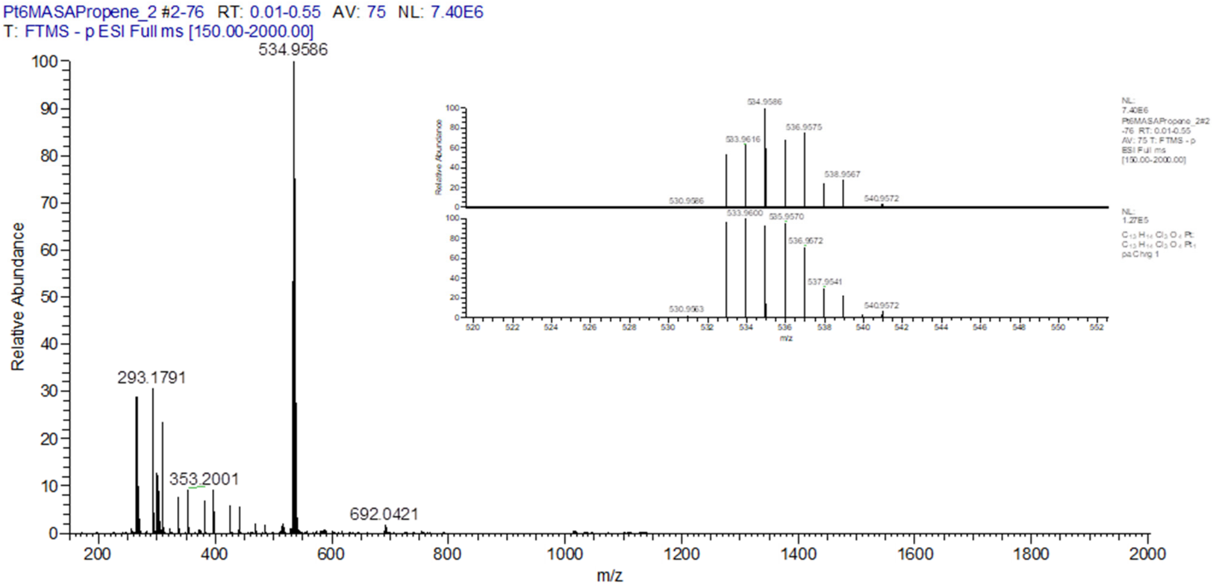

Supporting Figure S92: HR-ESI-MS spectrum of 3-CH_3-ASA-But-$PtCl_3$

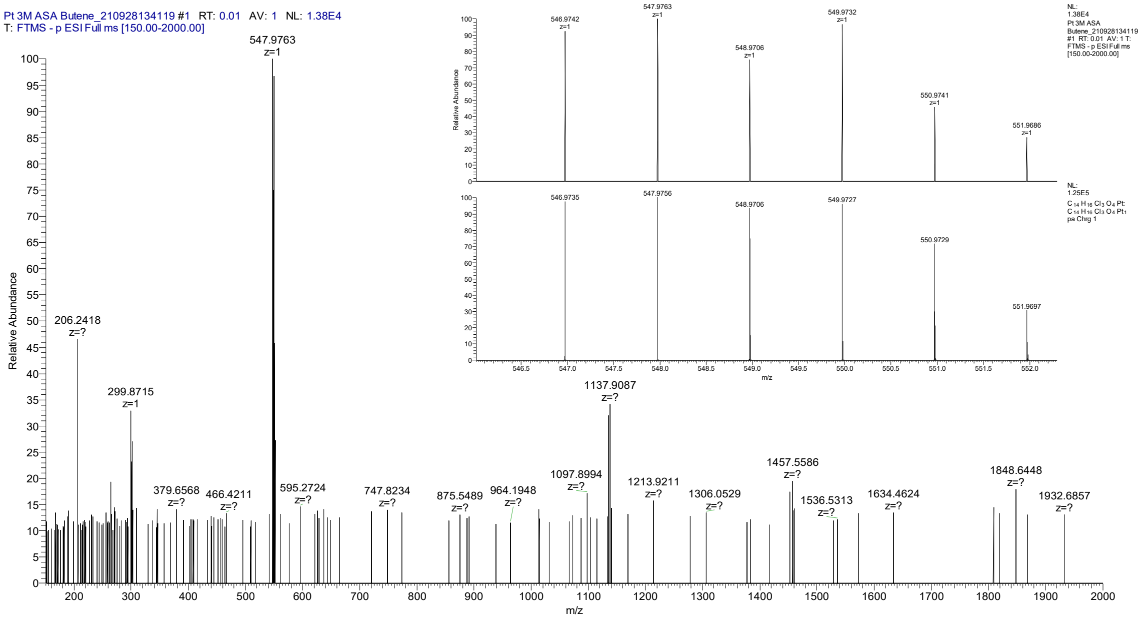

Supporting Figure S93: HR-ESI-MS spectrum of 4-CH_3-ASA-But-$PtCl_3$

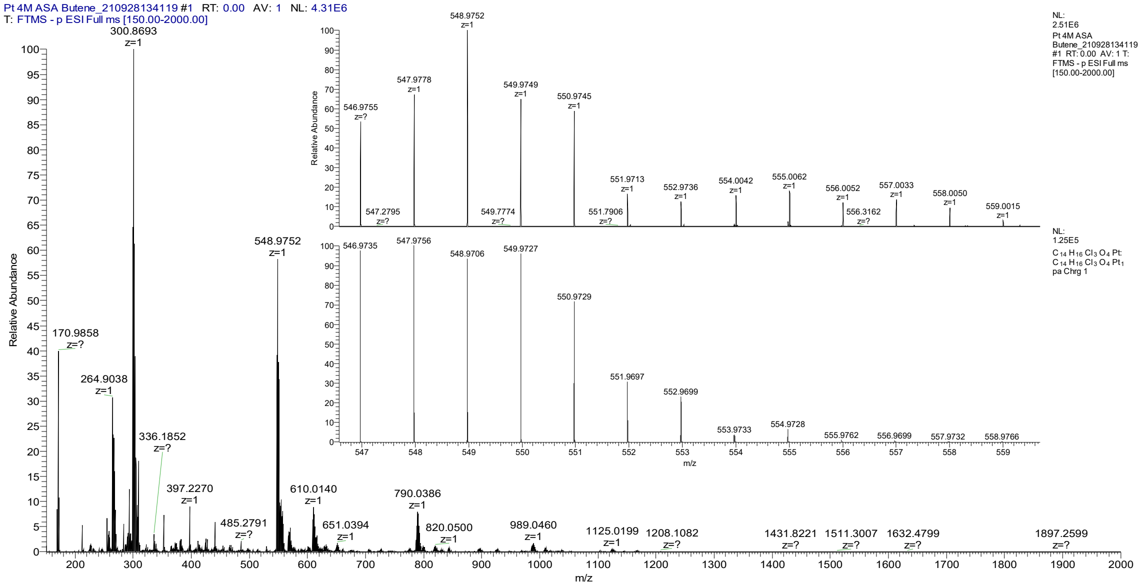

Supporting Figure S94: HR-ESI-MS spectrum of 5-CH_3-ASA-But-$PtCl_3$

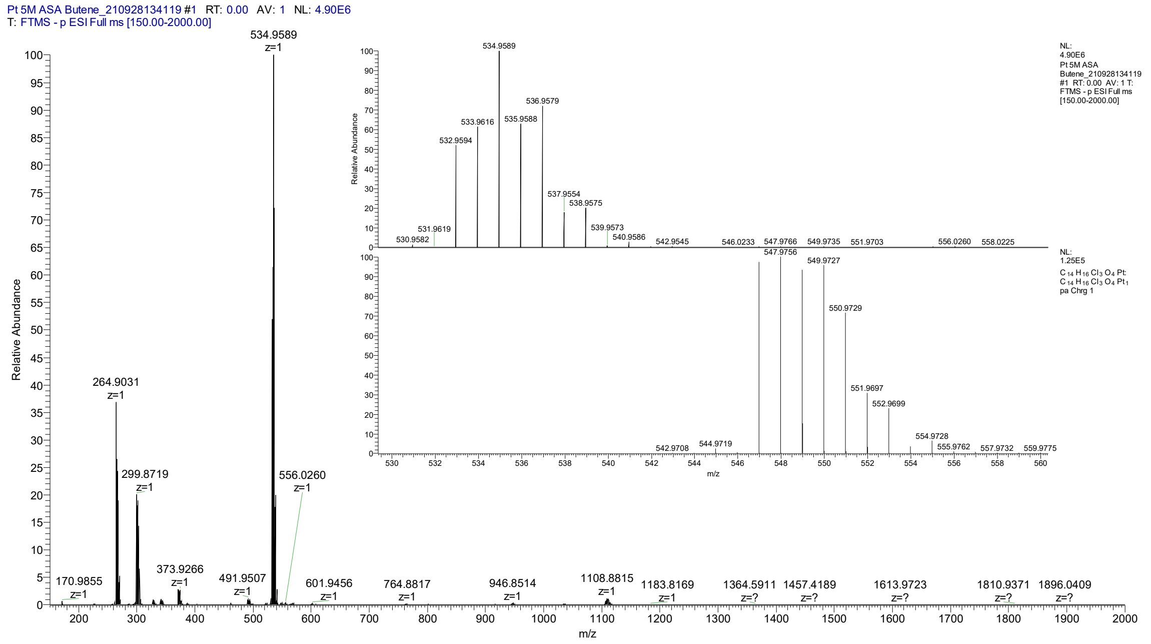

Supporting Figure S95: HR-ESI-MS spectrum of 6-CH_3-ASA-But-$PtCl_3$

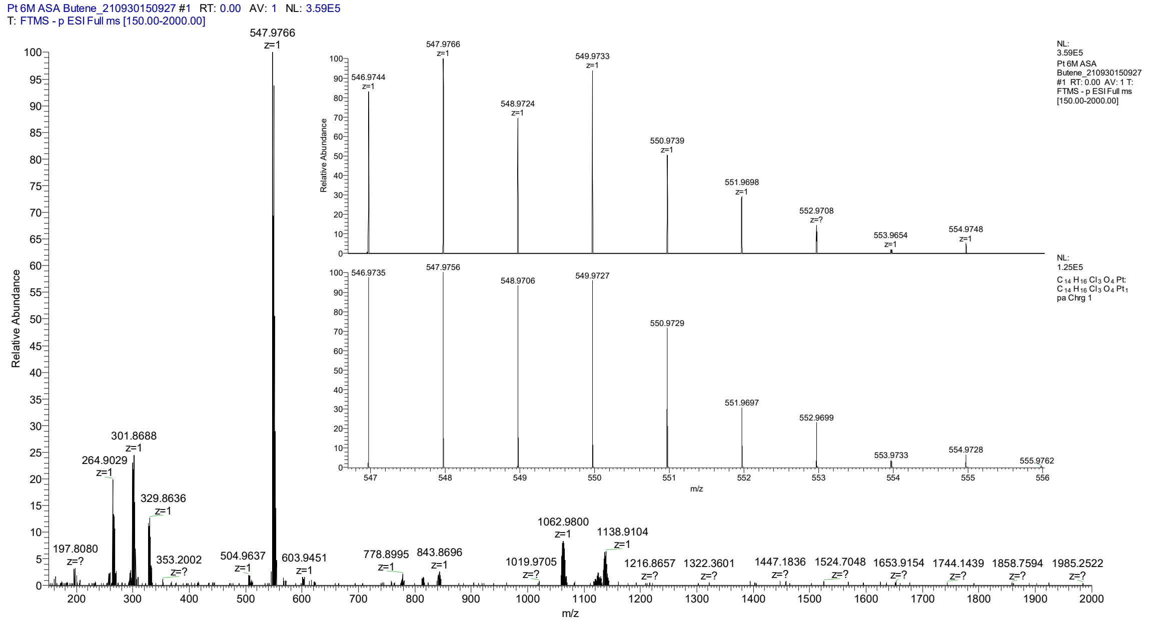

IR data of selected ligands and complexes

The conditions are given in the "Experimental section" of the article.

Table S1: IR data of selected ligands and complexes

**3-Cl-Prop-ASA**	**IR:** ν (cm^{-1}) = 3096 vw, 2999 vw, 2928 vw, 1764 m (C=O), 1717 s (C=O), 1650 (C=C, aliph.) 1593 m (C=C, arom.), 1443 m, 1369 m, 1259 s (C-O-C), 1188 s (C-O-C), 981 s, 907 s, 752 s (C-H, oop), 733 s.
**4-Cl-Prop-ASA**	**IR:** ν (cm^{-1}) = 3093 vw, 2983 vw, 2928 vw, 1766 m (C=O), 1703 s (C=O), 1648 w (C=C, aliph.), 1596 m (C=C, arom.), 1457 w, 1371 m, 1248 m (C-O-C), 1189 s (C-O-C), 933 s, 900 m, 775 m (C-H, oop).
**5-Cl-Prop-ASA**	**IR:** (cm^{-1}) = 3096 vw, 2983 vw, 2923 vw, 1756 s (C=O), 1721 s (C=O), 1648 (C=C, aliph.), 1599 m (C=C, arom.), 1437 w, 1370 m, 1241 s (C-O-C), 1197 s (C-O-C), 1100 s, 780 m (C-H, oop), 532 s.
**6-Cl-Prop-ASA**	**IR:** ν (cm^{-1}) = 3092 vw, 2983 vw, 2934 vw, 1772 m (C=O), 1733 s (C=O), 1649 w (C=C, aliph.), 1597 m (C=C, arom.), 1447 m, 1369 m, 1260 s (C-O-C), 1181 s (C-O-C), 1105 m, 932 s, 787 m (C-H, oop).
**3-Cl-But-ASA**	**IR:** ν (cm^{-1}) = 3091 vw, 2997 w, 3002 vw, 1768 s (C=O), 1711 s (C=O), 1643 m (C=C, aliph.), 1598 m (C=C, arom.), 1446 m, 1369m, 1287 s (C-O-C), 1189 s (C-O-C), 904 s, 809 m, 754 m (C-H, oop).
**4-Cl-But-ASA**	**IR:** ν (cm^{-1}) = 3072 vw, 2984 vw, 2944 vw, 1757 s (C=O), 1708 s (C=O), 1645 w (C=C, aliph.), 1597 s (C=C, arom.), 1483 m, 1368 m, 1288 m (C-O-C), 1190 s (C-O-C), 925 m, 892 m, 771 m (C-H, oop).
**5-Cl-But-ASA**	**IR:** ν (cm^{-1}) = 3089 vw, 2982 vw, 2957 vw, 1758 s (C=O), 1719 s (C=O), 1643 w (C=C, aliph.), 1600 m (C=C, arom.), 1475 m, 1370 m, 1293 s (C-O-C), 1200 s (C-O-C), 1100 s, 1081 s, 782 m (C-H, oop).
**6-Cl-But-ASA**	**IR:** ν (cm^{-1}) = 3081 vw, 2982 vw, 2960 vw, 1773 m (C=O), 1732 s (C=O), 1643 w (C=C. aliph.), 1597 m (C=C, arom.), 1447 m, 1369 m, 1264 m (C-O-C), 1181 s (C-O-C), 1105 m, 1059 m, 933 m, 786 m (C-H, oop).
**3-Cl-Prop-ASA-$PtCl_3$**	**IR:** ν (cm^{-1}) = 3073 vw, 2975 vw, 2933 vw, 1761 m (C=O), 1699 s C=O), 1595 w (C=C, arom.), 1492 w (C=C, aliph.), 1442 m, 1364 m, 1288 s (C-O-C), 1194 s (C-O-C), 984 m, 911 m, 756 m (C-H, oop).
**4-Cl-Prop-ASA-$PtCl_3$**	**IR:** ν (cm^{-1}) = 3070 vw, 2980 vw, 2931 vw, 1762 s (C=O), 1708 s (C=O), 1600 m (C=C, arom.), 1484 w (C=C, aliph.), 1440 m, 1369 m, 1286 s (C-O-C), 1200 s (C-O-C), 940 m, 899 m, 775 (C-H, oop).
**5-Cl-Prop-ASA-$PtCl_3$**	**IR:** ν (cm^{-1}) = 3081 vw, 2976 vw, 2931 vw, 1764 m (C=O), 1697 s (C=O), 1598 m (C=C, arom.), 1483 w (C=C, aliph.), 1447 m, 1367 m, 1282 s (C-O-C), 1188 s (C-O-C), 1107 m, 938 m, 774 m (C-H,oop).
**6-Cl-Prop-ASA-$PtCl_3$**	**IR:** ν (cm^{-1}) = 3077 vw, 2935 vw, 2868 vw, 1764 m (C=O), 1724 s (C=O), 1596 m (C=C, arom.), 1502 w (C=C, aliph.), 1446 m, 1368 m, 1266 s (C-O-C), 1185 s (C-O-C), 1104 s, 938 m, 788 m (C-H, oop).
**3-Cl-But-ASA-$PtCl_3$**	**IR:** ν (cm^{-1}) = 3075 vw, 2973 vw, 2873 vw, 1756 m (C=O), 1717 s (C=O), 1596 m (C=C, arom.), 1501 (C=C, aliph.), 1444 s, 1368 m, 1289 s (C-O-C), 1189 s (C-O-C), 906 s, 806 m, 756 m (C-H, oop).
**4-Cl-But-ASA-$PtCl_3$**	**IR:** ν (cm^{-1}) = 3075 vw, 2970 vw, 1758 s (C=O), 1711 s (C=O), 1599 s (C=C, arom.), 1505 (C=C, aliph.), 1484 m, 1370 m, 1298 s (C-O-C), 1190 s (C-O-C), 937 m, 895 m, 756 m (C-H, oop).
**5-Cl-But-ASA-$PtCl_3$**	**IR:** ν (cm^{-1}) = 1744 s (C=O), 1714 s (C=O), 1602 m (C=C, arom.), 1502 w (C=C, aliph.), 1484 m, 1369 m, 1294 m (C-O-C), 1203 s (C-O-C), 1104 s, 1028 s, 780 m (C-H, oop).
**6-Cl-But-ASA-$PtCl_3$**	**IR:** ν (cm^{-1}) = 2972 vw, 1748 s (C=O), 1703 s (C=O), 1595 m (C=C, arom.), 1502 w (C=C, aliph.), 1445 m, 1369 m, 1285 s (C-O-C), 1218 s (C-O-C), 1117 m, 1064 m, 942 m, 774 m (C-H, oop).

Inhibition of COX-1/2

The conditions are given in the "Experimental section" of the article.

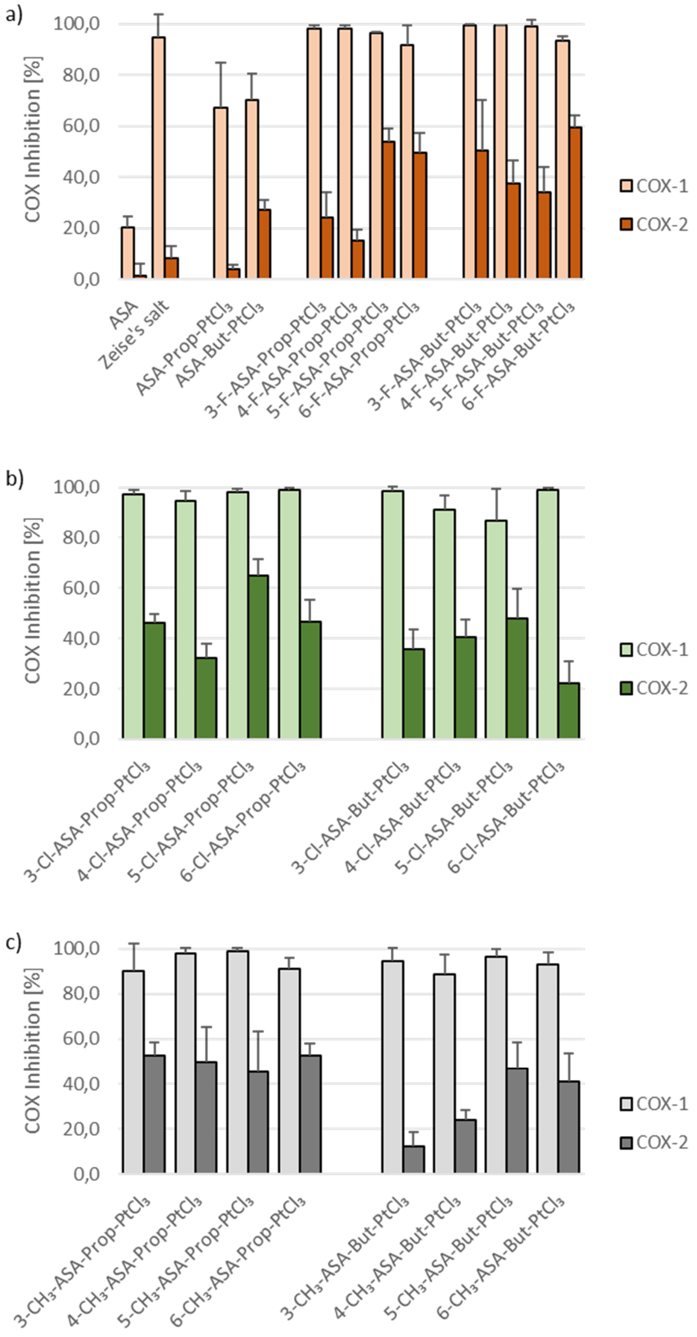

Figure S96: Inhibition of the isolated ovine/human recombinant COX-1 and COX-2 isoenzymes by a) ASA, Zeise's salt, and the fluorinated derivatives, b) the chlorinated derivatives, and c) the methylated derivatives at 10 μM; dissolved in MeOH; incubation time: 10 min.
